# Supplementary material for: The RhoB p.S73F mutation leads to cerebral palsy through dysregulation of lipid homeostasis
Source: EMBO Mol Med. 2024 Jul 30;16(9):3. doi: 10.1038/s44321-024-00113-2 (PMC11393352; doi:10.1038/s44321-024-00113-2)
Supplement: Supplementary file 2 — Appendix [file 44321_2024_113_MOESM2_ESM.pdf]

Table of Content

Appendix Table S1 ... 2
Appendix Table S2 ... 2
Appendix Table S3 ... 2
Appendix Table S4 ... 2
Appendix Table S5 ... 2
Appendix Table S6 ... 3
Appendix Table S7 ... 3
Appendix Table S8 ... 3
Appendix Table S9 ... 3
Appendix Table S10 ... 3
Appendix Table S11 ... 4
Appendix Table S12 ... 4
Appendix Table S13 ... 4
Appendix Table S14 ... 4
Appendix Table S15 ... 4
Appendix Table S16 ... 5
Appendix Table S17 ... 5
Appendix Table S18 ... 5
Appendix Table S19 ... 5
Appendix Table S20 ... 6
Appendix Table S21 ... 6
Appendix Table S22 ... 6
Appendix Table S23 ... 6
Appendix Table S24 ... 7
Appendix Table S25 ... 7
Appendix Table S26 ... 7
Appendix Table S27 ... 7
Appendix Table S28 ... 8
Appendix Table S29 ... 8
Appendix Table S30 ... 8
Appendix Table S31 ... 8
Appendix Table S32 ... 9
Appendix Table S33 ... 9
Appendix Table S34 ... 9
Appendix Table S35 ... 9
Appendix Table S36 ... 9
Appendix Table S37 ... 10
Appendix Table S38 ... 10
Appendix Table S39 ... 10

Appendix Table S1

Mean values and significance levels for Figure 1G

Comparison	Mean 1	Mean 2	Summary	P Value
WT vs RhoB$^{S73F/S73F}$	24.92	8.380	****	P=1.63^{E-08}
WT vs RhoB$^{S73F/S73F}$	8.236	4.988	****	P=1.45^{E-06}
WT vs RhoB$^{S73F/S73F}$	12.18	7.112	****	P=7.96^{E-07}

Appendix Table S2

Mean values and significance levels for Figure 1H

Comparison	Mean 1	Mean 2	Summary	P Value
WT vs RhoB$^{S73F/S73F}$	2.498	0.6400	***	P=9.91^{E-04}
WT vs RhoB$^{S73F/S73F}$	8404	2162	***	P=9.96^{E-04}

Appendix Table S3

Mean values and significance levels for Figure 1I

Comparison	Mean 1	Mean 2	Summary	P Value
WT vs RhoB$^{S73F/S73F}$	0.3720	1.358	****	P=2.10^{E-06}

Appendix Table S4

Mean values and significance levels for Figure 1J

Comparison	Mean 1	Mean 2	Summary	P Value
WT vs RhoB$^{S73F/S73F}$	0.4947	0.1584	****	P=1.58^{E-06}

Appendix Table S5

Mean values and significance levels for Figure 1K

Comparison	Mean 1	Mean 2	Summary	P Value

WT vs RhoB$^{S73F/S73F}$	23.51	60.00	***	P=8.17^{E-04}

Appendix Table S6

Mean values and significance levels for Figure 2B

Comparison	Mean 1	Mean 2	Summary	P Value
WT vs RhoB$^{S73F/S73F}$	327.8	596.0	****	P=3.34^{E-05}

Appendix Table S7

Mean values and significance levels for Figure 2C

Comparison	Mean 1	Mean 2	Summary	P Value
WT vs RhoB$^{S73F/S73F}$	1.000	0.6559	**	P=2.17^{E-03}

Appendix Table S8

Mean values and significance levels for Figure 2D

Comparison	Mean 1	Mean 2	Summary	P Value
WT vs RhoB$^{S73F/S73F}$	1.000	0.06411	****	P=7.37^{E-054}

Appendix Table S9

Mean values and significance levels for Figure 2F

Comparison	Mean 1	Mean 2	Summary	P Value
WT vs RhoB$^{S73F/S73F}$	0.6935	0.3353	*	P=2.79^{E-02}

Appendix Table S10

Mean values and significance levels for Figure 2G

Comparison	Mean 1	Mean 2	Summary	P Value
WT vs RhoB$^{S73F/S73F}$	104.2	78.29	**	P=2.31^{E-03}

Appendix Table S11

Mean values and significance levels for Figure 3C

Comparison	Mean 1	Mean 2	Summary	P Value
WT vs RhoB$^{S73F/S73F}$	1.000	0.8133	***	P=3.34^{E-04}

Appendix Table S12

Mean values and significance levels for Figure 3E

Comparison	Mean 1	Mean 2	Summary	P Value
WT vs RhoB$^{S73F/S73F}$	1.000	0.8358	**	P=1.07^{E-03}

Appendix Table S13

Mean values and significance levels for Figure 3F

Comparison	Mean 1	Mean 2	Summary	P Value
WT vs RhoB$^{S73F/S73F}$	1.000	0.6557	**	P=2.79^{E-03}

Appendix Table S14

Mean values and significance levels for Figure 3G

Comparison	Mean 1	Mean 2	Summary	P Value
WT vs RhoB$^{S73F/S73F}$	1.000	0.6362	*	P=3.04^{E-02}

Appendix Table S15

Mean values and significance levels for Figure 3H

Comparison	Mean 1	Mean 2	Summary	P Value
WT vs RhoB$^{S73F/S73F}$	1.000	0.7782	**	P=7.34^{E-03}

Appendix Table S16

Mean values and significance levels for Figure 4B

**Comparison**	**Mean 1**	**Mean 2**	**Summary**	**P Value**
WT vs RhoB$^{S73F/S73F}$	36.59	65.06	*	P=3.15^{E-02}
WT vs RhoB$^{S73F/S73F}$	21.23	46.34	*	P=2.01^{E-02}

Appendix Table S17

Mean values and significance levels for Figure 4C

**Comparison**	**Mean 1**	**Mean 2**	**Summary**	**P Value**
WT vs RhoB$^{S73F/S73F}$	0.8936	1.508	**	P=2.73^{E-03}
WT vs RhoB$^{S73F/S73F}$	1.207	1.862	***	P=3.11^{E-04}
WT vs RhoB$^{S73F/S73F}$	1.166	1.794	***	P=8.67^{E-04}

Appendix Table S18

Mean values and significance levels for Figure 4D

**Comparison**	**Mean 1**	**Mean 2**	**Summary**	**P Value**
WT vs RhoB$^{S73F/S73F}$	4.472	8.600	*	P=1.19^{E-02}
WT vs RhoB$^{S73F/S73F}$	6.192	10.84	**	P=1.96^{E-03}
WT vs RhoB$^{S73F/S73F}$	7.396	13.42	*	P=1.04^{E-02}

Appendix Table S19

Mean values and significance levels for Figure 4E

**Comparison**	**Mean 1**	**Mean 2**	**Summary**	**P Value**
WT vs RhoB$^{S73F/S73F}$	1.000	1.804	*	P=1.87^{E-02}
WT vs RhoB$^{S73F/S73F}$	1.000	5.898	*	P=1.01^{E-02}
WT vs RhoB$^{S73F/S73F}$	1.000	5.225	**	P=2.44^{E-03}

Appendix Table S20

Mean values and significance levels for Figure 4F

Comparison	Mean 1	Mean 2	Summary	P Value
WT vs RhoB$^{S73F/S73F}$	1.000	1.435	*	P=1.20^{E-02}
WT vs RhoB$^{S73F/S73F}$	1.000	1.688	**	P=1.01^{E-03}
WT vs RhoB$^{S73F/S73F}$	1.000	1.478	*	P=4.89^{E-02}

Appendix Table S21

Mean values and significance levels for Figure 4G

Comparison	Mean 1	Mean 2	Summary	P Value
WT vs RhoB$^{S73F/S73F}$	1.000	1.735	*	P=4.99^{E-02}
WT vs RhoB$^{S73F/S73F}$	1.000	1.486	**	P=4.35^{E-03}
WT vs RhoB$^{S73F/S73F}$	1.000	1.963	*	P=4.59^{E-02}

Appendix Table S22

Mean values and significance levels for Figure 5D

Comparison	Mean 1	Mean 2	Summary	P Value
WT vs RhoB$^{S73F/S73F}$	1.000	1.434	*	P=1.09^{E-02}
WT vs RhoB$^{S73F/S73F}$	1.000	1.387	****	P=9.14^{E-05}
WT vs RhoB$^{S73F/S73F}$	1.000	1.388	*	P=2.08^{E-02}

Appendix Table S23

Mean values and significance levels for Figure 5E

Comparison	Mean 1	Mean 2	Summary	P Value
WT vs RhoB$^{S73F/S73F}$	1.000	1.157	**	P=4.24^{E-03}
WT vs RhoB$^{S73F/S73F}$	1.000	1.419	*	P=1.73^{E-02}
WT vs RhoB$^{S73F/S73F}$	1.000	1.308	*	P=1.23^{E-02}

Appendix Table S24

Mean values and significance levels for Figure 5F

Comparison	Mean 1	Mean 2	Summary	Adjusted P Value
WT vs WT+LYN	1.000	1.292	*	$P=4.98^{E-02}$
WT vs WT+LYN Y397D	1.000	1.836	***	$P=2.92^{E-04}$

Appendix Table S25

Mean values and significance levels for Figure 5G

Comparison	Mean 1	Mean 2	Summary	Adjusted P Value
WT vs WT-LYN	1.000	0.6090	*	$P=3.02^{E-02}$
$RhoB^{S73F/S73F}$ vs $RhoB^{S73F/S73F}$-LYN	1.398	0.9341	*	$P=1.23^{E-02}$

Appendix Table S26

Mean values and significance levels for Figure 6B

Comparison	Mean 1	Mean 2	Summary	Adjusted P Value
WT vs $RhoB^{S73F/S73F}$	77.76	163.7	****	$P=3.00^{E-06}$
$RhoB^{S73F/S73F}$ vs $RhoB^{S73F/S73F}$+ATV	163.7	85.95	****	$P=6.39^{E-06}$

Appendix Table S27

Mean values and significance levels for Figure 6D

Comparison	Mean 1	Mean 2	Summary	Adjusted P Value
WT vs $RhoB^{S73F/S73F}$	1.204	1.685	***	$P=1.87^{E-04}$
$RhoB^{S73F/S73F}$ vs $RhoB^{S73F/S73F}$+ATV	1.685	1.411	**	$P=7.53^{E-03}$

Appendix Table S28

Mean values and significance levels for Figure 6E

**Comparison**	**Mean 1**	**Mean 2**	**Summary**	**Adjusted P Value**
WT vs $RhoB^{S73F/S73F}$	0.4773	0.5271	*	$P=1.21^{E-02}$
$RhoB^{S73F/S73F}$ vs $RhoB^{S73F/S73F}$+ATV	0.5271	0.4828	*	$P=3.02^{E-02}$

Appendix Table S29

Mean values and significance levels for Figure 6F

**Comparison**	**Mean 1**	**Mean 2**	**Summary**	**Adjusted P Value**
WT vs $RhoB^{S73F/S73F}$	1.000	1.874	**	$P=2.20^{E-03}$
$RhoB^{S73F/S73F}$ vs $RhoB^{S73F/S73F}$+ATV	1.874	1.338	*	$P=3.53^{E-02}$

Appendix Table S30

Mean values and significance levels for Figure 6G

**Comparison**	**Mean 1**	**Mean 2**	**Summary**	**Adjusted P Value**
WT vs $RhoB^{S73F/S73F}$	1.000	1.841	***	$P=5.73^{E-04}$
$RhoB^{S73F/S73F}$ vs $RhoB^{S73F/S73F}$+ATV	1.841	1.340	*	$P=1.45^{E-02}$

Appendix Table S31

Mean values and significance levels for Figure 6H

**Comparison**	**Mean 1**	**Mean 2**	**Summary**	**Adjusted P Value**
WT vs $RhoB^{S73F/S73F}$	1.000	2.082	****	$P=6.66^{E-05}$
$RhoB^{S73F/S73F}$ vs $RhoB^{S73F/S73F}$+ATV	2.082	1.625	*	$P=1.82^{E-02}$

Appendix Table S32

Mean values and significance levels for Figure 6I

**Comparison**	**Mean 1**	**Mean 2**	**Summary**	**Adjusted P Value**
WT vs $RhoB^{S73F/S73F}$	1.000	0.5782	***	$P=1.28^{E-04}$
$RhoB^{S73F/S73F}$ vs $RhoB^{S73F/S73F}$+ATV	0.5782	0.7551	*	$P=2.99^{E-02}$

Appendix Table S33

Mean values and significance levels for Figure EV1I

**Comparison**	**Mean 1**	**Mean 2**	**Summary**	**P Value**
WT vs $RhoB^{S73F/S73F}$	24.06	12.79	****	$P=1.24^{E-06}$
WT vs $RhoB^{S73F/S73F}$	8.600	3.108	****	$P=5.75^{E-09}$
WT vs $RhoB^{S73F/S73F}$	12.20	6.624	****	$P=2.75^{E-07}$

Appendix Table S34

Mean values and significance levels for Figure EV2D

**Comparison**	**Mean 1**	**Mean 2**	**Summary**	**P Value**
WT vs $RhoB^{S73F/S73F}$	1.000	0.8543	*	$P=4.71^{E-02}$

Appendix Table S35

Mean values and significance levels for Figure EV2E

**Comparison**	**Mean 1**	**Mean 2**	**Summary**	**P Value**
WT vs $RhoB^{S73F/S73F}$	1.000	1.311	**	$P=1.95^{E-03}$

Appendix Table S36

Mean values and significance levels for Figure EV3A

**Comparison**	**Mean 1**	**Mean 2**	**Summary**	**P Value**

WT vs $RhoB^{S73F/S73F}$	1.000	1.493	*	P=4.48^{E-02}
WT vs $RhoB^{S73F/S73F}$	1.000	1.797	*	P=3.49^{E-02}
WT vs $RhoB^{S73F/S73F}$	1.000	1.224	*	P=3.37^{E-02}

Appendix Table S37

Mean values and significance levels for Figure EV3B

Comparison	Mean 1	Mean 2	Summary	P Value
WT vs $RhoB^{S73F/S73F}$	1.000	1.320	**	P=3.34^{E-03}

Appendix Table S38

Mean values and significance levels for Figure EV3F

Comparison	Mean 1	Mean 2	Summary	P Value
WT vs $RhoB^{S73F/S73F}$	1.000	1.338	**	P=5.03^{E-03}

Appendix Table S39

Mean values and significance levels for Figure EV3G

Comparison	Mean 1	Mean 2	Summary	P Value
WT vs $RhoB^{S73F/S73F}$	1.000	1.218	***	2.56^{E-04}
