# Supplementary material for: The RhoB p.S73F mutation leads to cerebral palsy through dysregulation of lipid homeostasis
Source: EMBO Mol Med. 2024 Jul 30;16(9):3. doi: 10.1038/s44321-024-00113-2 (PMC11393352; doi:10.1038/s44321-024-00113-2)
Supplement: Supplementary file 4 — Movie EV1 [file 44321_2024_113_MOESM4_ESM.zip › Movie EV1/Movie EV1 WT.docx]

Movie EV1 shows the WT Rabbit in free motion.
