# Supplementary material for: The RhoB p.S73F mutation leads to cerebral palsy through dysregulation of lipid homeostasis
Source: EMBO Mol Med. 2024 Jul 30;16(9):3. doi: 10.1038/s44321-024-00113-2 (PMC11393352; doi:10.1038/s44321-024-00113-2)
Supplement: Supplementary file 6 — Movie EV3 [file 44321_2024_113_MOESM6_ESM.zip › Movie EV3/Movie EV3 RHOB Heterozygote.docx]

Movie EV3 shows the RhoB^S73F/+^ Rabbit in free motion.
