# Supplementary material for: The RhoB p.S73F mutation leads to cerebral palsy through dysregulation of lipid homeostasis
Source: EMBO Mol Med. 2024 Jul 30;16(9):3. doi: 10.1038/s44321-024-00113-2 (PMC11393352; doi:10.1038/s44321-024-00113-2)
Supplement: Supplementary file 10 — Movie EV7 [file 44321_2024_113_MOESM10_ESM.zip › Movie EV7/Movie EV7 RHOB Homozygote.docx]

Movie EV2 shows the RhoB^S73F/S73F^ Rabbit free climbing.
