# Supplementary figures and images for: The RhoB p.S73F mutation leads to cerebral palsy through dysregulation of lipid homeostasis (part 1 of 2)
Source: EMBO Mol Med. 2024 Jul 30;16(9):3. doi: 10.1038/s44321-024-00113-2 (PMC11393352; doi:10.1038/s44321-024-00113-2)

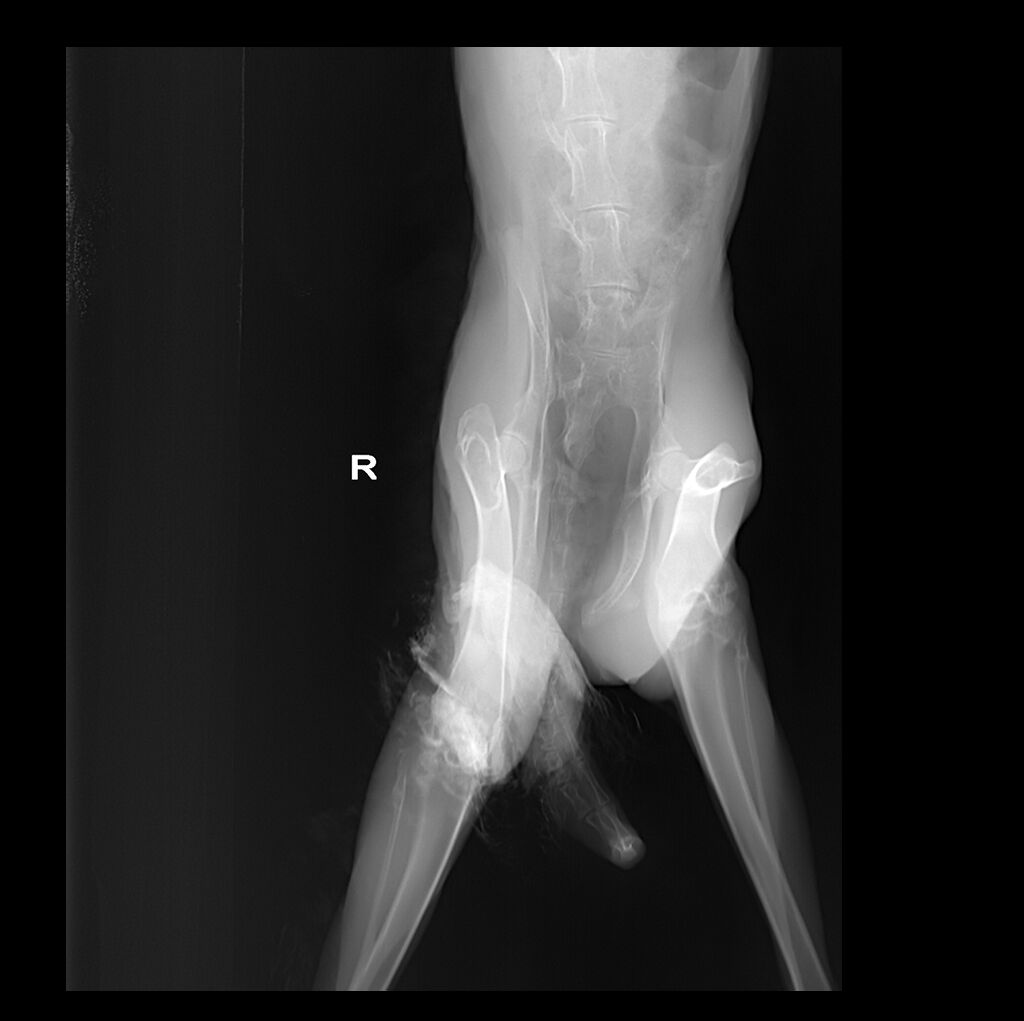

Supplement: Supplementary file 12 — Source data Fig. 2 [file 44321_2024_113_MOESM12_ESM.zip › Figure 2/2A/RHOB S73F.tif]

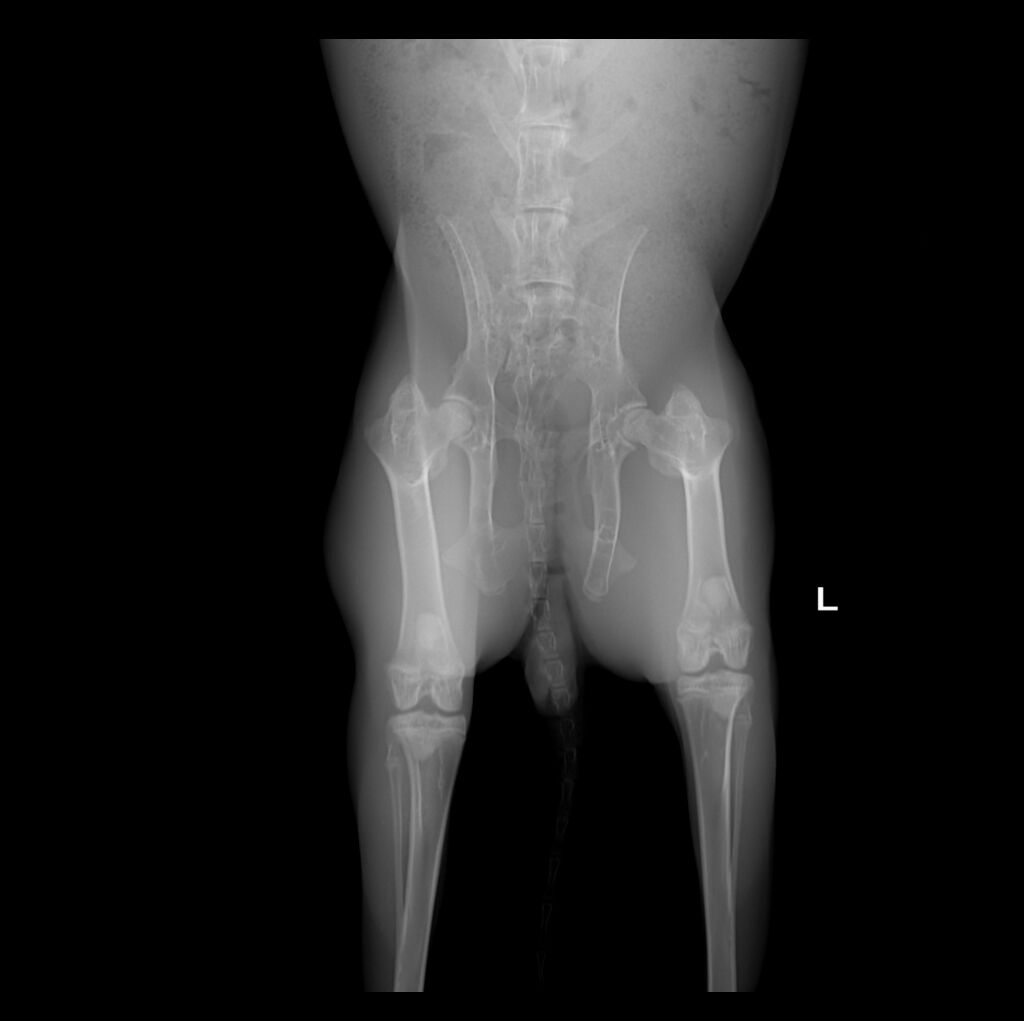

Supplement: Supplementary file 12 — Source data Fig. 2 [file 44321_2024_113_MOESM12_ESM.zip › Figure 2/2A/WT.tif]

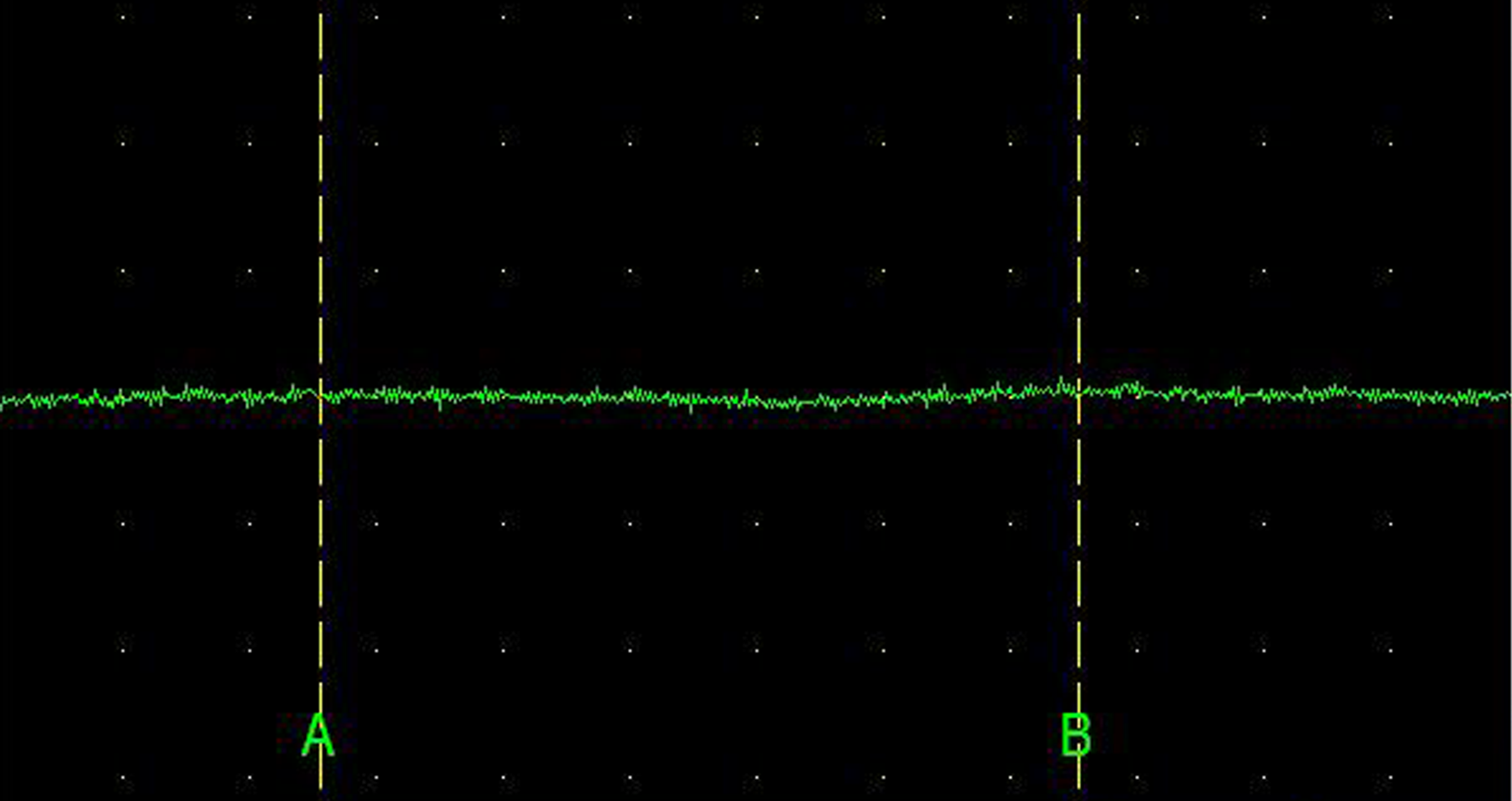

Supplement: Supplementary file 12 — Source data Fig. 2 [file 44321_2024_113_MOESM12_ESM.zip › Figure 2/2E/RHOB S73F.tif]

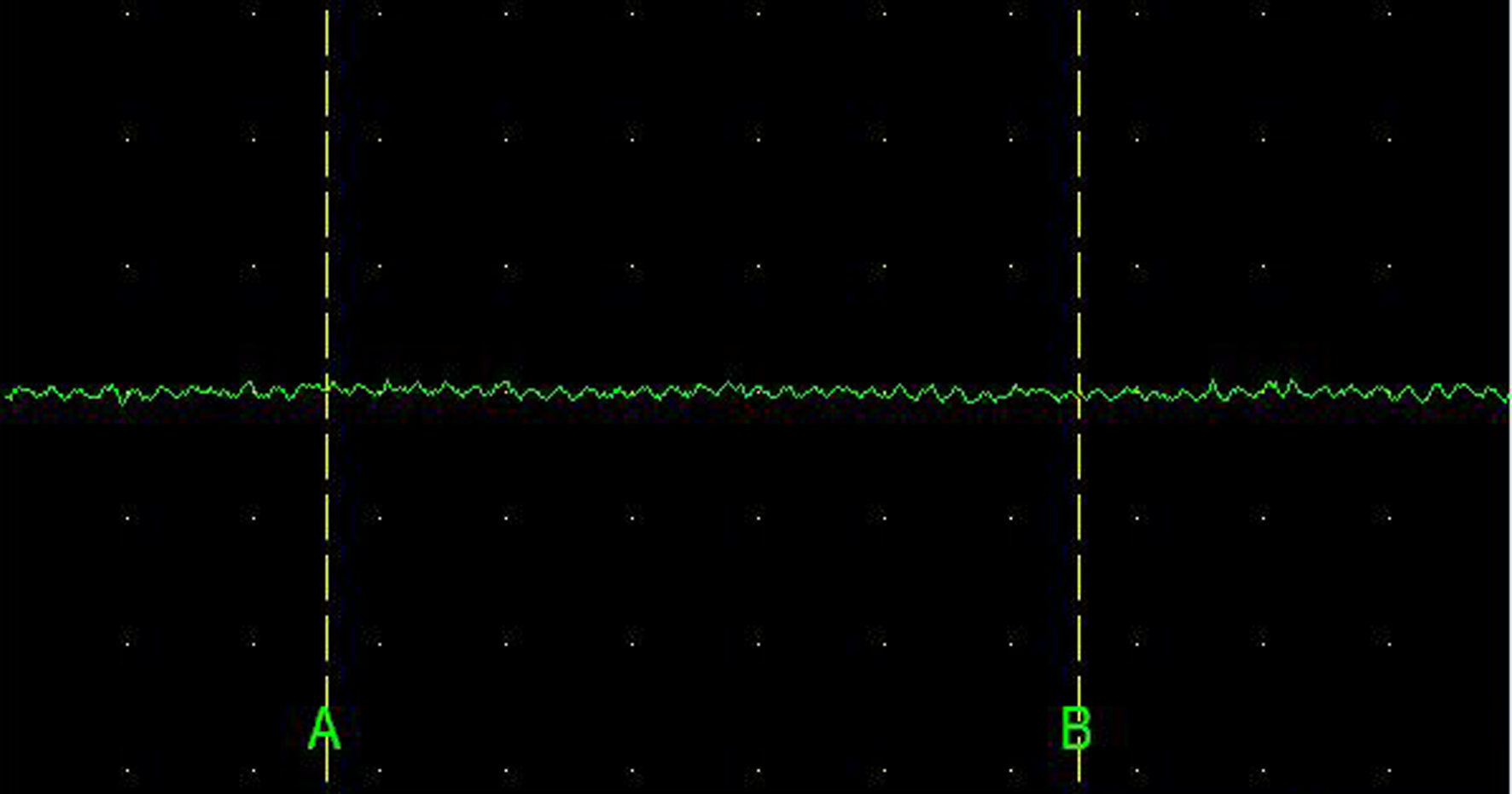

Supplement: Supplementary file 12 — Source data Fig. 2 [file 44321_2024_113_MOESM12_ESM.zip › Figure 2/2E/WT.tif]

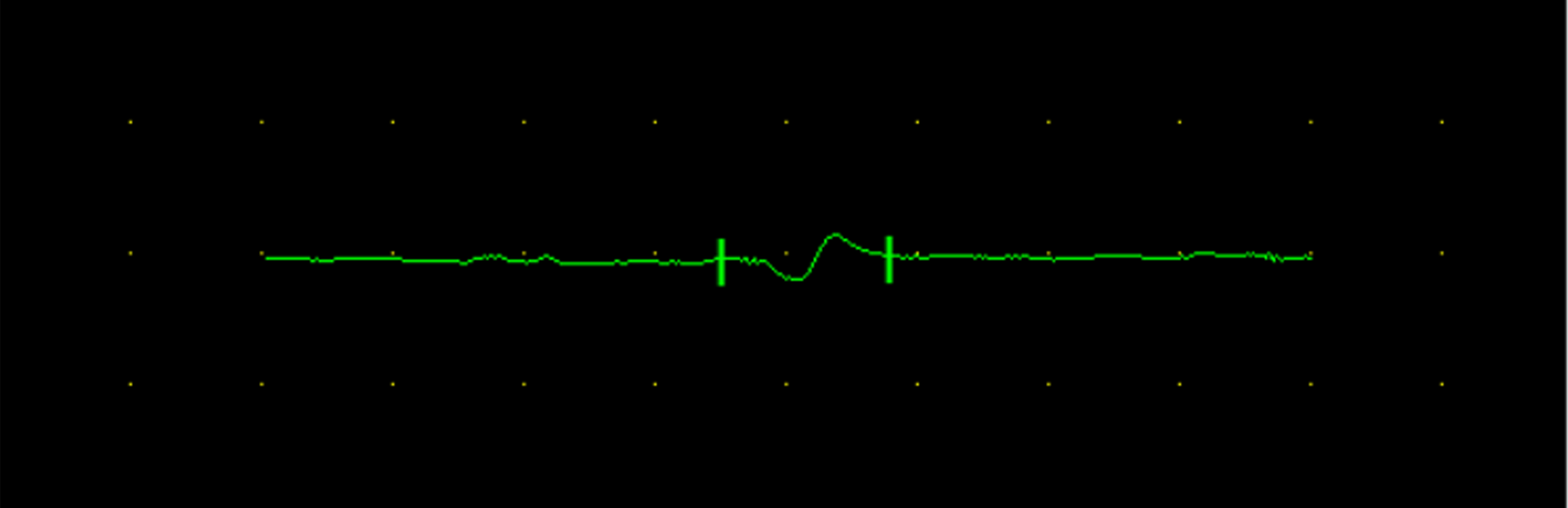

Supplement: Supplementary file 12 — Source data Fig. 2 [file 44321_2024_113_MOESM12_ESM.zip › Figure 2/2F/RHOB S73F.tif]

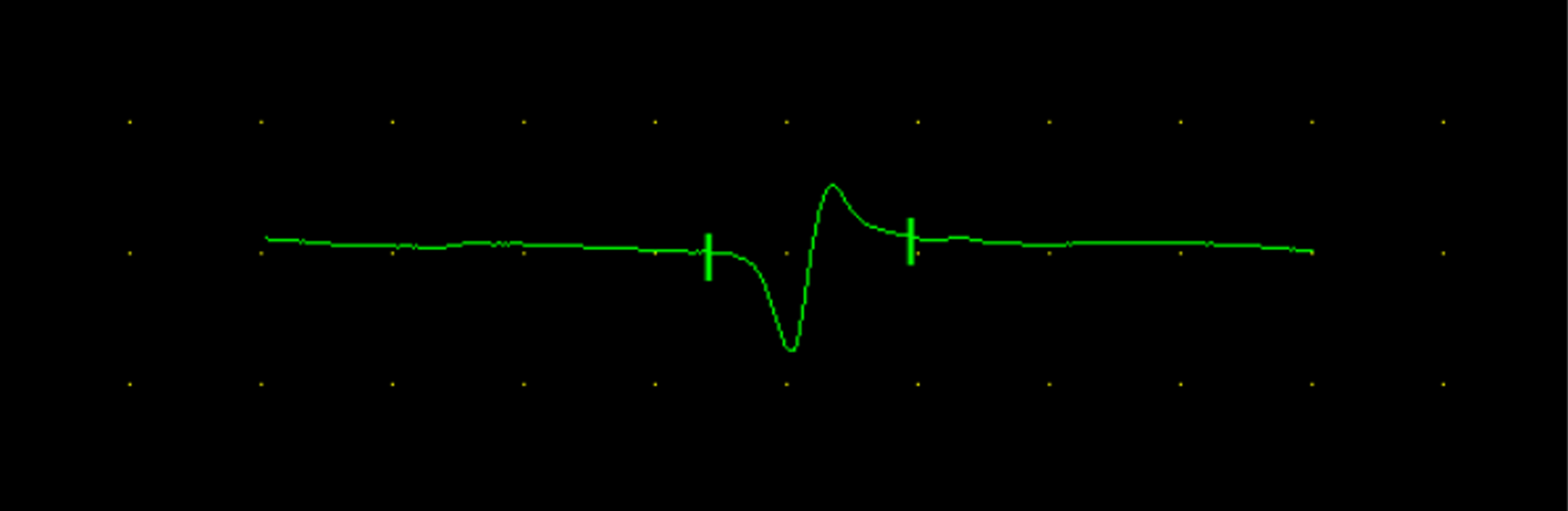

Supplement: Supplementary file 12 — Source data Fig. 2 [file 44321_2024_113_MOESM12_ESM.zip › Figure 2/2F/WT.tif]

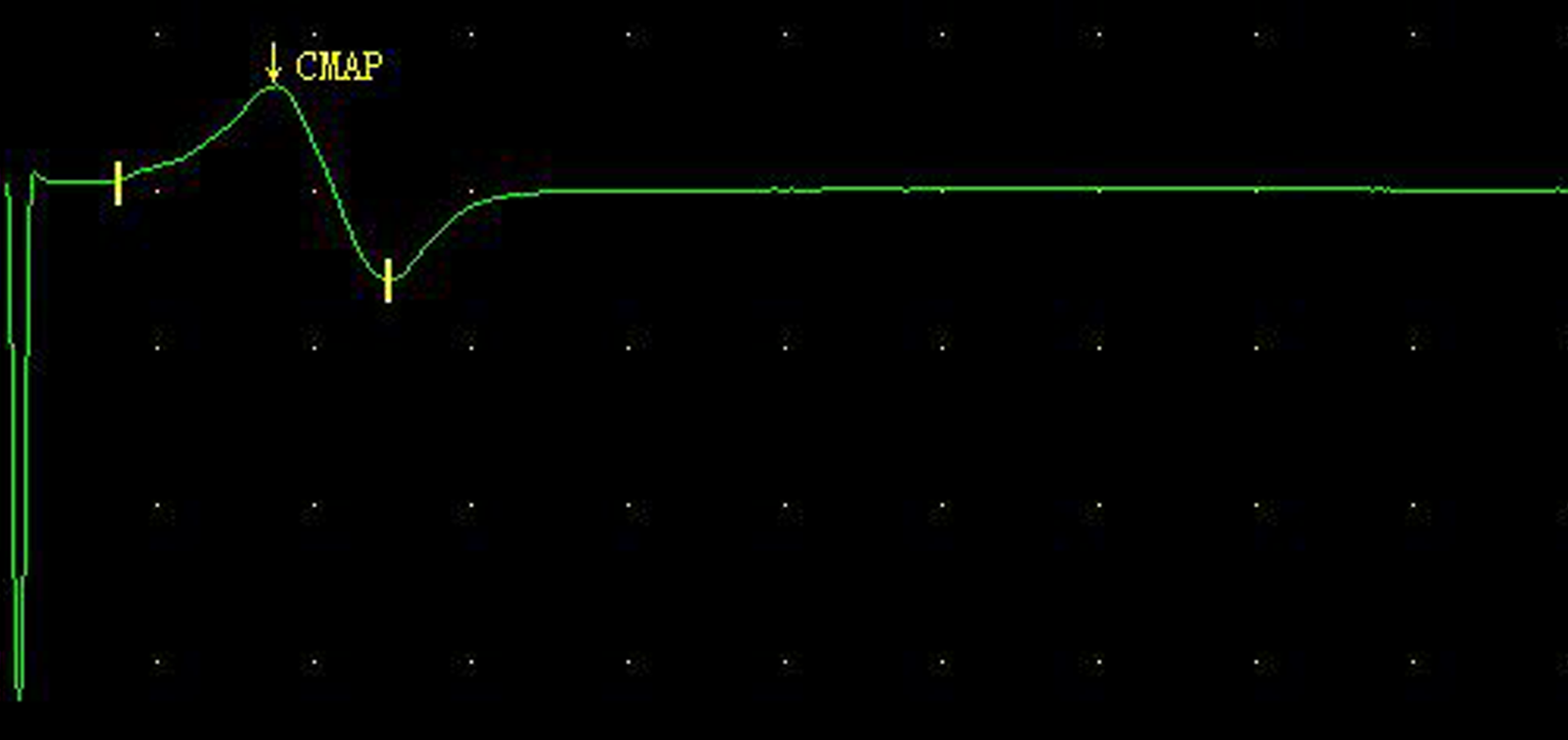

Supplement: Supplementary file 12 — Source data Fig. 2 [file 44321_2024_113_MOESM12_ESM.zip › Figure 2/2G/RHOB S73F (1).tif]

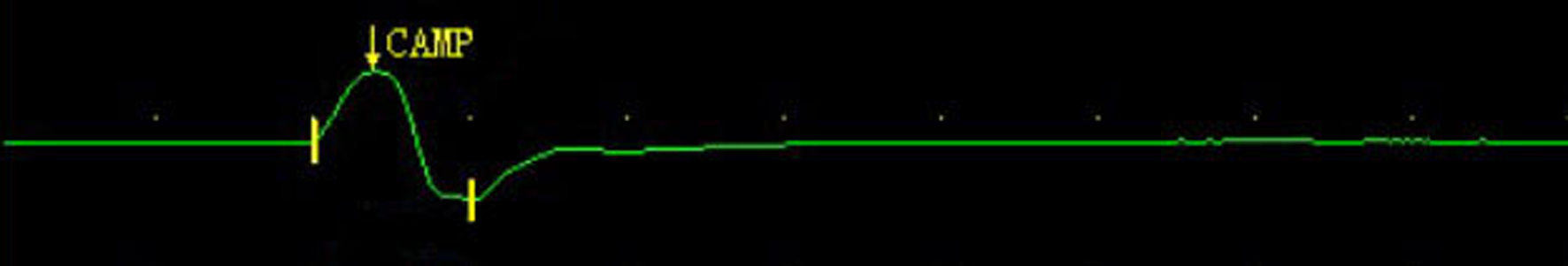

Supplement: Supplementary file 12 — Source data Fig. 2 [file 44321_2024_113_MOESM12_ESM.zip › Figure 2/2G/RHOB S73F (2).tif]

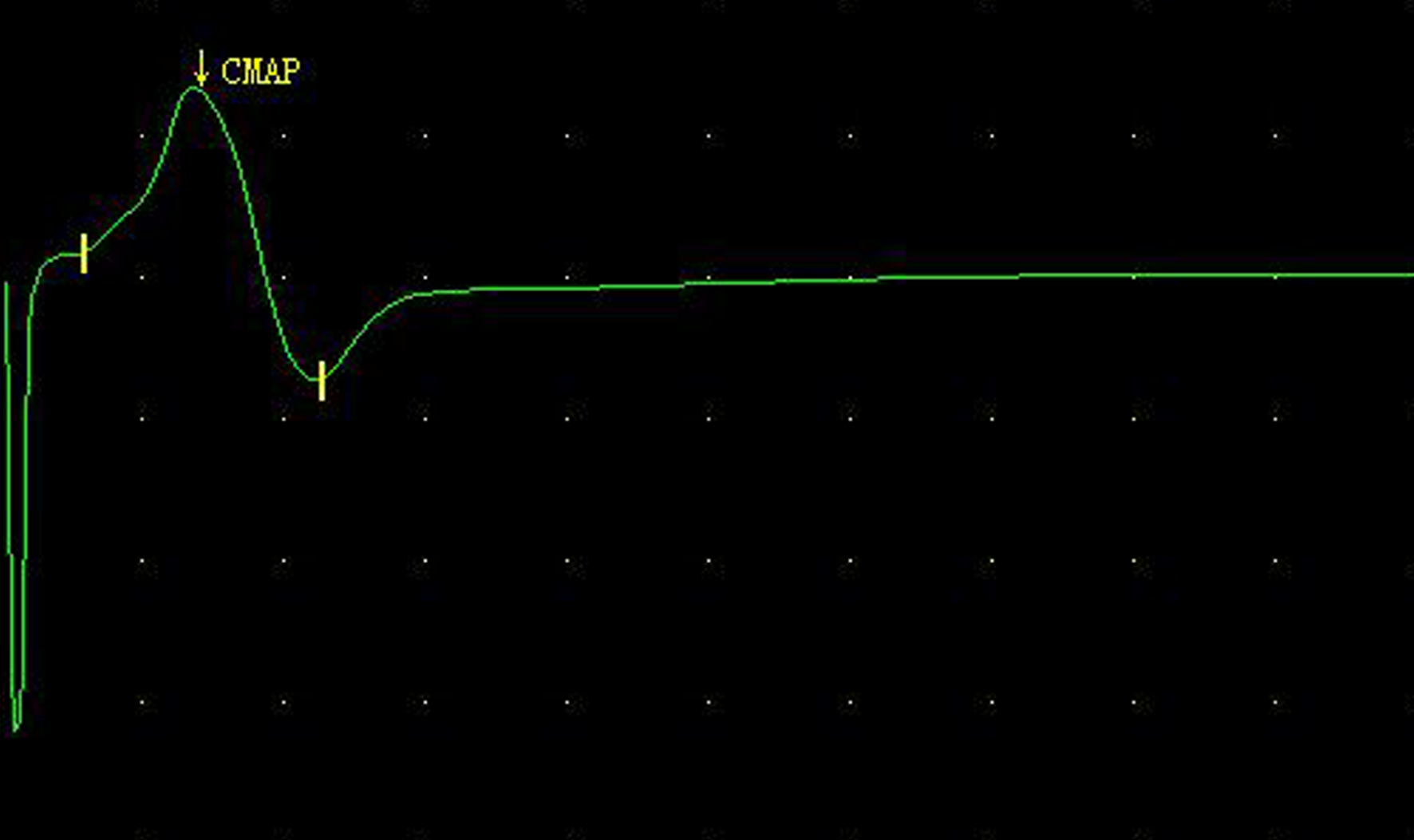

Supplement: Supplementary file 12 — Source data Fig. 2 [file 44321_2024_113_MOESM12_ESM.zip › Figure 2/2G/WT (1).tif]

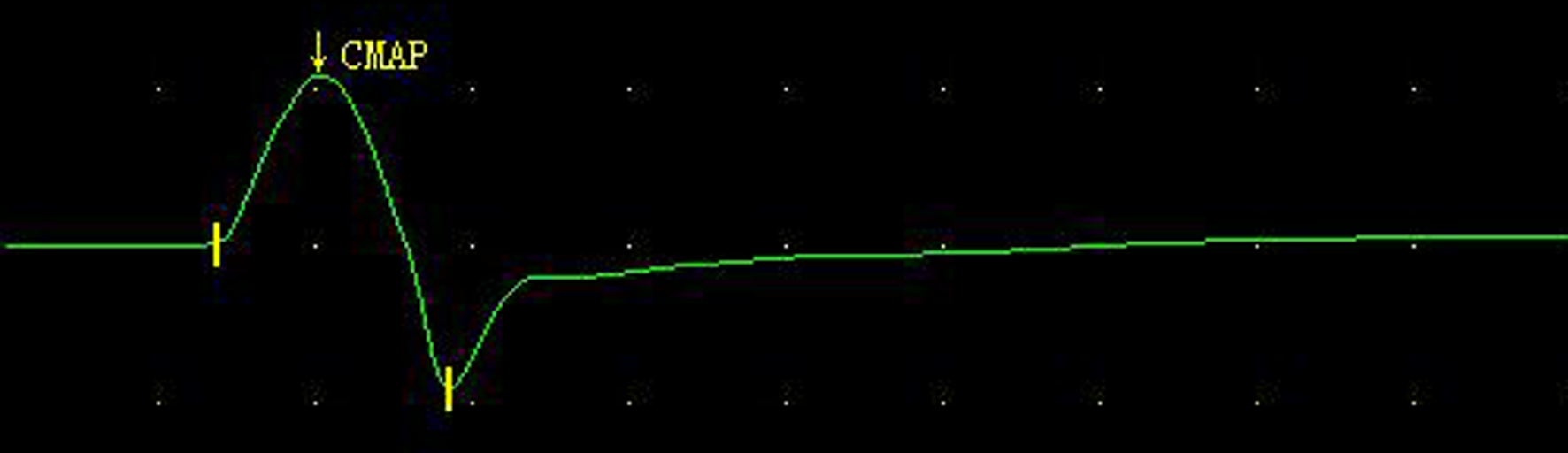

Supplement: Supplementary file 12 — Source data Fig. 2 [file 44321_2024_113_MOESM12_ESM.zip › Figure 2/2G/WT (2).tif]

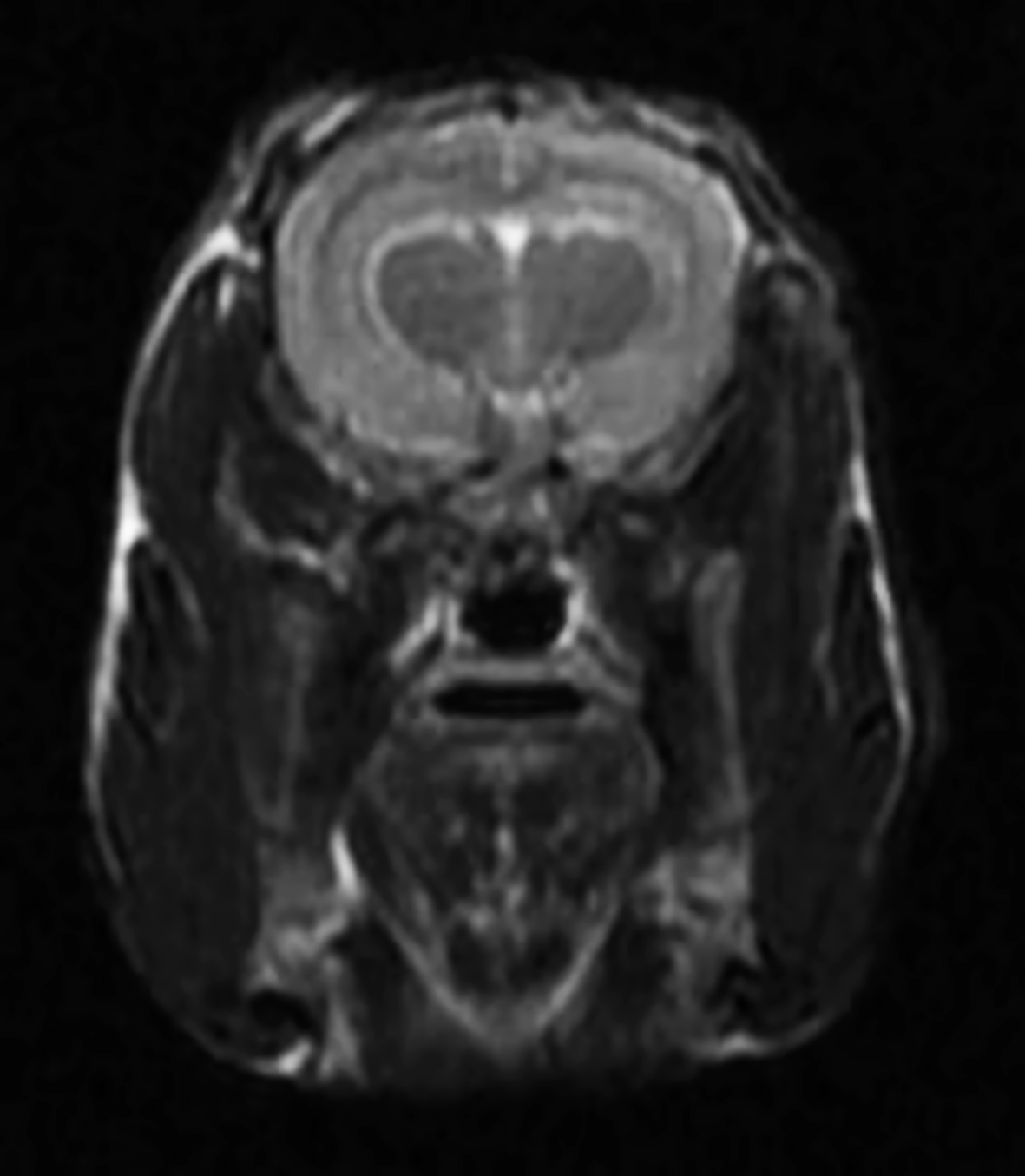

Supplement: Supplementary file 13 — Source data Fig. 3 [file 44321_2024_113_MOESM13_ESM.zip › Figure 3/3A/RHOB (1).tif]

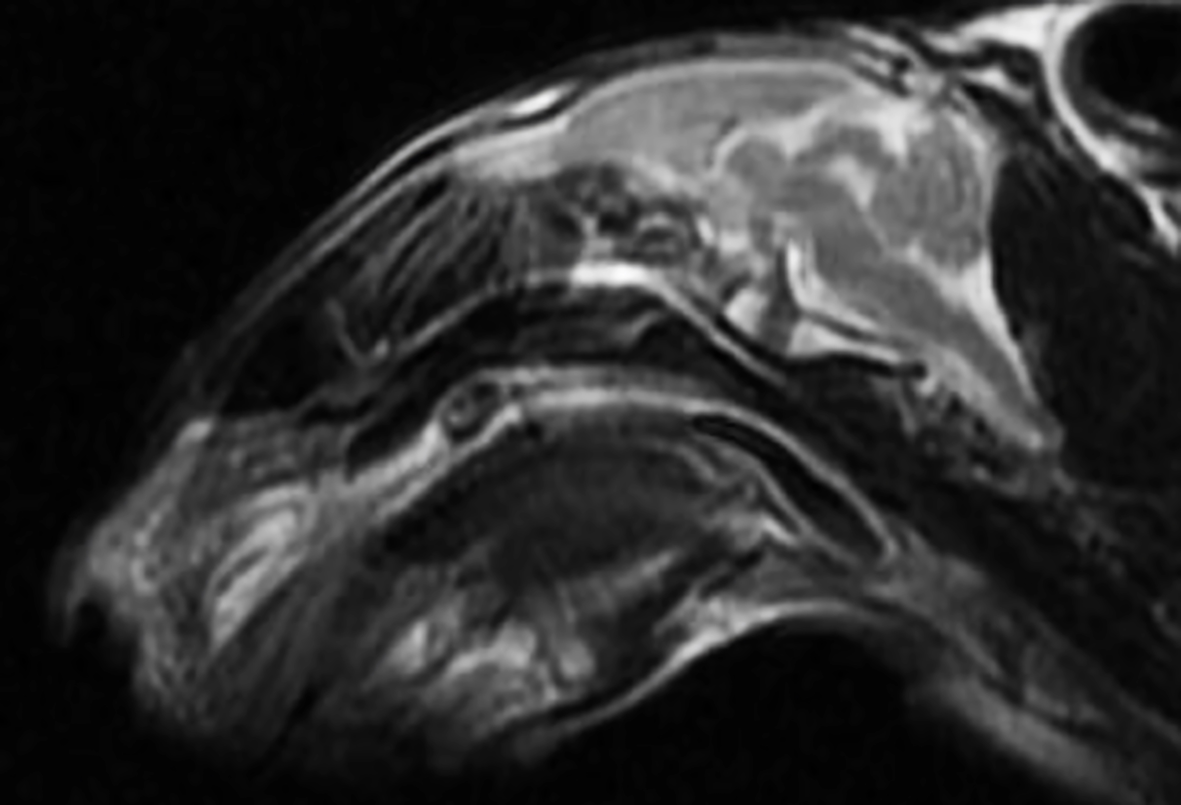

Supplement: Supplementary file 13 — Source data Fig. 3 [file 44321_2024_113_MOESM13_ESM.zip › Figure 3/3A/RHOB (2).tif]

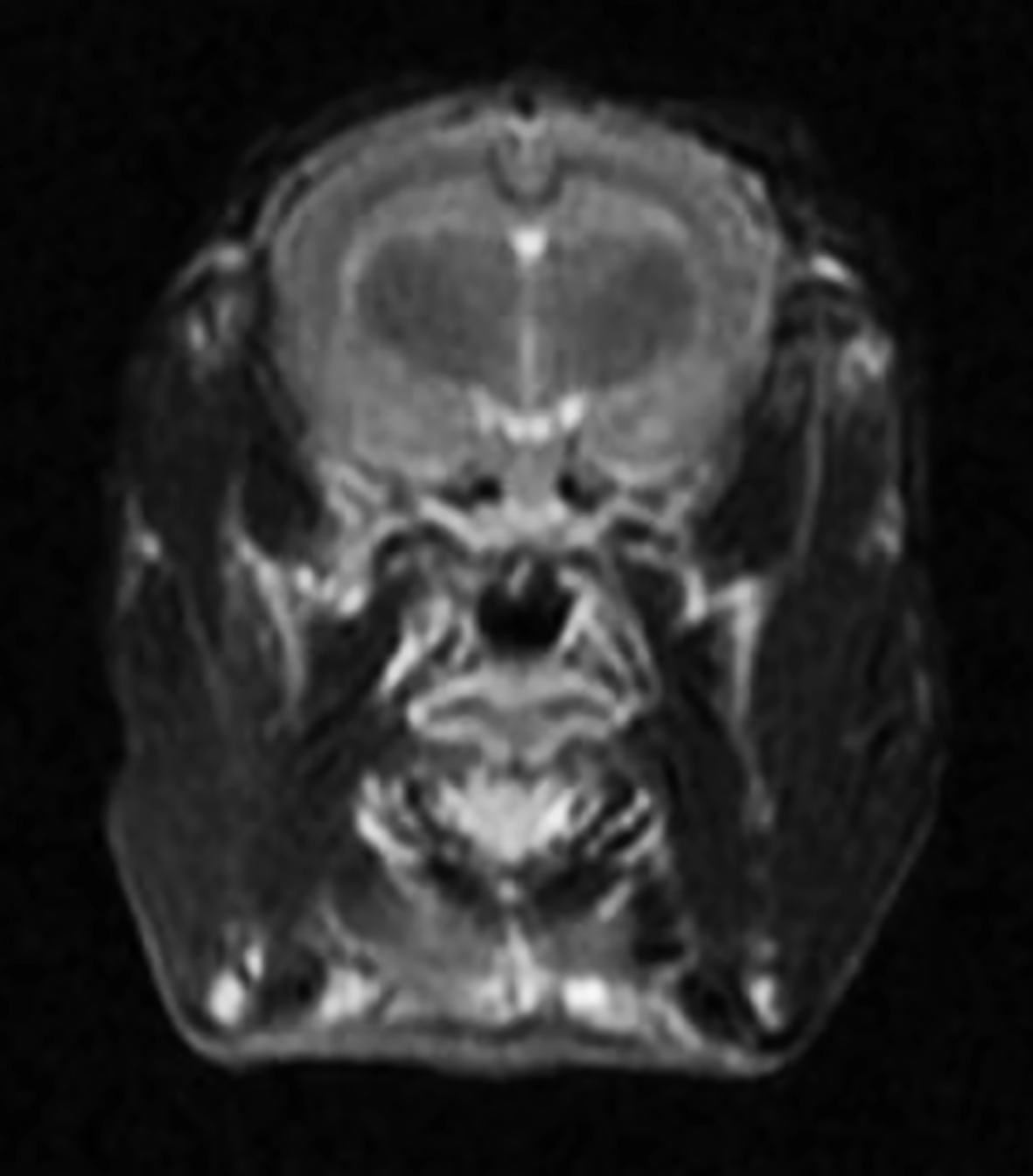

Supplement: Supplementary file 13 — Source data Fig. 3 [file 44321_2024_113_MOESM13_ESM.zip › Figure 3/3A/WT (1).tif]

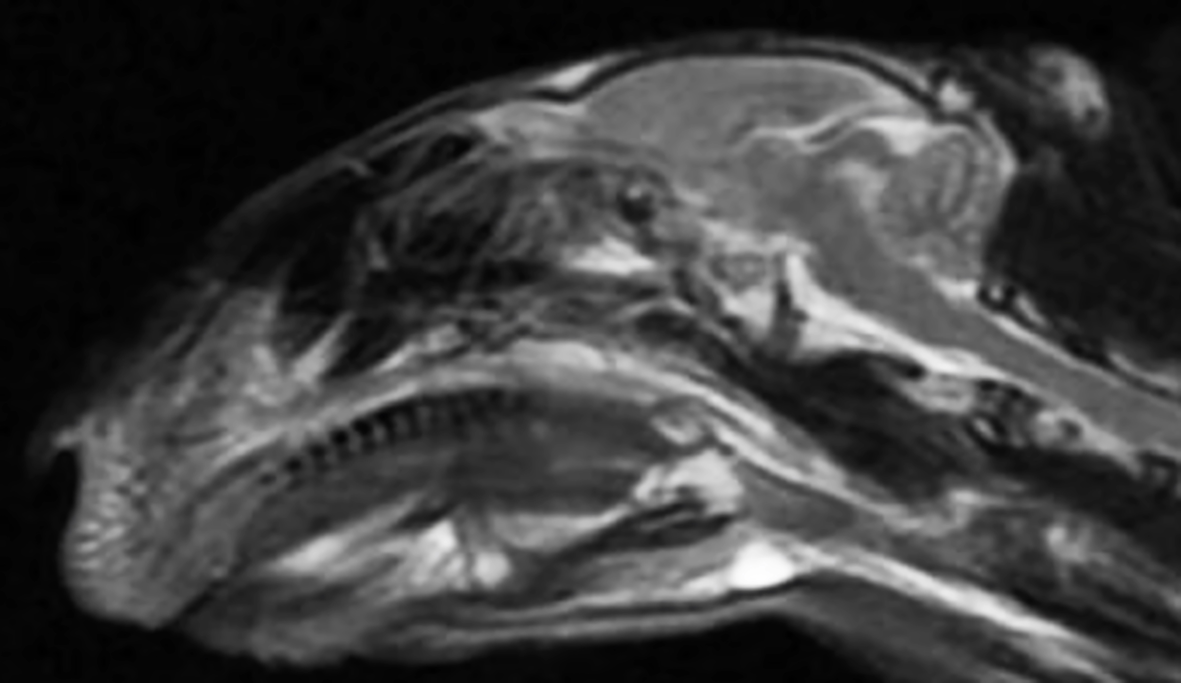

Supplement: Supplementary file 13 — Source data Fig. 3 [file 44321_2024_113_MOESM13_ESM.zip › Figure 3/3A/WT (2).tif]

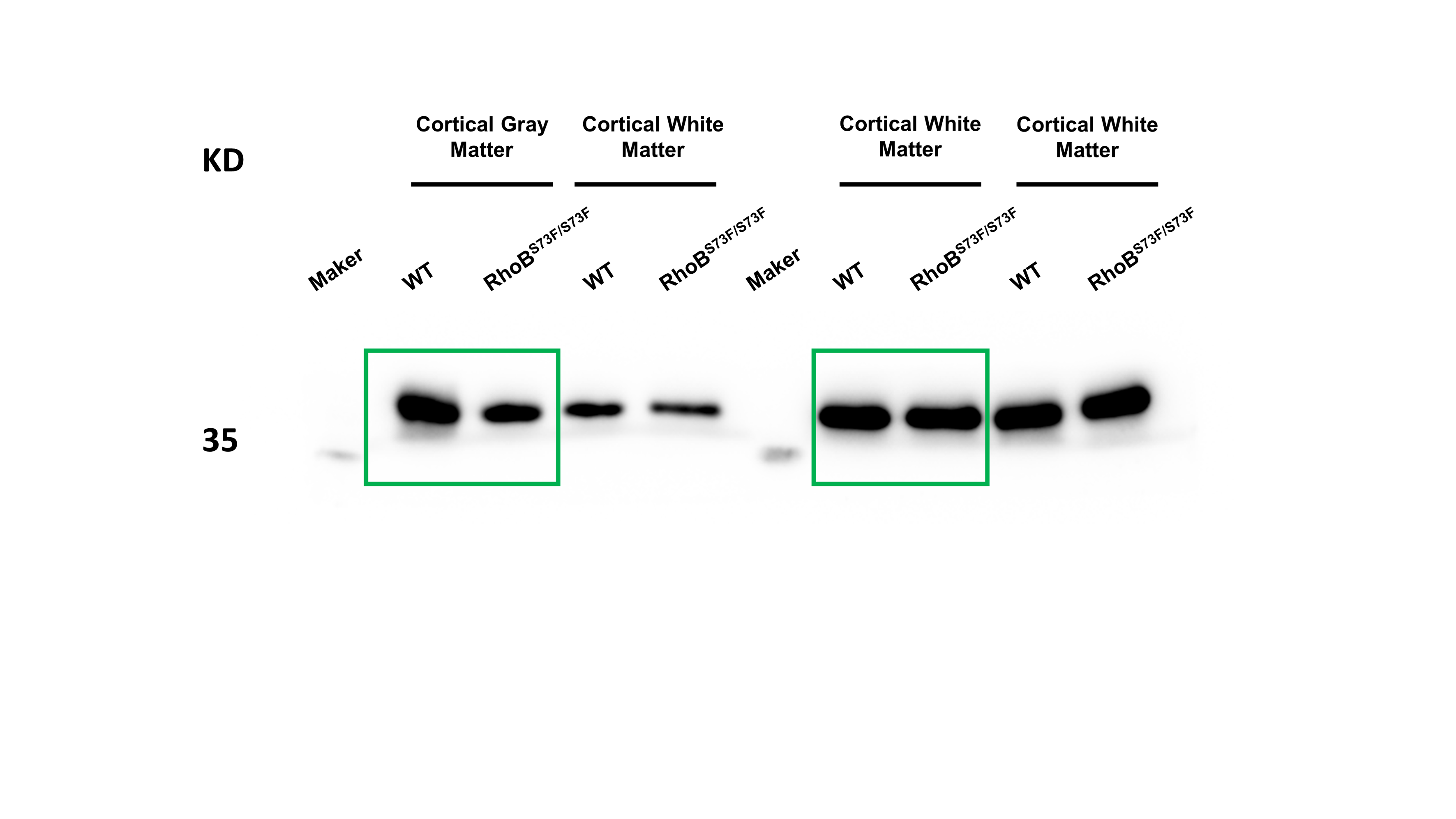

Supplement: Supplementary file 14 — Source data Fig. 4 [file 44321_2024_113_MOESM14_ESM.zip › Figure 4/4E/replicate/western GAPDH in Cortical Gray Matter&Cortical White Matter replicate.tif]

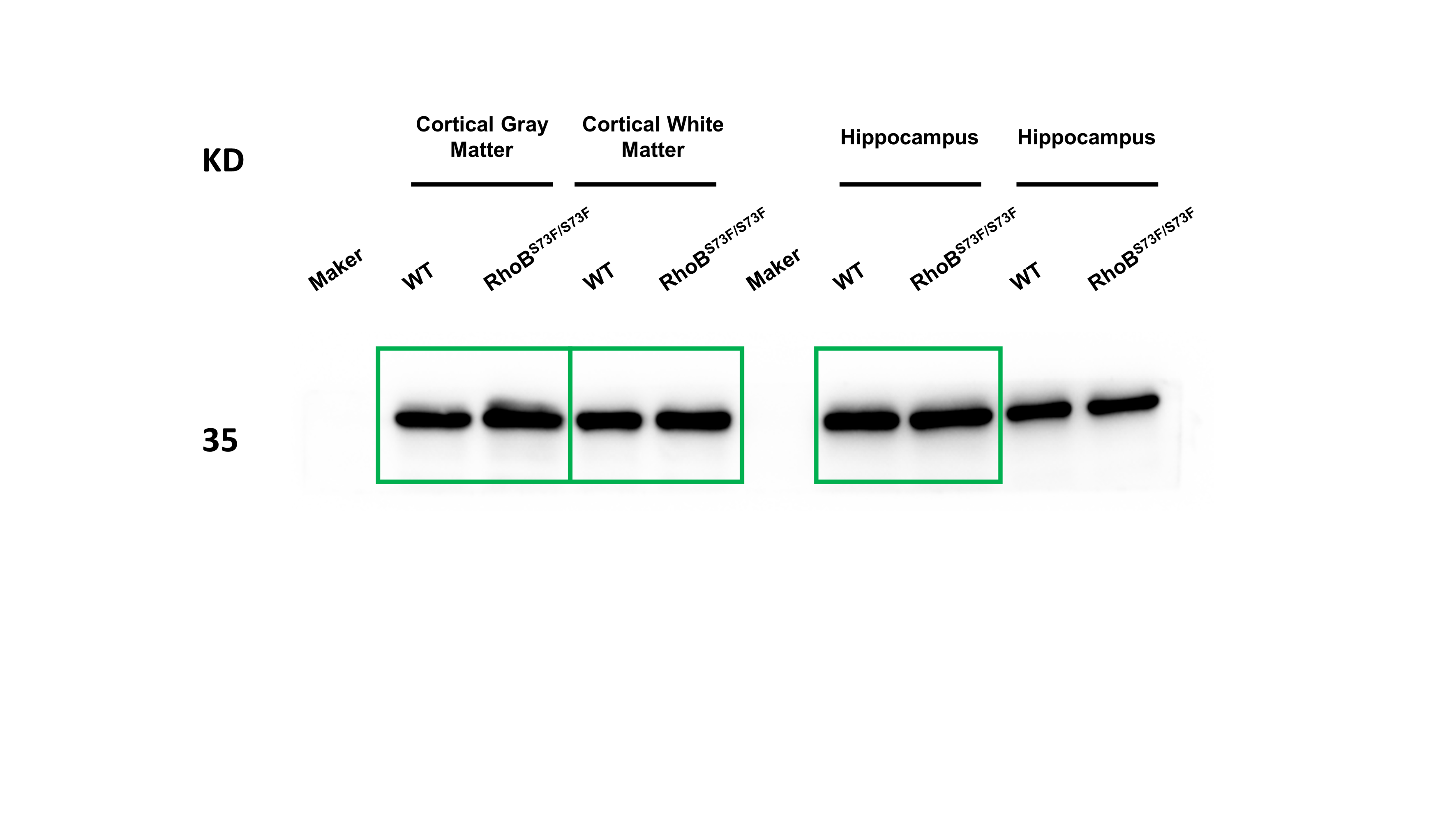

Supplement: Supplementary file 14 — Source data Fig. 4 [file 44321_2024_113_MOESM14_ESM.zip › Figure 4/4E/replicate/western Gapdh replicate.tif]

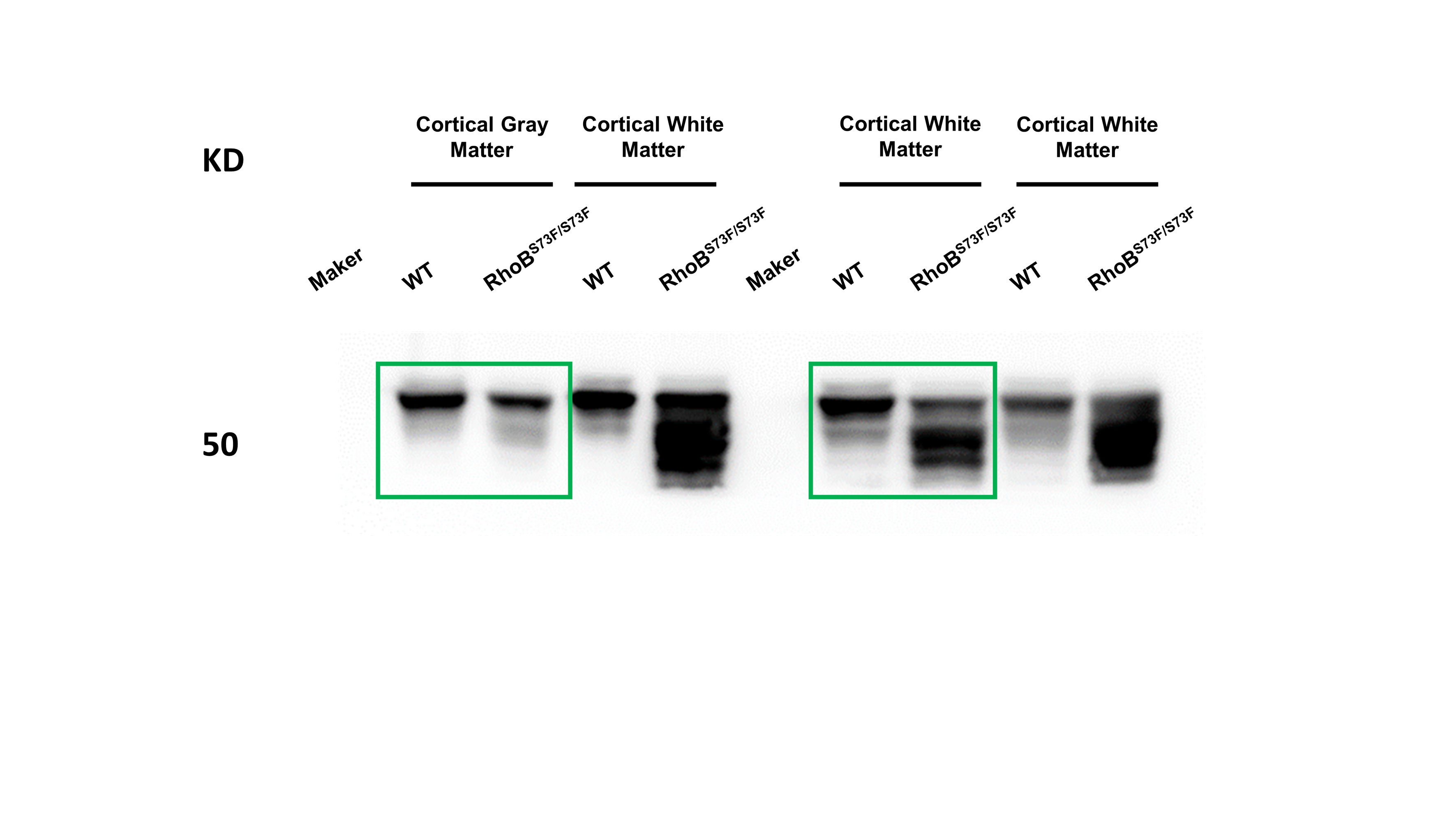

Supplement: Supplementary file 14 — Source data Fig. 4 [file 44321_2024_113_MOESM14_ESM.zip › Figure 4/4E/replicate/western GFAP in Cortical Gray Matter&Cortical White Matter replicate.tif]

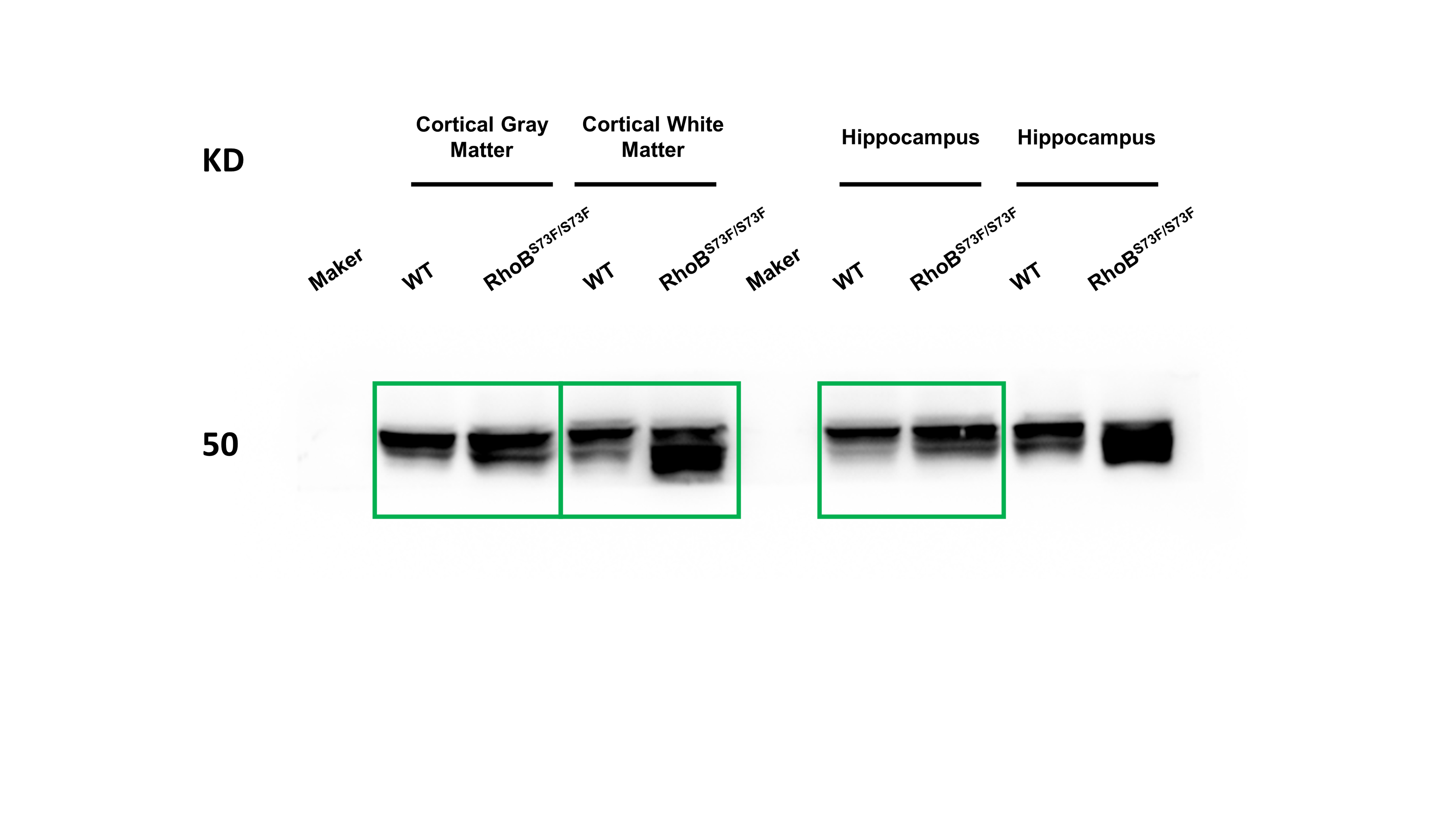

Supplement: Supplementary file 14 — Source data Fig. 4 [file 44321_2024_113_MOESM14_ESM.zip › Figure 4/4E/replicate/western GFAP replicate.tif]

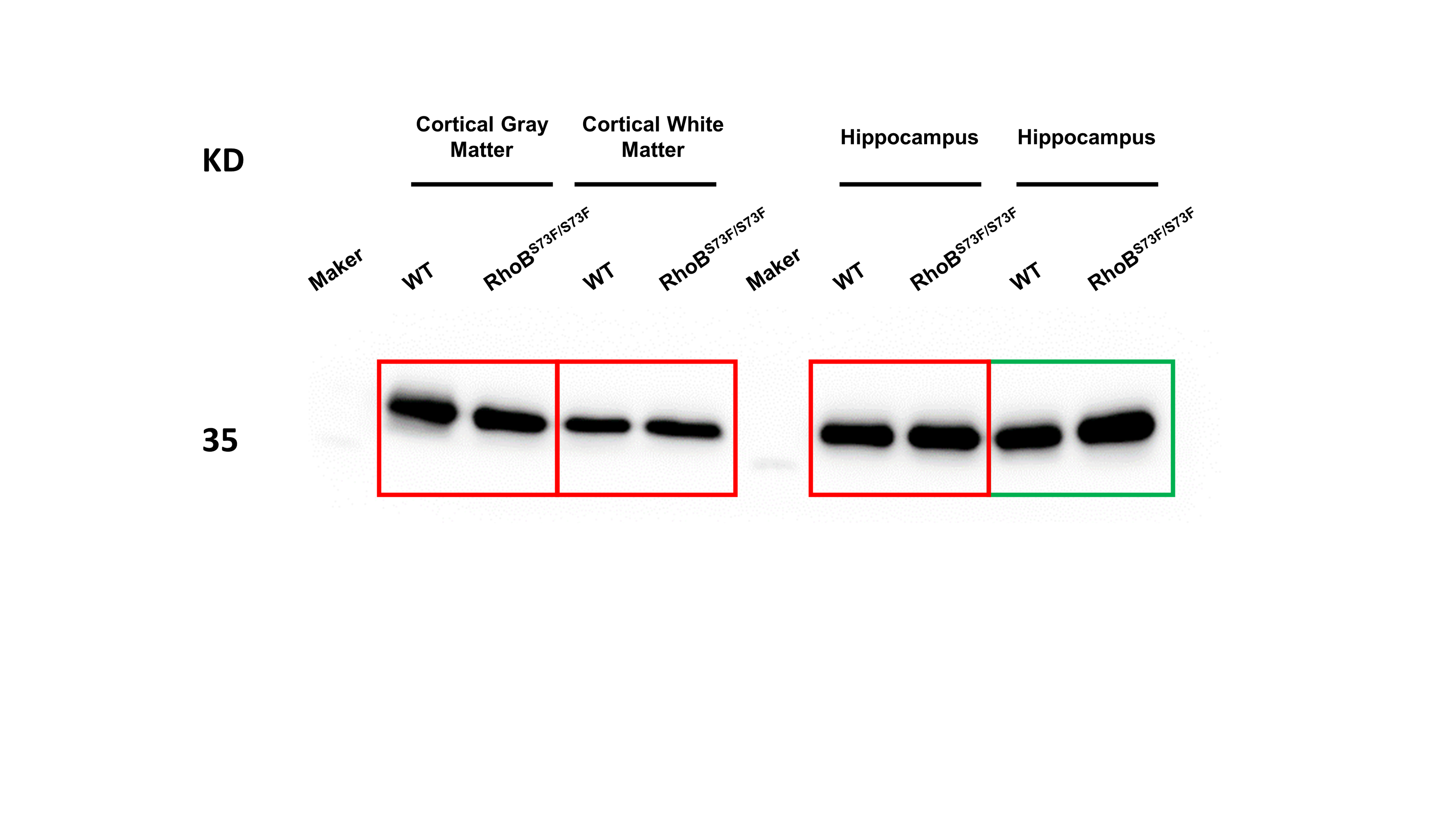

Supplement: Supplementary file 14 — Source data Fig. 4 [file 44321_2024_113_MOESM14_ESM.zip › Figure 4/4E/western Gapdh.tif]

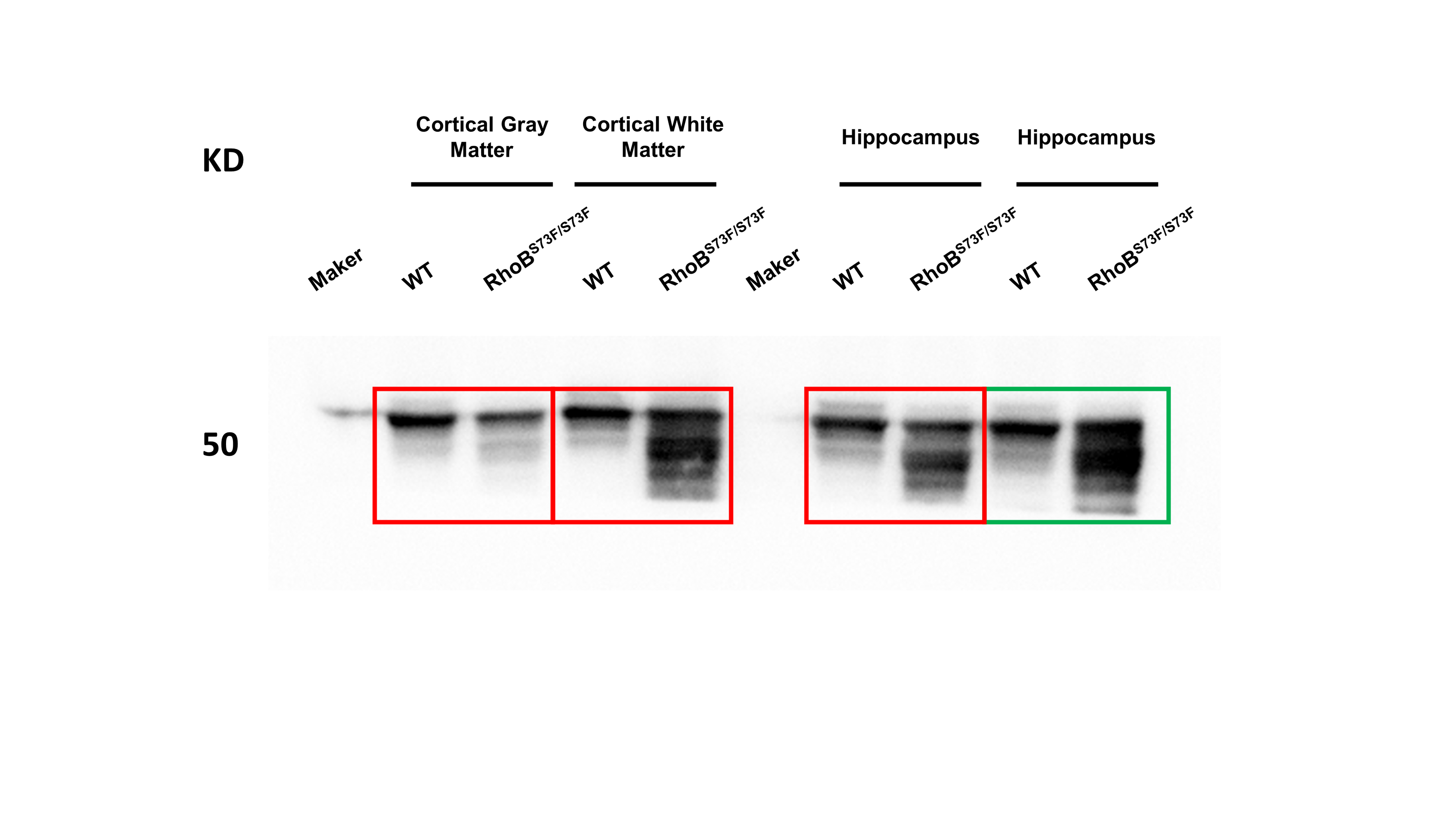

Supplement: Supplementary file 14 — Source data Fig. 4 [file 44321_2024_113_MOESM14_ESM.zip › Figure 4/4E/western GFAP.tif]

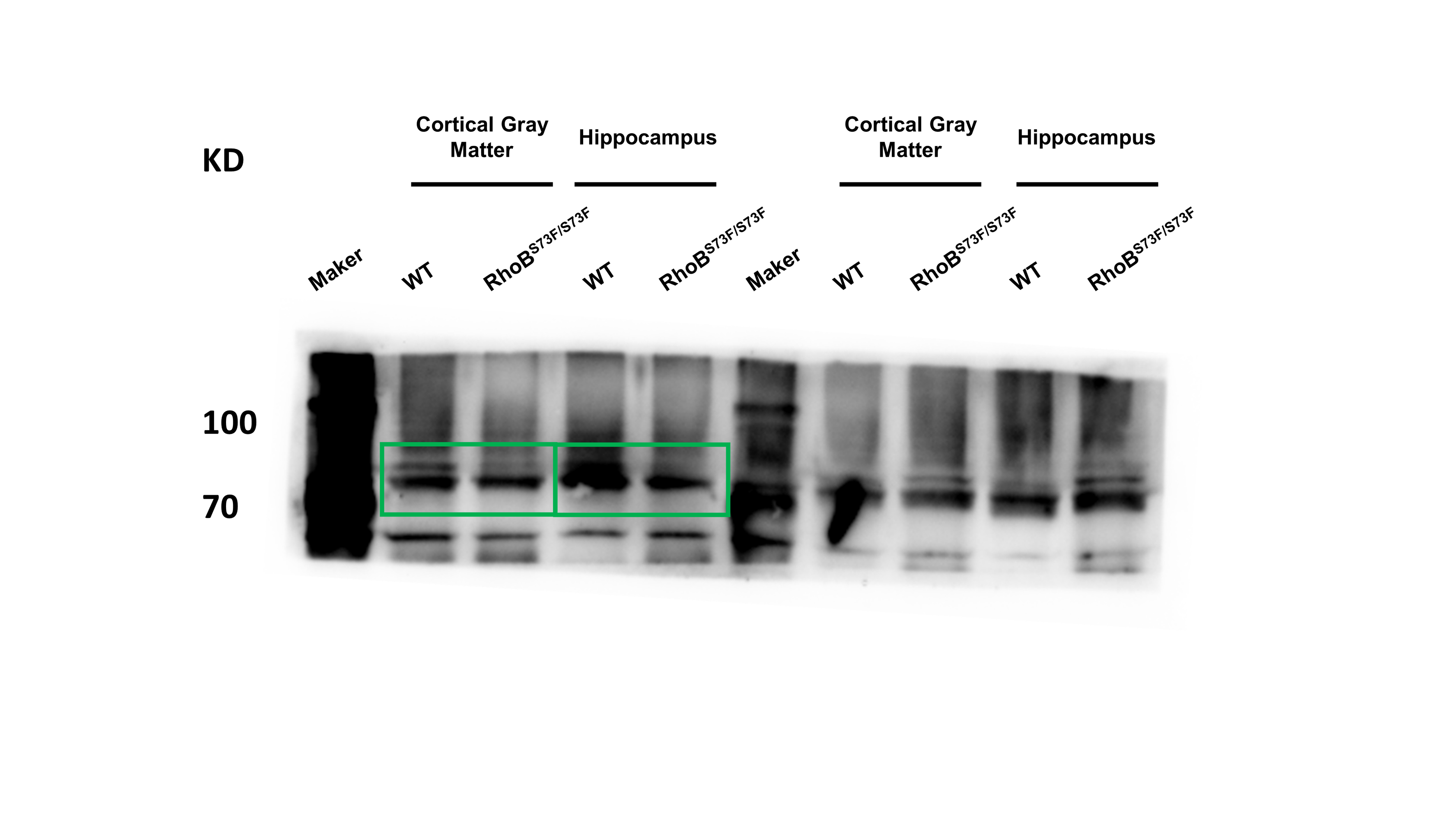

Supplement: Supplementary file 14 — Source data Fig. 4 [file 44321_2024_113_MOESM14_ESM.zip › Figure 4/4F/replicate/western Calpain1 in Cortical Gray Matter&Hippocampus replicate.tif]

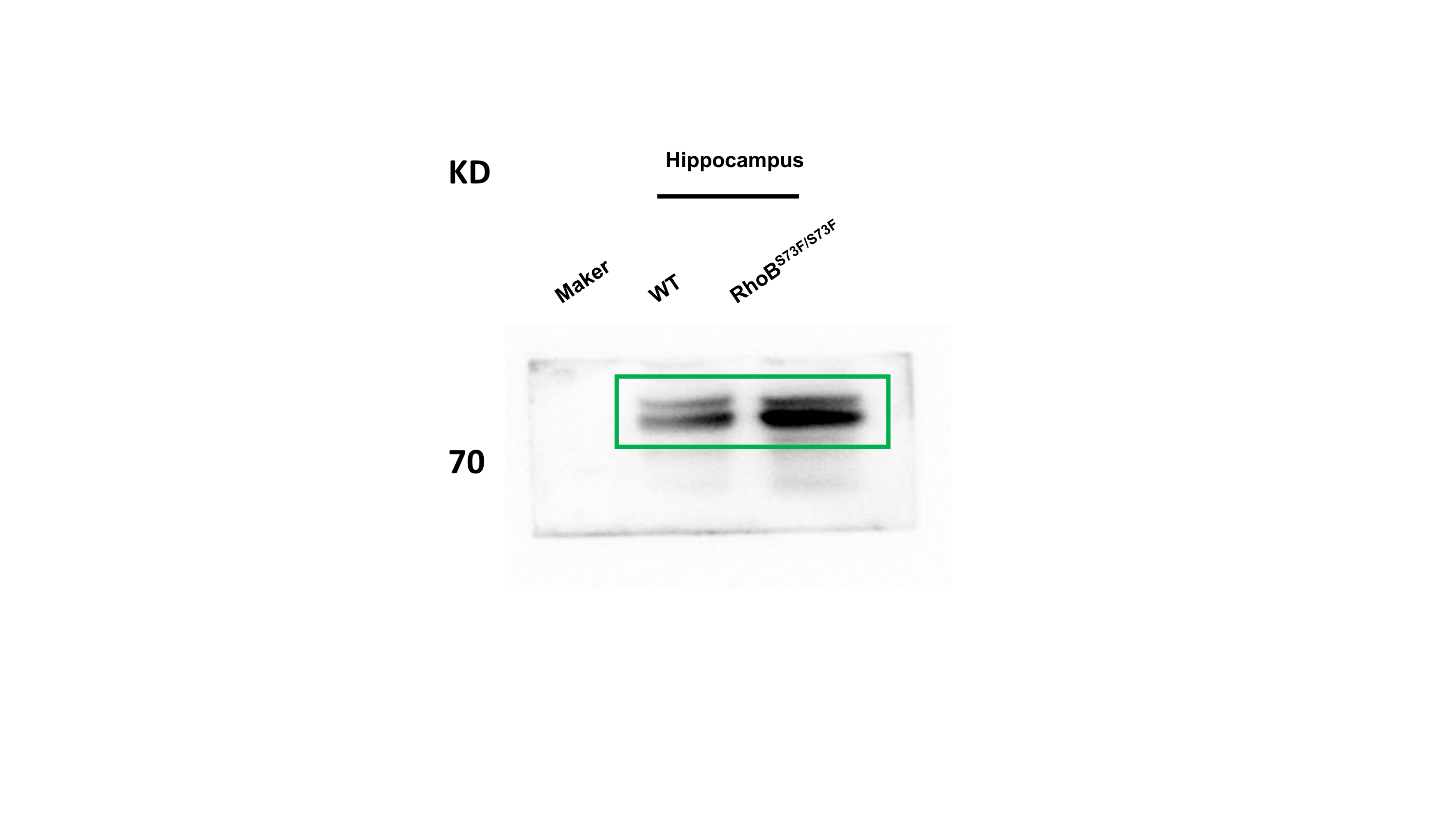

Supplement: Supplementary file 14 — Source data Fig. 4 [file 44321_2024_113_MOESM14_ESM.zip › Figure 4/4F/replicate/western Calpain1 in Hippocampus replicate.tif]

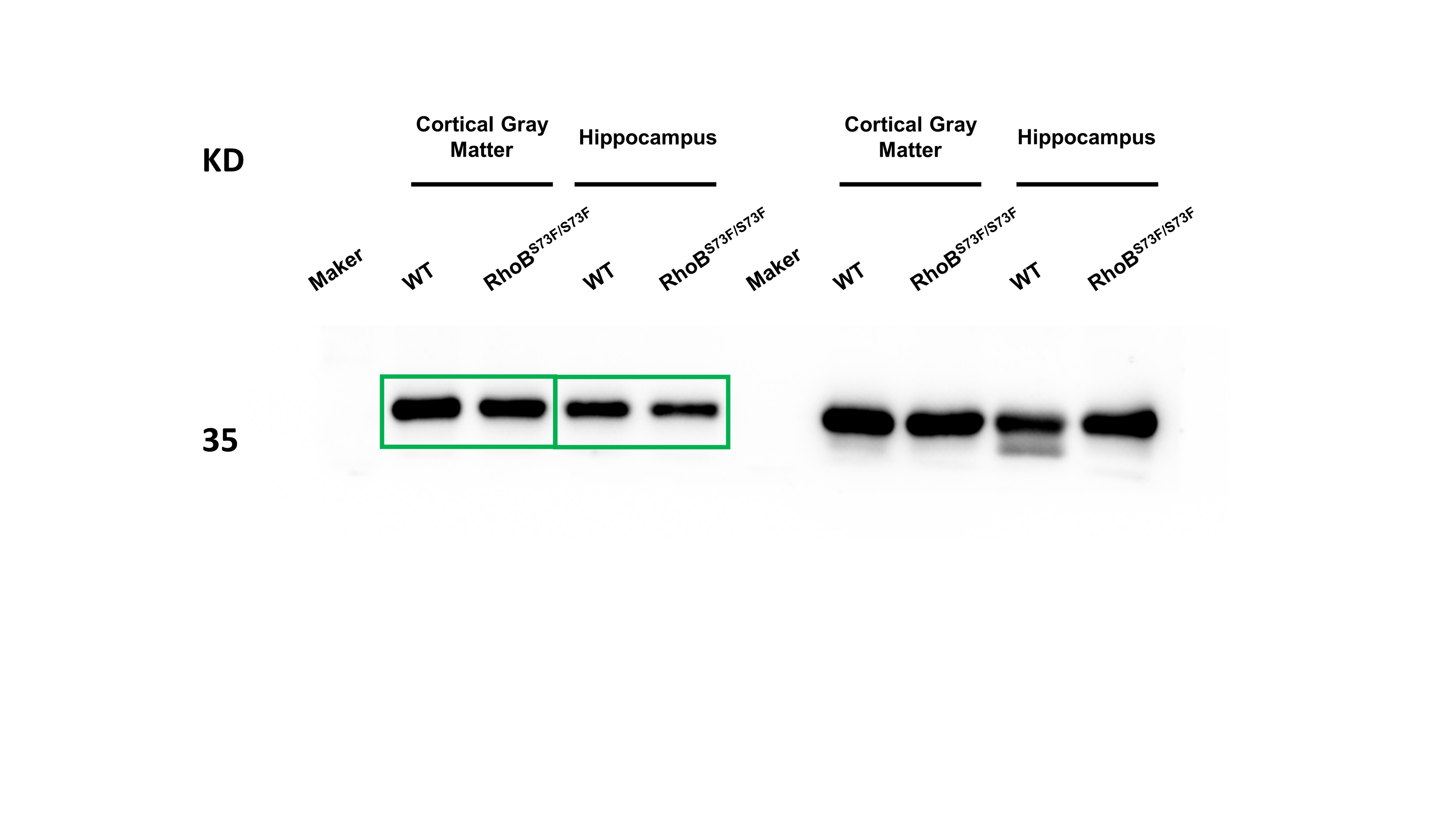

Supplement: Supplementary file 14 — Source data Fig. 4 [file 44321_2024_113_MOESM14_ESM.zip › Figure 4/4F/replicate/western Gapdh in Cortical Gray Matter&Hippocampus replicate.tif]

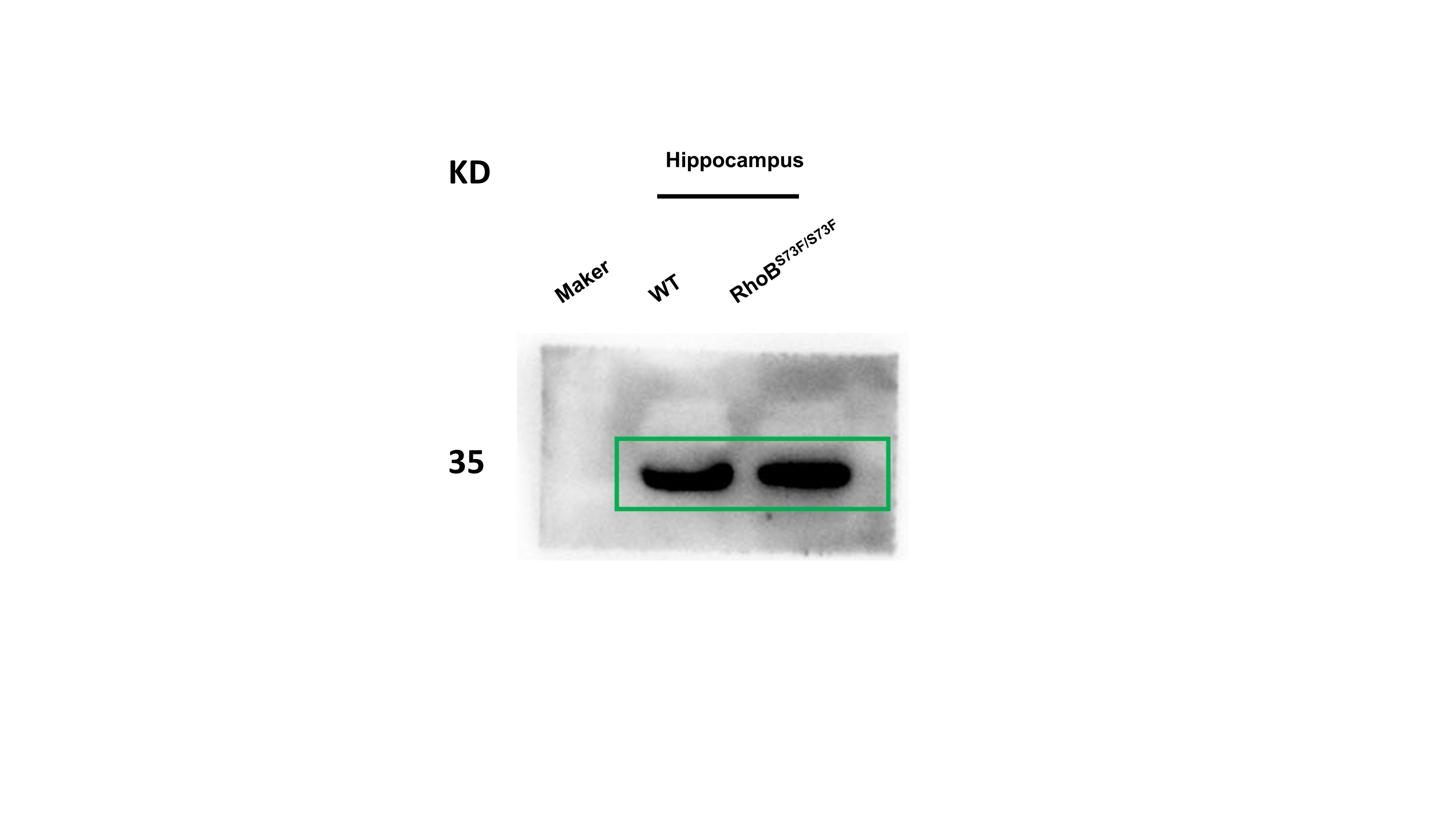

Supplement: Supplementary file 14 — Source data Fig. 4 [file 44321_2024_113_MOESM14_ESM.zip › Figure 4/4F/replicate/western Gapdh in Cortical Hippocampus replicate.tif]

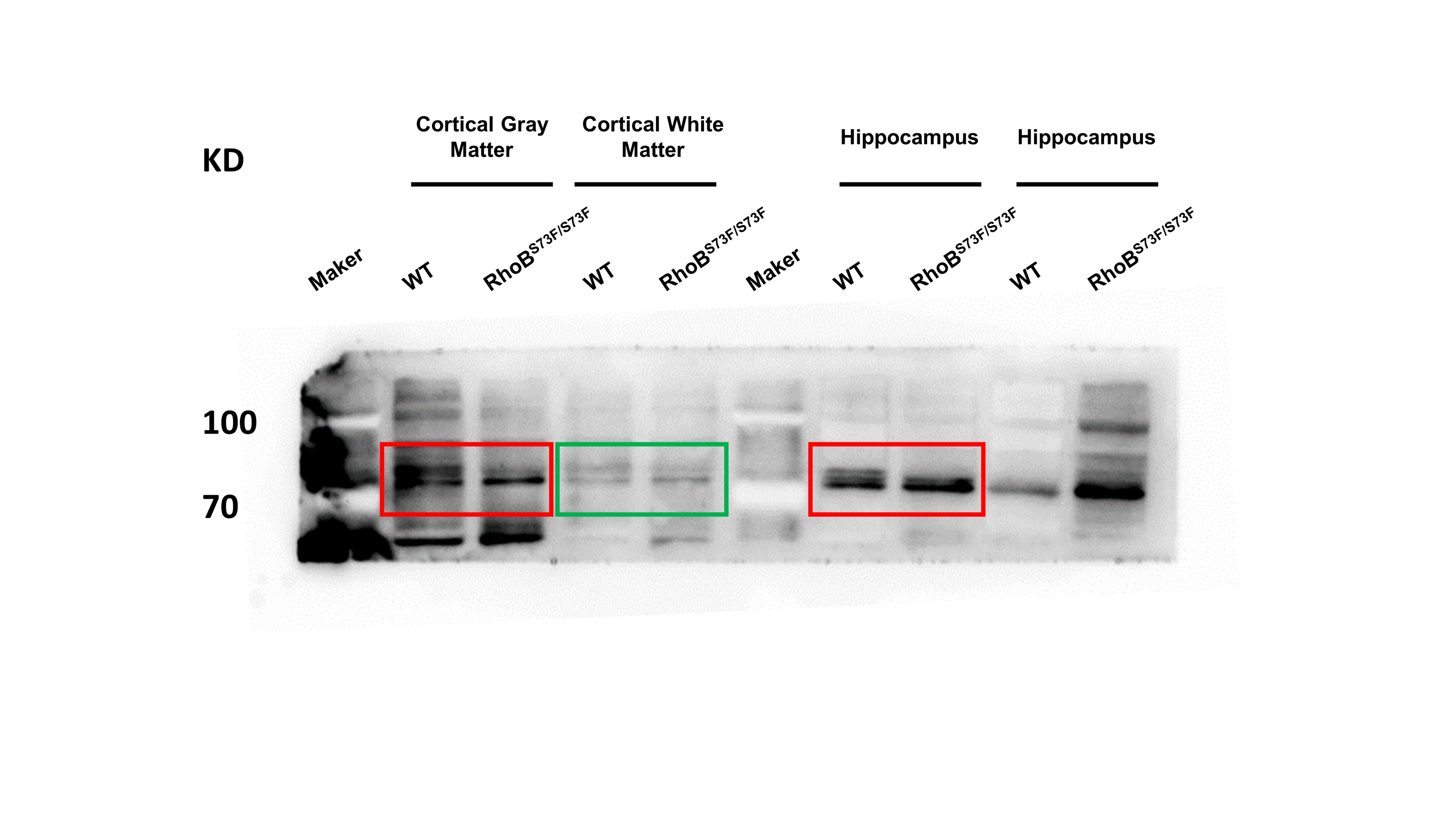

Supplement: Supplementary file 14 — Source data Fig. 4 [file 44321_2024_113_MOESM14_ESM.zip › Figure 4/4F/western Calpain1 in Cortical Gray Matter&Hippocampus.tif]

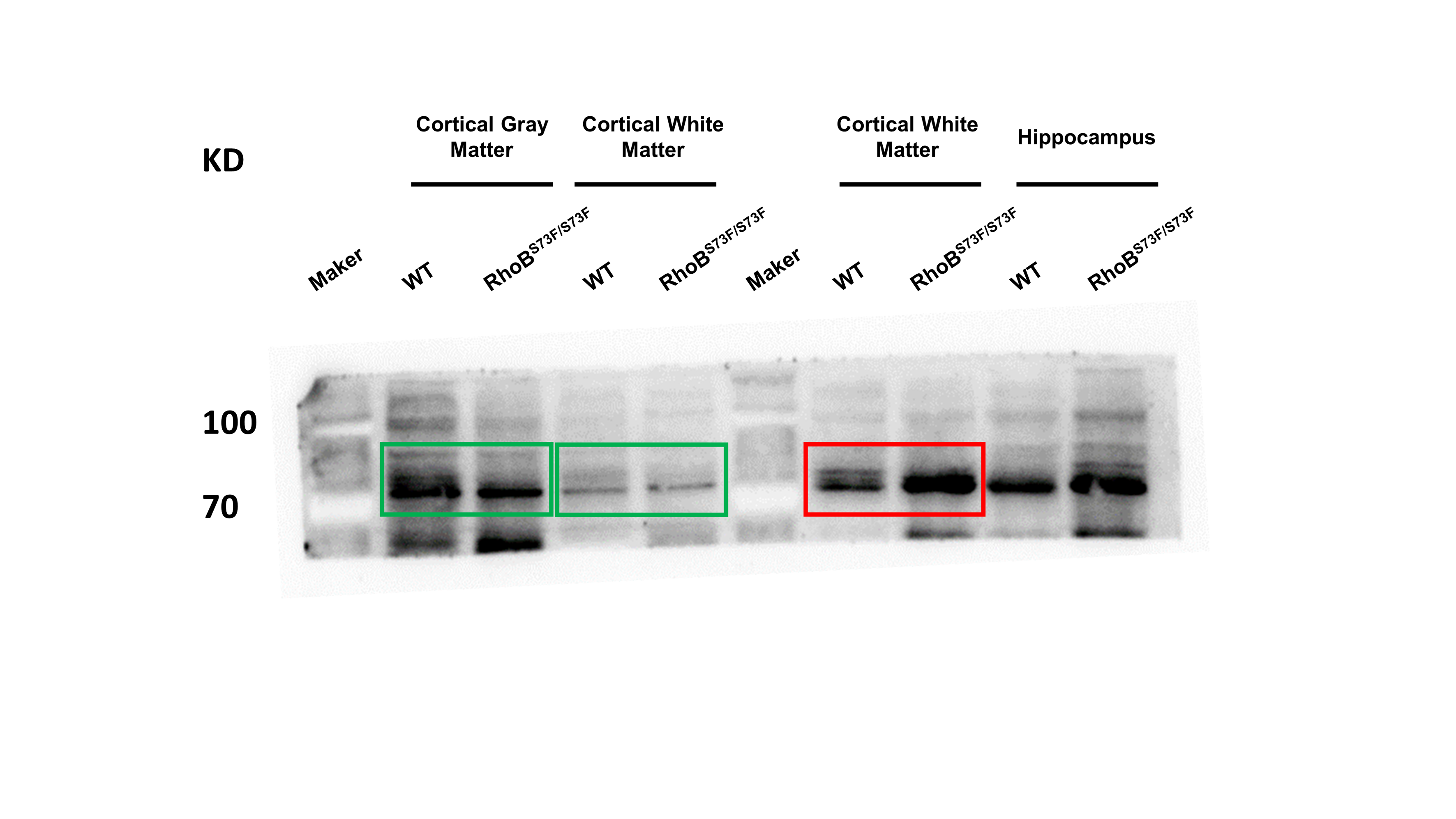

Supplement: Supplementary file 14 — Source data Fig. 4 [file 44321_2024_113_MOESM14_ESM.zip › Figure 4/4F/western Calpain1 in Cortical White Matter.tif]

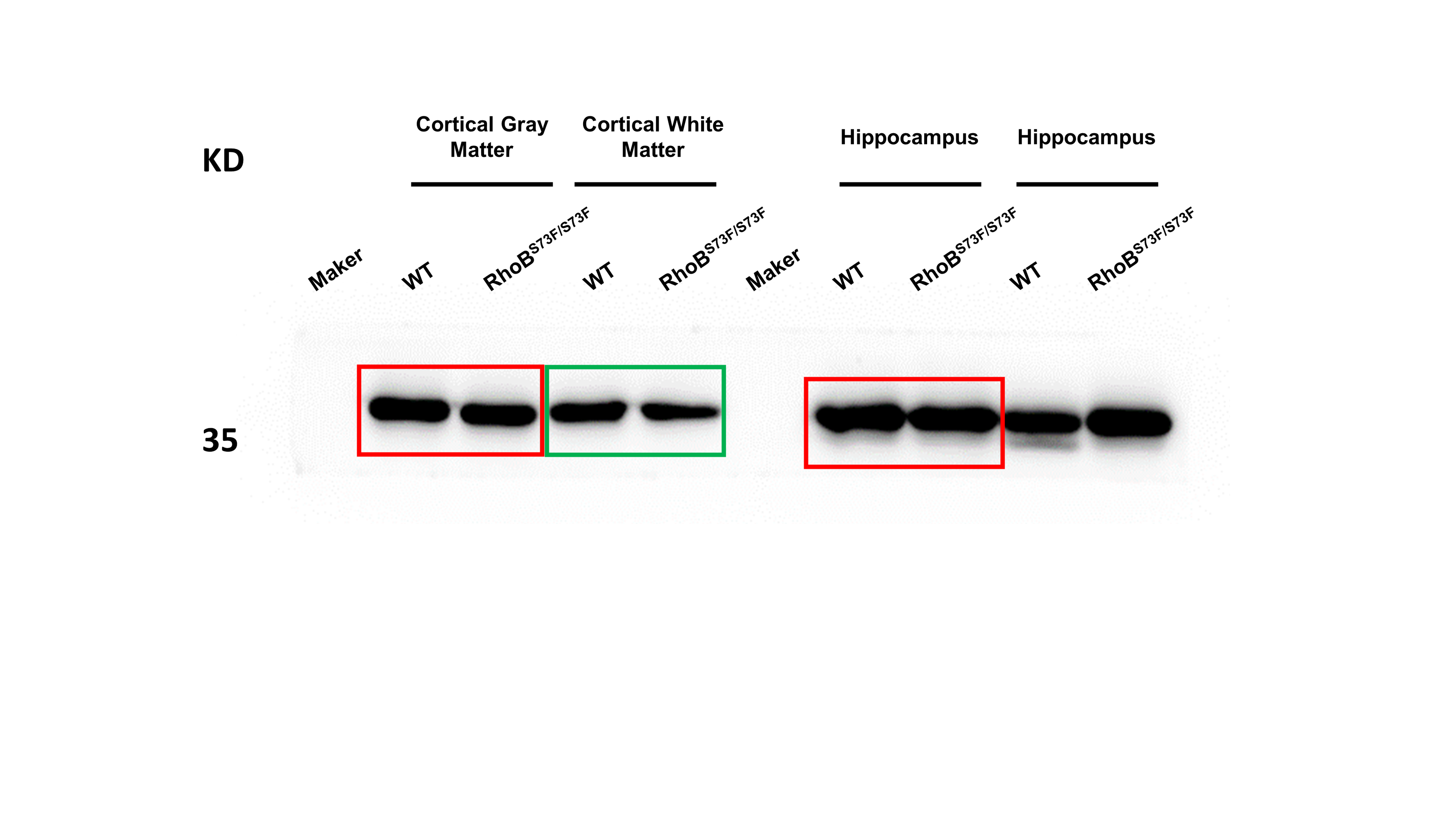

Supplement: Supplementary file 14 — Source data Fig. 4 [file 44321_2024_113_MOESM14_ESM.zip › Figure 4/4F/western Gapdh in Cortical Gray Matter&Hippocampus.tif]

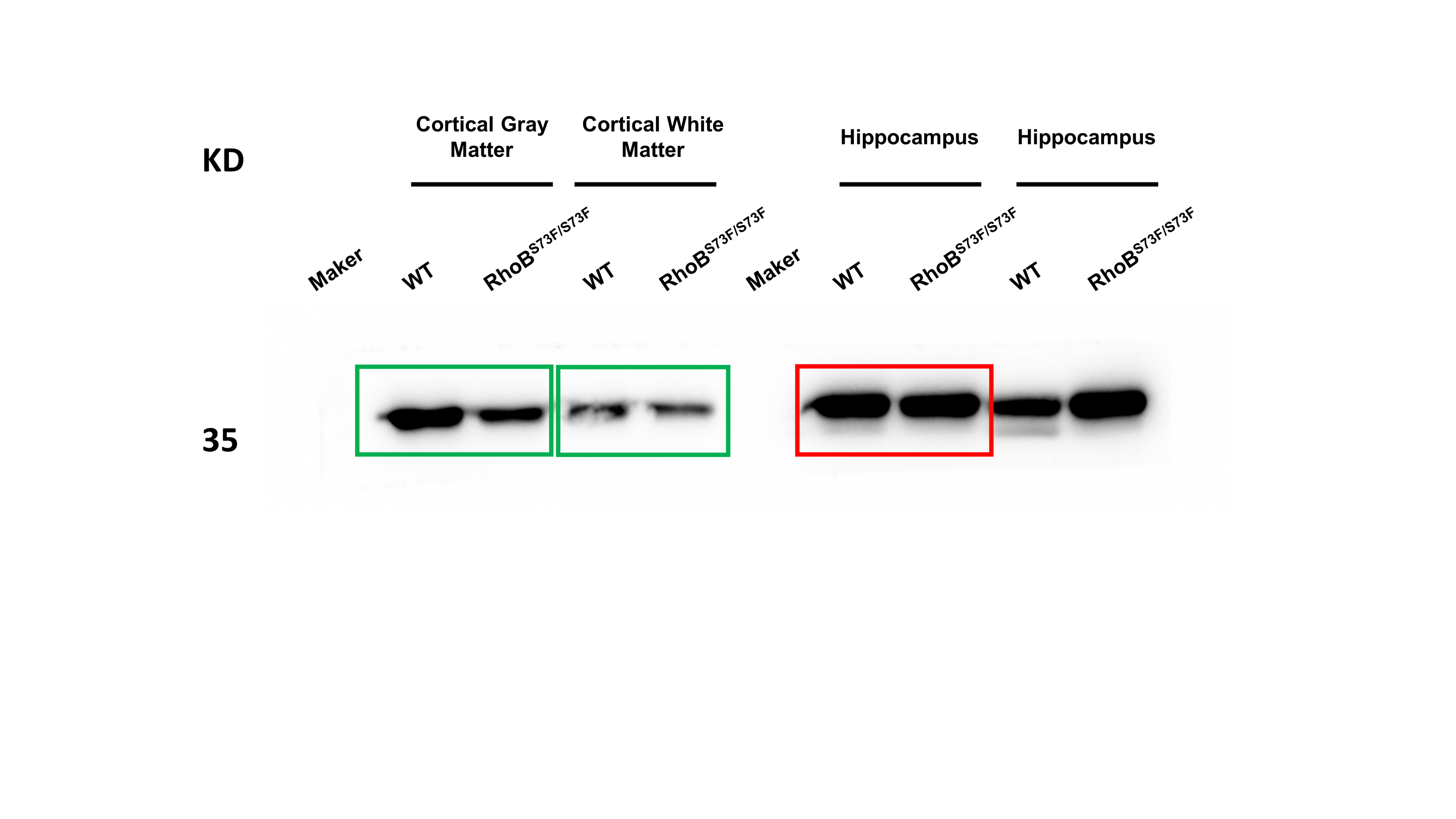

Supplement: Supplementary file 14 — Source data Fig. 4 [file 44321_2024_113_MOESM14_ESM.zip › Figure 4/4F/western Gapdh in Cortical White Matter.tif]

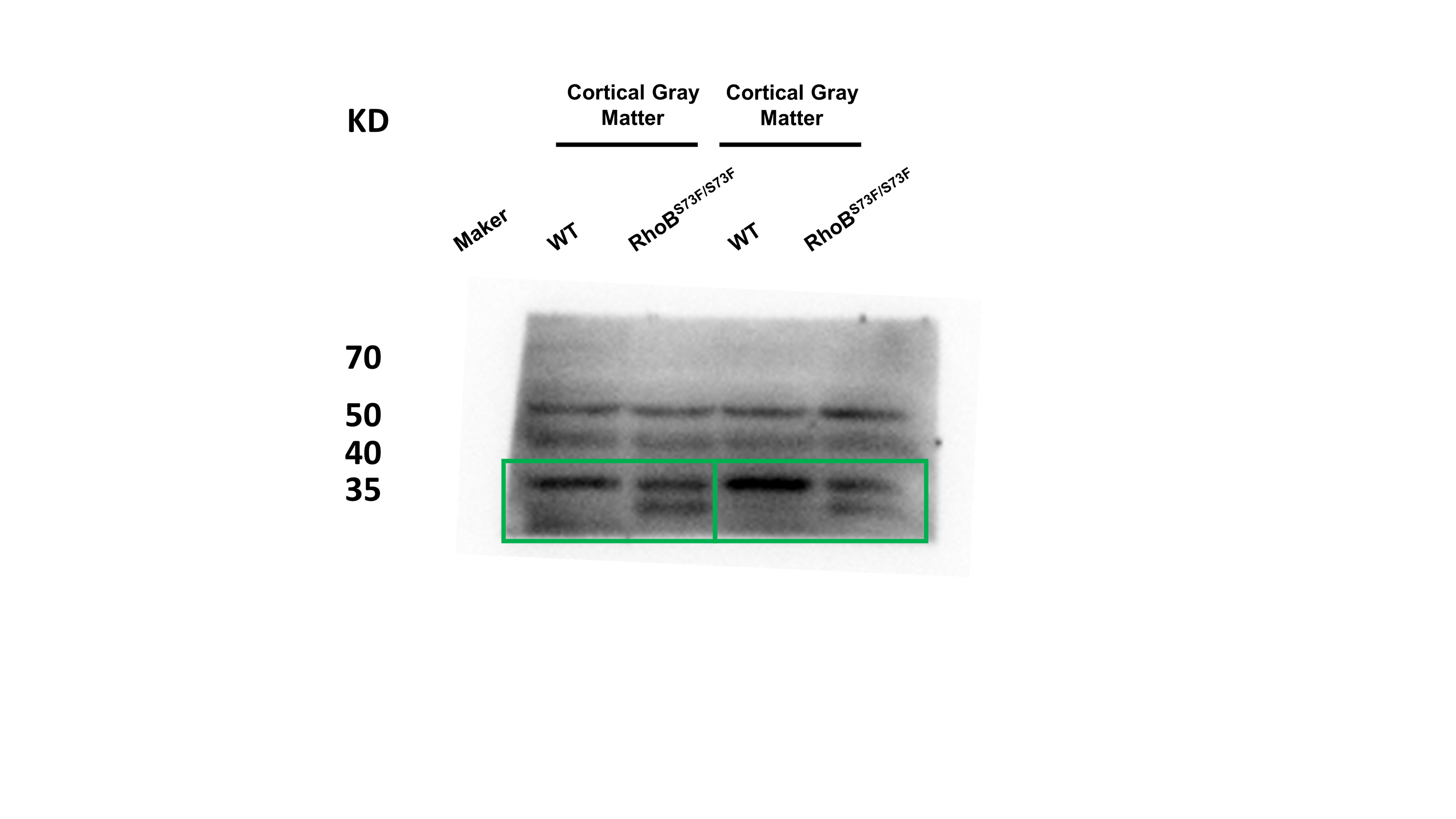

Supplement: Supplementary file 14 — Source data Fig. 4 [file 44321_2024_113_MOESM14_ESM.zip › Figure 4/4G/replicate/western Caspase3 in Cortical Gray Matter replicate.tif]

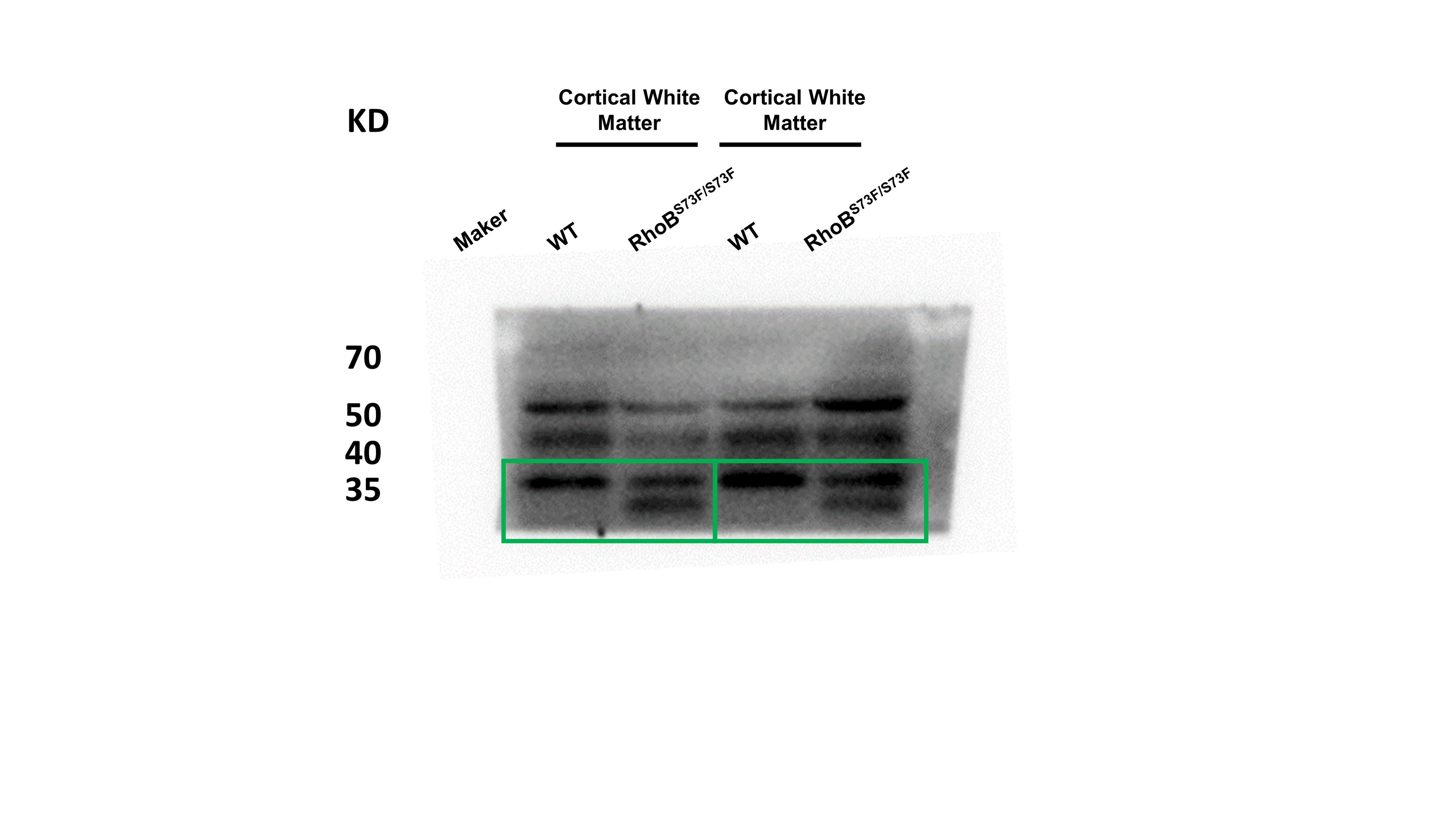

Supplement: Supplementary file 14 — Source data Fig. 4 [file 44321_2024_113_MOESM14_ESM.zip › Figure 4/4G/replicate/western Caspase3 in Cortical White Matter replicate.tif]

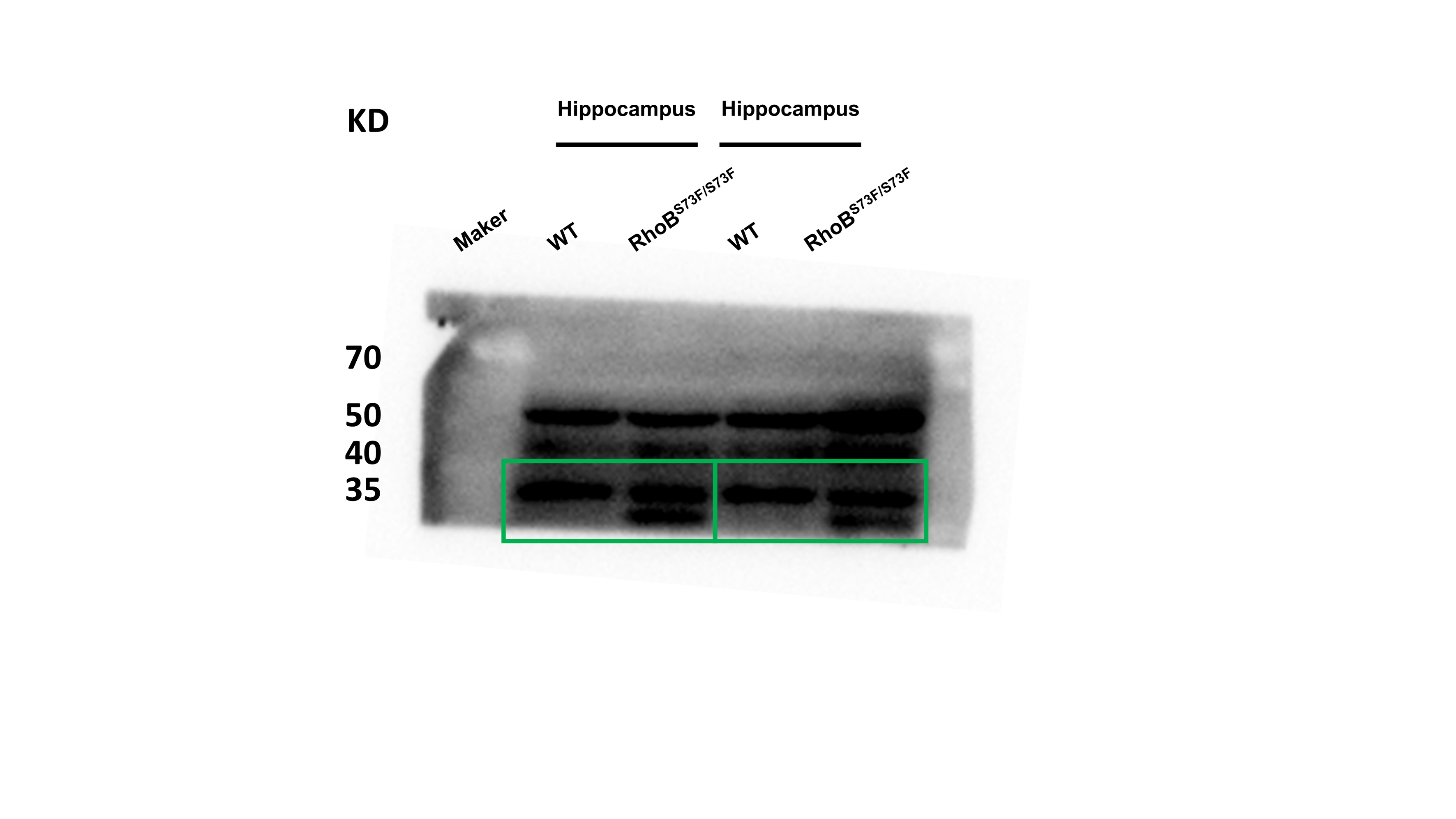

Supplement: Supplementary file 14 — Source data Fig. 4 [file 44321_2024_113_MOESM14_ESM.zip › Figure 4/4G/replicate/western Caspase3 in Hippocampus replicate.tif]

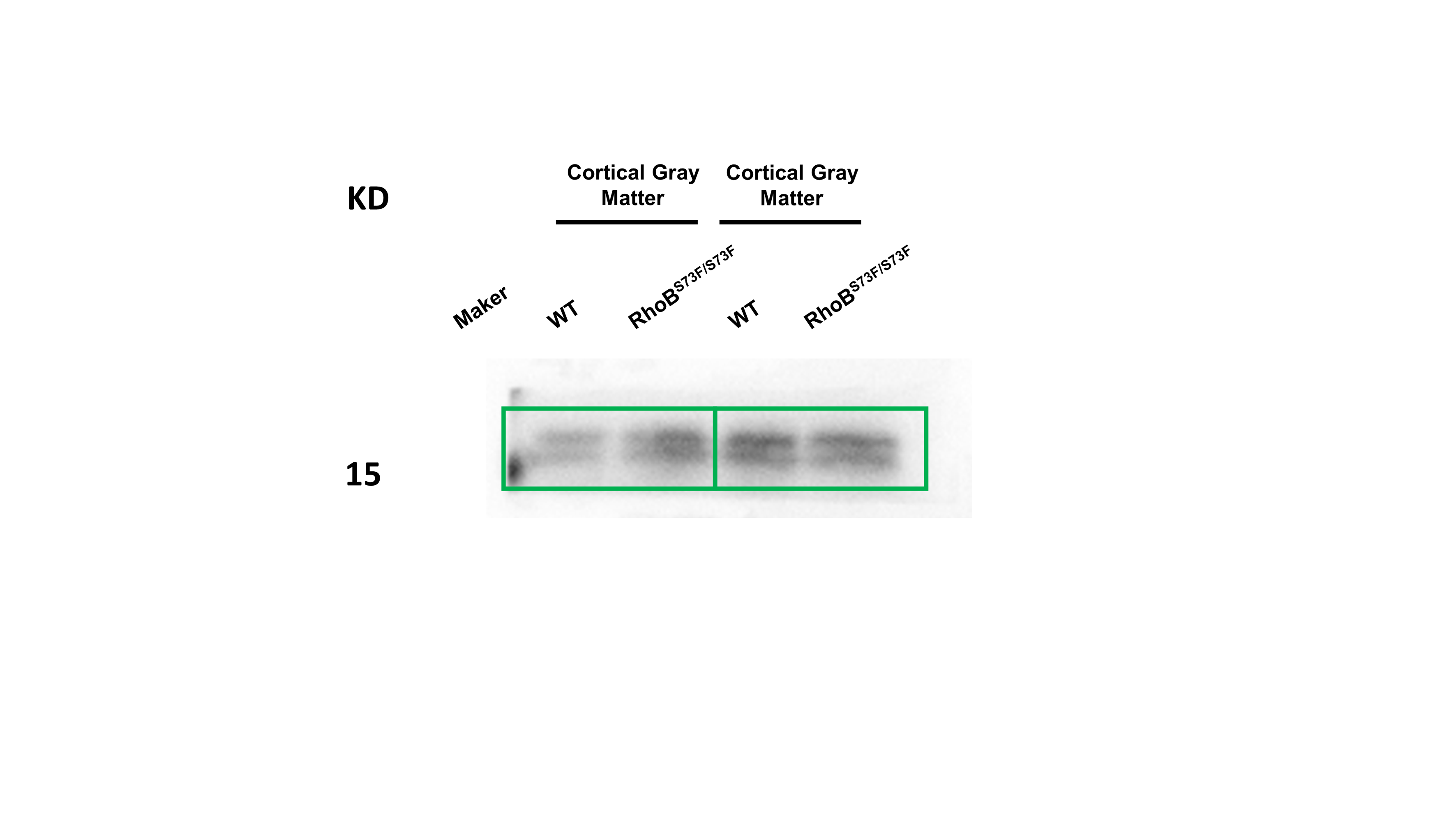

Supplement: Supplementary file 14 — Source data Fig. 4 [file 44321_2024_113_MOESM14_ESM.zip › Figure 4/4G/replicate/western Cleaved Caspase3 in Cortical Gray Matter replicate.tif]

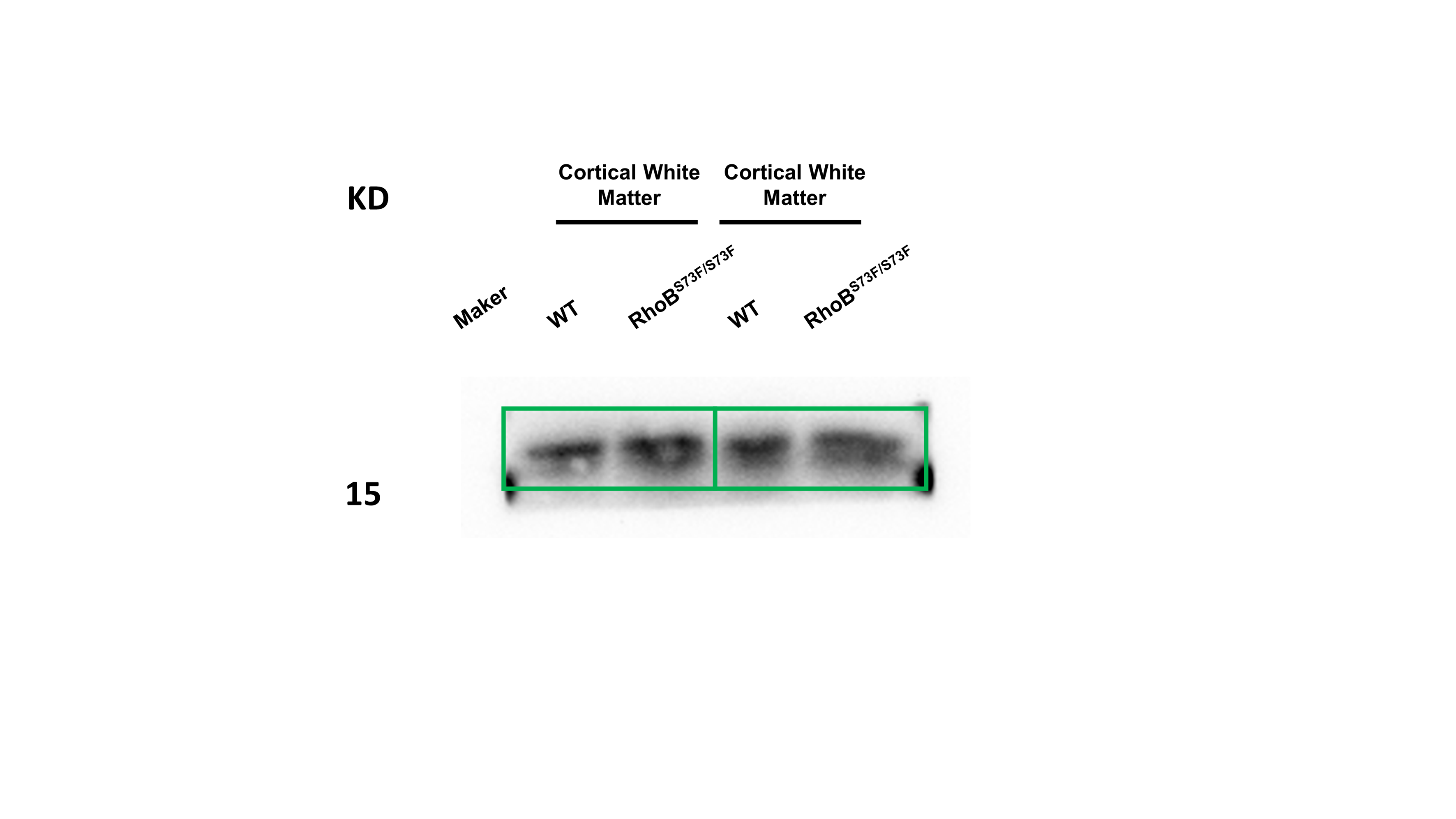

Supplement: Supplementary file 14 — Source data Fig. 4 [file 44321_2024_113_MOESM14_ESM.zip › Figure 4/4G/replicate/western Cleaved Caspase3 in Cortical White Matter replicate.tif]

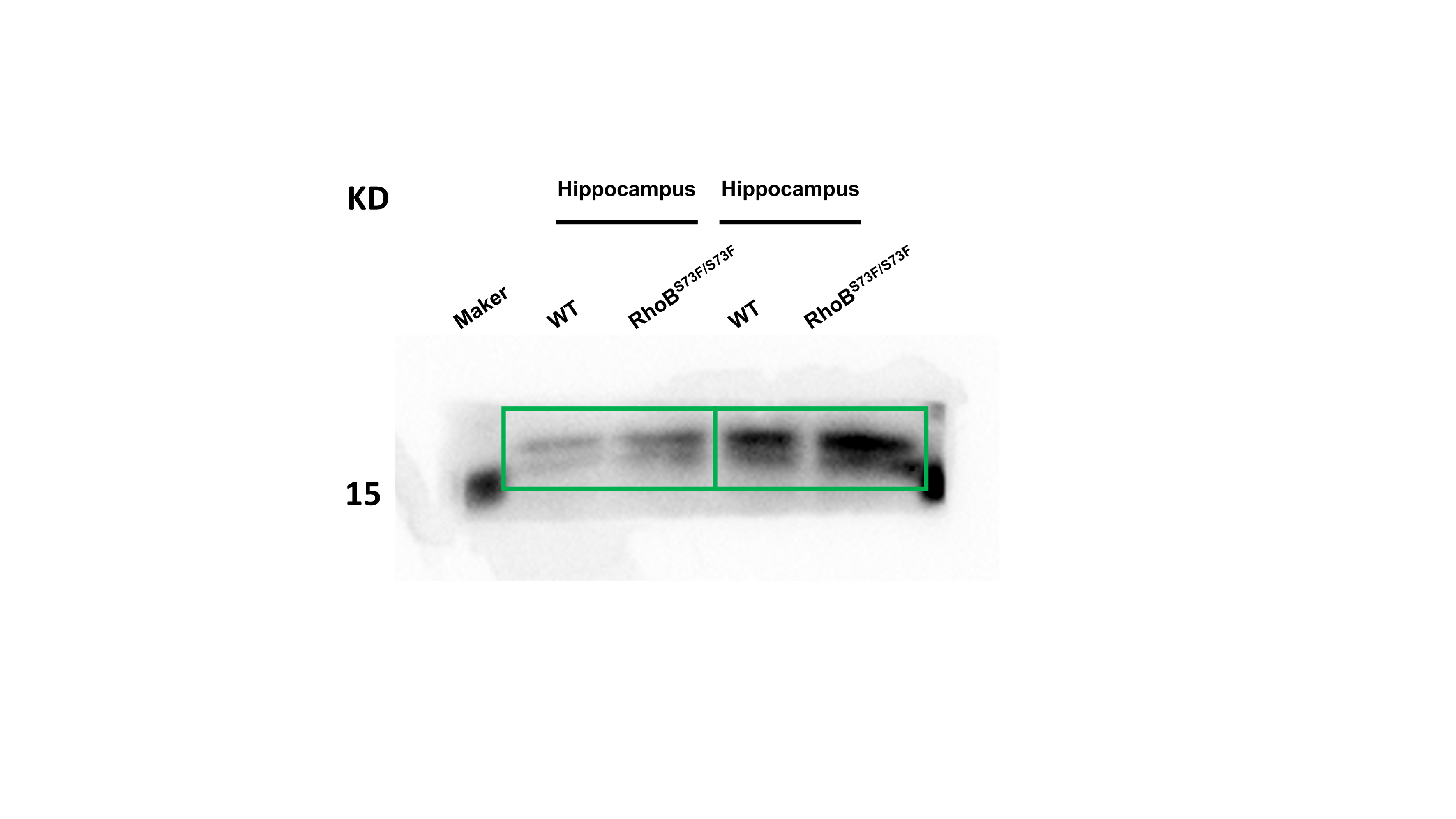

Supplement: Supplementary file 14 — Source data Fig. 4 [file 44321_2024_113_MOESM14_ESM.zip › Figure 4/4G/replicate/western Cleaved Caspase3 in Hippocampus replicate.tif]

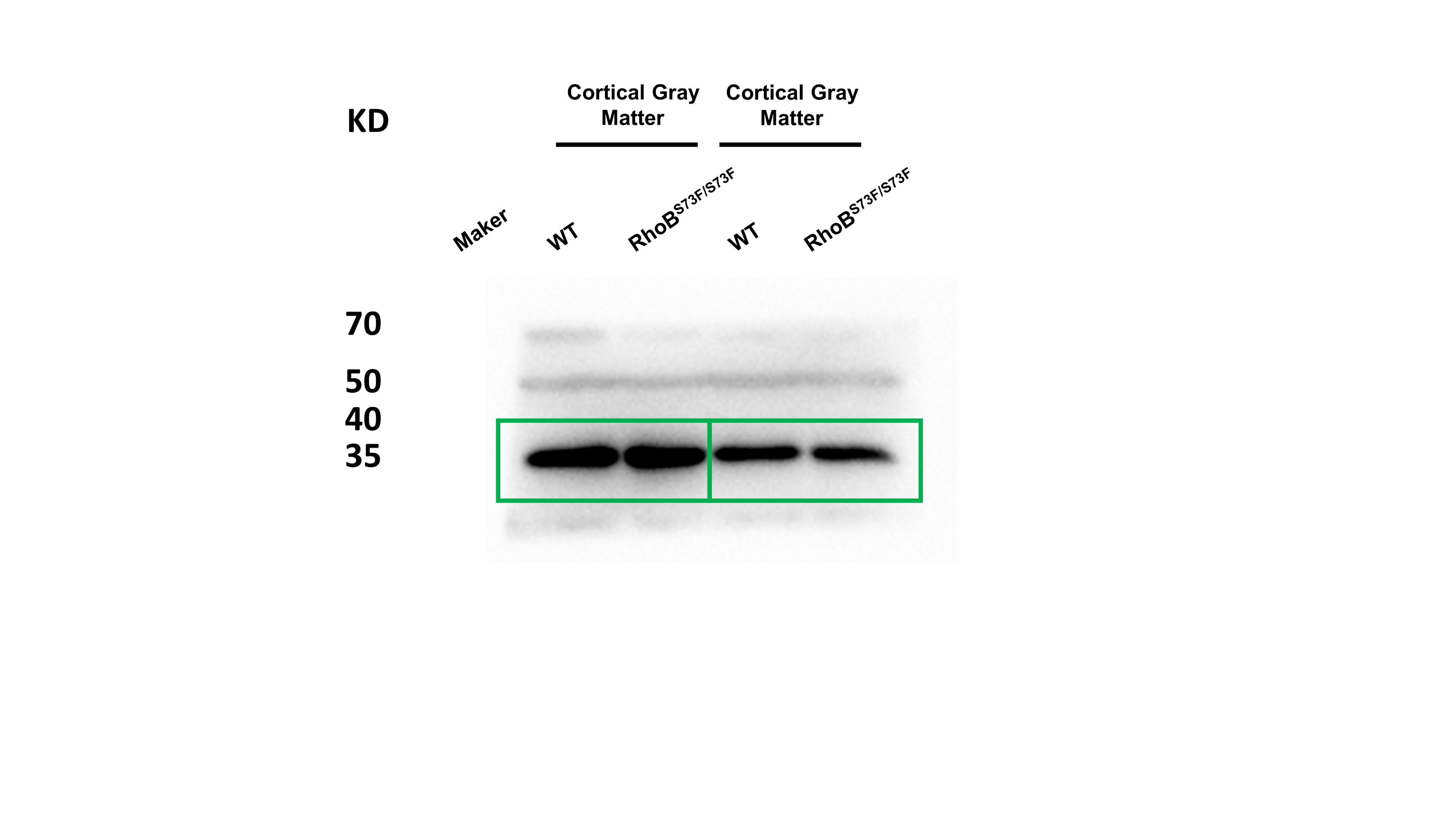

Supplement: Supplementary file 14 — Source data Fig. 4 [file 44321_2024_113_MOESM14_ESM.zip › Figure 4/4G/replicate/western Gapdh in Cortical Gray Matter replicate.tif]

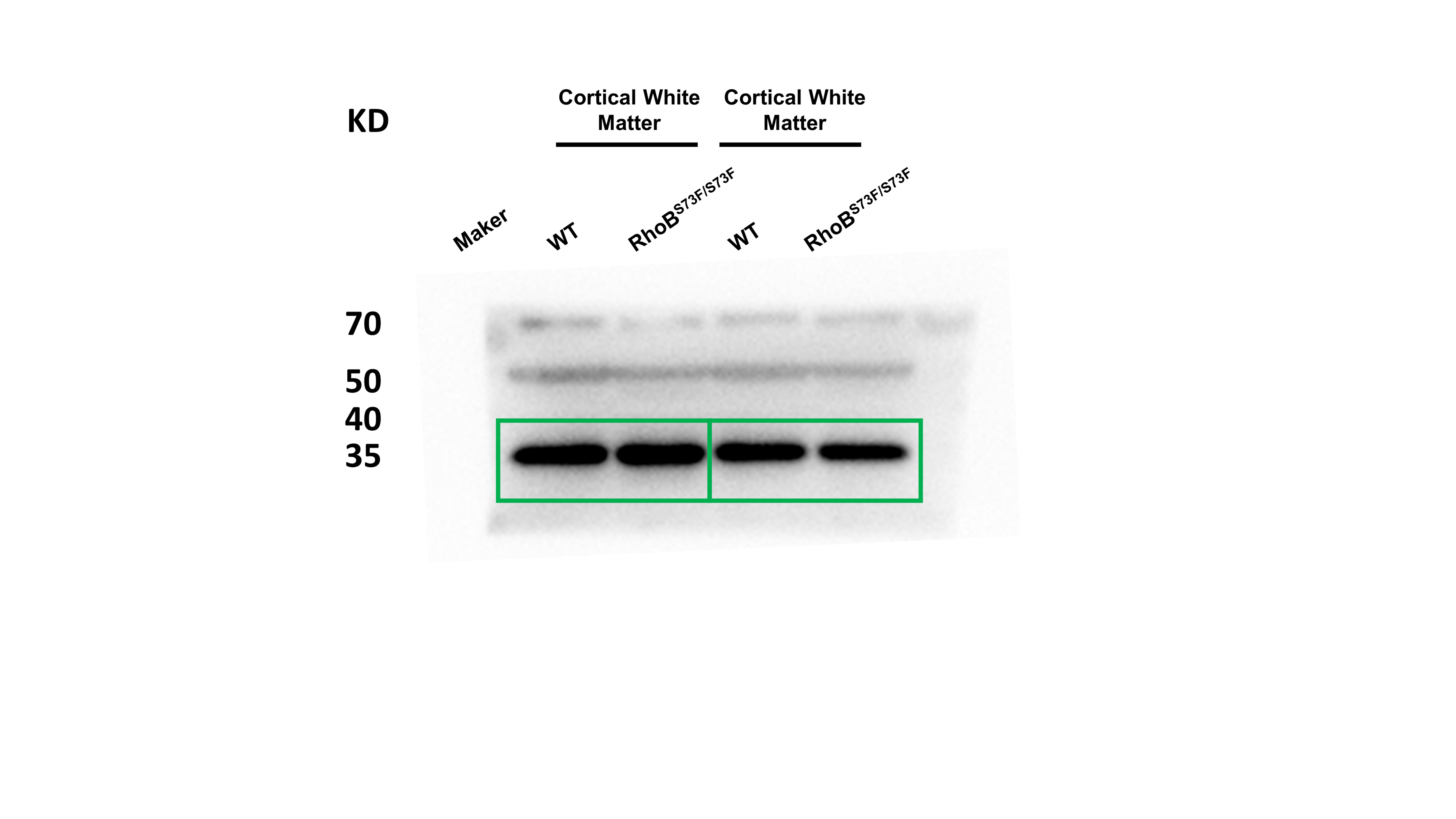

Supplement: Supplementary file 14 — Source data Fig. 4 [file 44321_2024_113_MOESM14_ESM.zip › Figure 4/4G/replicate/western Gapdh in Cortical White Matter replicate.tif]

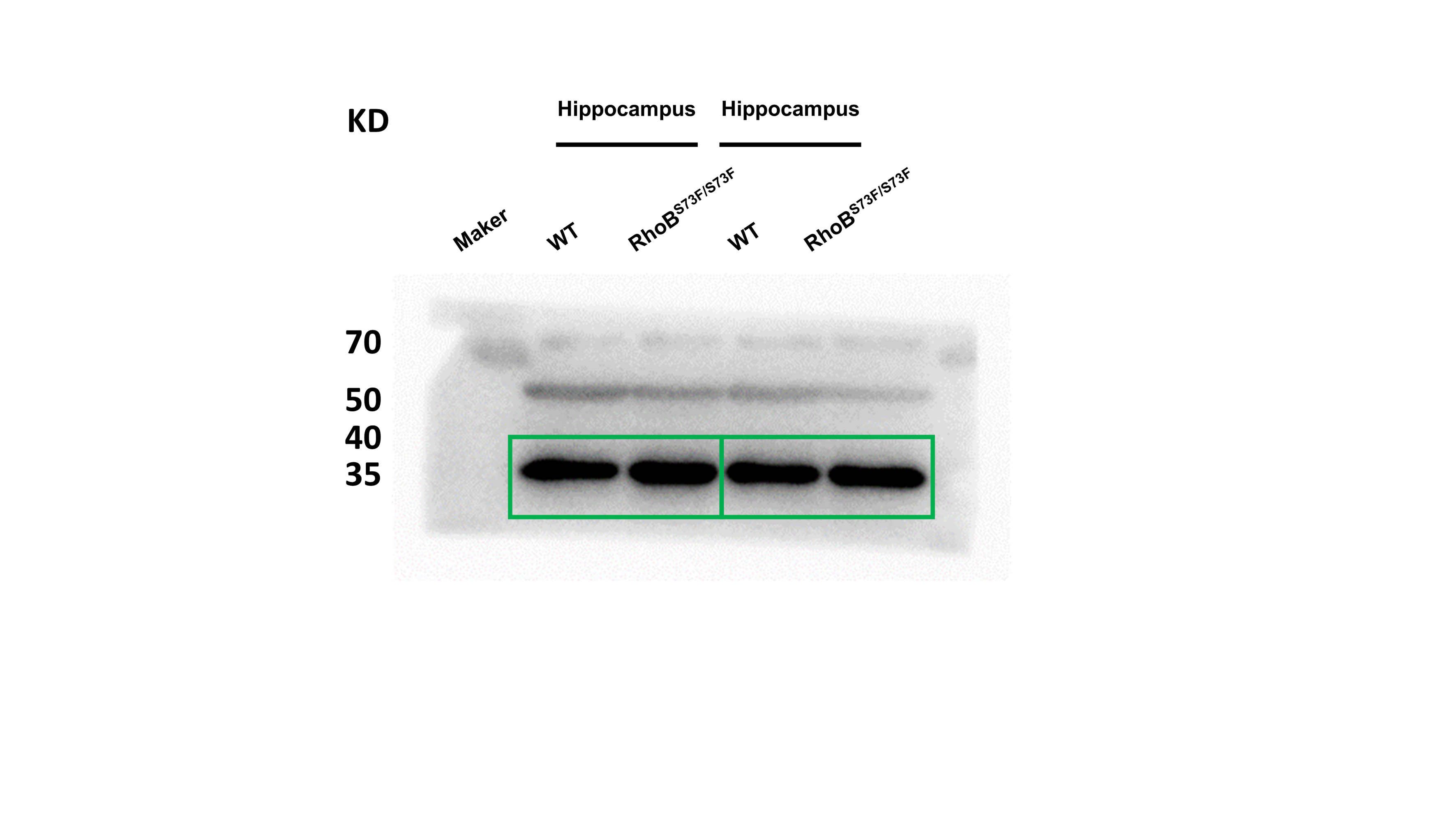

Supplement: Supplementary file 14 — Source data Fig. 4 [file 44321_2024_113_MOESM14_ESM.zip › Figure 4/4G/replicate/western Gapdh in Hippocampus replicate.tif]

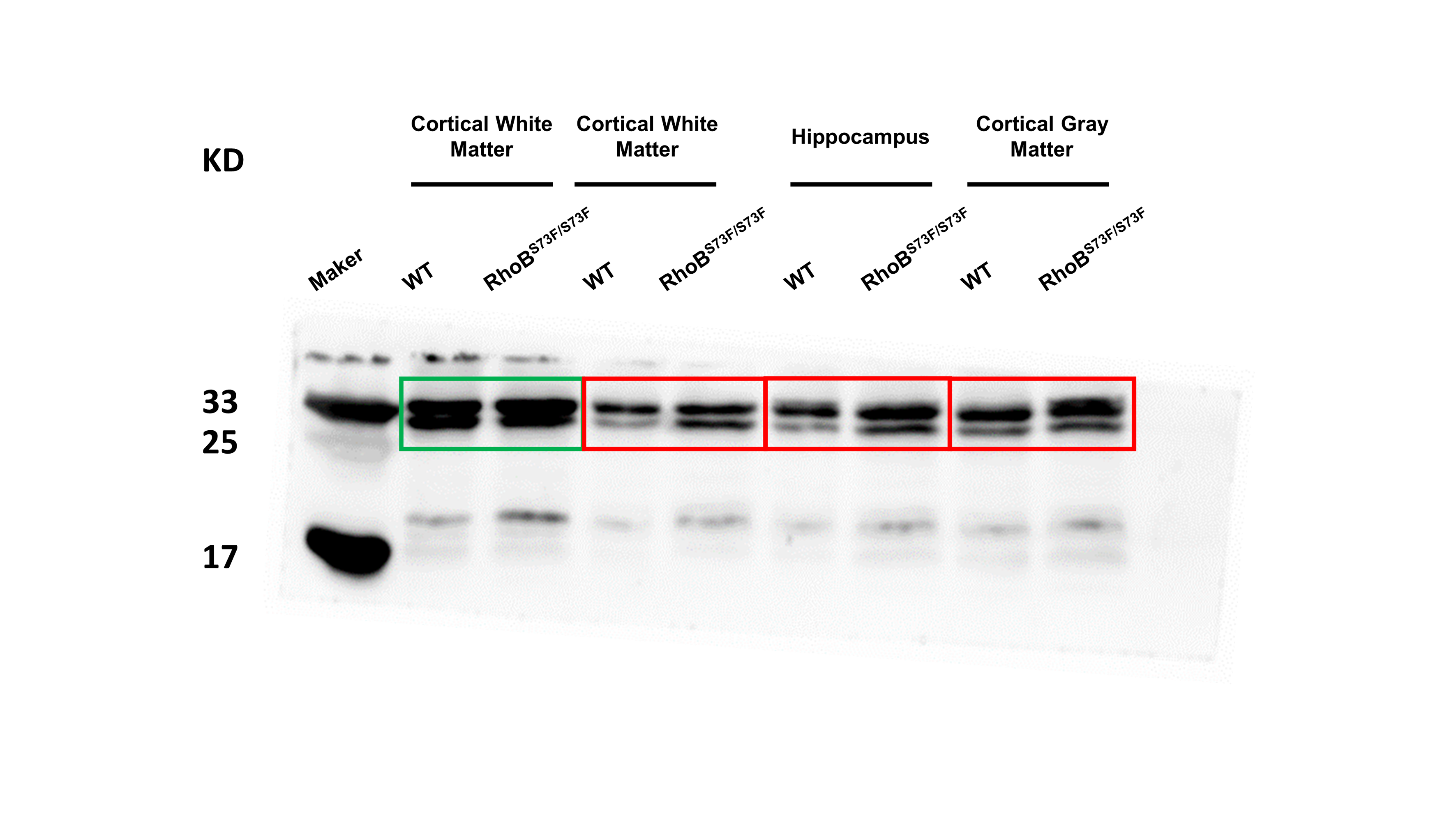

Supplement: Supplementary file 14 — Source data Fig. 4 [file 44321_2024_113_MOESM14_ESM.zip › Figure 4/4G/western Caspase3.tif]

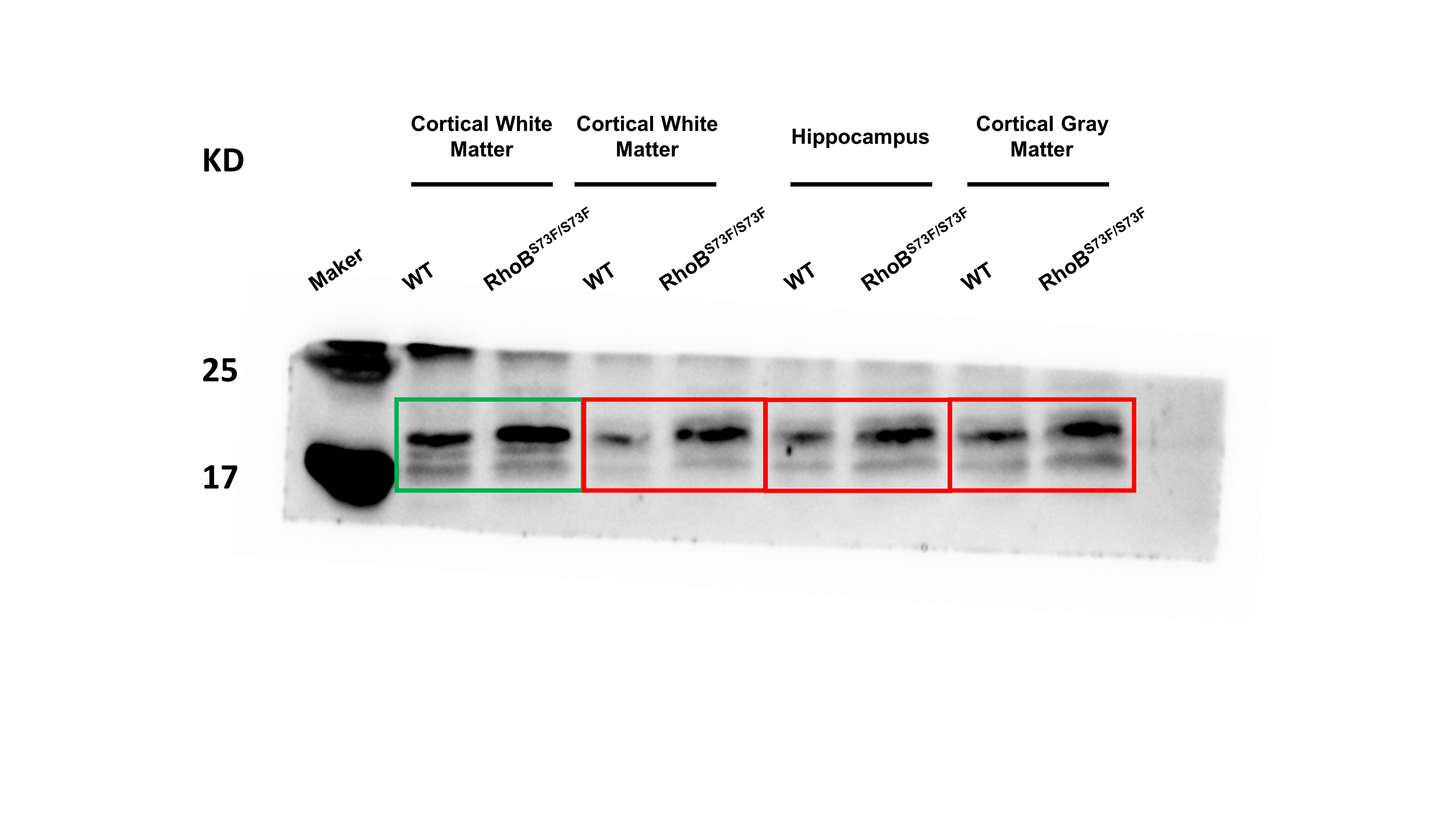

Supplement: Supplementary file 14 — Source data Fig. 4 [file 44321_2024_113_MOESM14_ESM.zip › Figure 4/4G/western Cleaved Caspase3.tif]

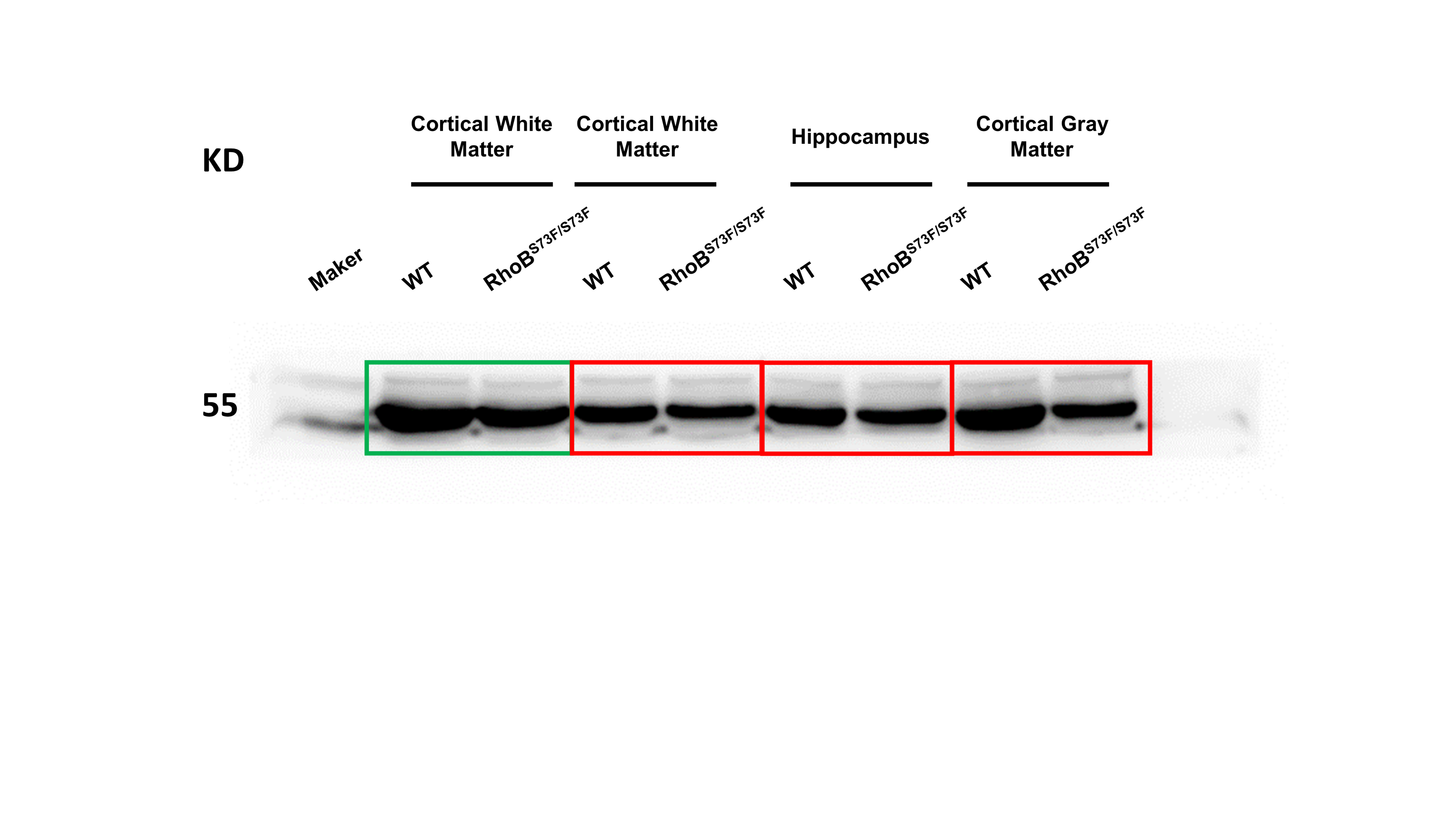

Supplement: Supplementary file 14 — Source data Fig. 4 [file 44321_2024_113_MOESM14_ESM.zip › Figure 4/4G/western Gapdh.tif]

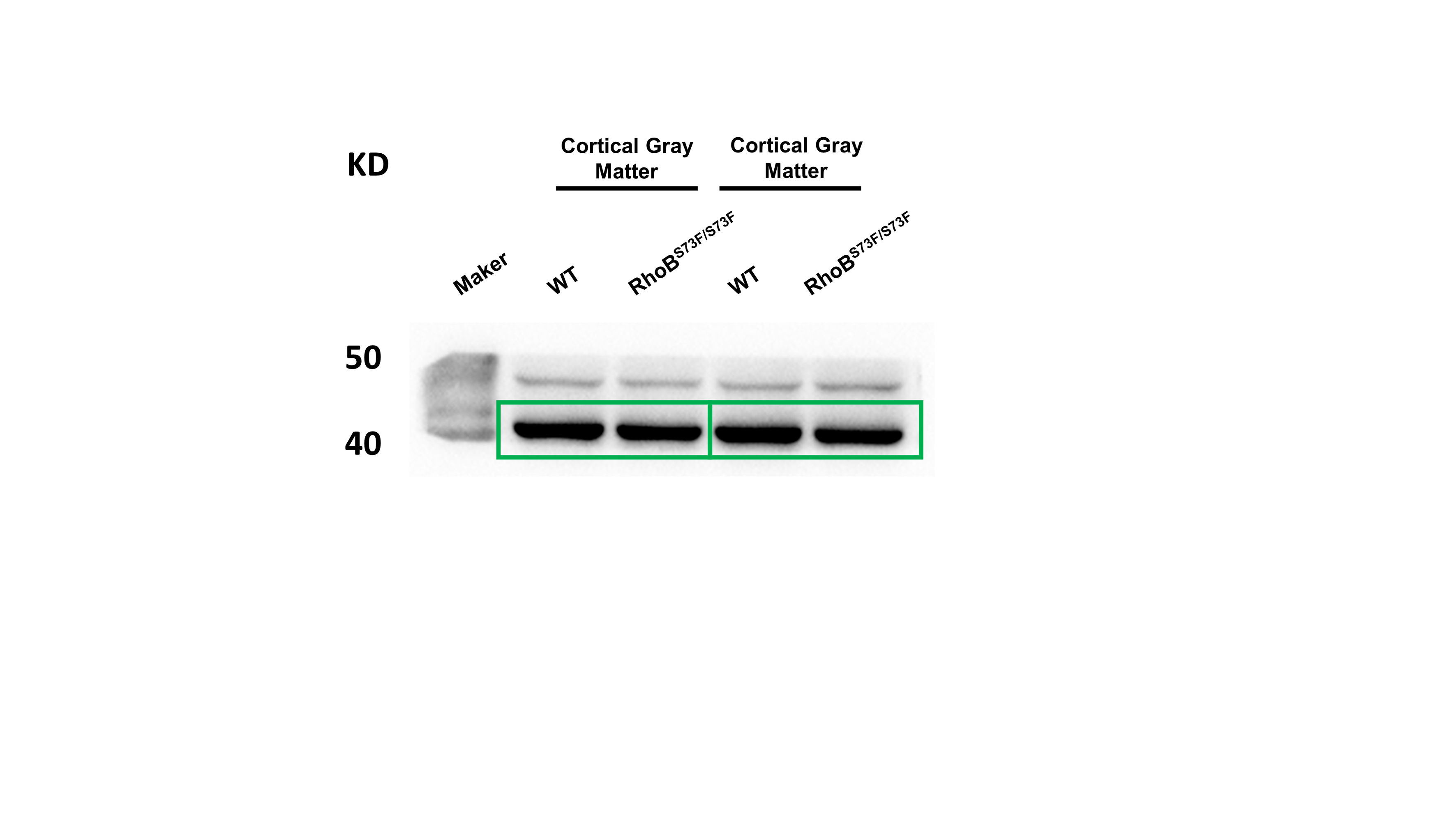

Supplement: Supplementary file 15 — Source data Fig. 5 [file 44321_2024_113_MOESM15_ESM.zip › Figure 5/5D/replicate/western Acat1 in Cortical Gray Matter replicate.tif]

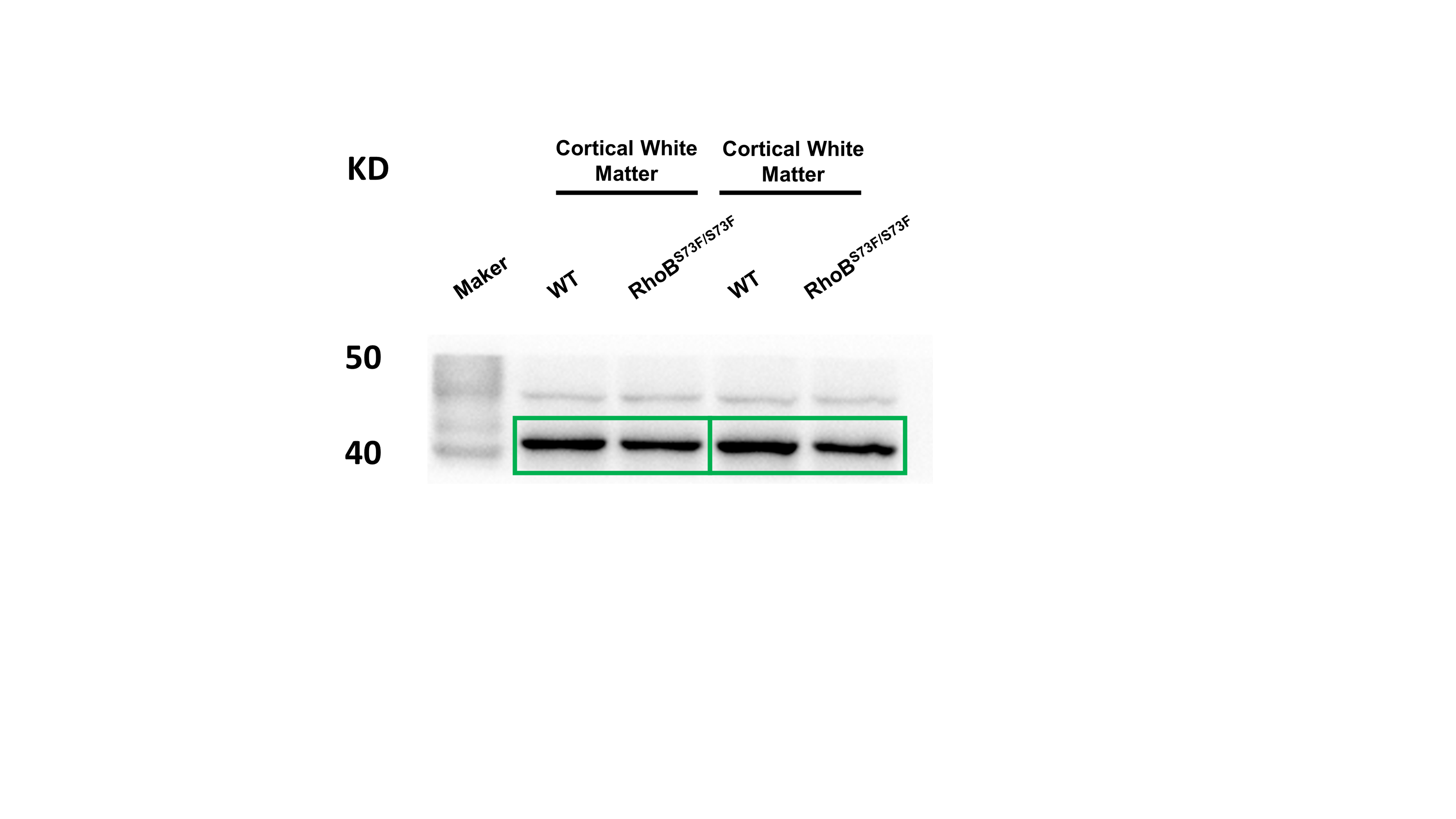

Supplement: Supplementary file 15 — Source data Fig. 5 [file 44321_2024_113_MOESM15_ESM.zip › Figure 5/5D/replicate/western Acat1 in Cortical White Matter replicate.tif]

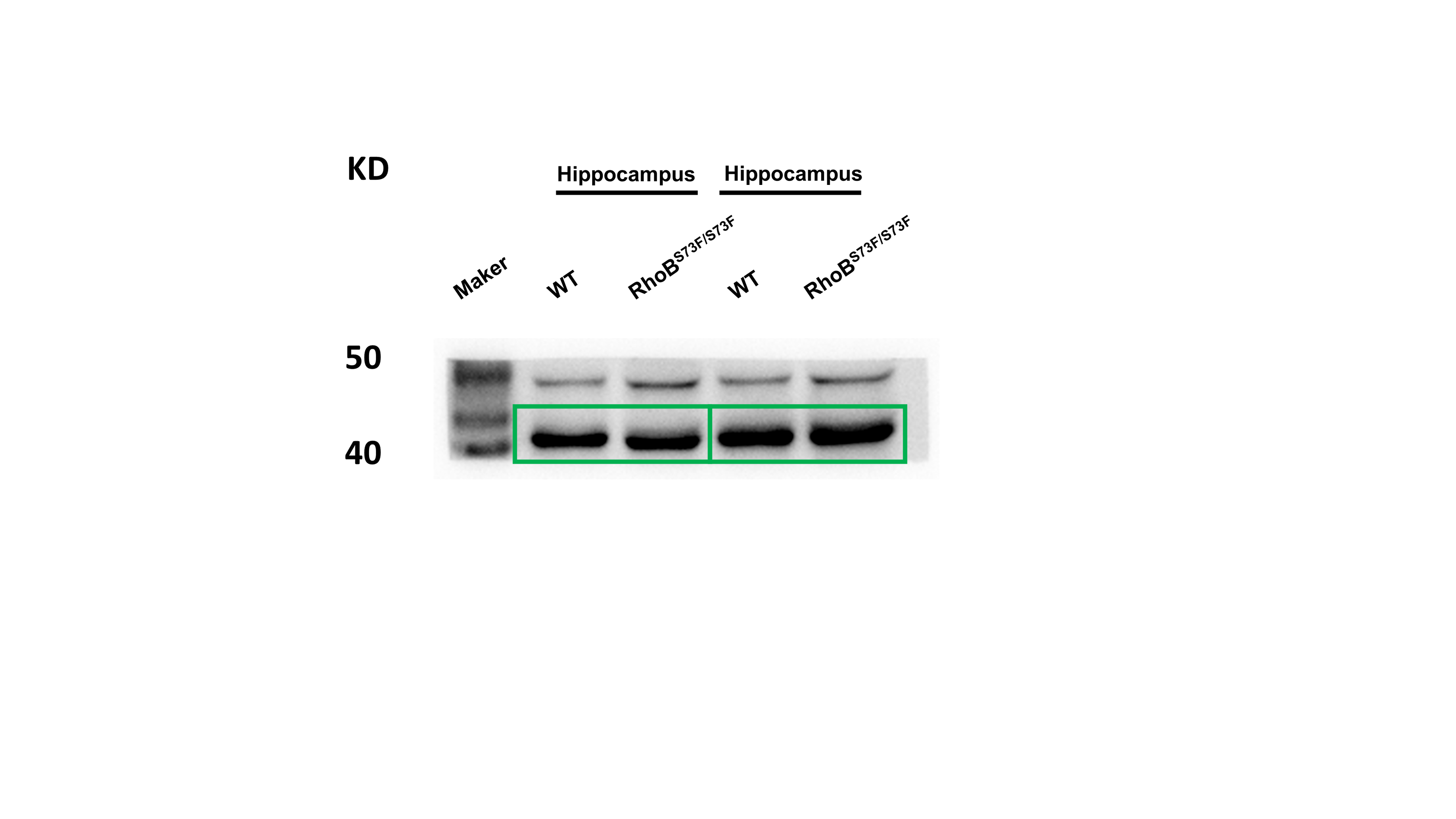

Supplement: Supplementary file 15 — Source data Fig. 5 [file 44321_2024_113_MOESM15_ESM.zip › Figure 5/5D/replicate/western Acat1 in Hippocampus replicate.tif]

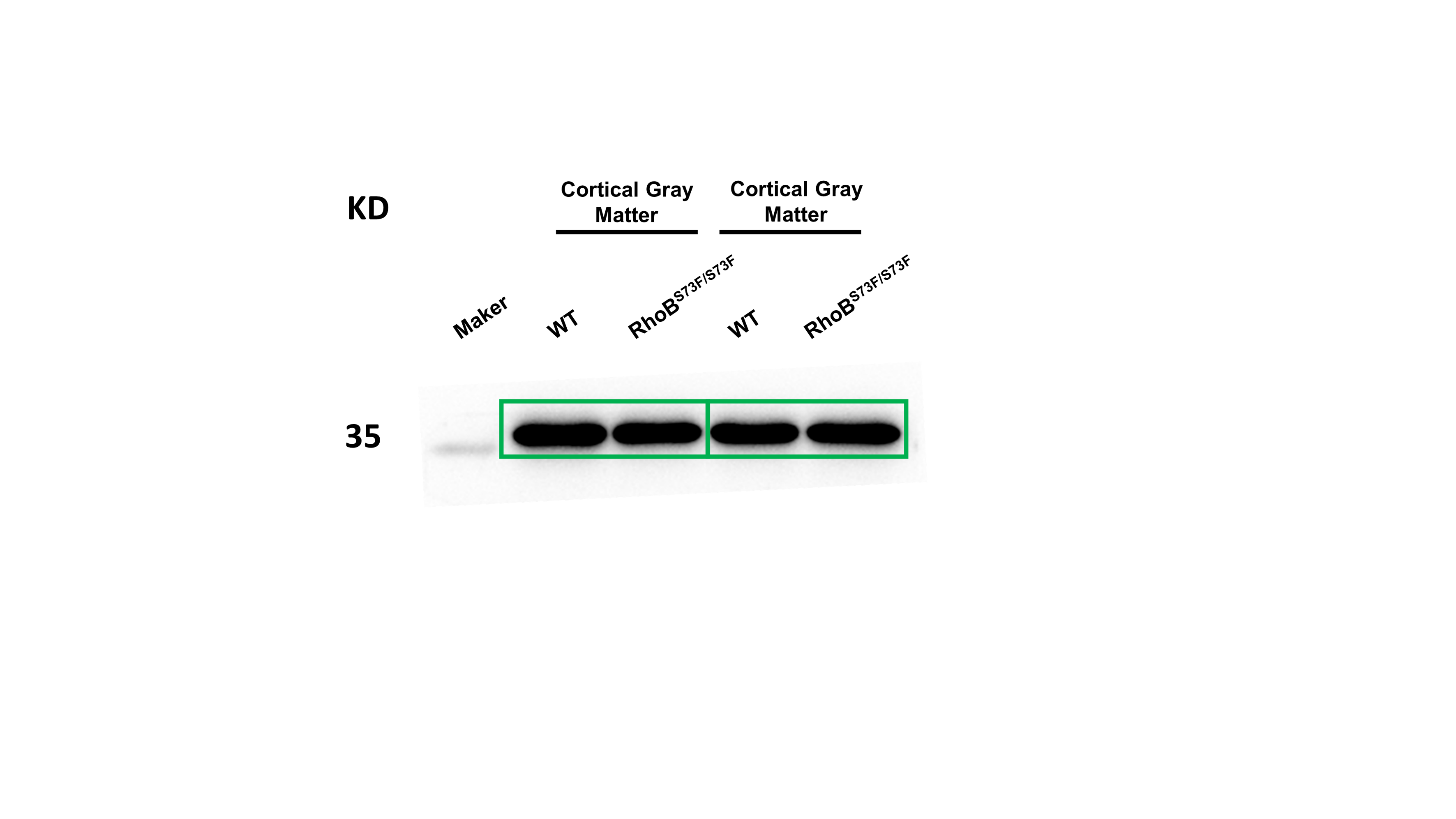

Supplement: Supplementary file 15 — Source data Fig. 5 [file 44321_2024_113_MOESM15_ESM.zip › Figure 5/5D/replicate/western Gapdh in Cortical Gray Matter replicate.tif]

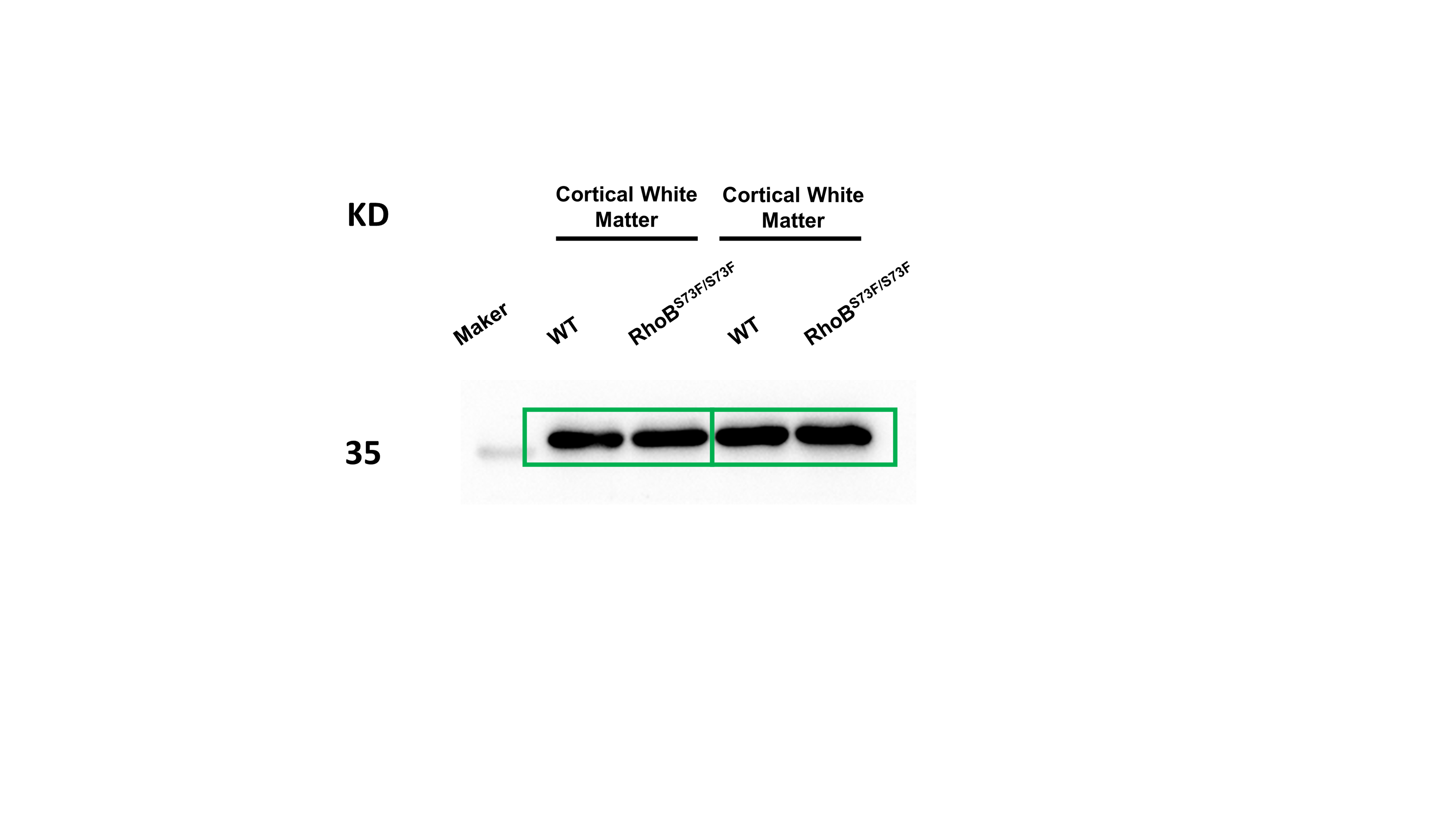

Supplement: Supplementary file 15 — Source data Fig. 5 [file 44321_2024_113_MOESM15_ESM.zip › Figure 5/5D/replicate/western Gapdh in Cortical White Matter replicate.tif]

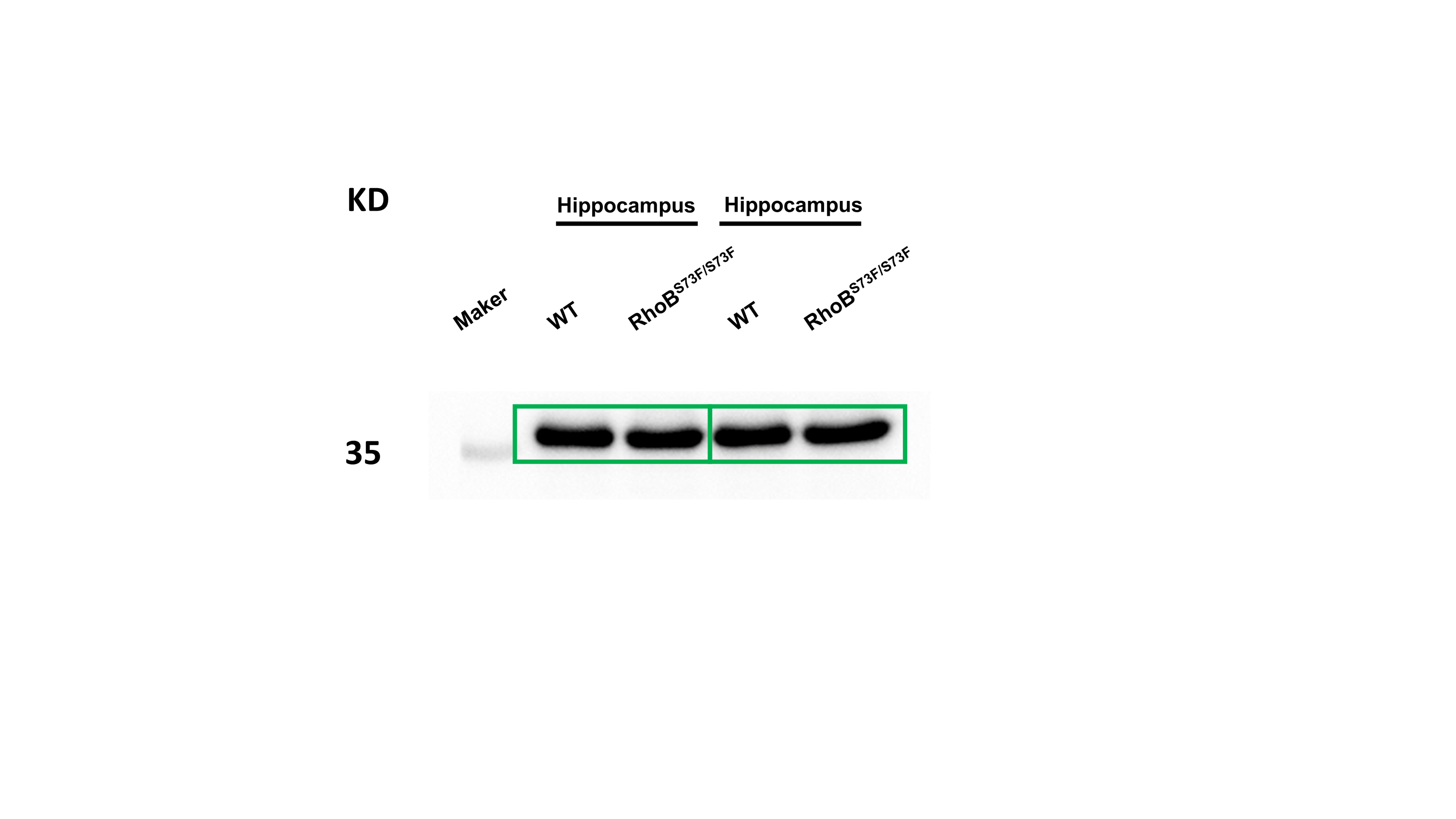

Supplement: Supplementary file 15 — Source data Fig. 5 [file 44321_2024_113_MOESM15_ESM.zip › Figure 5/5D/replicate/western Gapdh in Hippocampus replicate.tif]

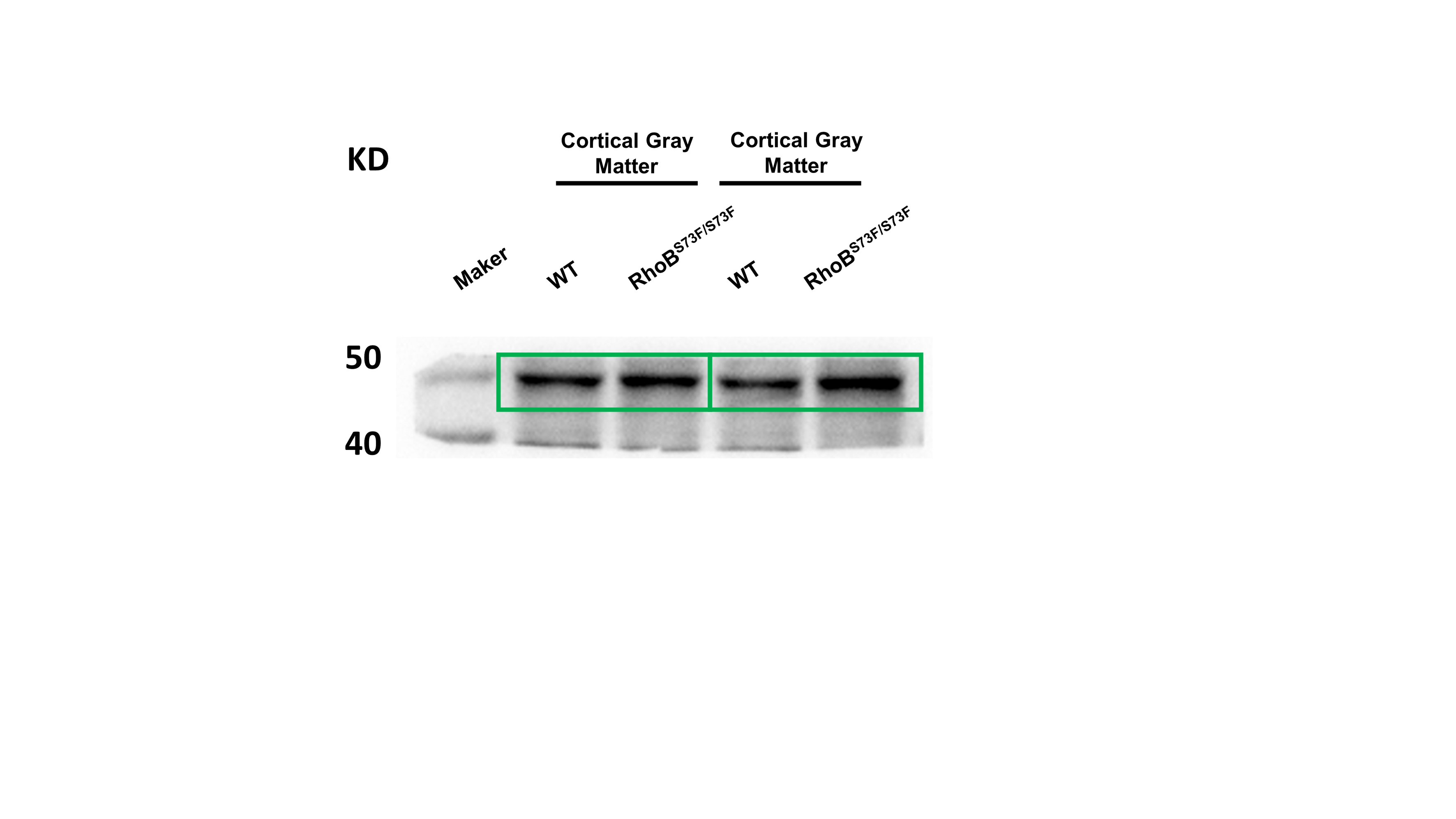

Supplement: Supplementary file 15 — Source data Fig. 5 [file 44321_2024_113_MOESM15_ESM.zip › Figure 5/5D/replicate/western P-Acat1 in Cortical Gray Matter replicate.tif]

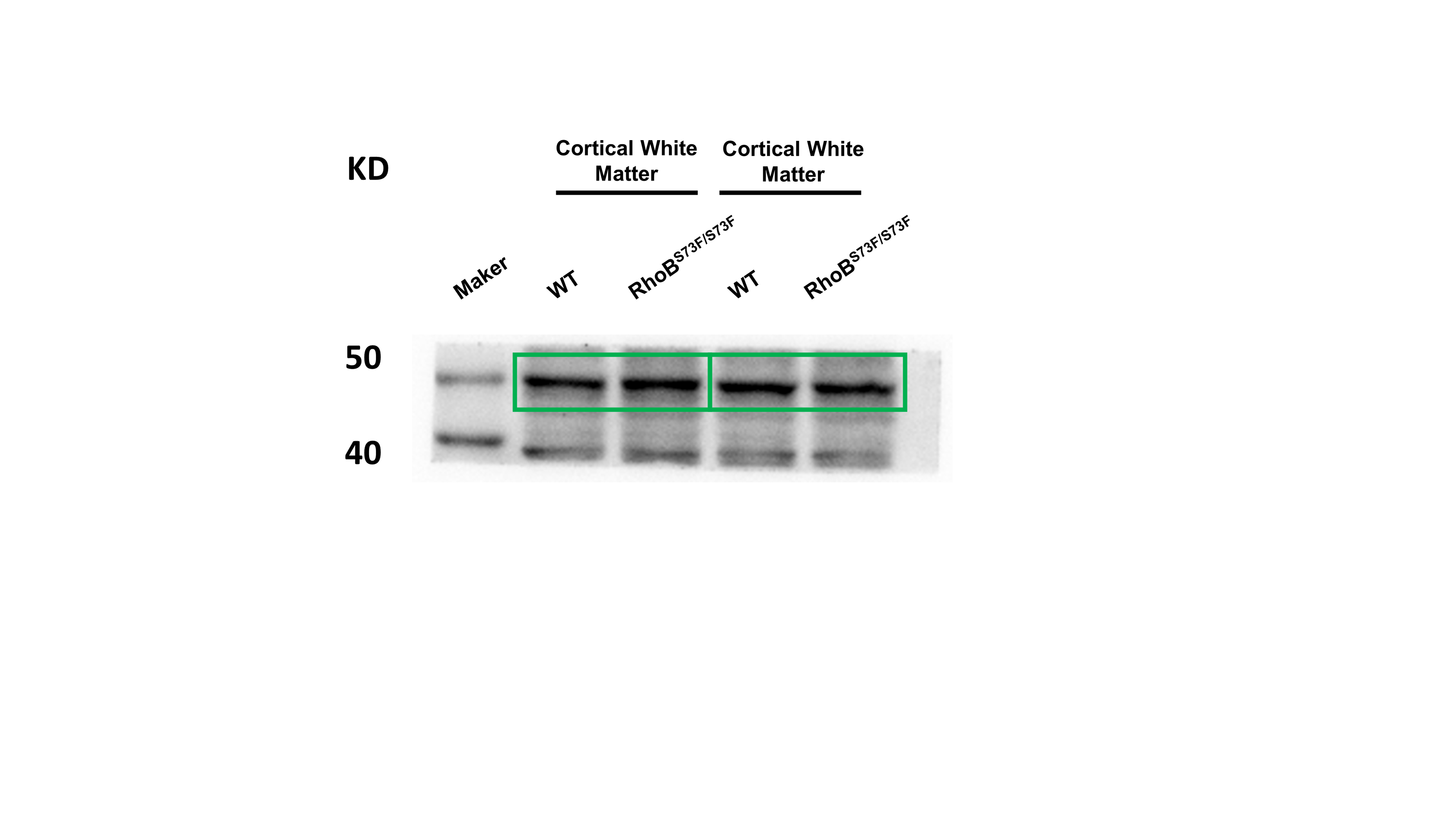

Supplement: Supplementary file 15 — Source data Fig. 5 [file 44321_2024_113_MOESM15_ESM.zip › Figure 5/5D/replicate/western P-Acat1 in Cortical White Matter replicate.tif]

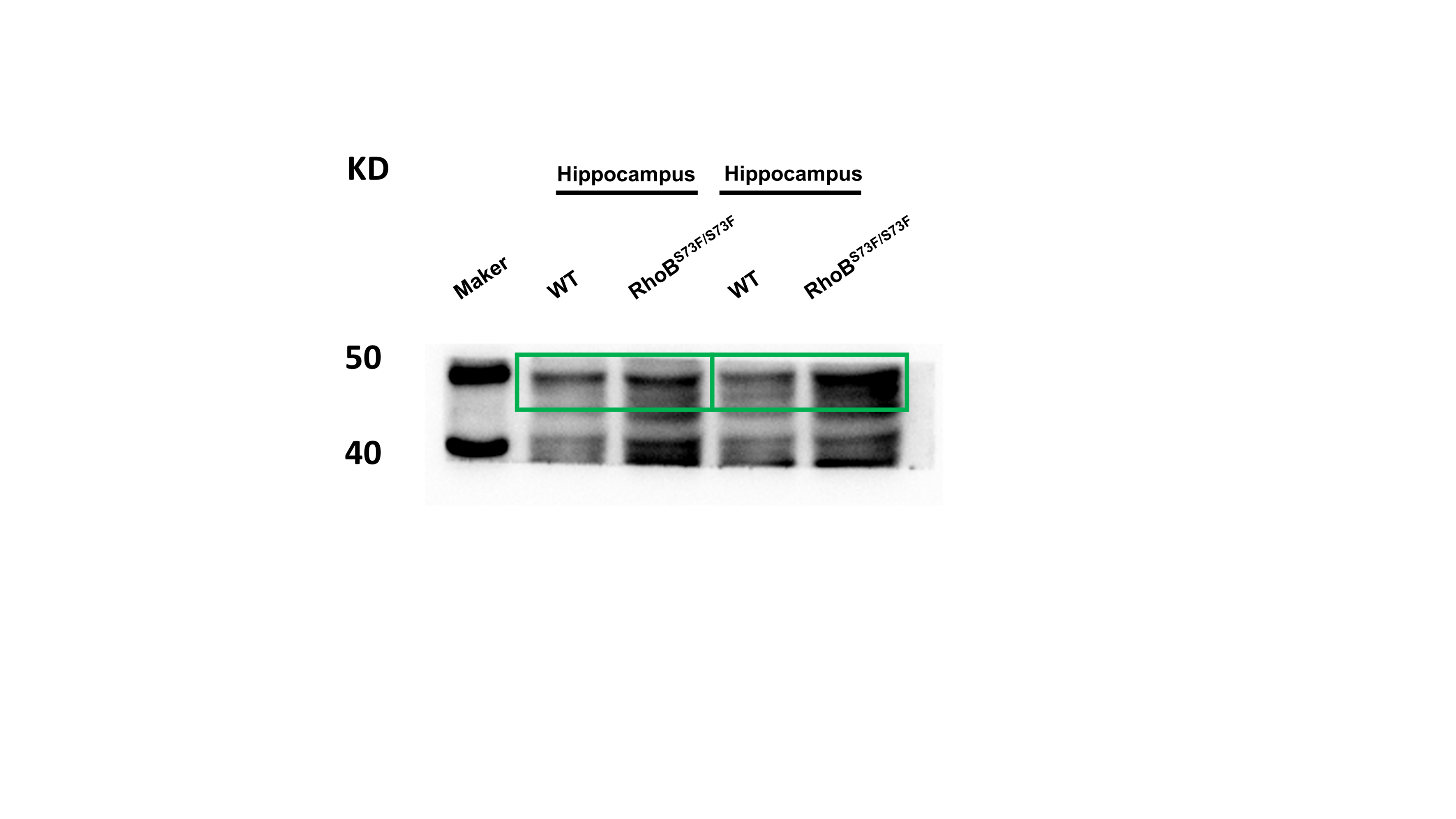

Supplement: Supplementary file 15 — Source data Fig. 5 [file 44321_2024_113_MOESM15_ESM.zip › Figure 5/5D/replicate/western P-Acat1 in Hippocampus replicate.tif]

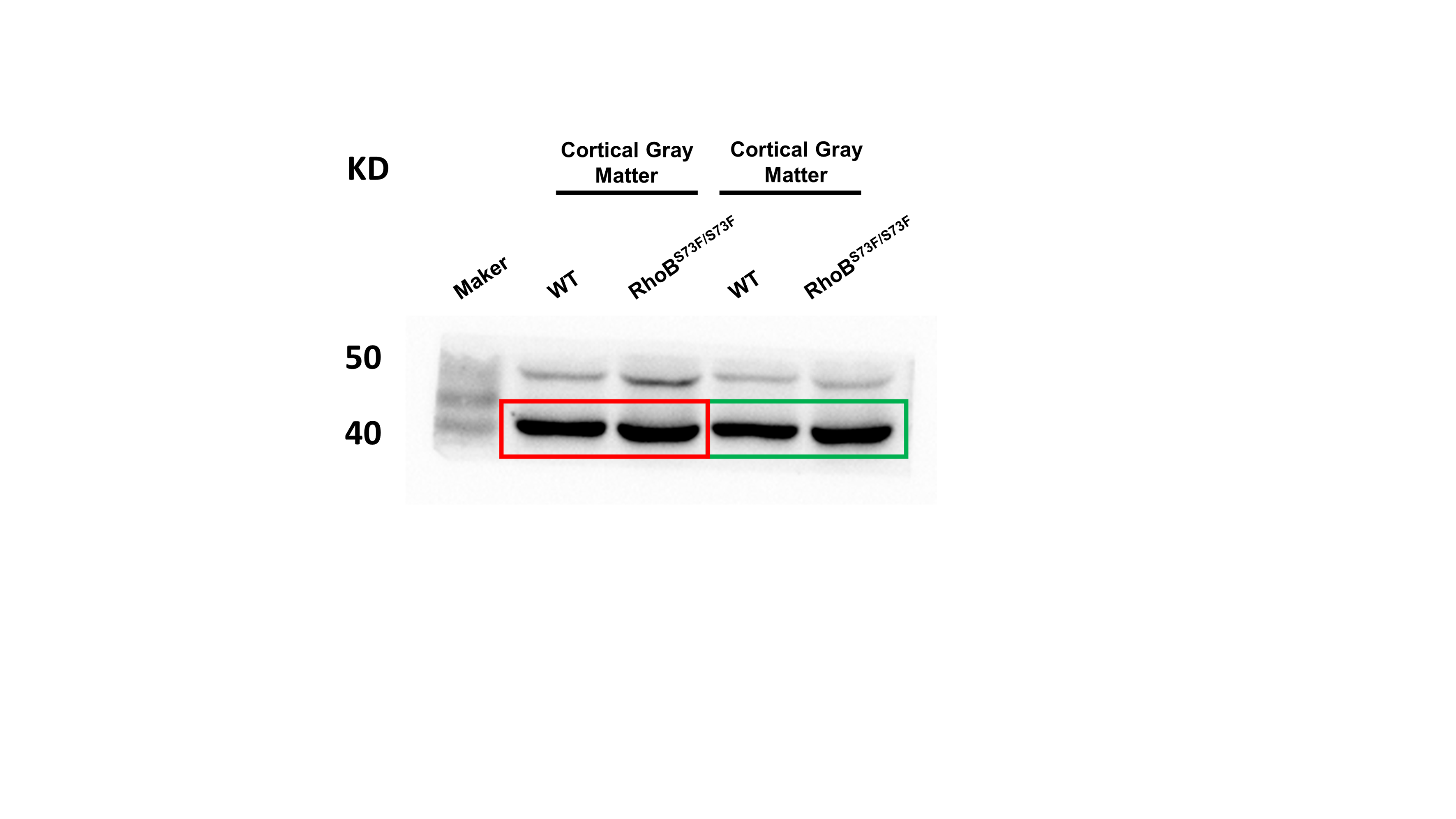

Supplement: Supplementary file 15 — Source data Fig. 5 [file 44321_2024_113_MOESM15_ESM.zip › Figure 5/5D/western Acat1 in Cortical Gray Matter.tif]

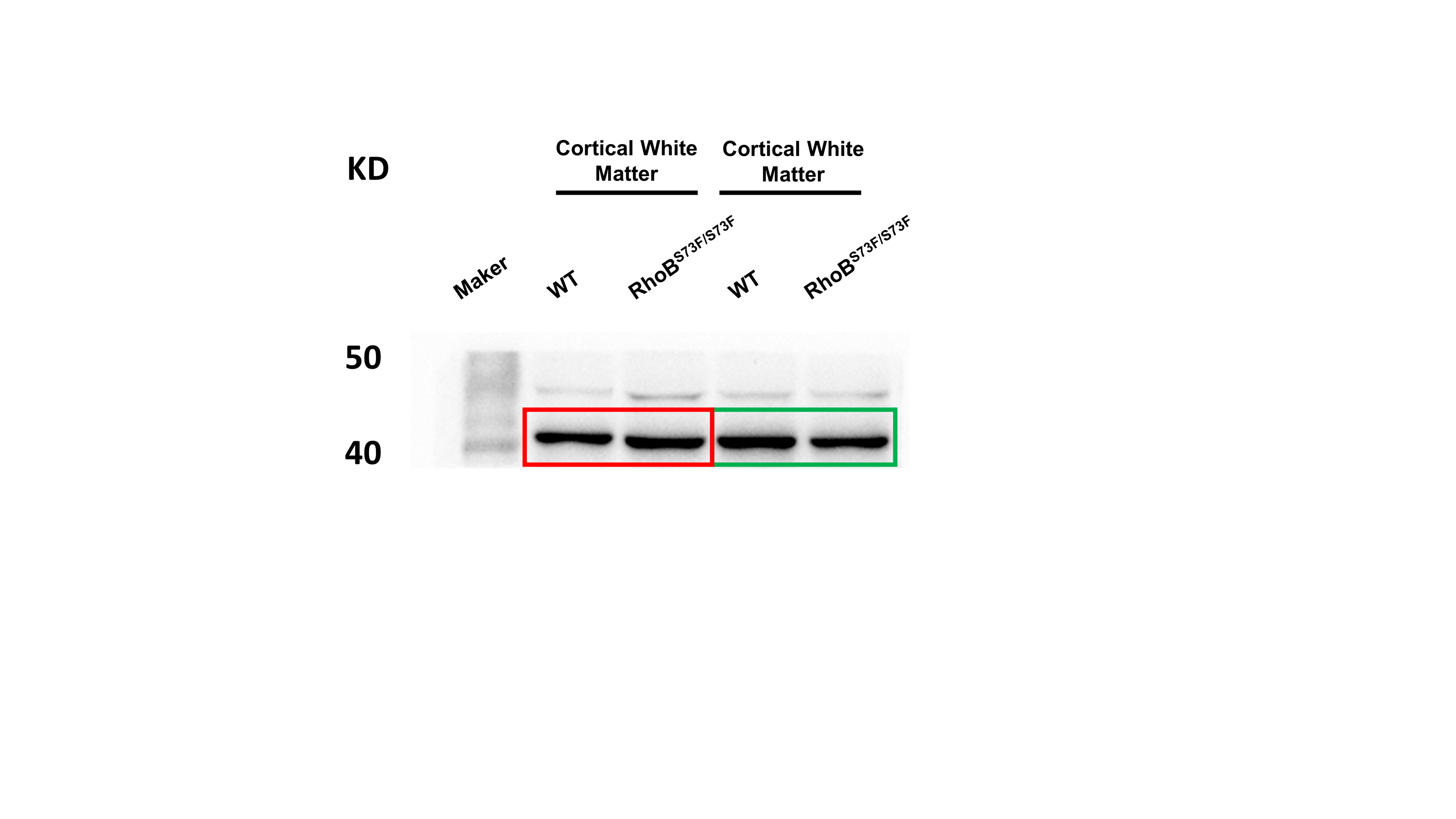

Supplement: Supplementary file 15 — Source data Fig. 5 [file 44321_2024_113_MOESM15_ESM.zip › Figure 5/5D/western Acat1 in Cortical White Matter.tif]

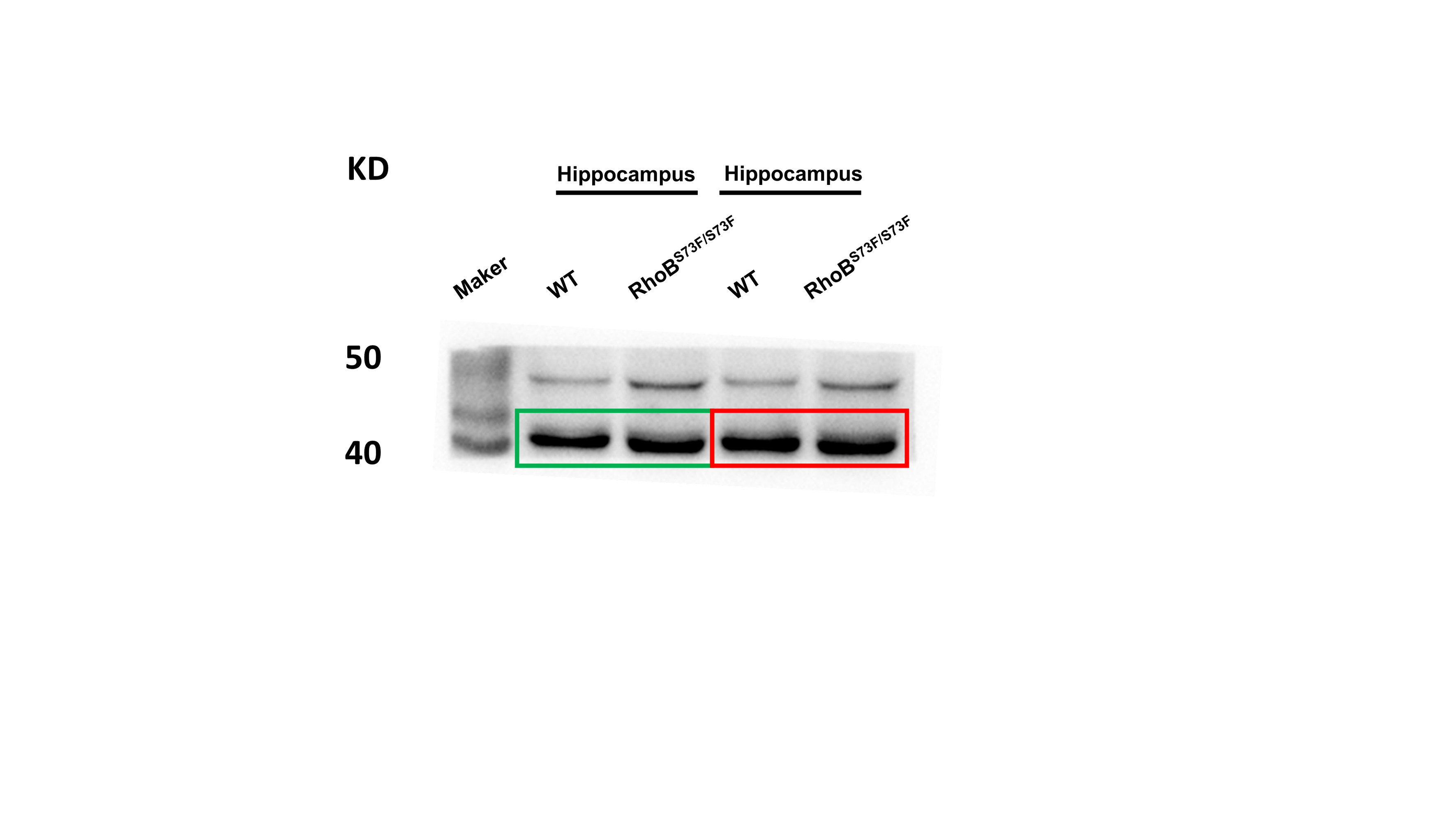

Supplement: Supplementary file 15 — Source data Fig. 5 [file 44321_2024_113_MOESM15_ESM.zip › Figure 5/5D/western Acat1 in Hippocampus.tif]

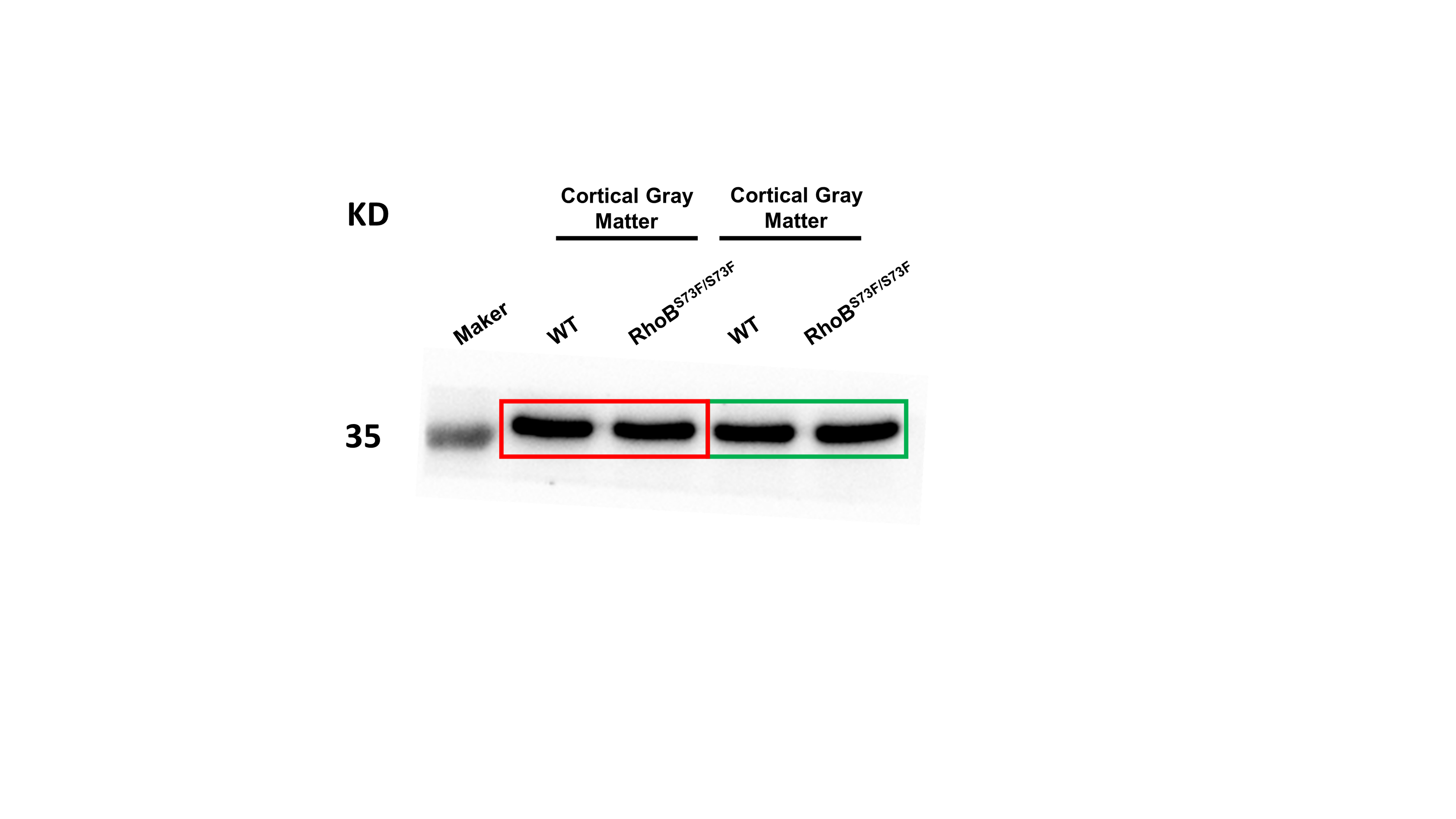

Supplement: Supplementary file 15 — Source data Fig. 5 [file 44321_2024_113_MOESM15_ESM.zip › Figure 5/5D/western Gapdh in Cortical Gray Matter.tif]

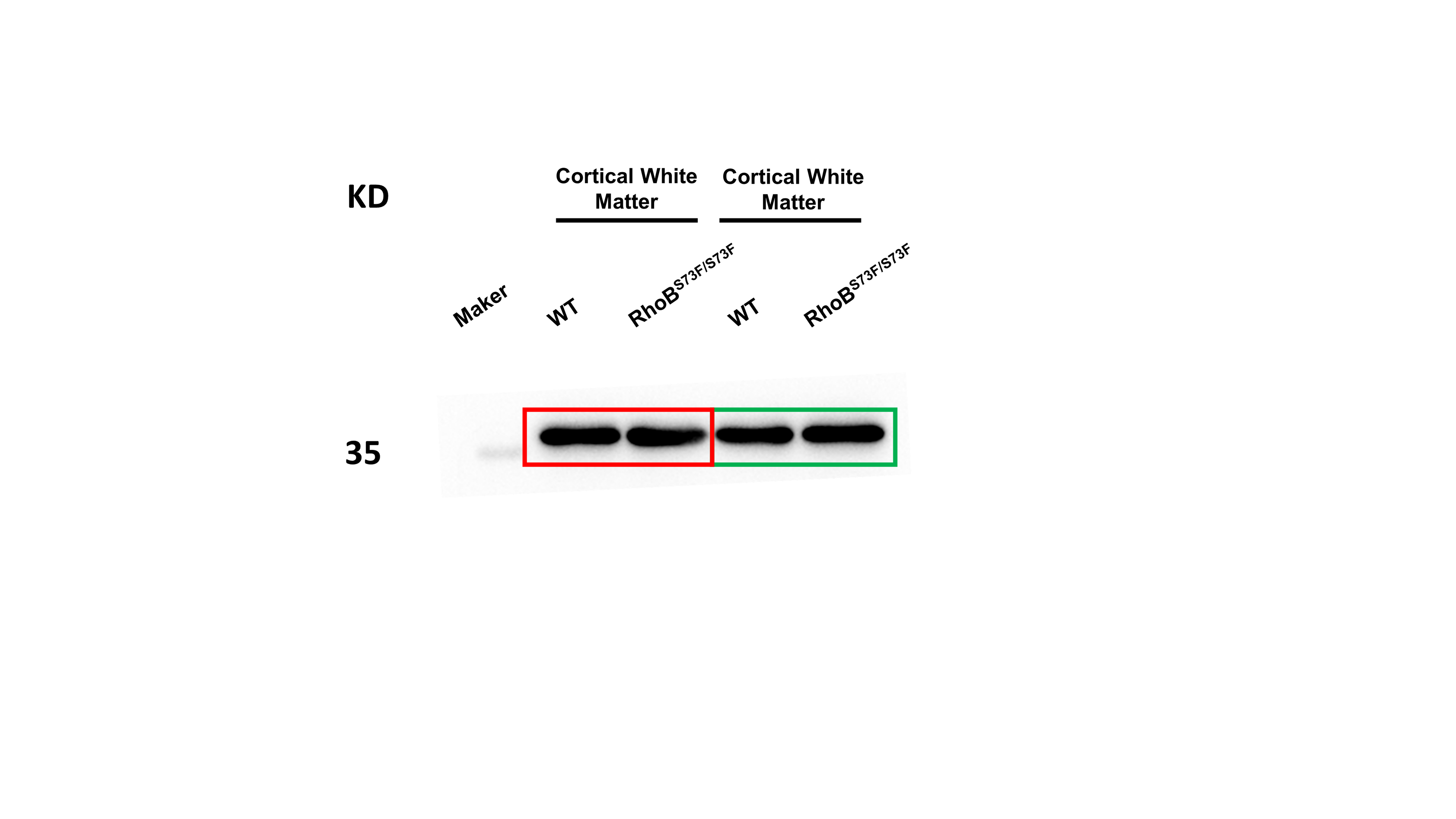

Supplement: Supplementary file 15 — Source data Fig. 5 [file 44321_2024_113_MOESM15_ESM.zip › Figure 5/5D/western Gapdh in Cortical White Matter.tif]

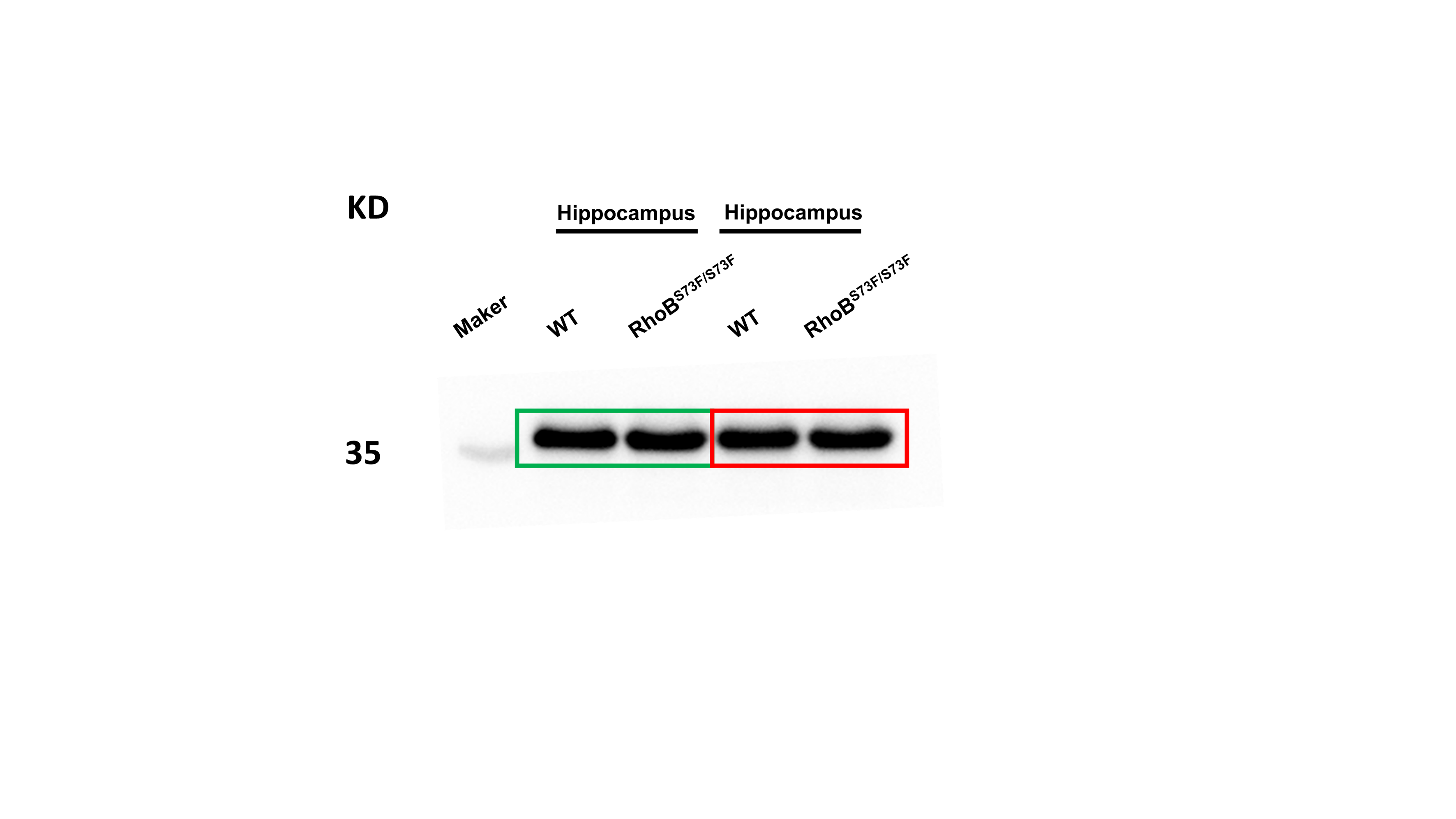

Supplement: Supplementary file 15 — Source data Fig. 5 [file 44321_2024_113_MOESM15_ESM.zip › Figure 5/5D/western Gapdh in Hippocampus.tif]

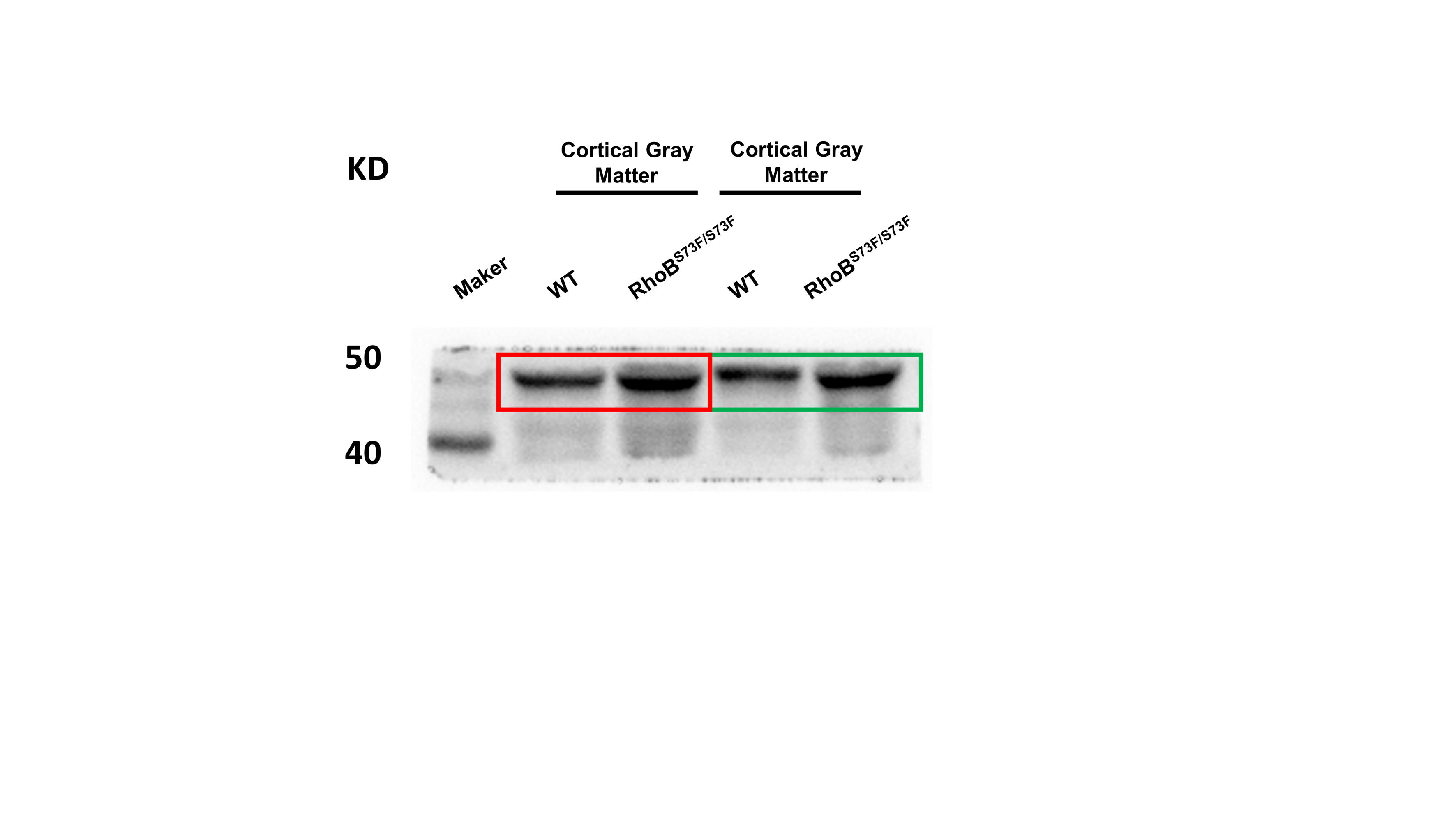

Supplement: Supplementary file 15 — Source data Fig. 5 [file 44321_2024_113_MOESM15_ESM.zip › Figure 5/5D/western P-Acat1 in Cortical Gray Matter.tif]

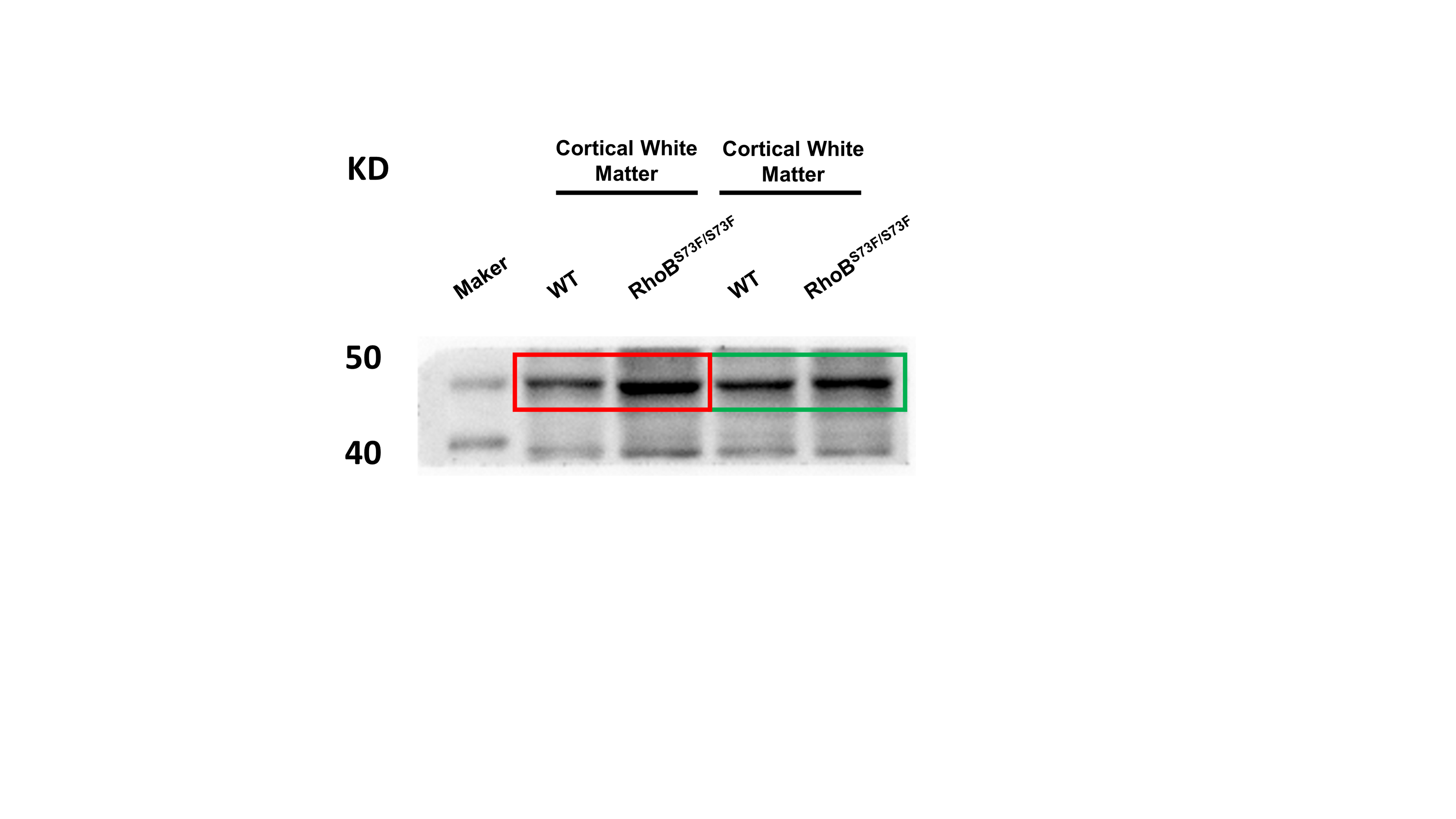

Supplement: Supplementary file 15 — Source data Fig. 5 [file 44321_2024_113_MOESM15_ESM.zip › Figure 5/5D/western P-Acat1 in Cortical White Matter.tif]

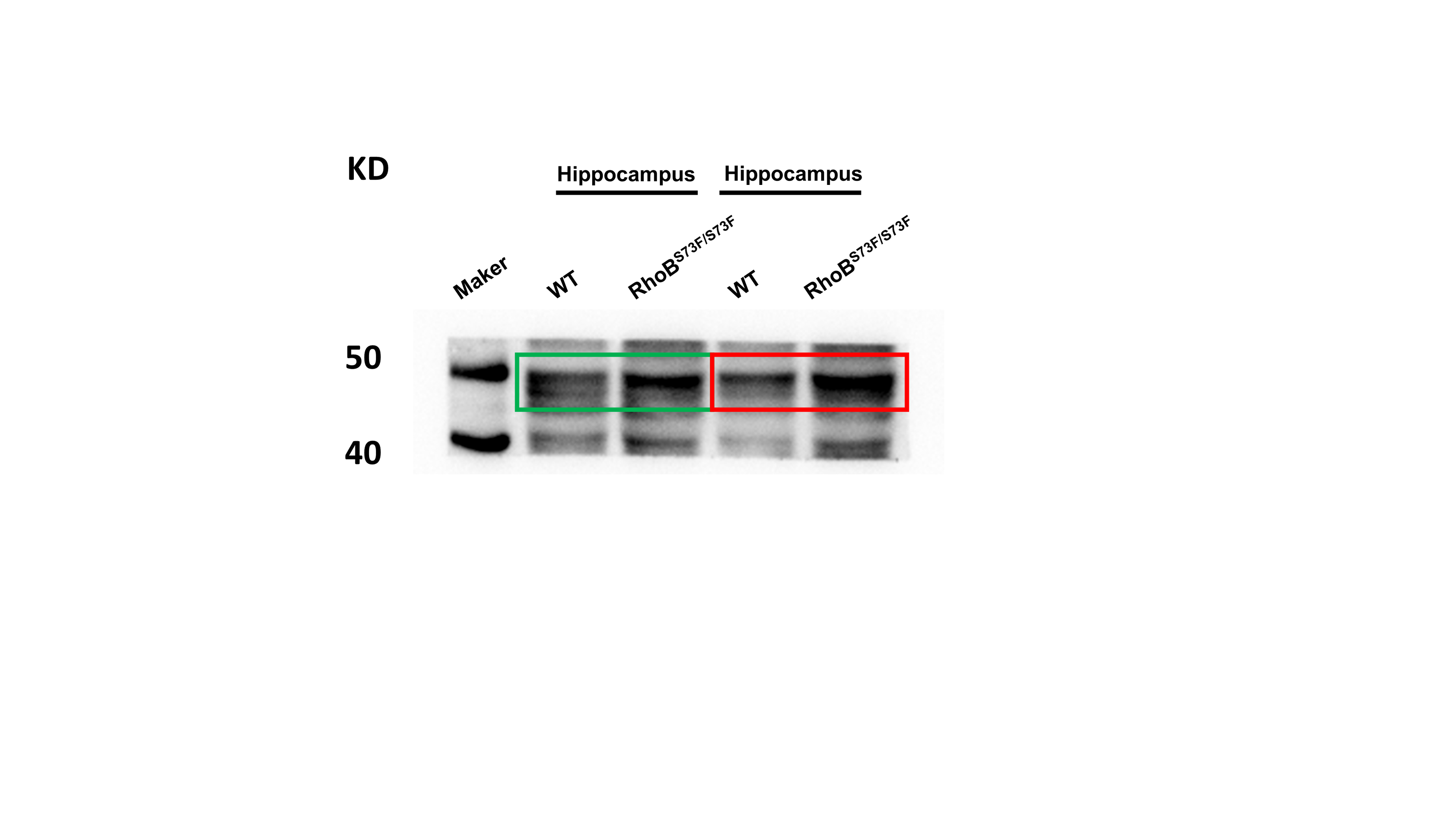

Supplement: Supplementary file 15 — Source data Fig. 5 [file 44321_2024_113_MOESM15_ESM.zip › Figure 5/5D/western P-Acat1 in Hippocampus.tif]

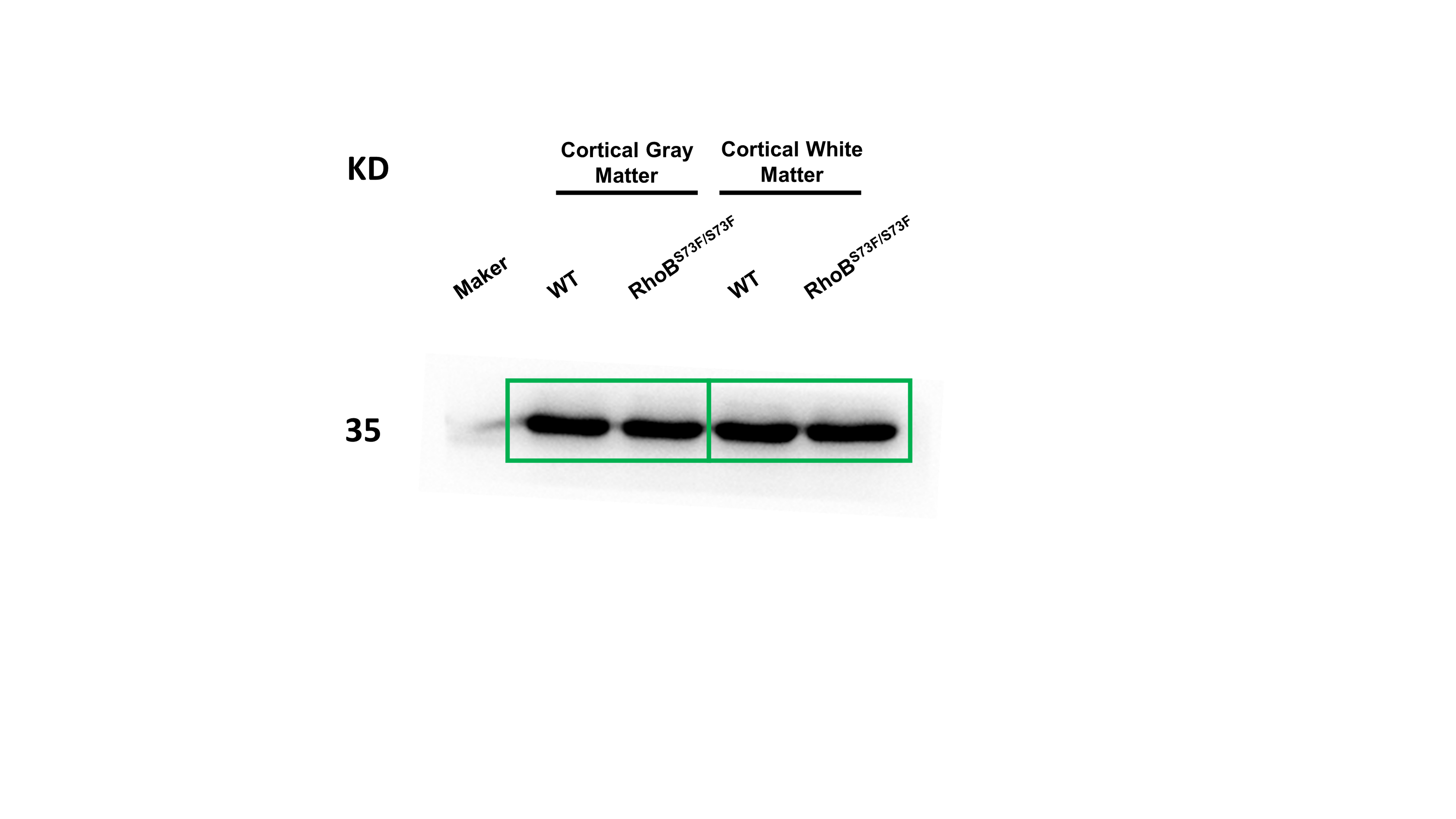

Supplement: Supplementary file 15 — Source data Fig. 5 [file 44321_2024_113_MOESM15_ESM.zip › Figure 5/5E/replicate/western Gapdh in Cortical Gray Matter&Cortical White Matter replicate.tif]

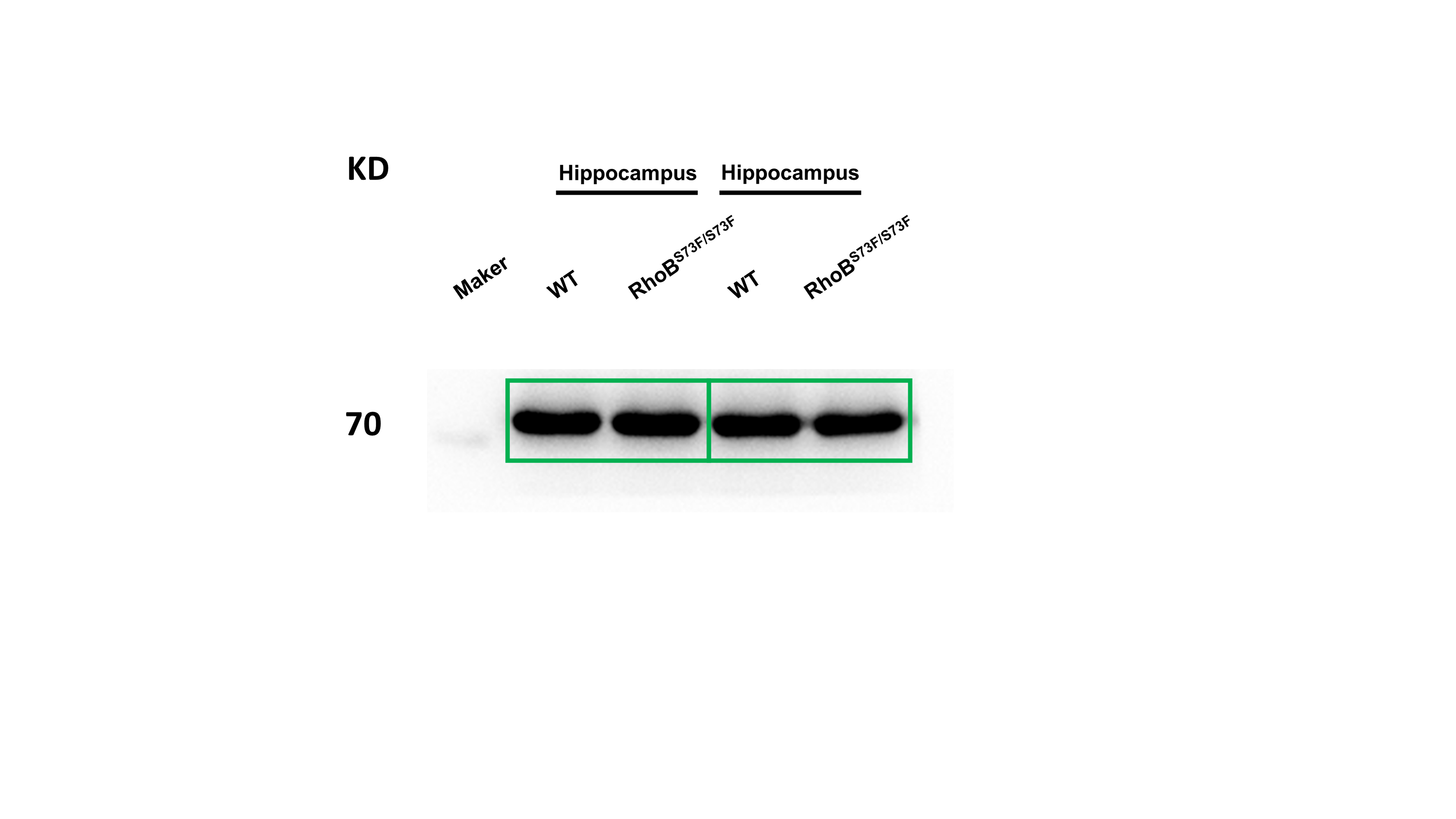

Supplement: Supplementary file 15 — Source data Fig. 5 [file 44321_2024_113_MOESM15_ESM.zip › Figure 5/5E/replicate/western Gapdh in Hippocampus replicate.tif]

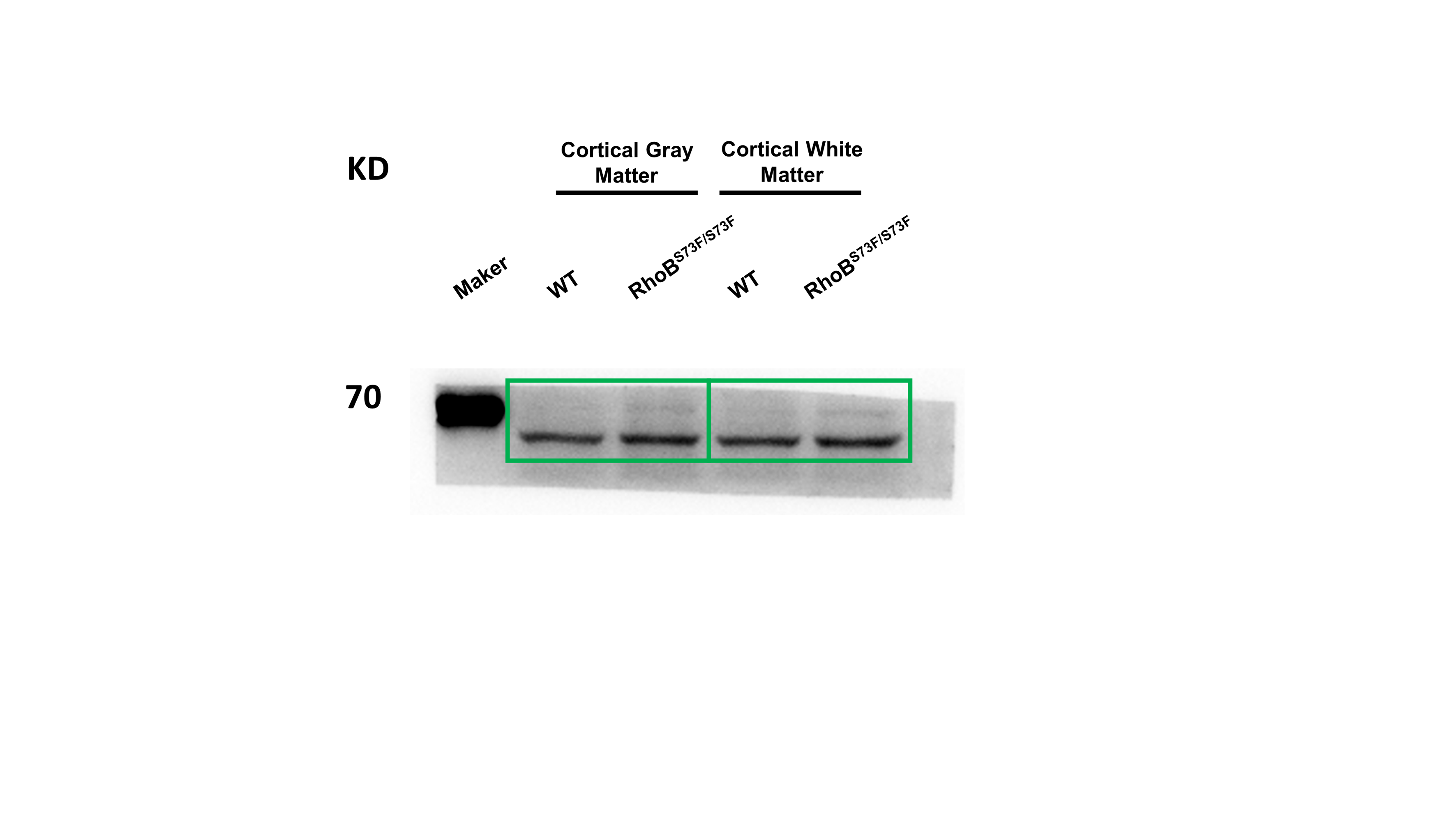

Supplement: Supplementary file 15 — Source data Fig. 5 [file 44321_2024_113_MOESM15_ESM.zip › Figure 5/5E/replicate/western LYN in Cortical Gray Matter&Cortical White Matter replicate.tif]

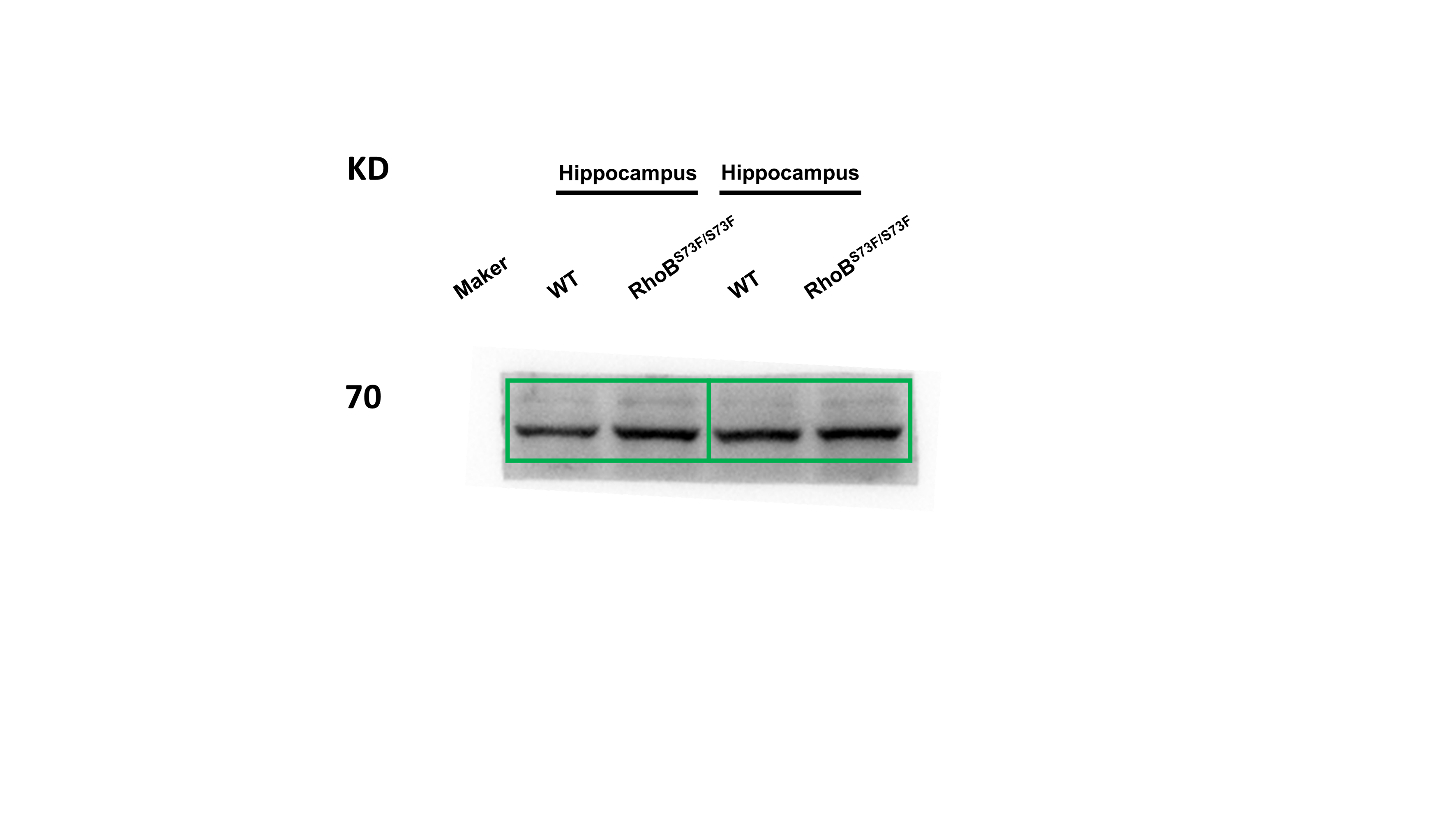

Supplement: Supplementary file 15 — Source data Fig. 5 [file 44321_2024_113_MOESM15_ESM.zip › Figure 5/5E/replicate/western LYN in Hippocampus replicate.tif]

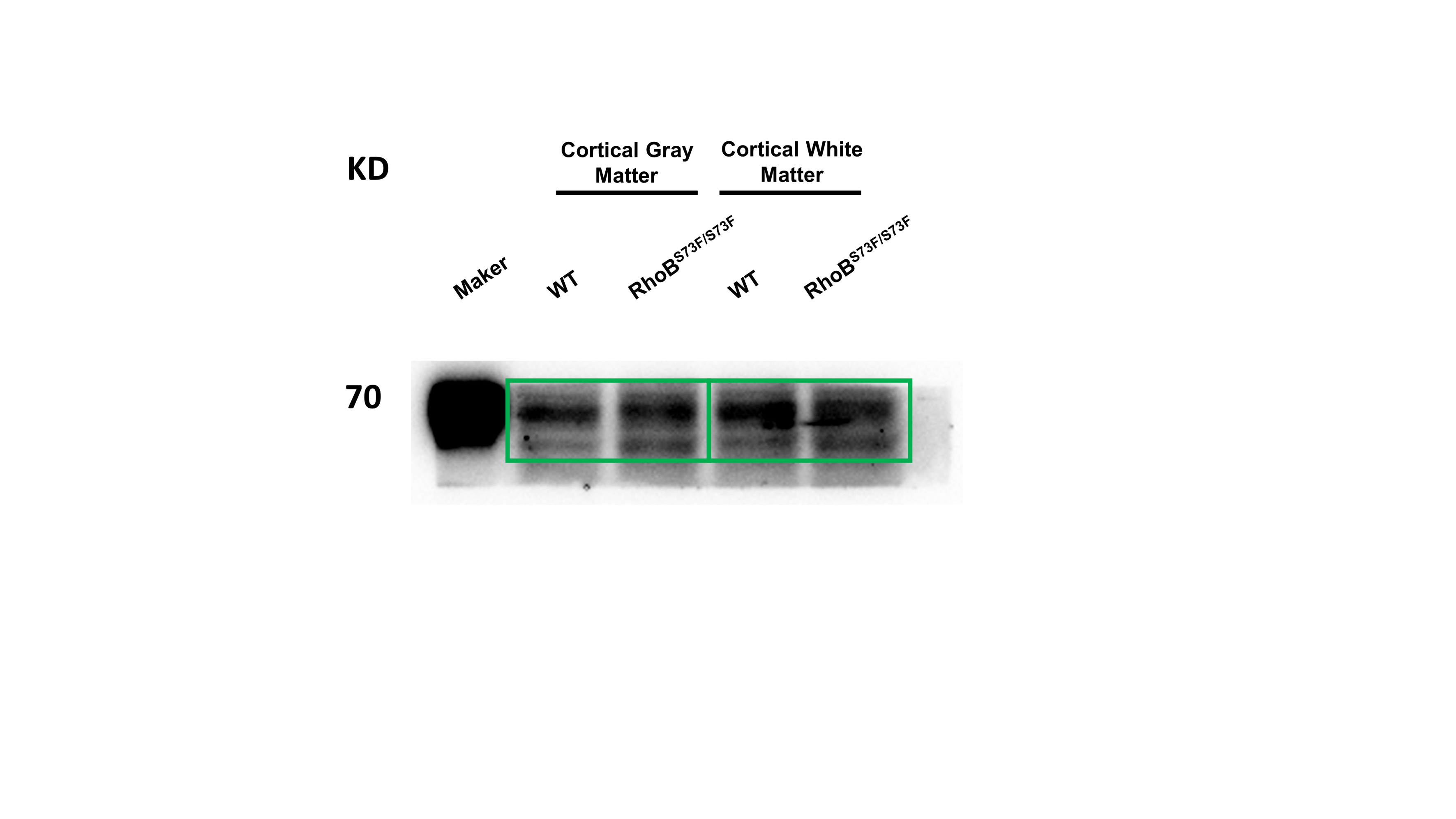

Supplement: Supplementary file 15 — Source data Fig. 5 [file 44321_2024_113_MOESM15_ESM.zip › Figure 5/5E/replicate/western P-LYN in Cortical Gray Matter&Cortical White Matter replicate.tif]

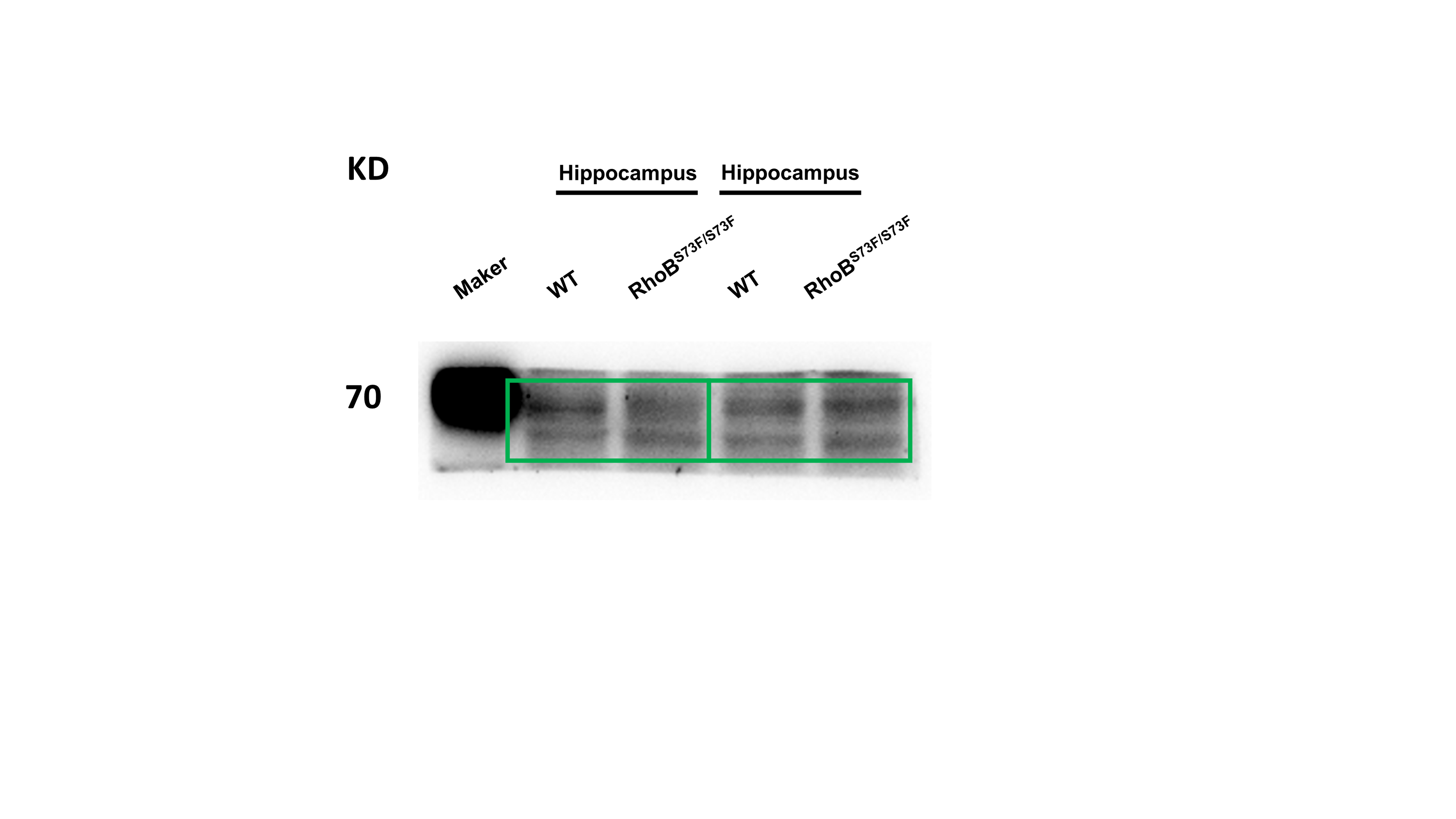

Supplement: Supplementary file 15 — Source data Fig. 5 [file 44321_2024_113_MOESM15_ESM.zip › Figure 5/5E/replicate/western P-LYN in Hippocampus replicate.tif]

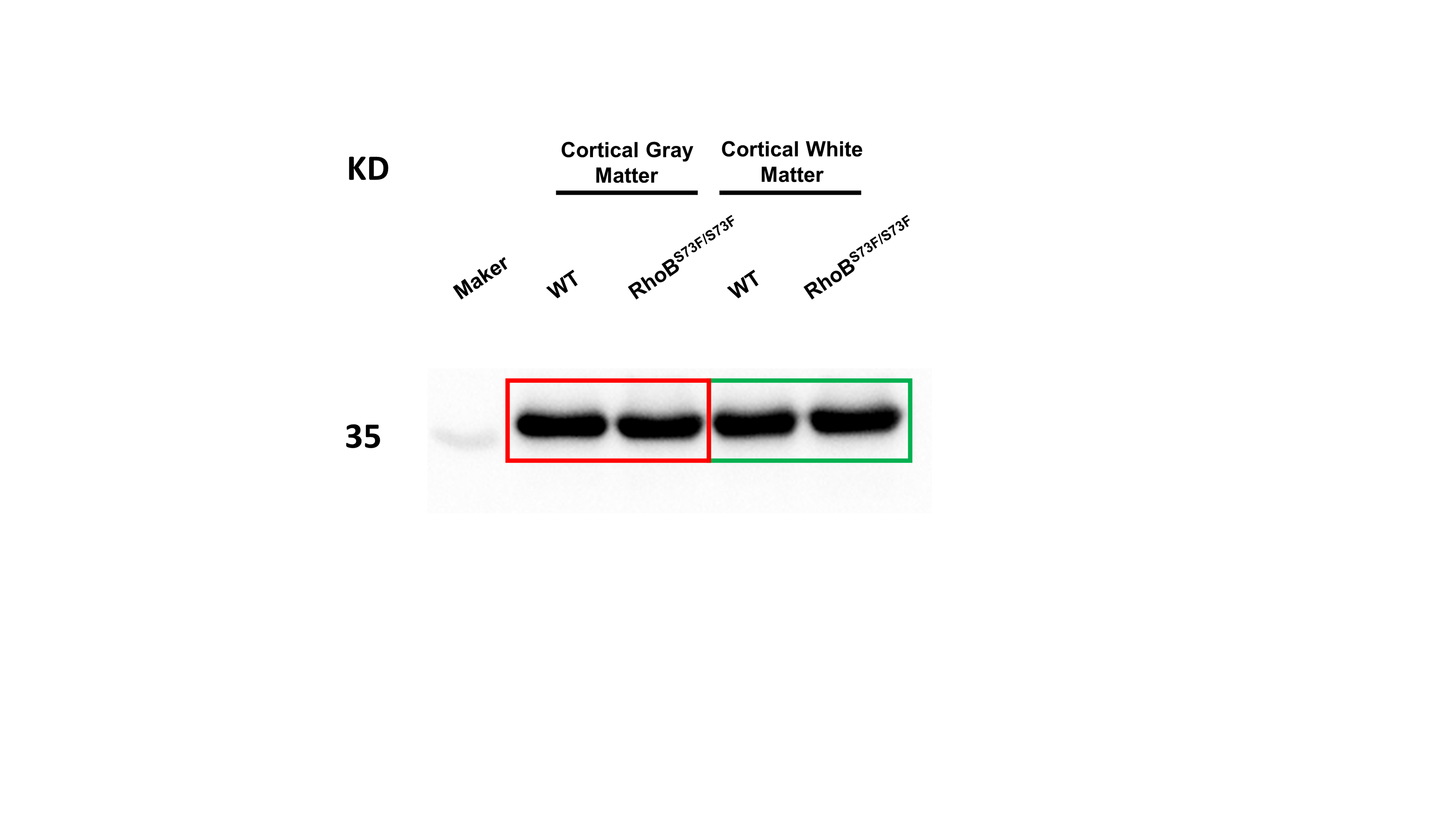

Supplement: Supplementary file 15 — Source data Fig. 5 [file 44321_2024_113_MOESM15_ESM.zip › Figure 5/5E/western Gapdh in Cortical Gray Matter.tif]

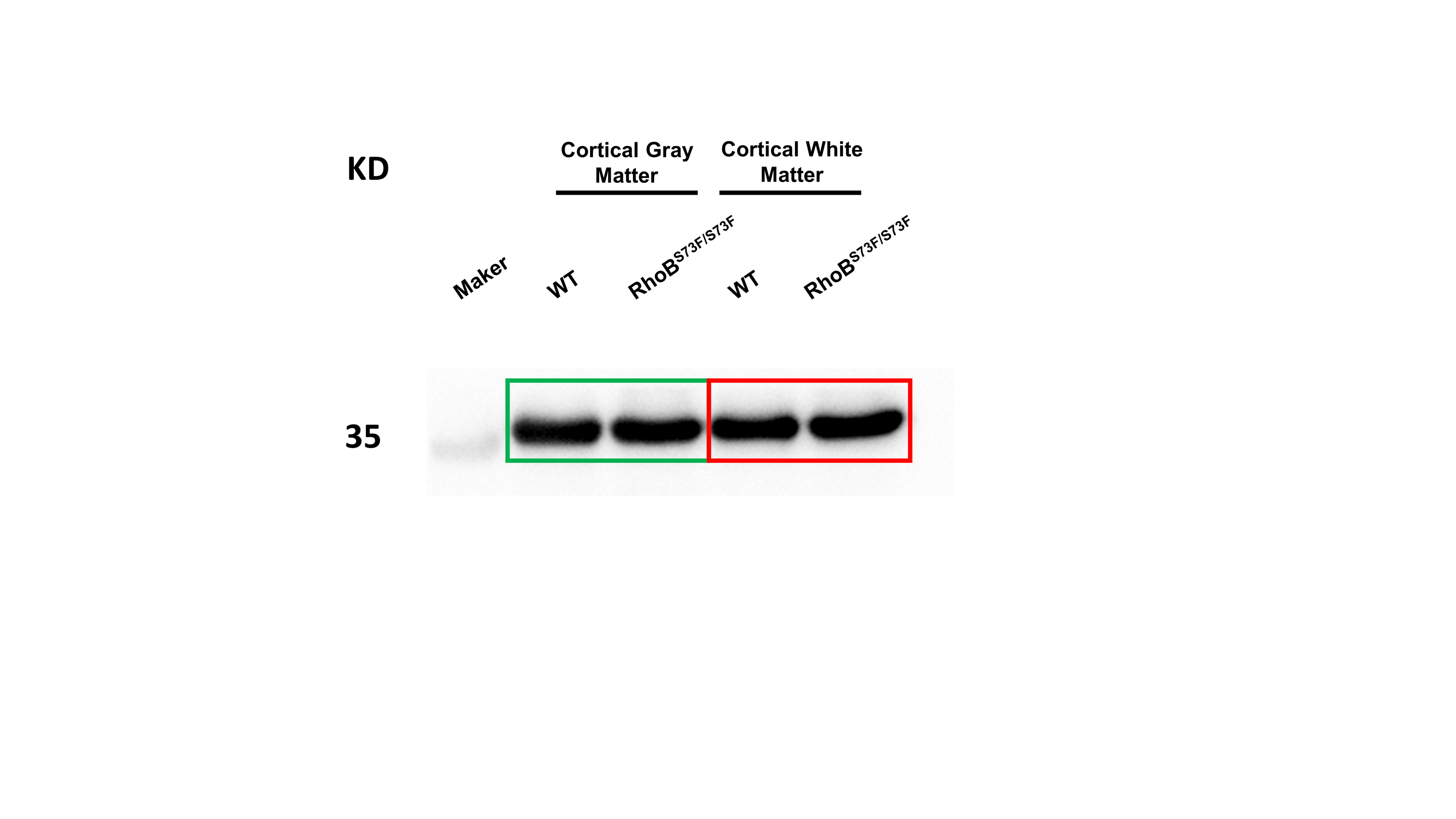

Supplement: Supplementary file 15 — Source data Fig. 5 [file 44321_2024_113_MOESM15_ESM.zip › Figure 5/5E/western Gapdh in Cortical White Matter.tif]

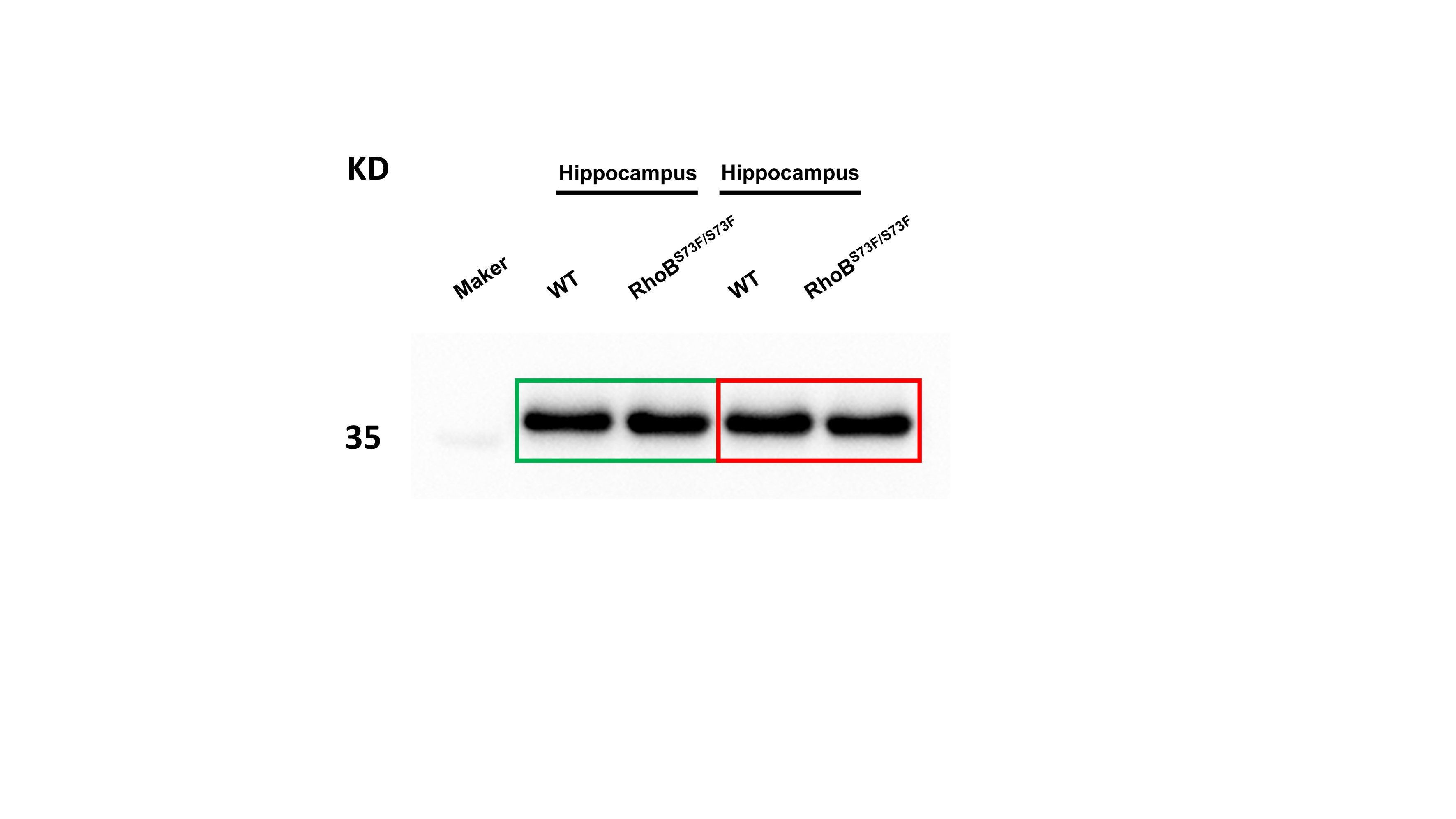

Supplement: Supplementary file 15 — Source data Fig. 5 [file 44321_2024_113_MOESM15_ESM.zip › Figure 5/5E/western Gapdh in Hippocampus.tif]

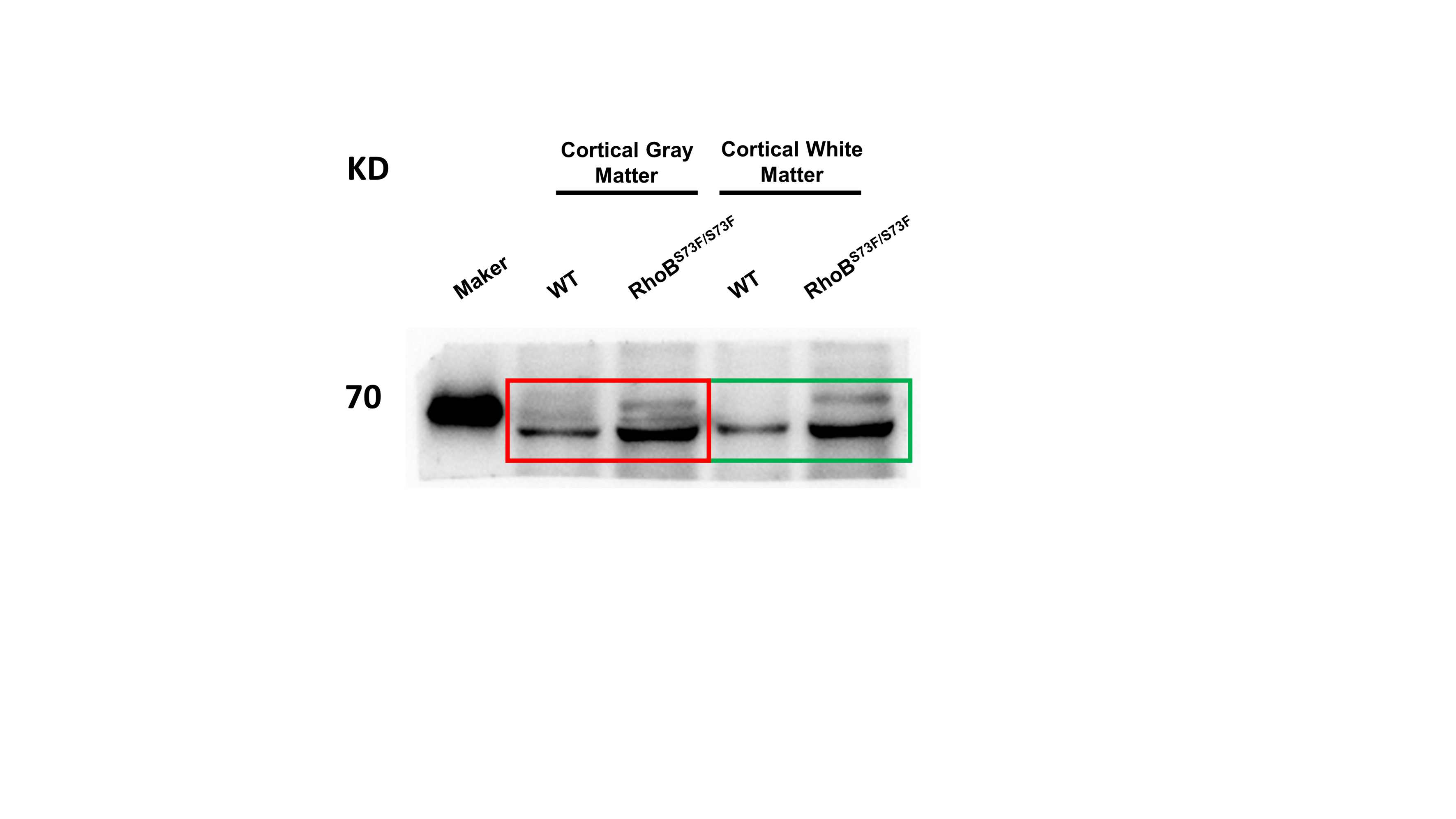

Supplement: Supplementary file 15 — Source data Fig. 5 [file 44321_2024_113_MOESM15_ESM.zip › Figure 5/5E/western LYN in Cortical Gray Matter.tif]

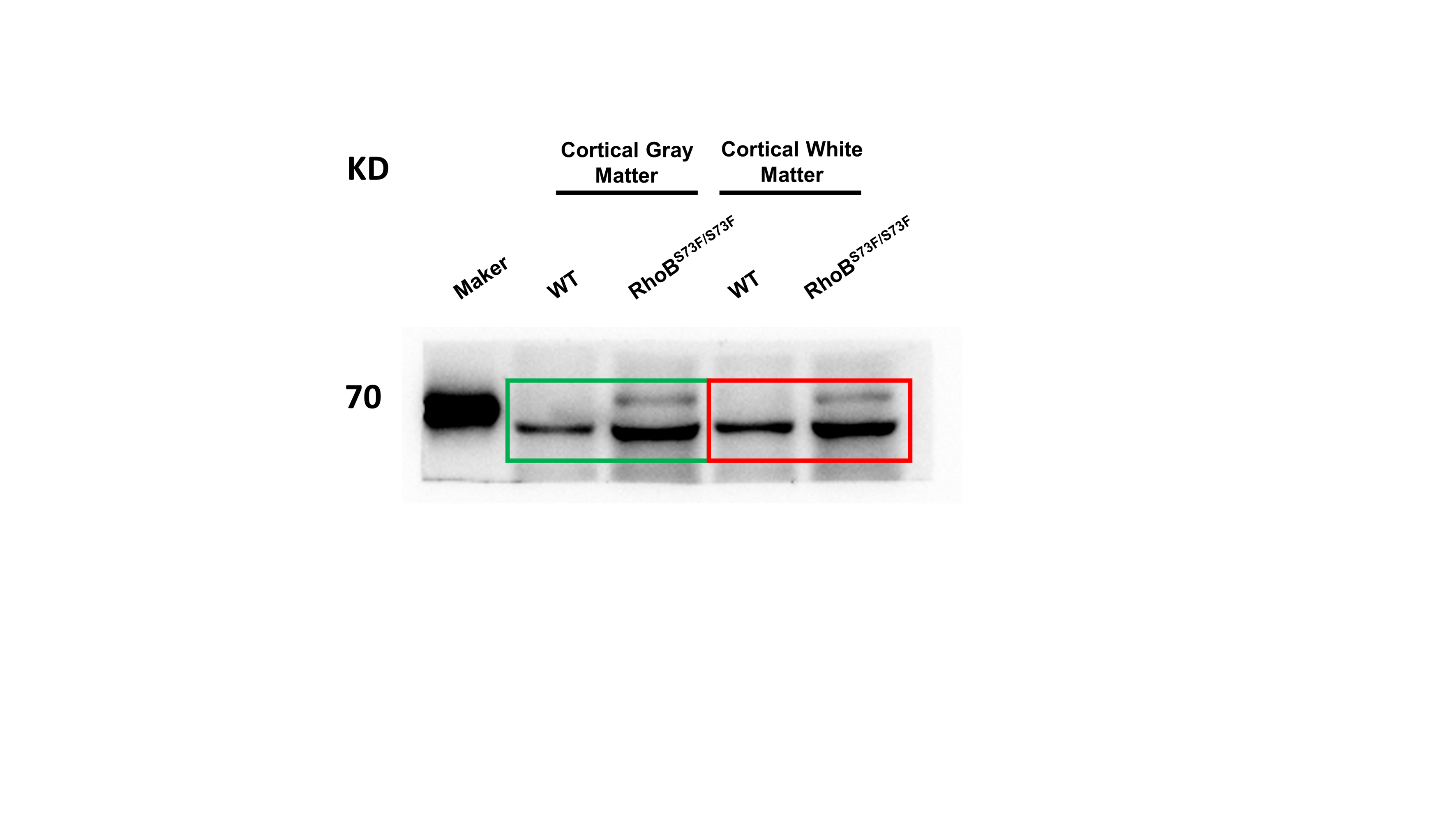

Supplement: Supplementary file 15 — Source data Fig. 5 [file 44321_2024_113_MOESM15_ESM.zip › Figure 5/5E/western LYN in Cortical White Matter.tif]

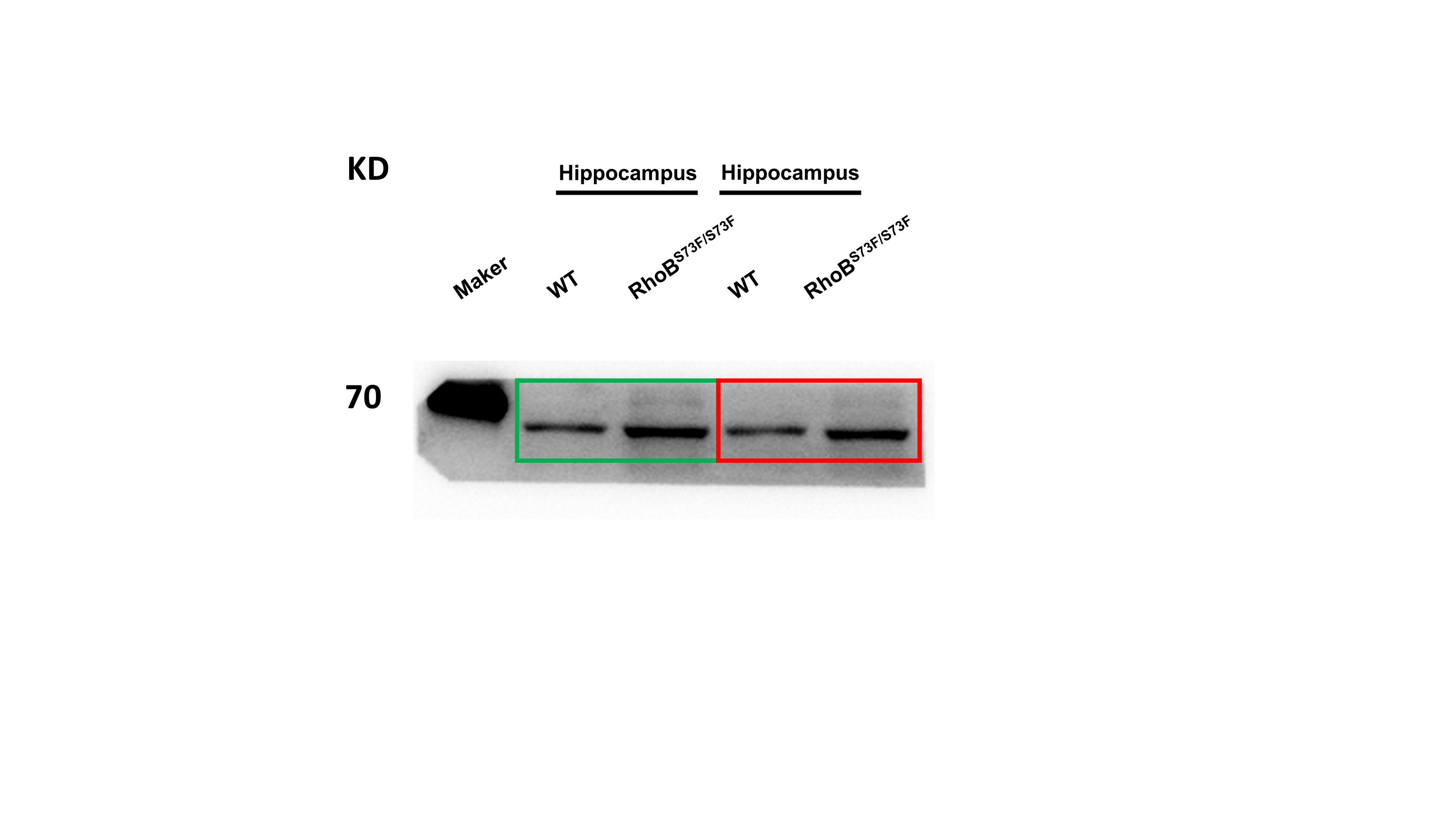

Supplement: Supplementary file 15 — Source data Fig. 5 [file 44321_2024_113_MOESM15_ESM.zip › Figure 5/5E/western LYN in Hippocampus.tif]

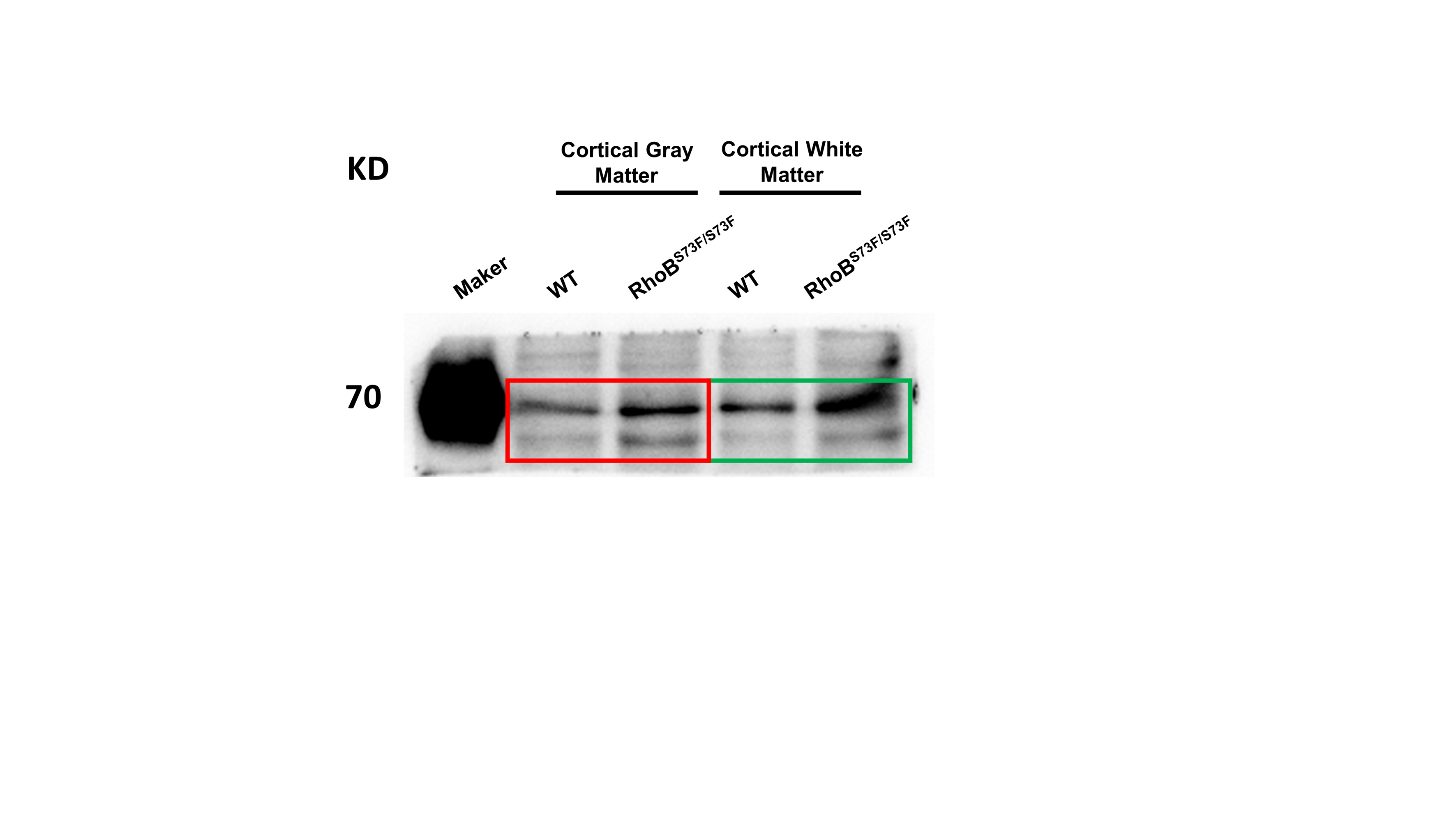

Supplement: Supplementary file 15 — Source data Fig. 5 [file 44321_2024_113_MOESM15_ESM.zip › Figure 5/5E/western P-LYN in Cortical Gray Matter.tif]

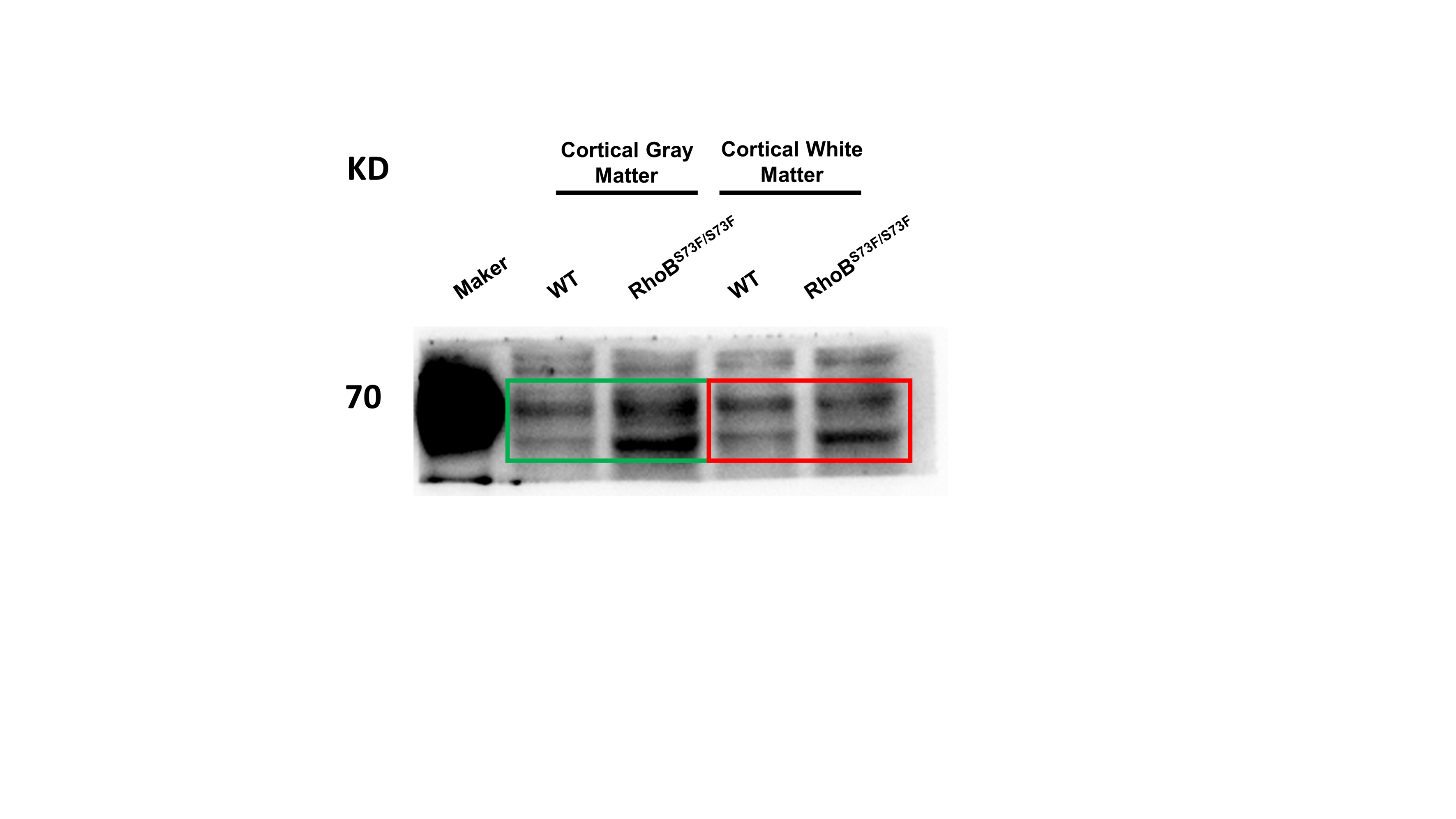

Supplement: Supplementary file 15 — Source data Fig. 5 [file 44321_2024_113_MOESM15_ESM.zip › Figure 5/5E/western P-LYN in Cortical White Matter.tif]

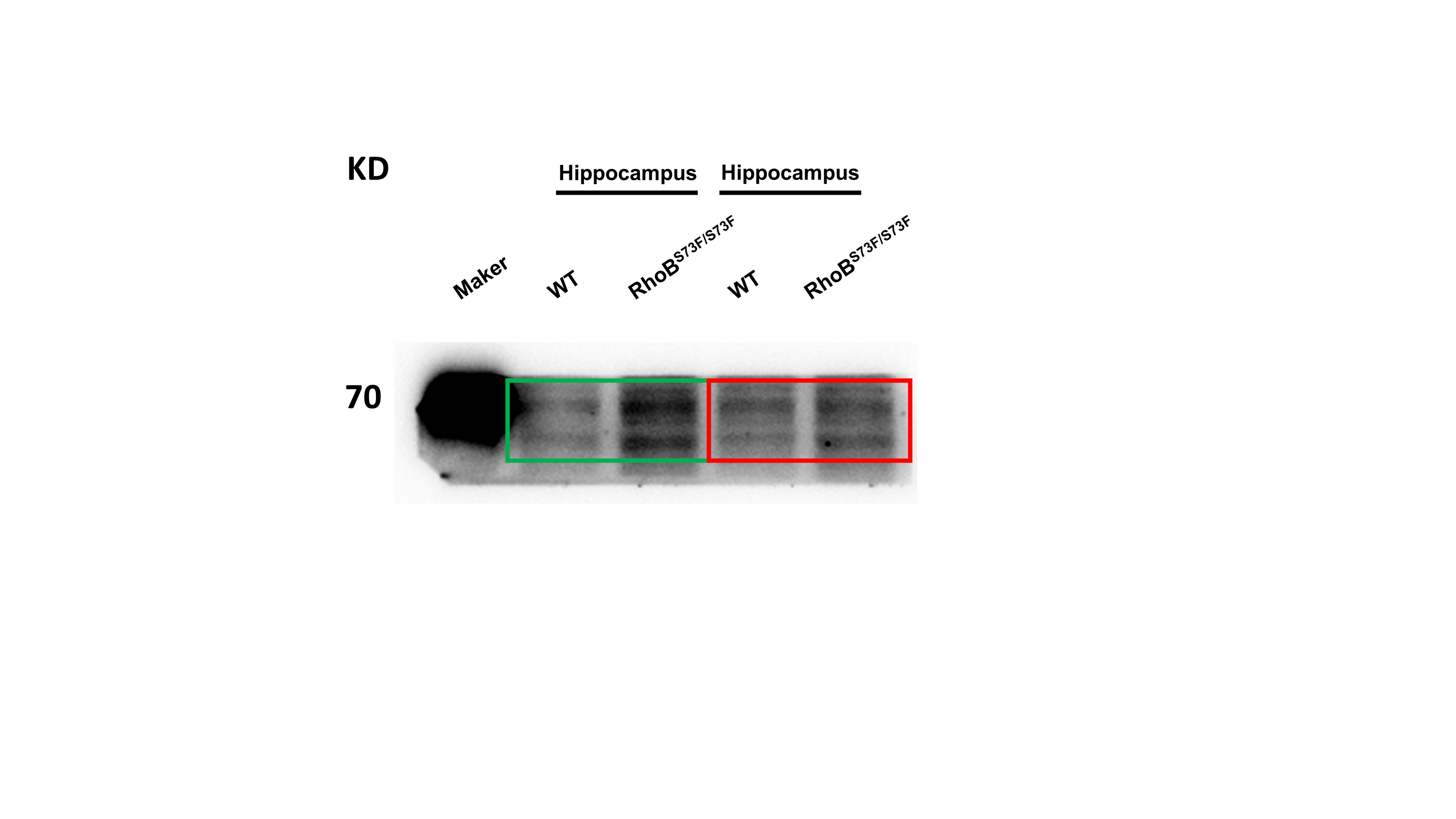

Supplement: Supplementary file 15 — Source data Fig. 5 [file 44321_2024_113_MOESM15_ESM.zip › Figure 5/5E/western P-LYN in Hippocampus.tif]

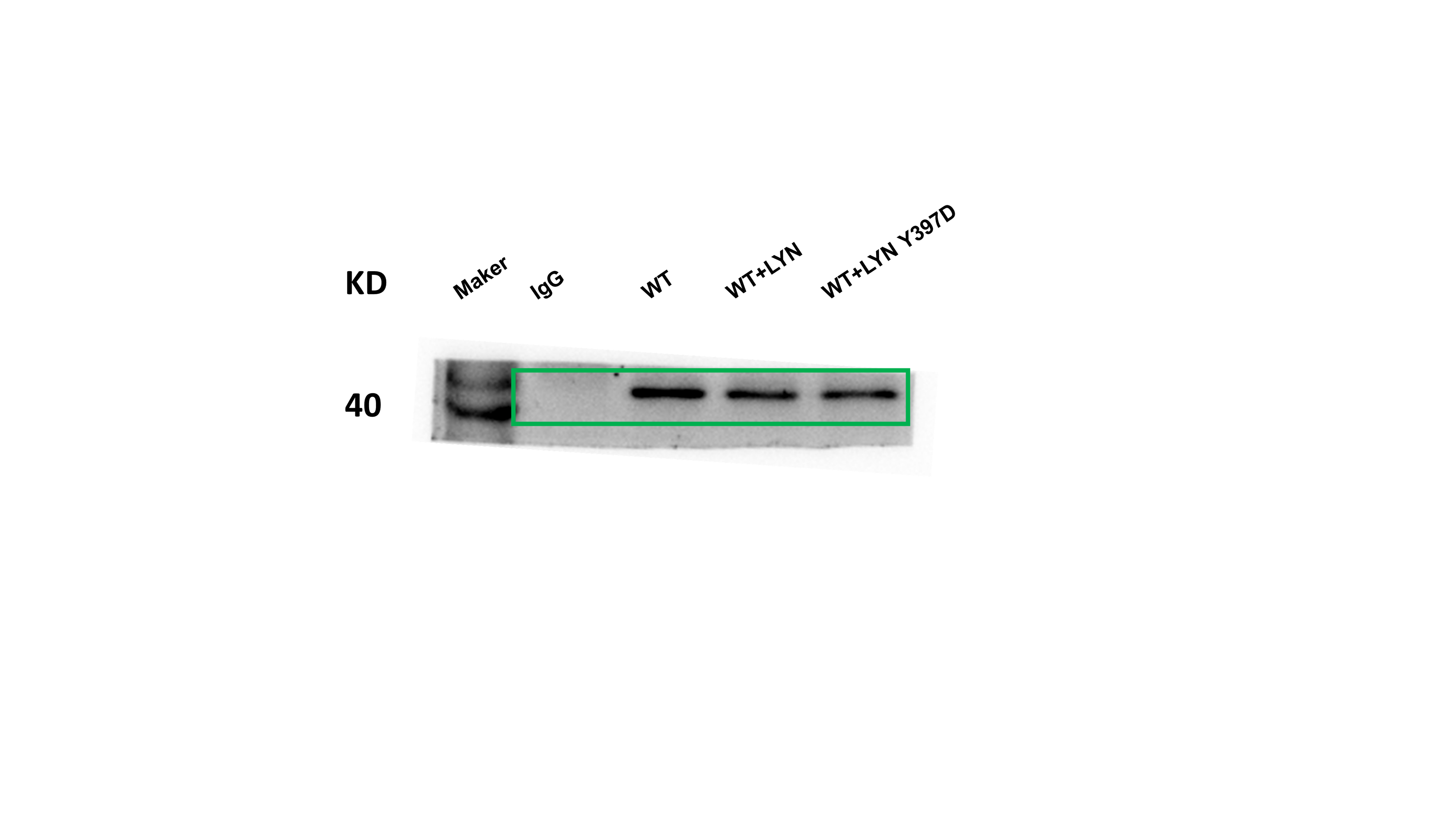

Supplement: Supplementary file 15 — Source data Fig. 5 [file 44321_2024_113_MOESM15_ESM.zip › Figure 5/5F/replicate/western Acat1 replicate (1).tif]

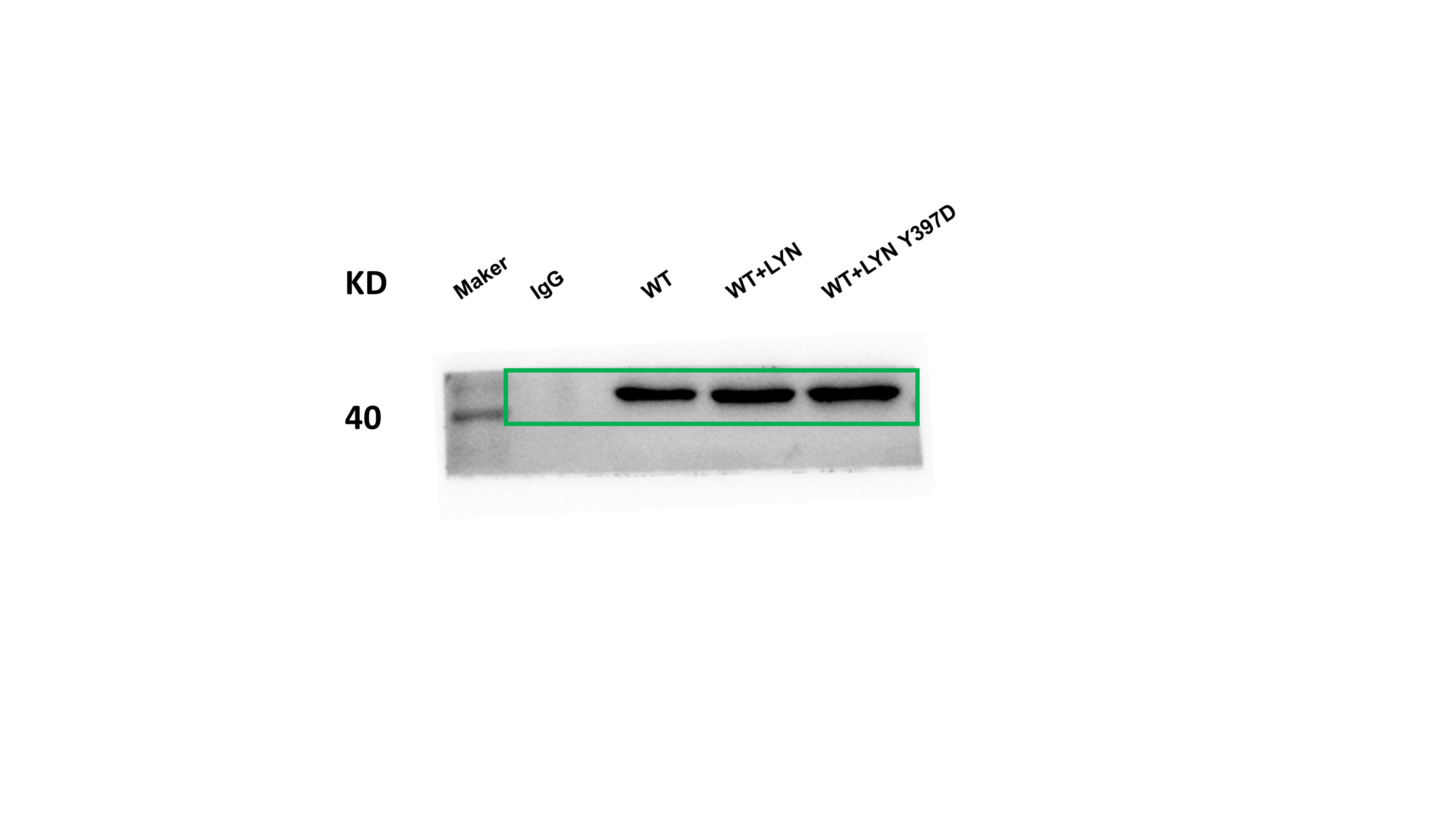

Supplement: Supplementary file 15 — Source data Fig. 5 [file 44321_2024_113_MOESM15_ESM.zip › Figure 5/5F/replicate/western Acat1 replicate (2).tif]

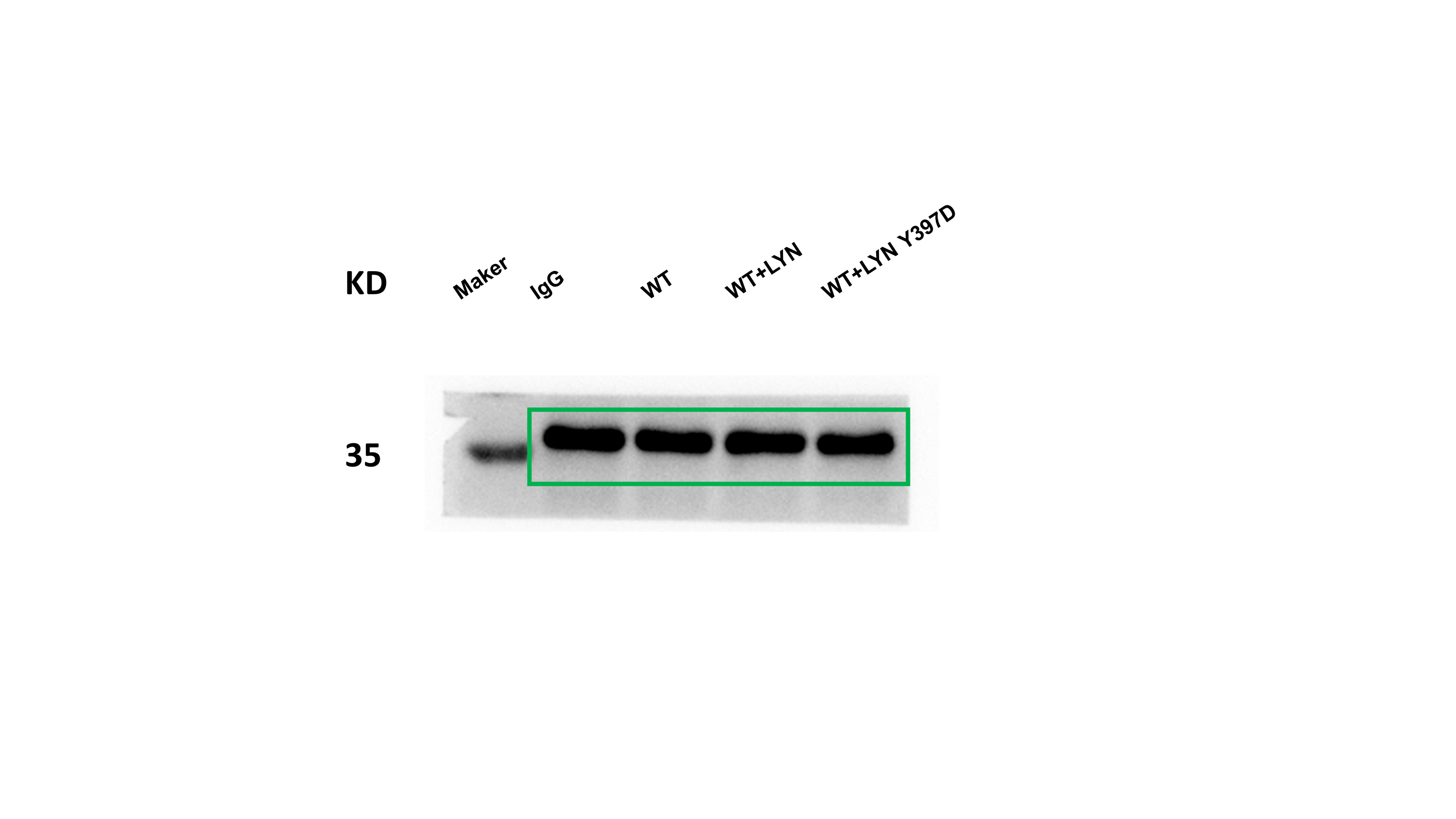

Supplement: Supplementary file 15 — Source data Fig. 5 [file 44321_2024_113_MOESM15_ESM.zip › Figure 5/5F/replicate/western Gapdh replicate (1).tif]

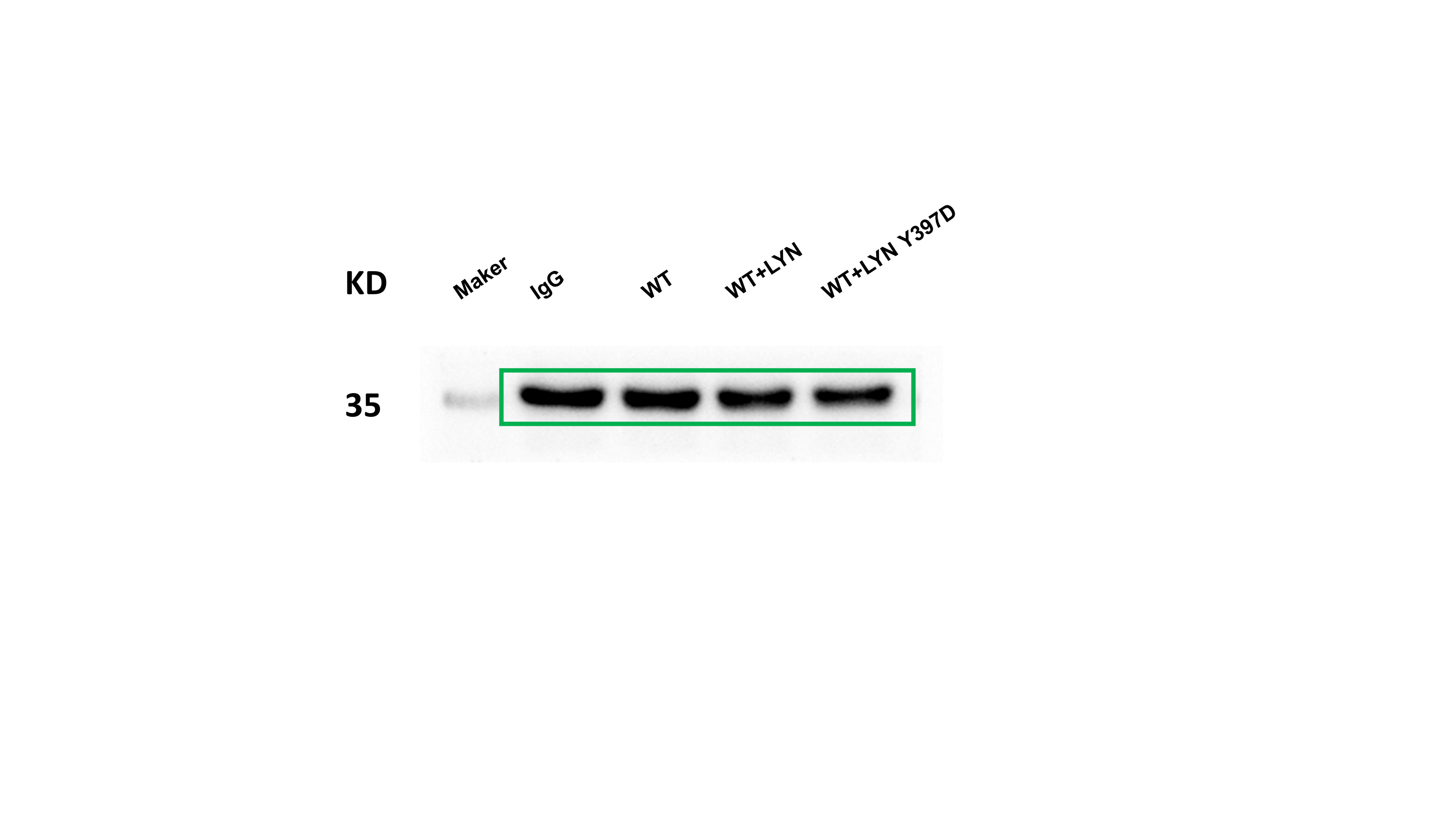

Supplement: Supplementary file 15 — Source data Fig. 5 [file 44321_2024_113_MOESM15_ESM.zip › Figure 5/5F/replicate/western Gapdh replicate (2).tif]

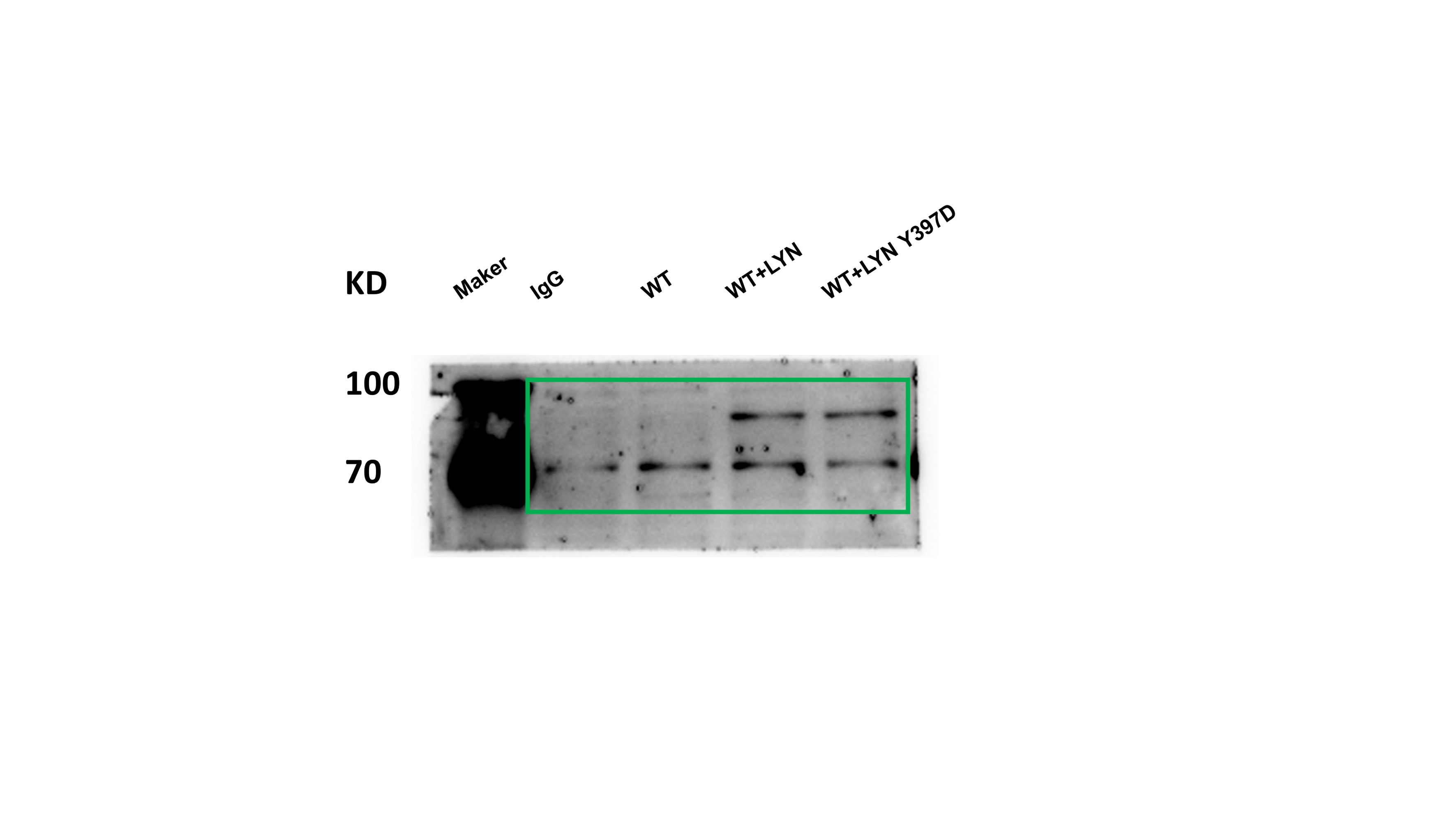

Supplement: Supplementary file 15 — Source data Fig. 5 [file 44321_2024_113_MOESM15_ESM.zip › Figure 5/5F/replicate/western LYN replicate (1).tif]

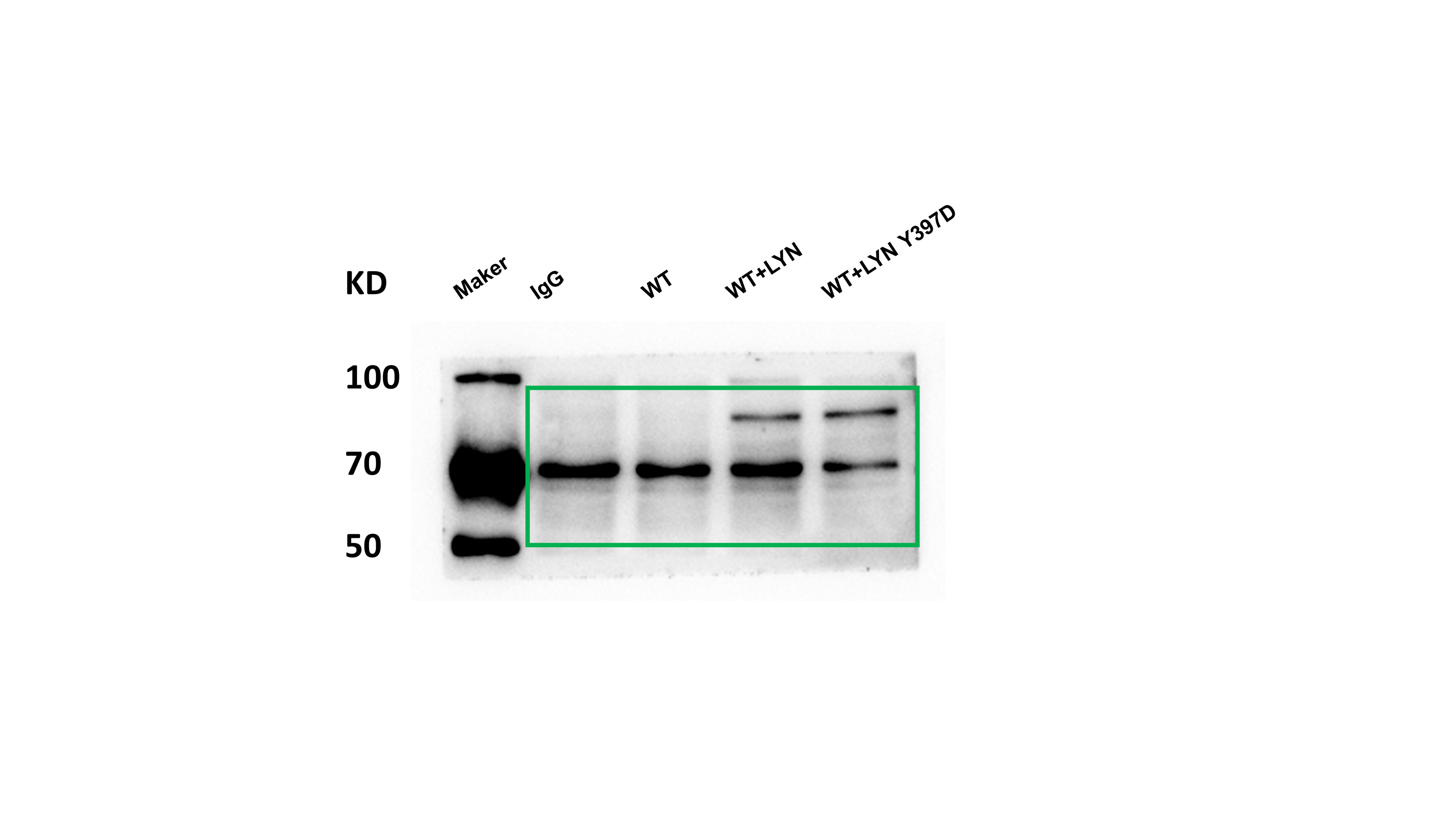

Supplement: Supplementary file 15 — Source data Fig. 5 [file 44321_2024_113_MOESM15_ESM.zip › Figure 5/5F/replicate/western LYN replicate (2).tif]

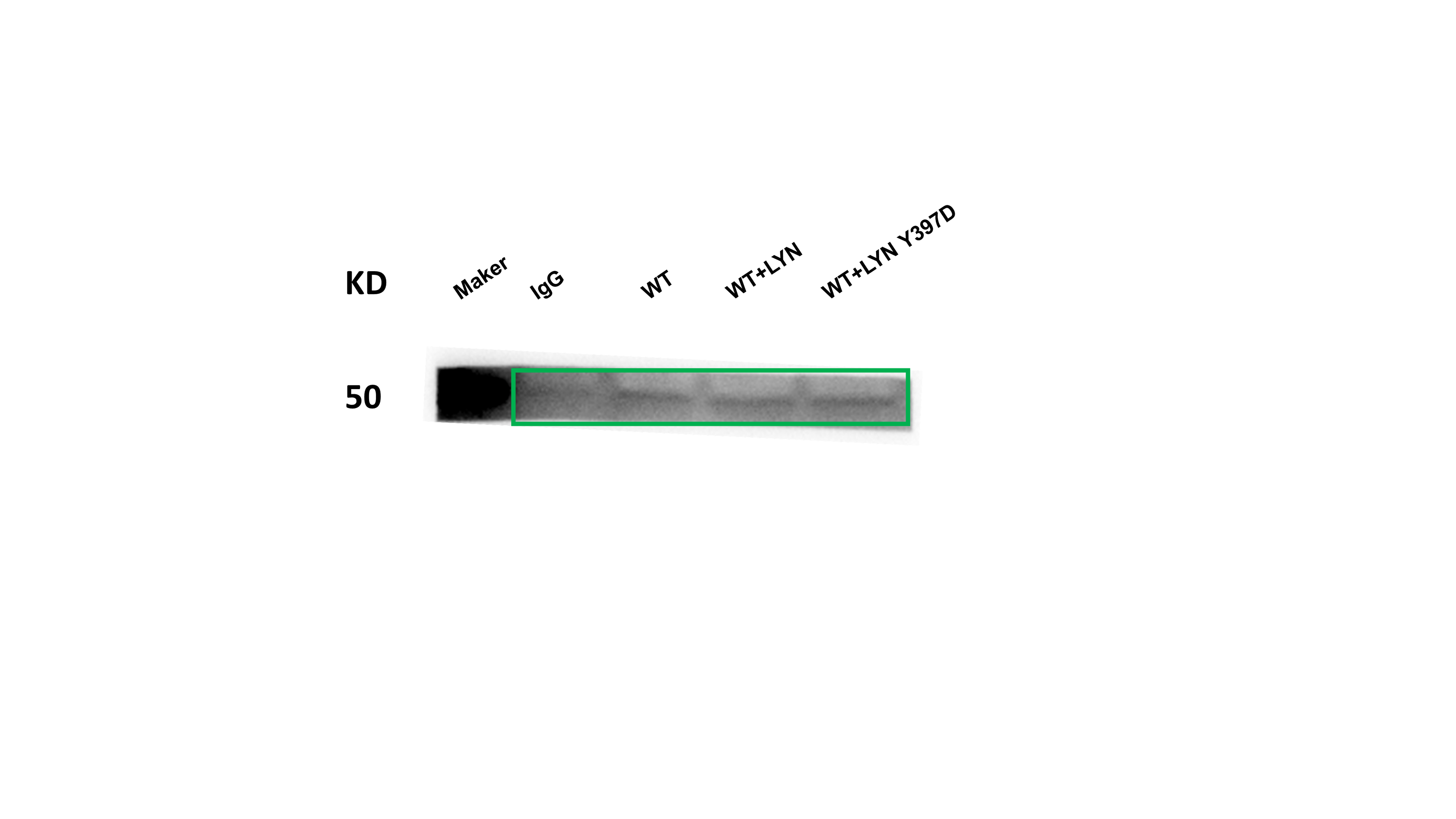

Supplement: Supplementary file 15 — Source data Fig. 5 [file 44321_2024_113_MOESM15_ESM.zip › Figure 5/5F/replicate/western P-Tyr replicate (1).tif]

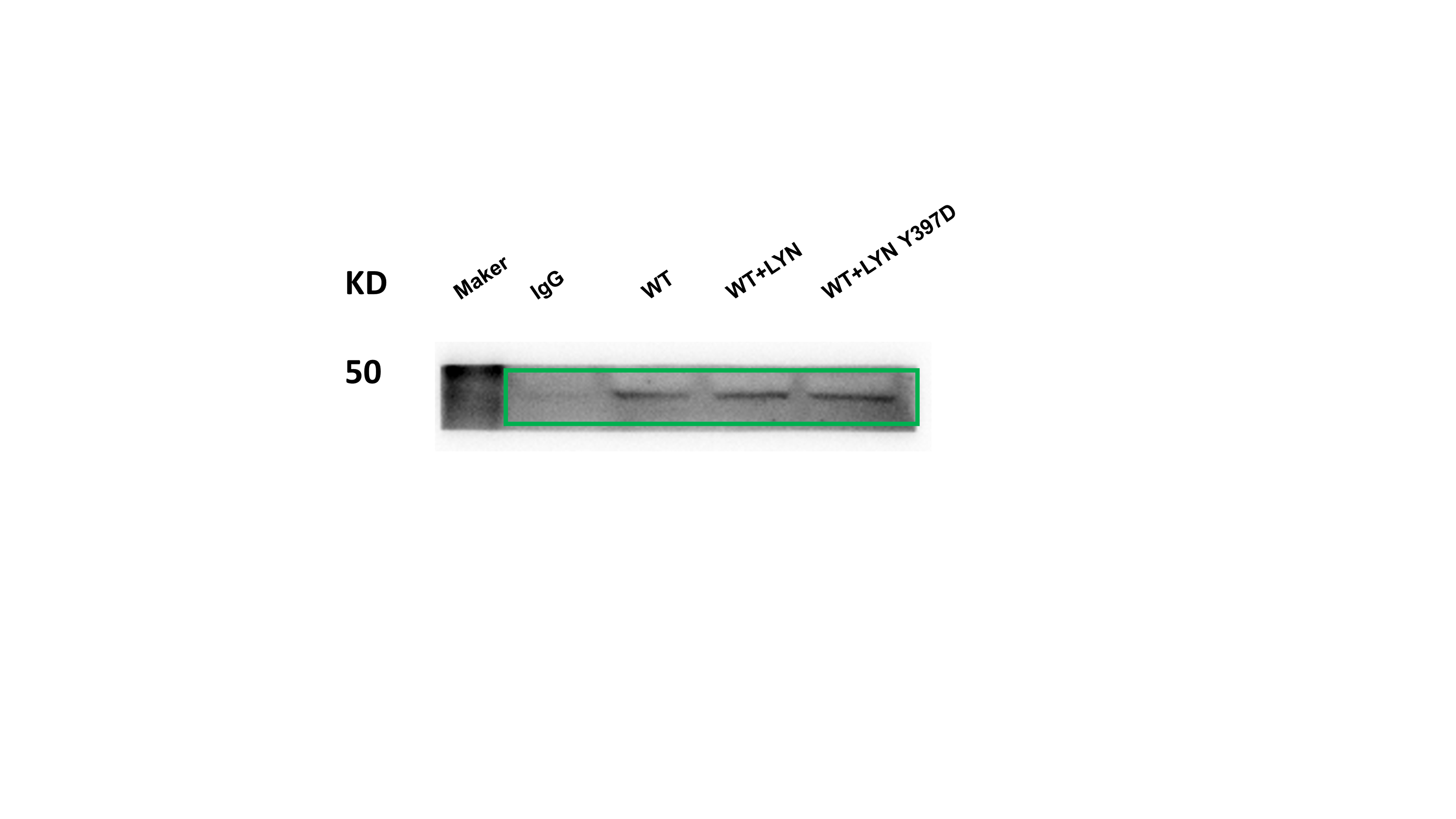

Supplement: Supplementary file 15 — Source data Fig. 5 [file 44321_2024_113_MOESM15_ESM.zip › Figure 5/5F/replicate/western P-Tyr replicate (2).tif]

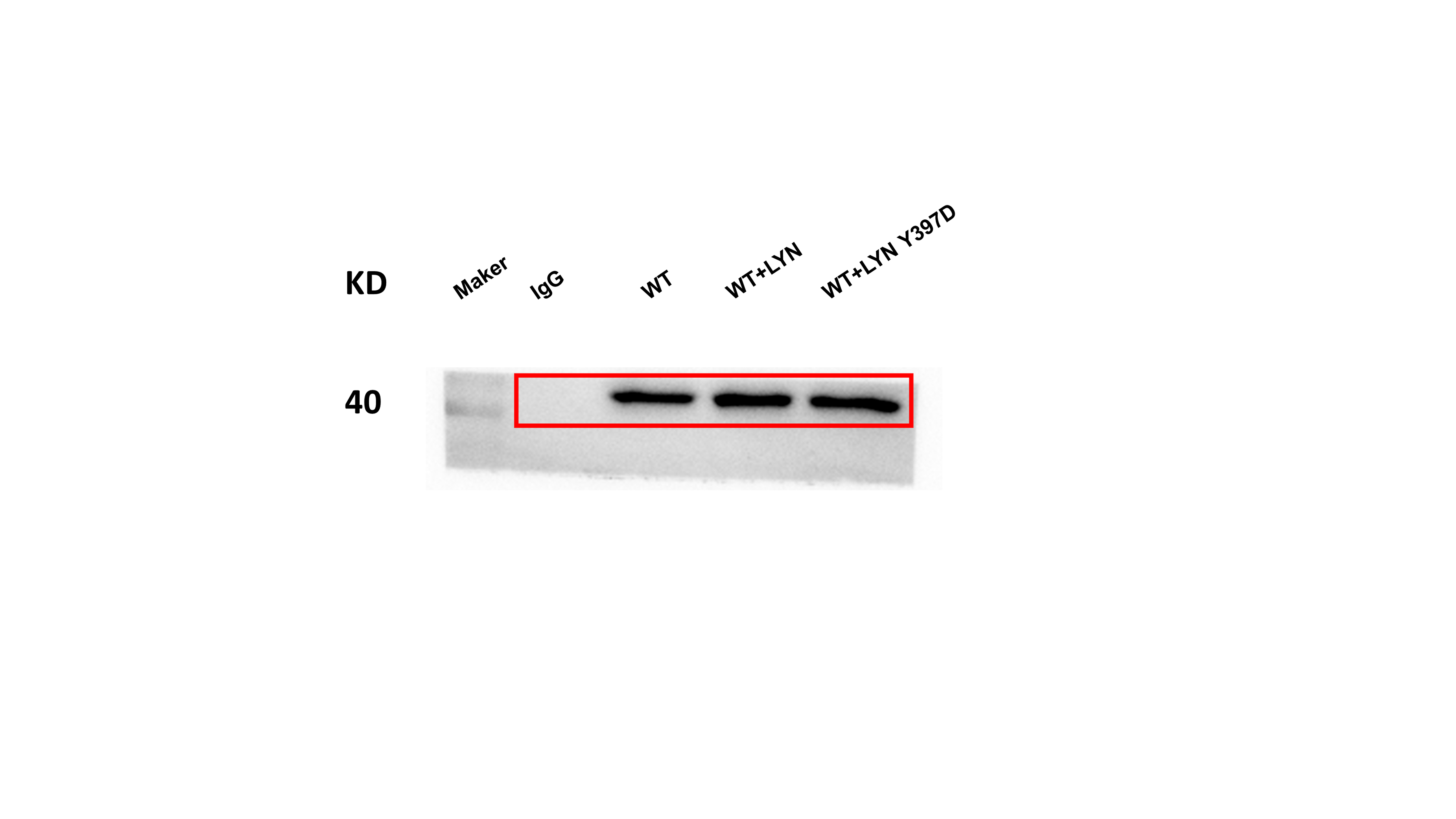

Supplement: Supplementary file 15 — Source data Fig. 5 [file 44321_2024_113_MOESM15_ESM.zip › Figure 5/5F/western Acat1.tif]

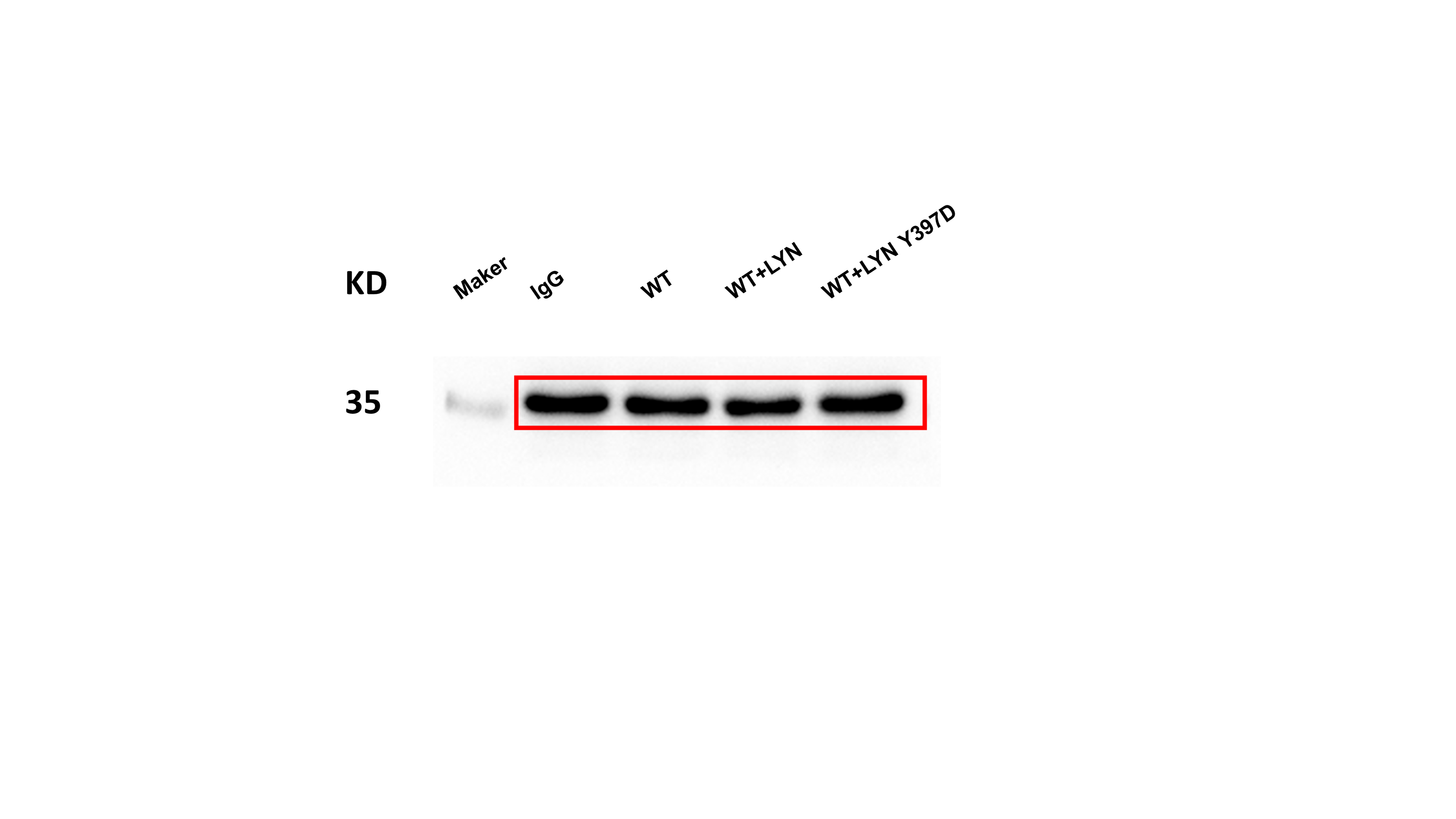

Supplement: Supplementary file 15 — Source data Fig. 5 [file 44321_2024_113_MOESM15_ESM.zip › Figure 5/5F/western Gapdh.tif]

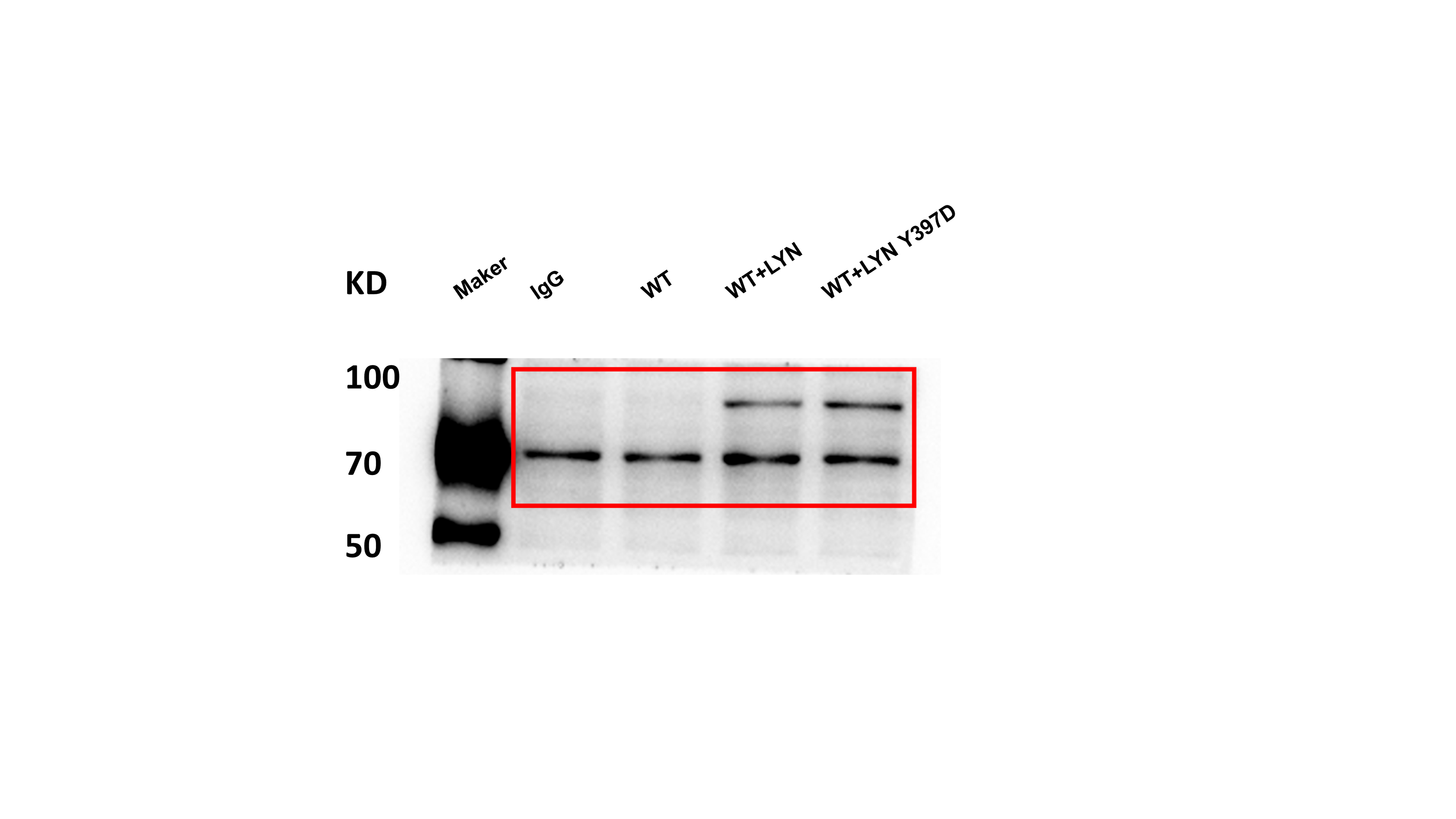

Supplement: Supplementary file 15 — Source data Fig. 5 [file 44321_2024_113_MOESM15_ESM.zip › Figure 5/5F/western LYN.tif]

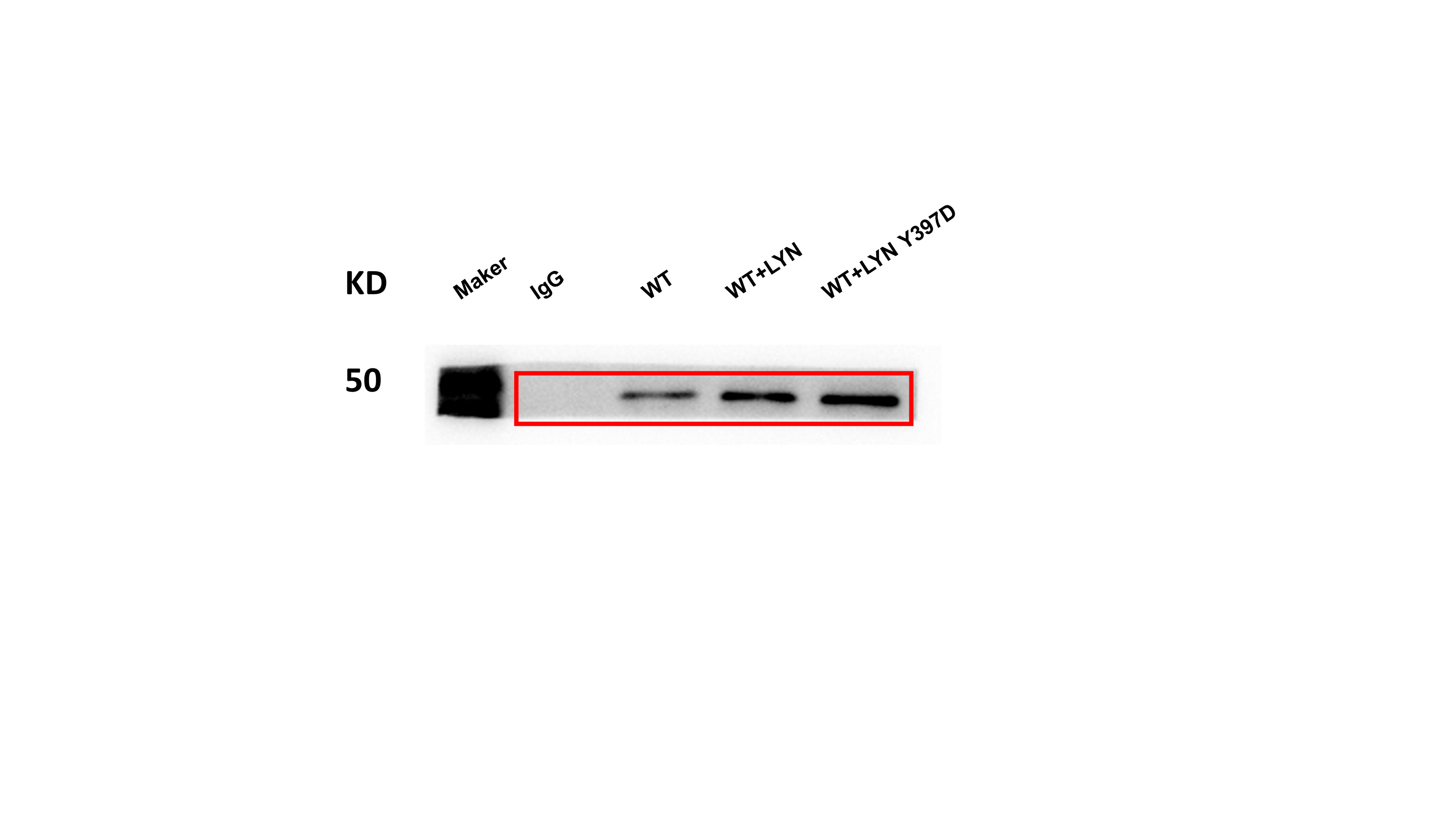

Supplement: Supplementary file 15 — Source data Fig. 5 [file 44321_2024_113_MOESM15_ESM.zip › Figure 5/5F/western P-Tyr.tif]

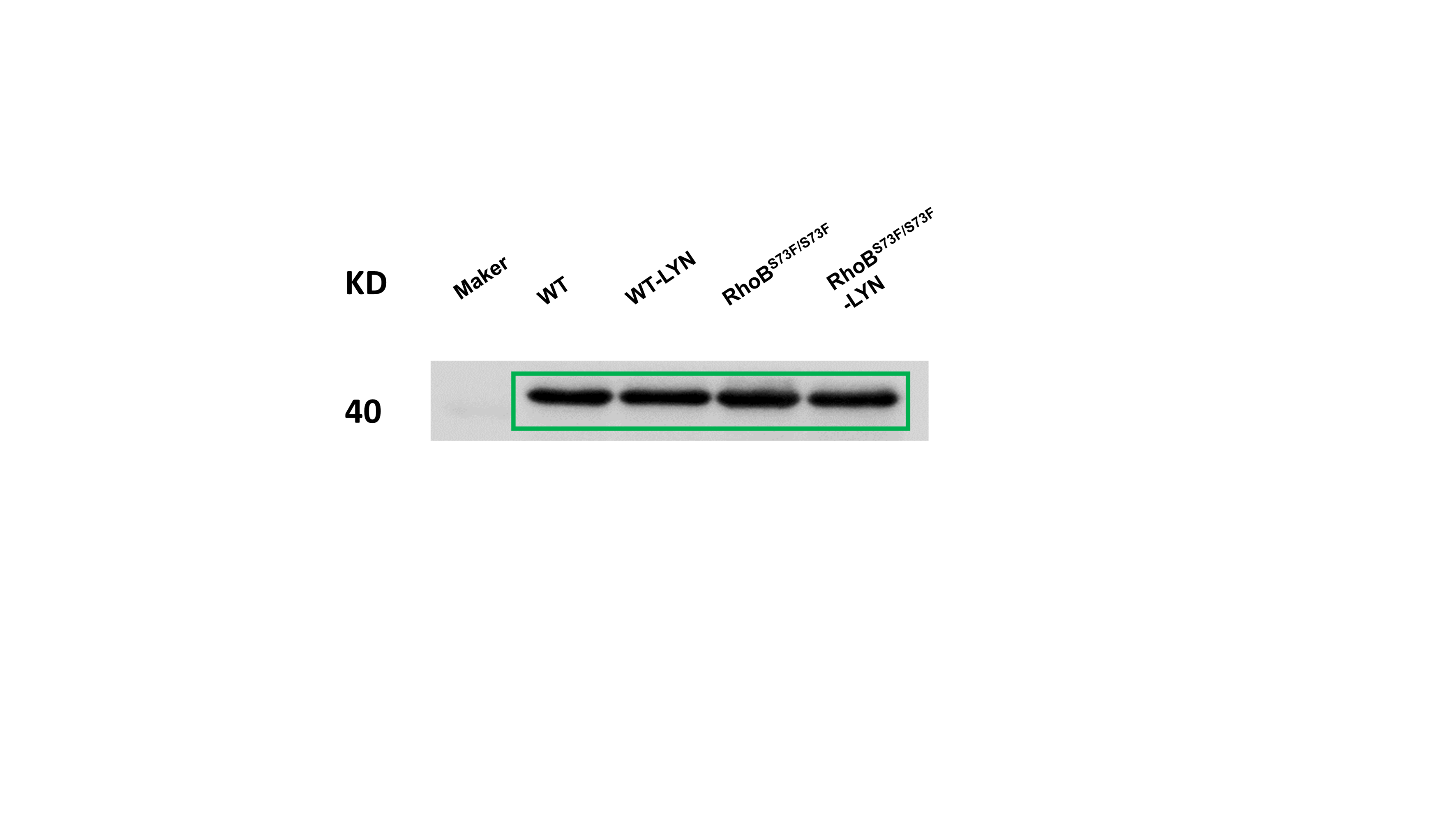

Supplement: Supplementary file 15 — Source data Fig. 5 [file 44321_2024_113_MOESM15_ESM.zip › Figure 5/5G/replicate/western Acat1 replicate (1).tif]

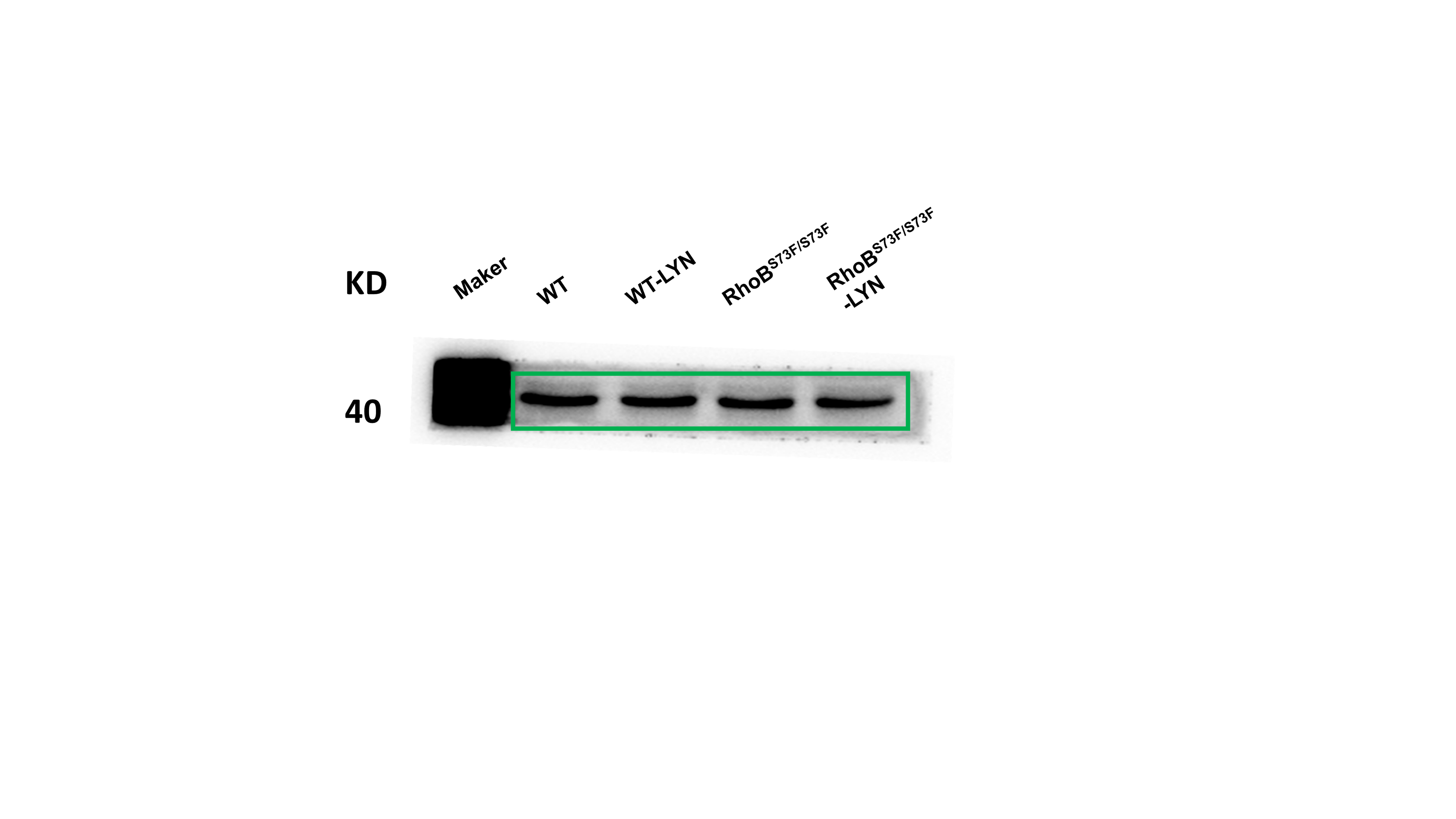

Supplement: Supplementary file 15 — Source data Fig. 5 [file 44321_2024_113_MOESM15_ESM.zip › Figure 5/5G/replicate/western Acat1 replicate (2).tif]

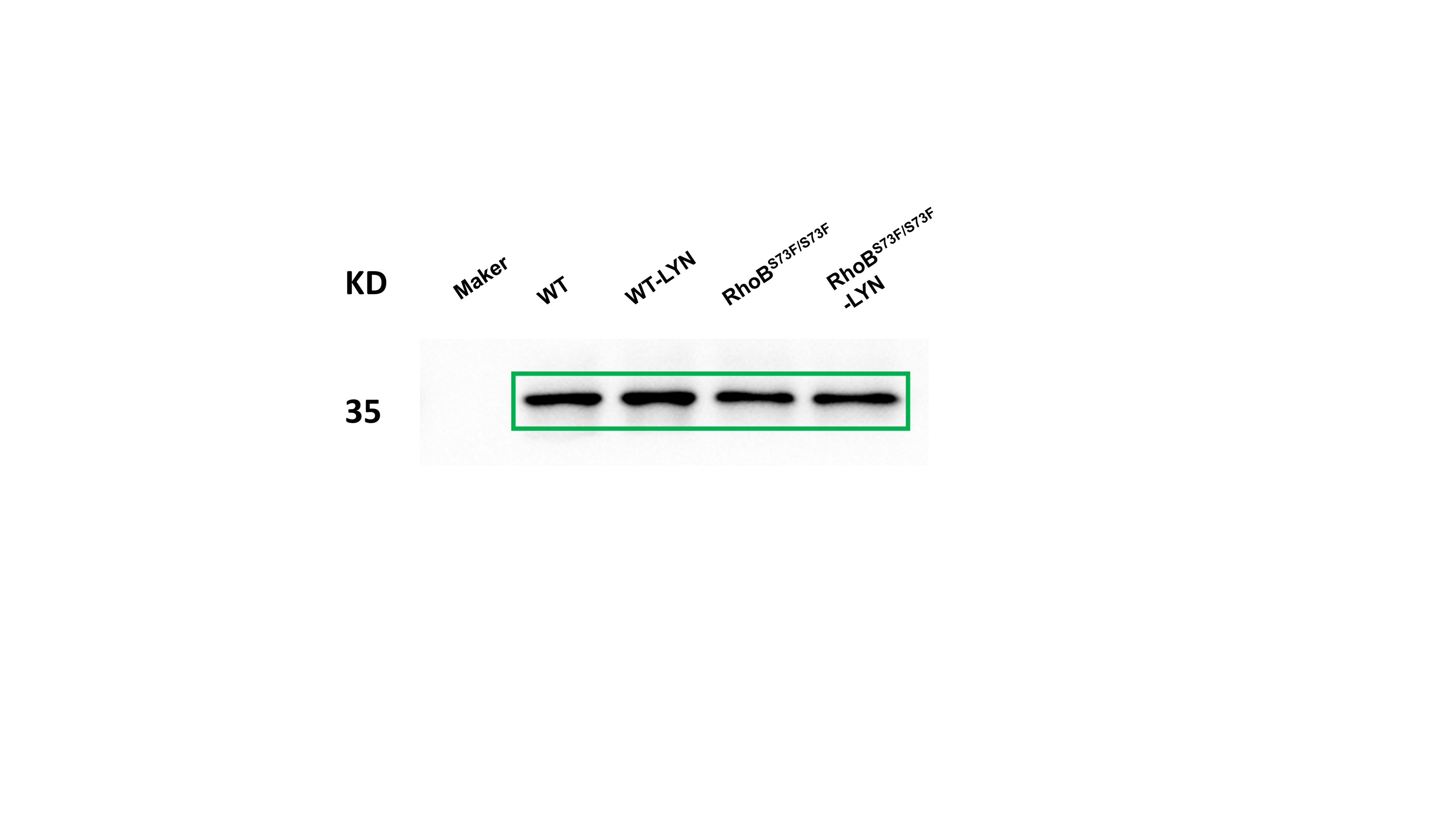

Supplement: Supplementary file 15 — Source data Fig. 5 [file 44321_2024_113_MOESM15_ESM.zip › Figure 5/5G/replicate/western Gapdh replicate (1).tif]

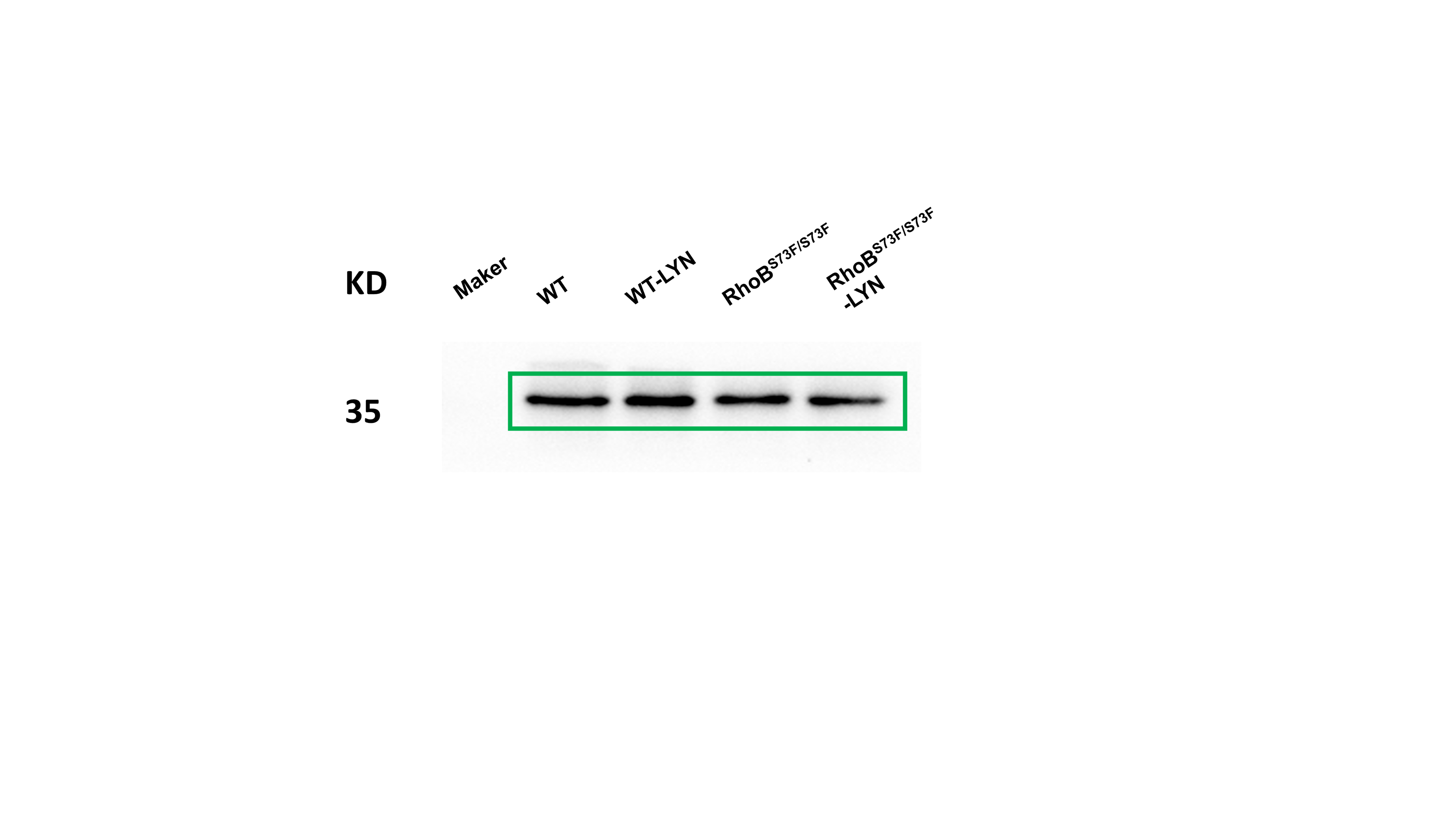

Supplement: Supplementary file 15 — Source data Fig. 5 [file 44321_2024_113_MOESM15_ESM.zip › Figure 5/5G/replicate/western Gapdh replicate (2).tif]

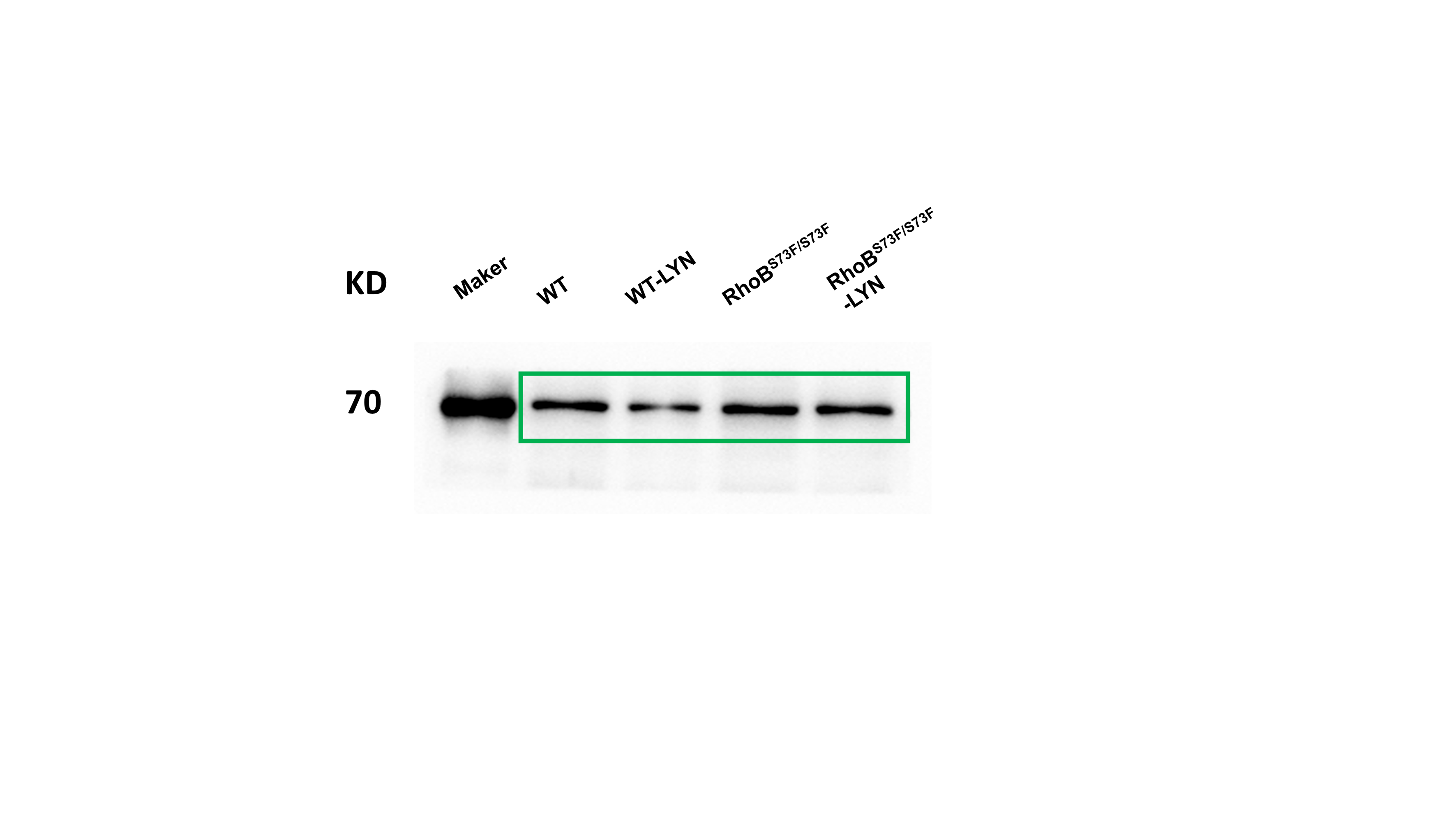

Supplement: Supplementary file 15 — Source data Fig. 5 [file 44321_2024_113_MOESM15_ESM.zip › Figure 5/5G/replicate/western LYN replicate (1).tif]

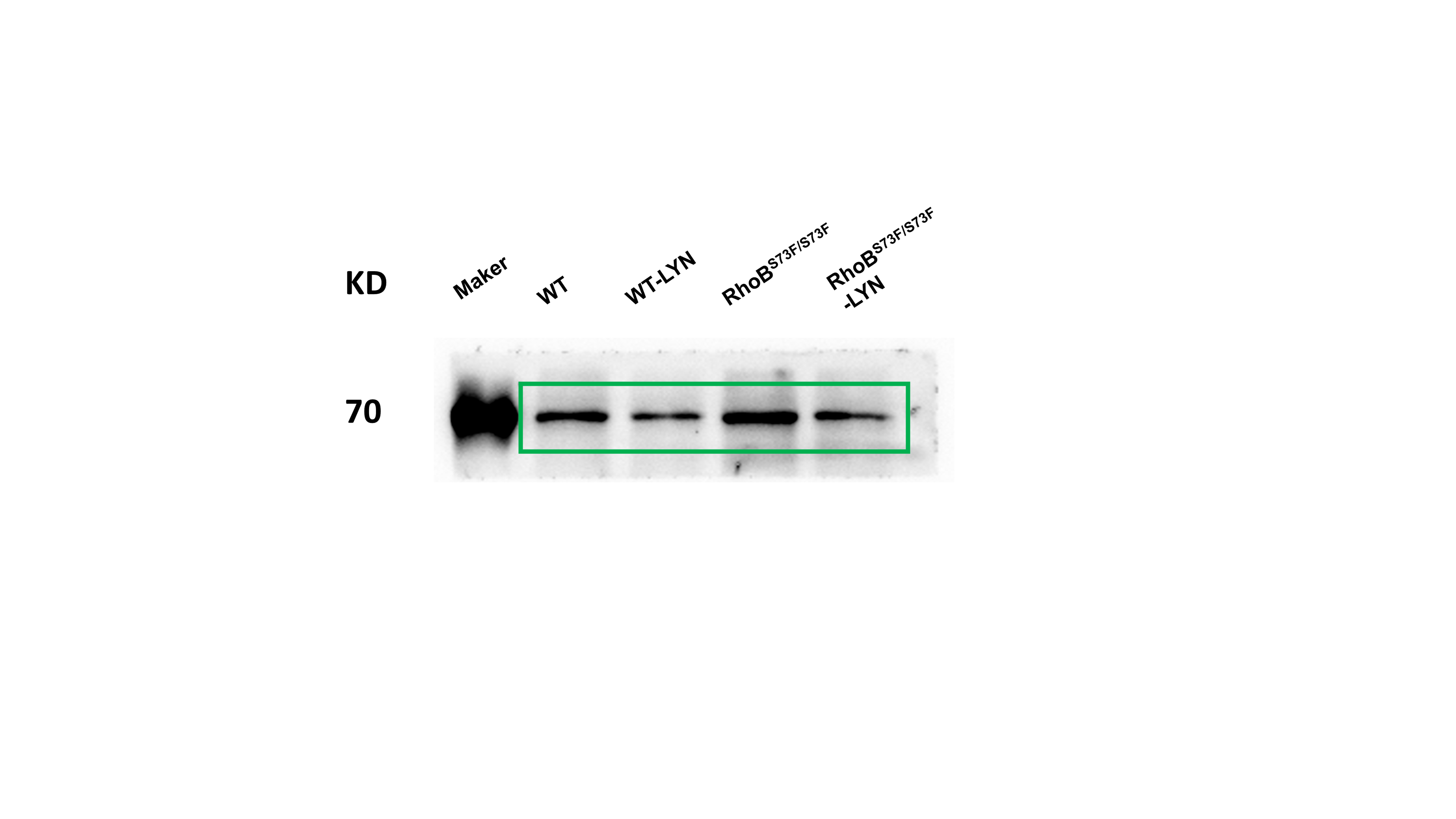

Supplement: Supplementary file 15 — Source data Fig. 5 [file 44321_2024_113_MOESM15_ESM.zip › Figure 5/5G/replicate/western LYN replicate (2).tif]

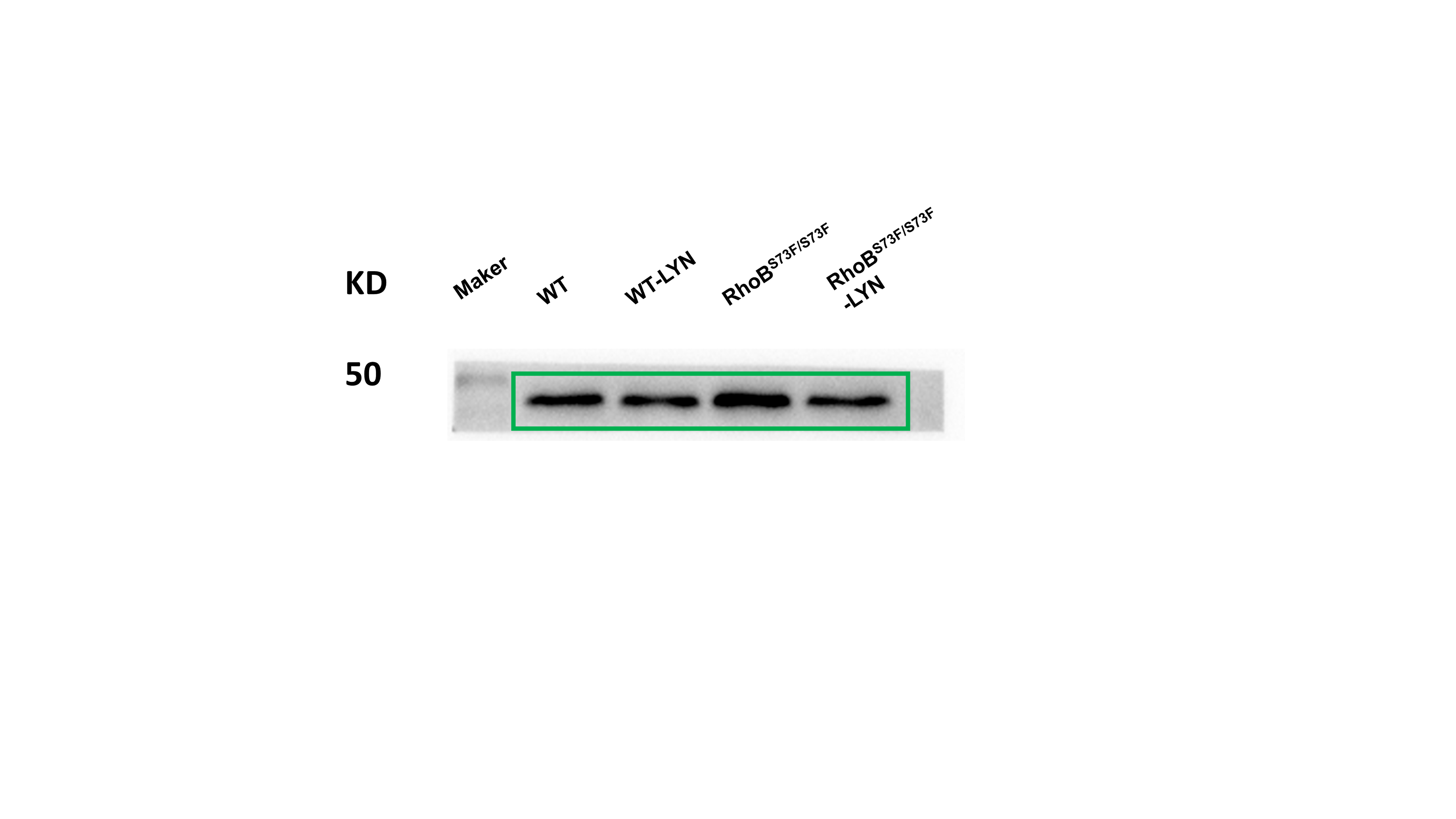

Supplement: Supplementary file 15 — Source data Fig. 5 [file 44321_2024_113_MOESM15_ESM.zip › Figure 5/5G/replicate/western P-Tyr replicate (1).tif]

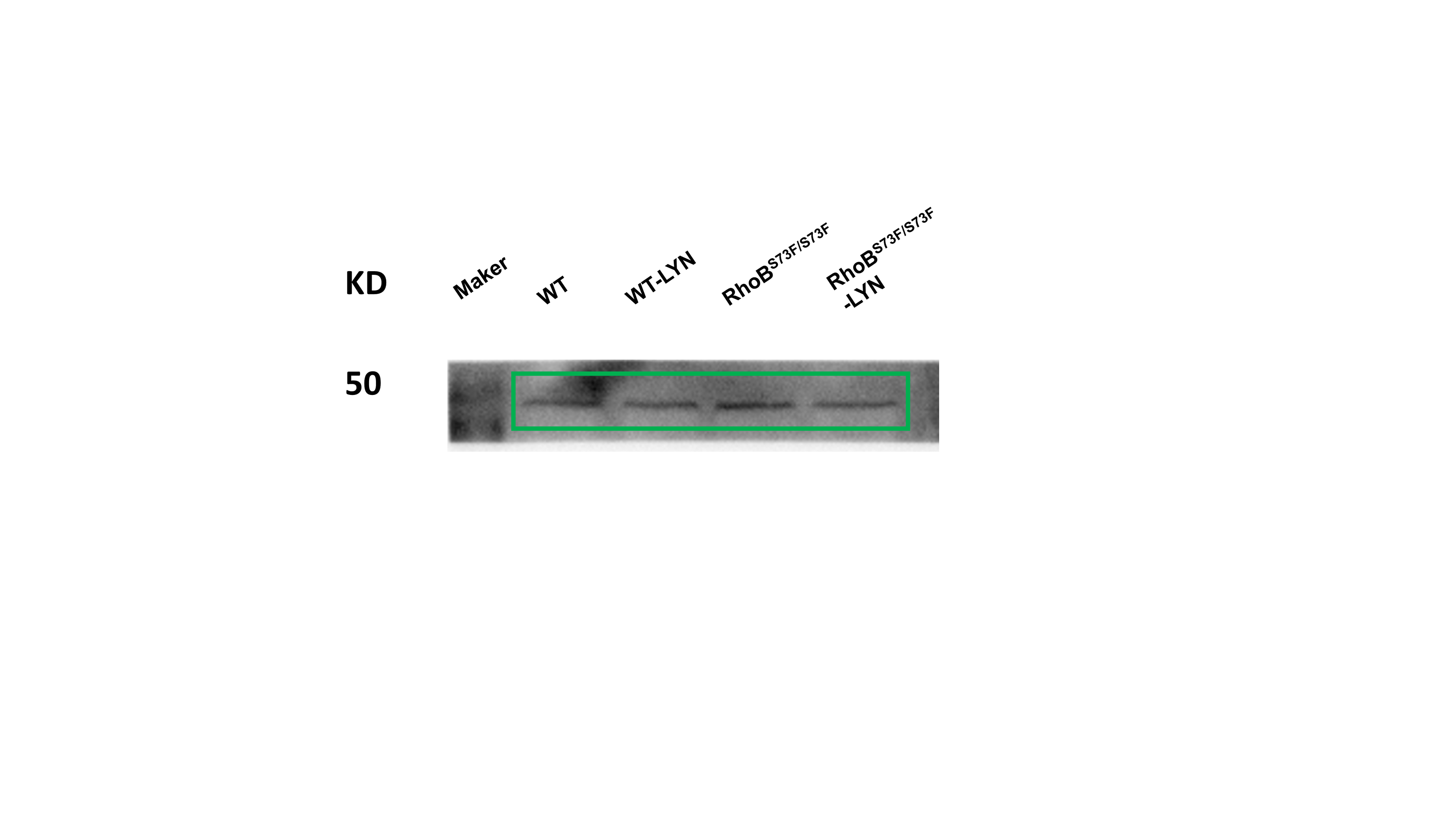

Supplement: Supplementary file 15 — Source data Fig. 5 [file 44321_2024_113_MOESM15_ESM.zip › Figure 5/5G/replicate/western P-Tyr replicate (2).tif]

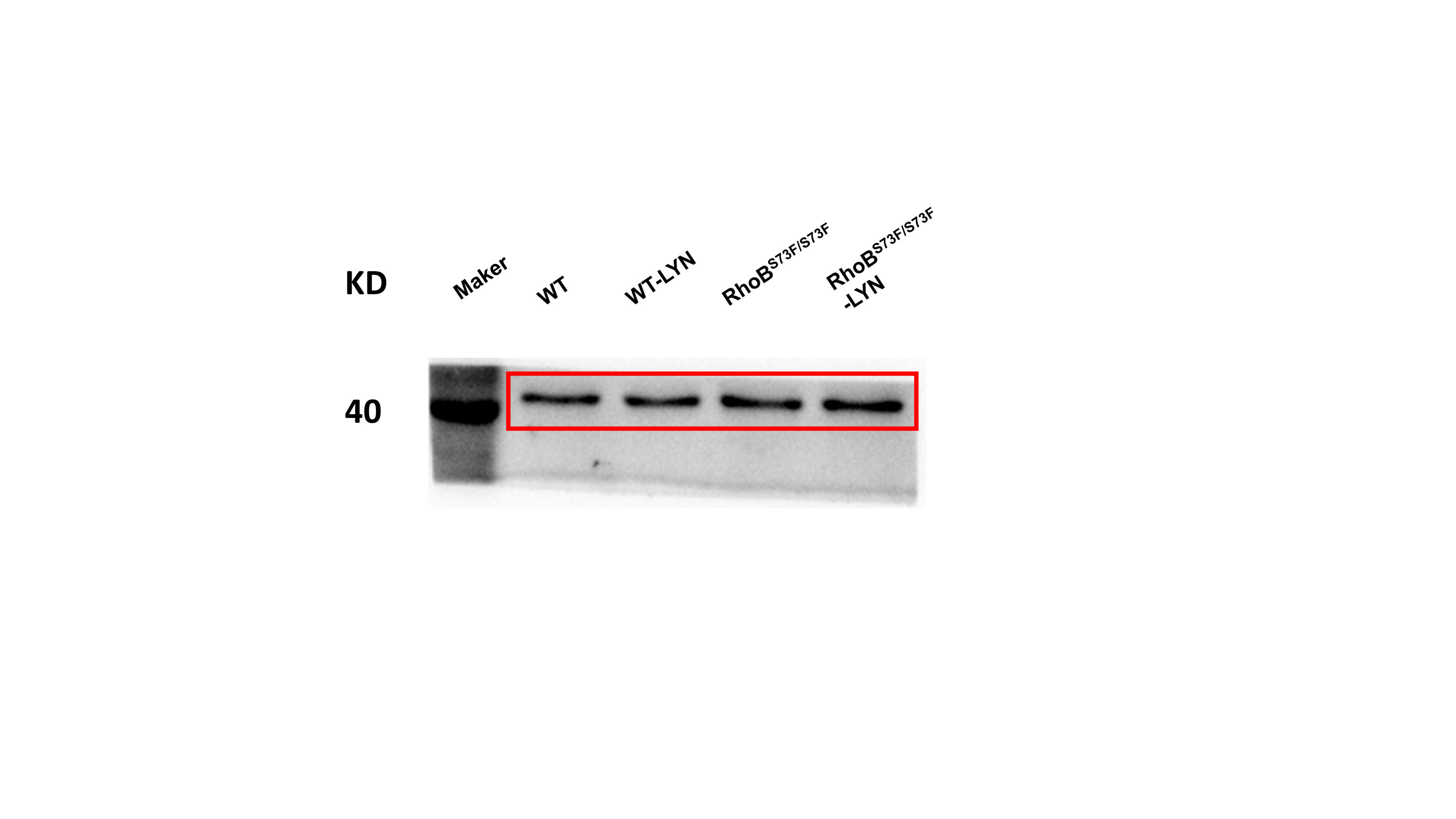

Supplement: Supplementary file 15 — Source data Fig. 5 [file 44321_2024_113_MOESM15_ESM.zip › Figure 5/5G/western Acat1.tif]

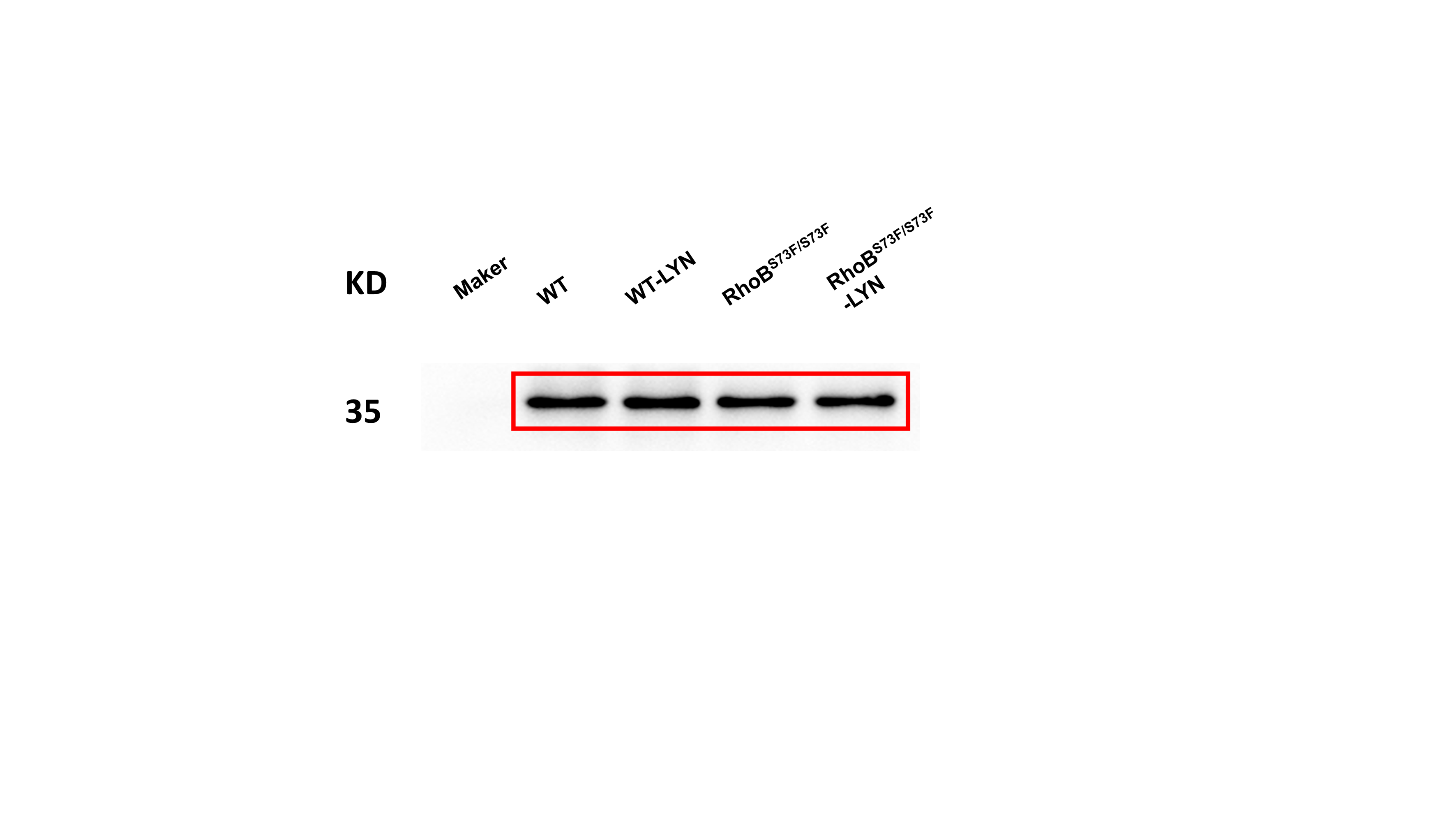

Supplement: Supplementary file 15 — Source data Fig. 5 [file 44321_2024_113_MOESM15_ESM.zip › Figure 5/5G/western Gapdh.tif]

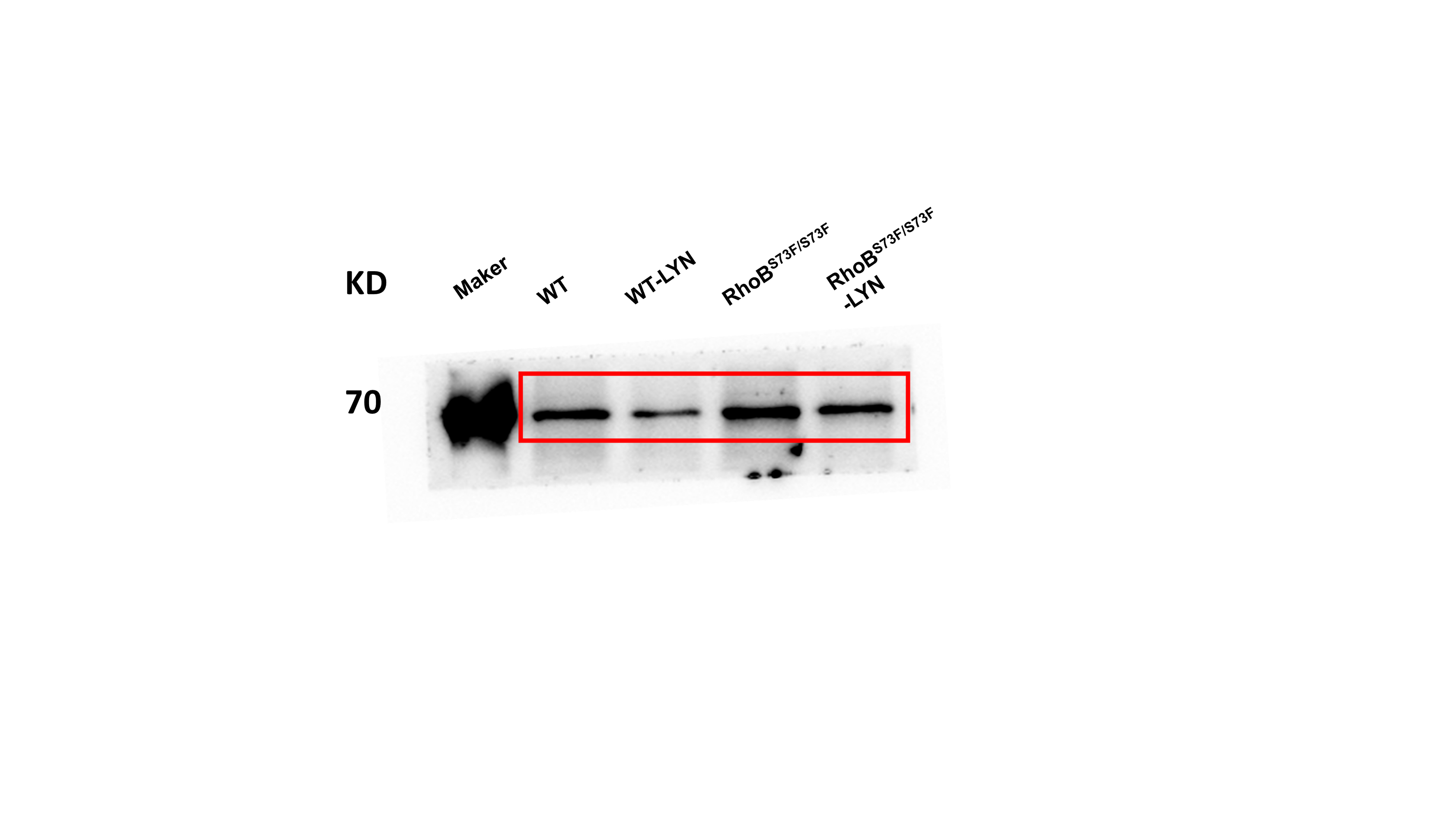

Supplement: Supplementary file 15 — Source data Fig. 5 [file 44321_2024_113_MOESM15_ESM.zip › Figure 5/5G/western LYN.tif]

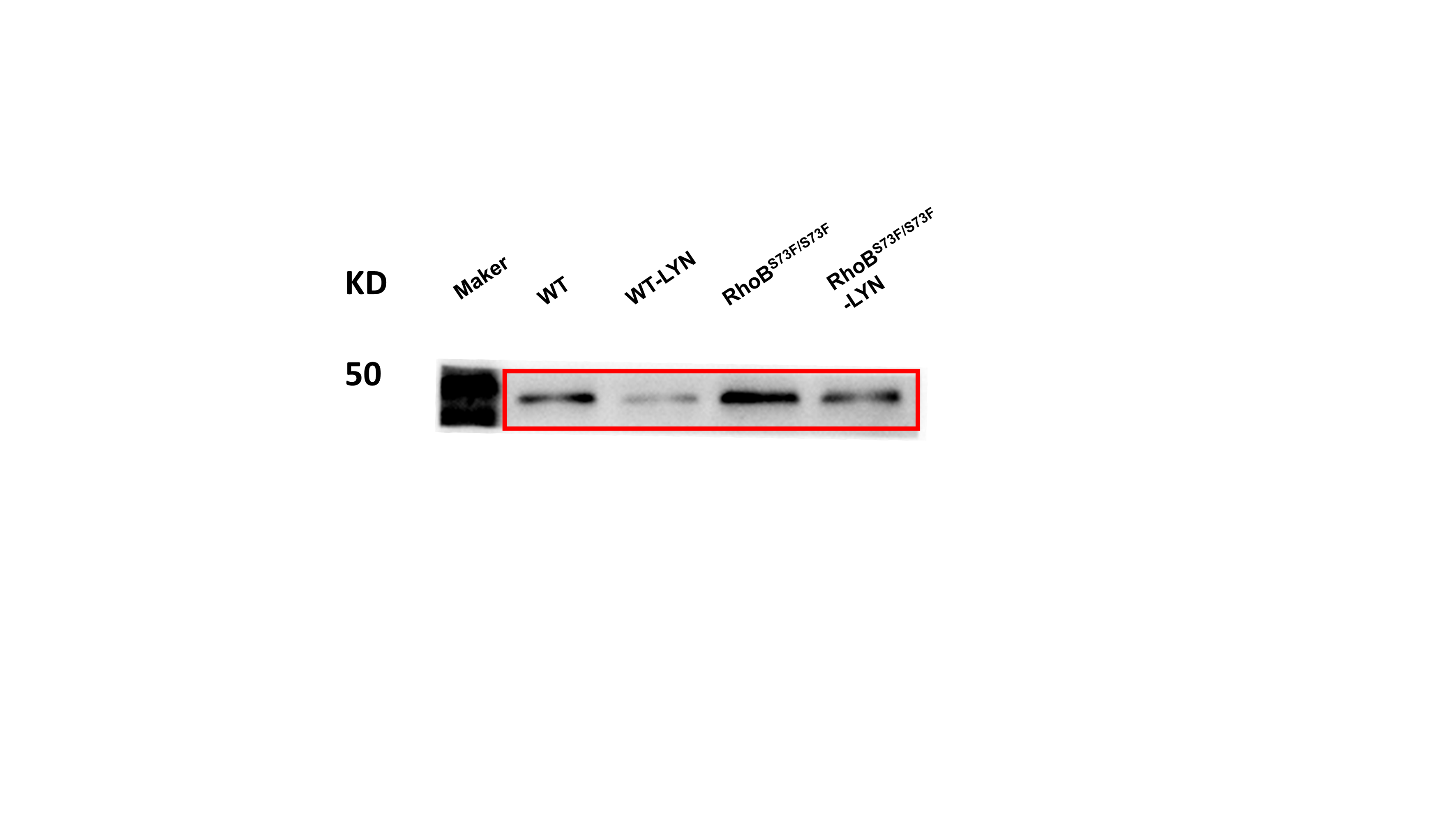

Supplement: Supplementary file 15 — Source data Fig. 5 [file 44321_2024_113_MOESM15_ESM.zip › Figure 5/5G/western P-Tyr.tif]

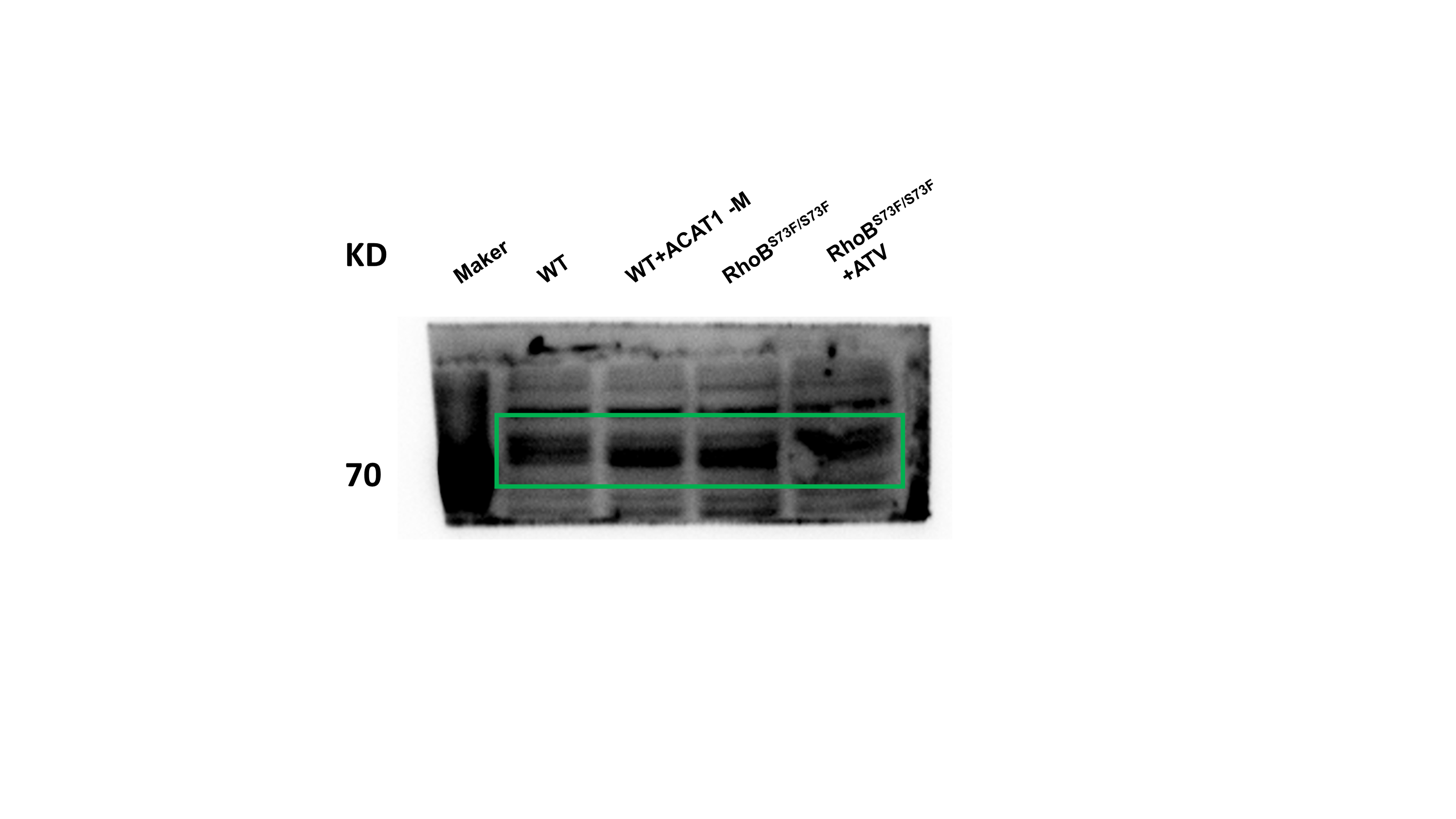

Supplement: Supplementary file 16 — Source data Fig. 6 [file 44321_2024_113_MOESM16_ESM.zip › Figure 6/6F/replicate/western Calpain1 replicate (1).tif]

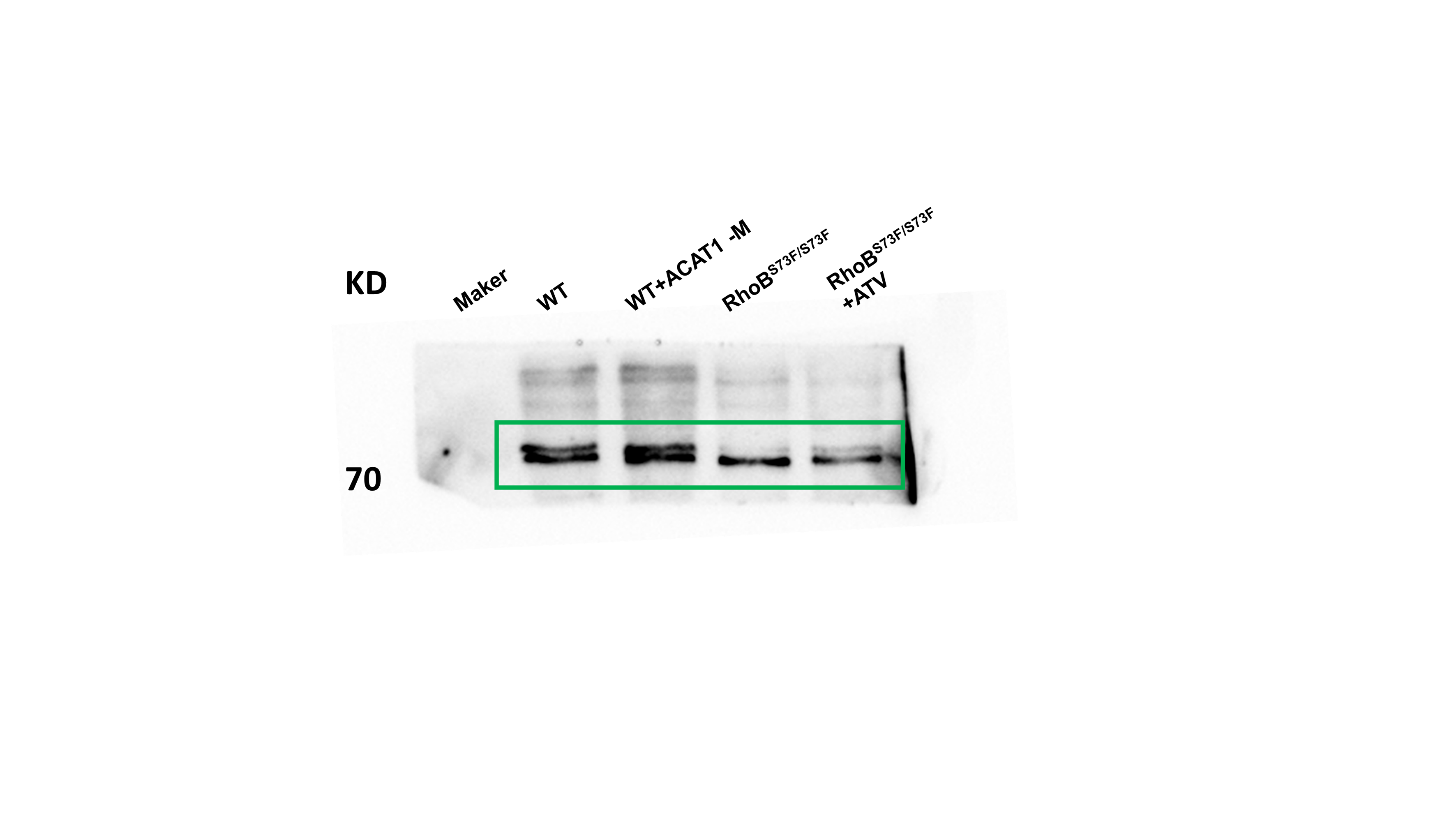

Supplement: Supplementary file 16 — Source data Fig. 6 [file 44321_2024_113_MOESM16_ESM.zip › Figure 6/6F/replicate/western Calpain1 replicate (2).tif]

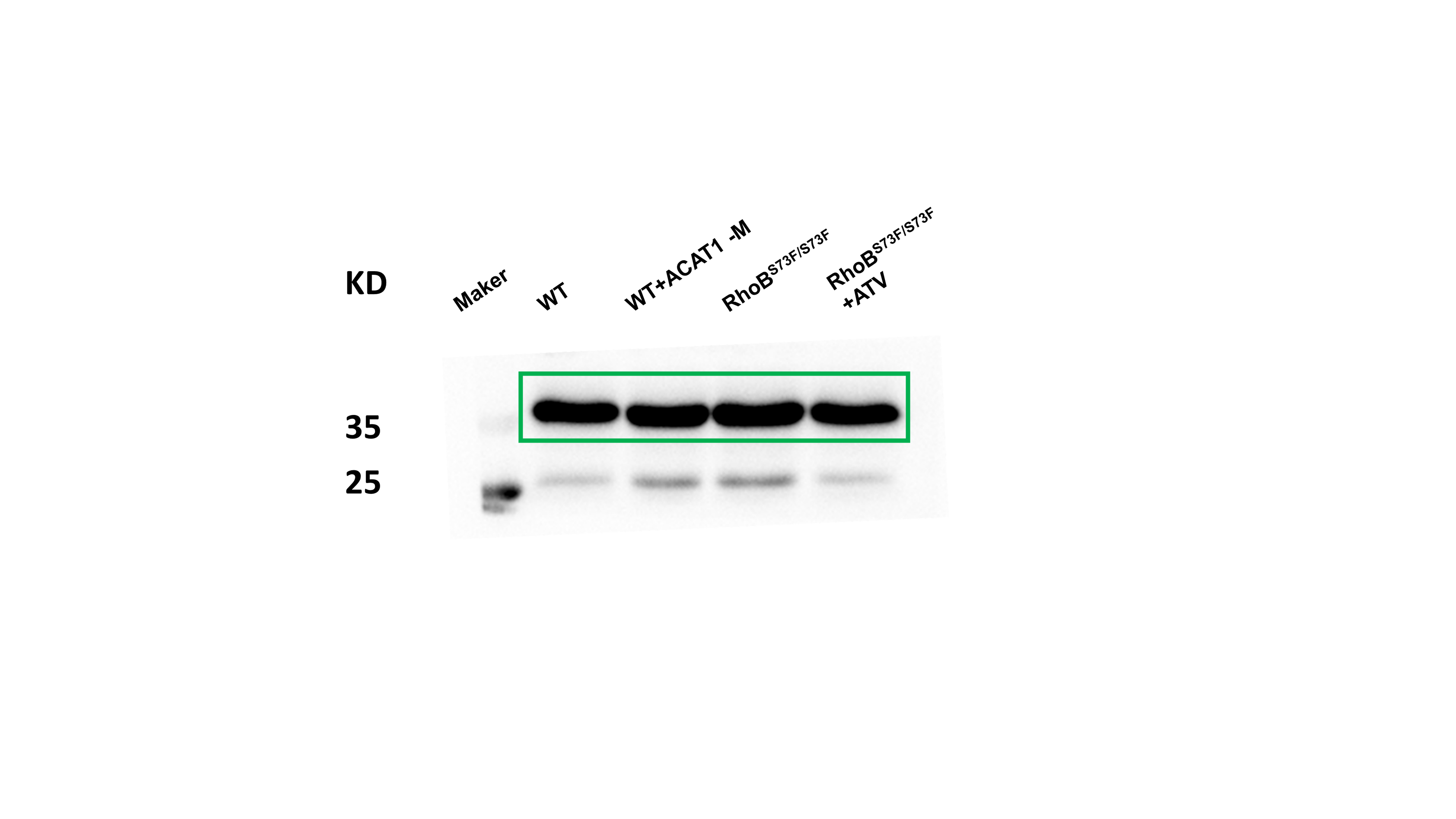

Supplement: Supplementary file 16 — Source data Fig. 6 [file 44321_2024_113_MOESM16_ESM.zip › Figure 6/6F/replicate/western Gapdh replicate (1).tif]
