# Supplementary figures and images for: The RhoB p.S73F mutation leads to cerebral palsy through dysregulation of lipid homeostasis (part 2 of 2)
Source: EMBO Mol Med. 2024 Jul 30;16(9):3. doi: 10.1038/s44321-024-00113-2 (PMC11393352; doi:10.1038/s44321-024-00113-2)

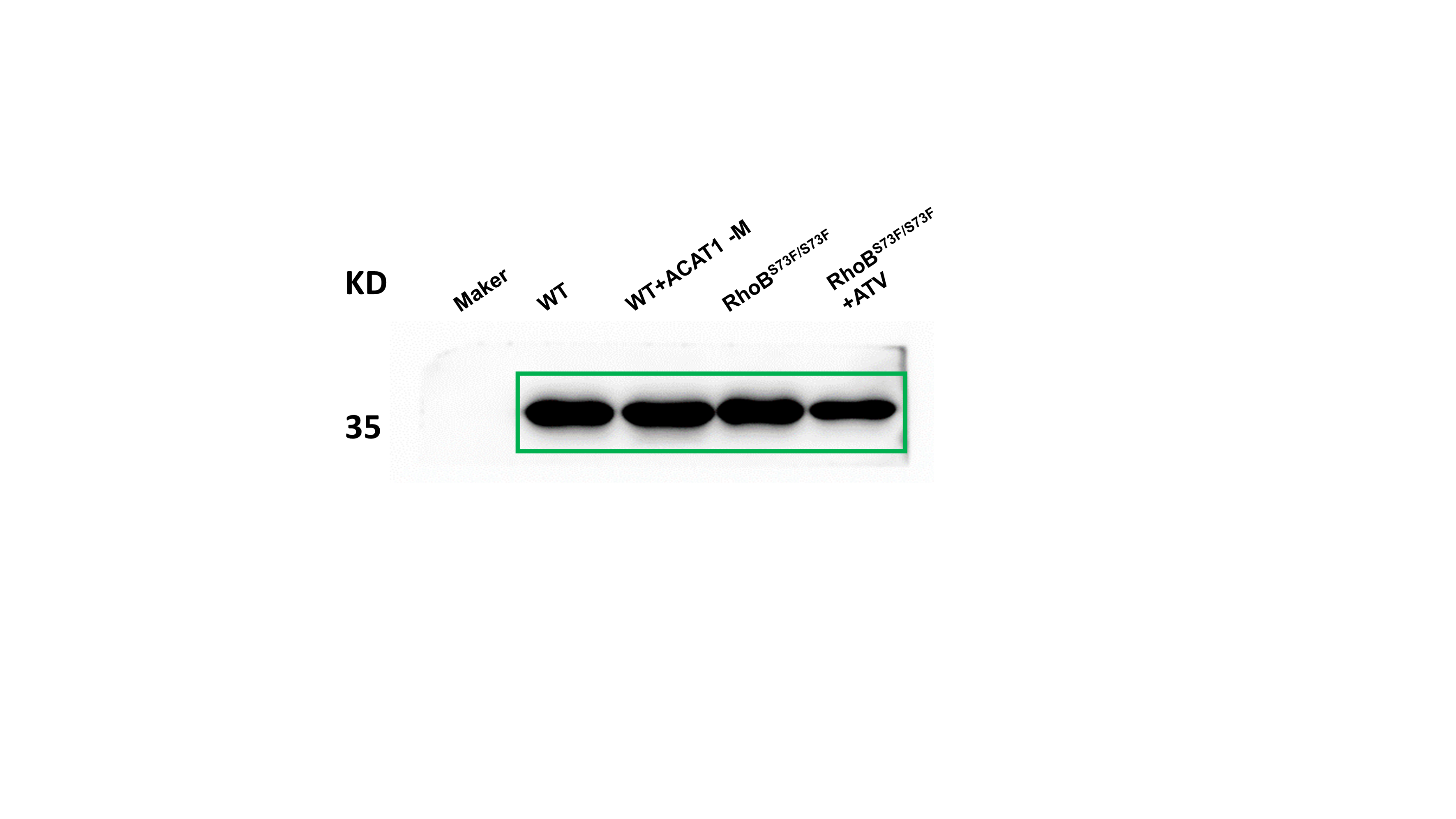

Supplement: Supplementary file 16 — Source data Fig. 6 [file 44321_2024_113_MOESM16_ESM.zip › Figure 6/6F/replicate/western Gapdh replicate (2).tif]

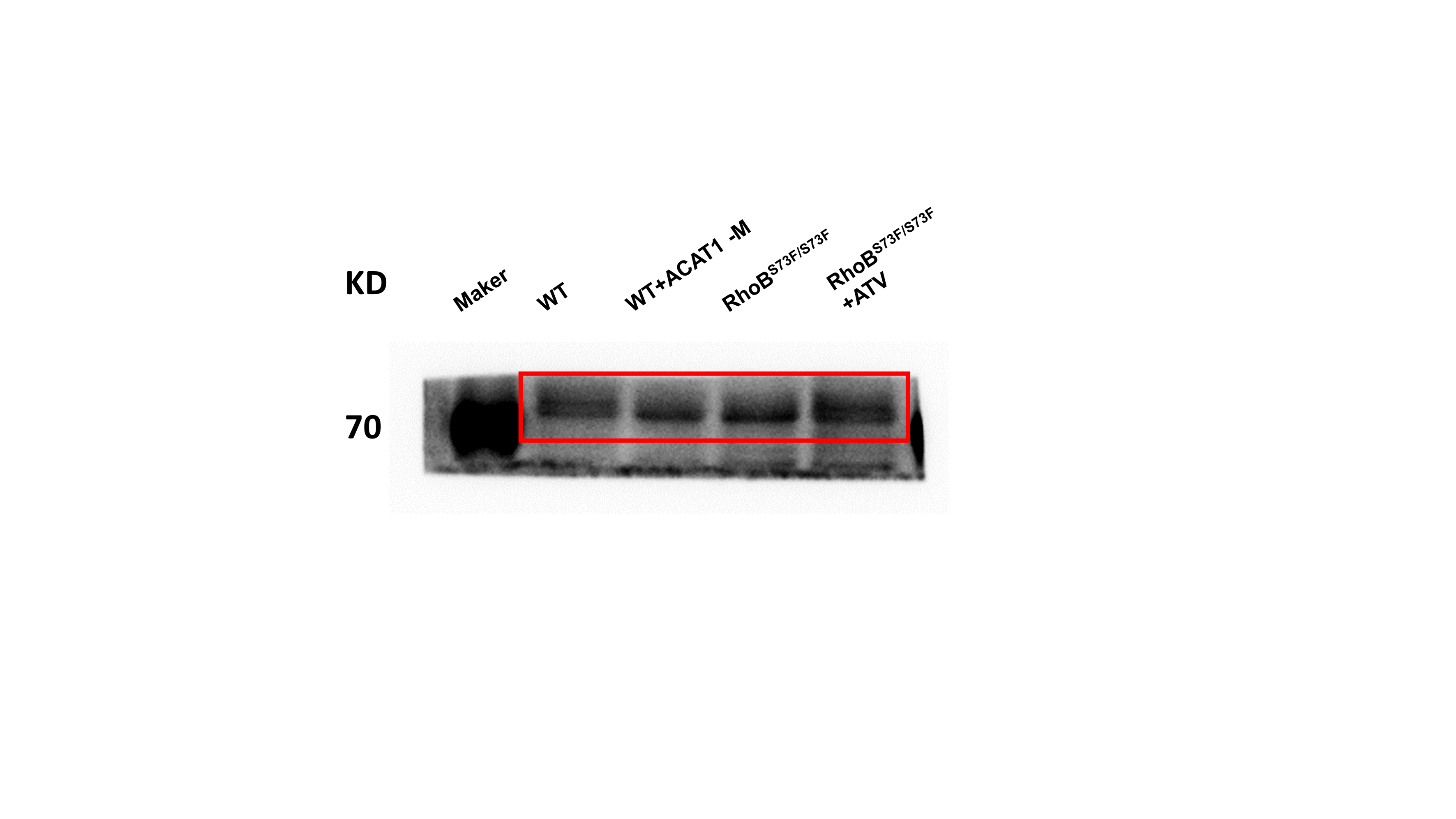

Supplement: Supplementary file 16 — Source data Fig. 6 [file 44321_2024_113_MOESM16_ESM.zip › Figure 6/6F/western Calpain1.tif]

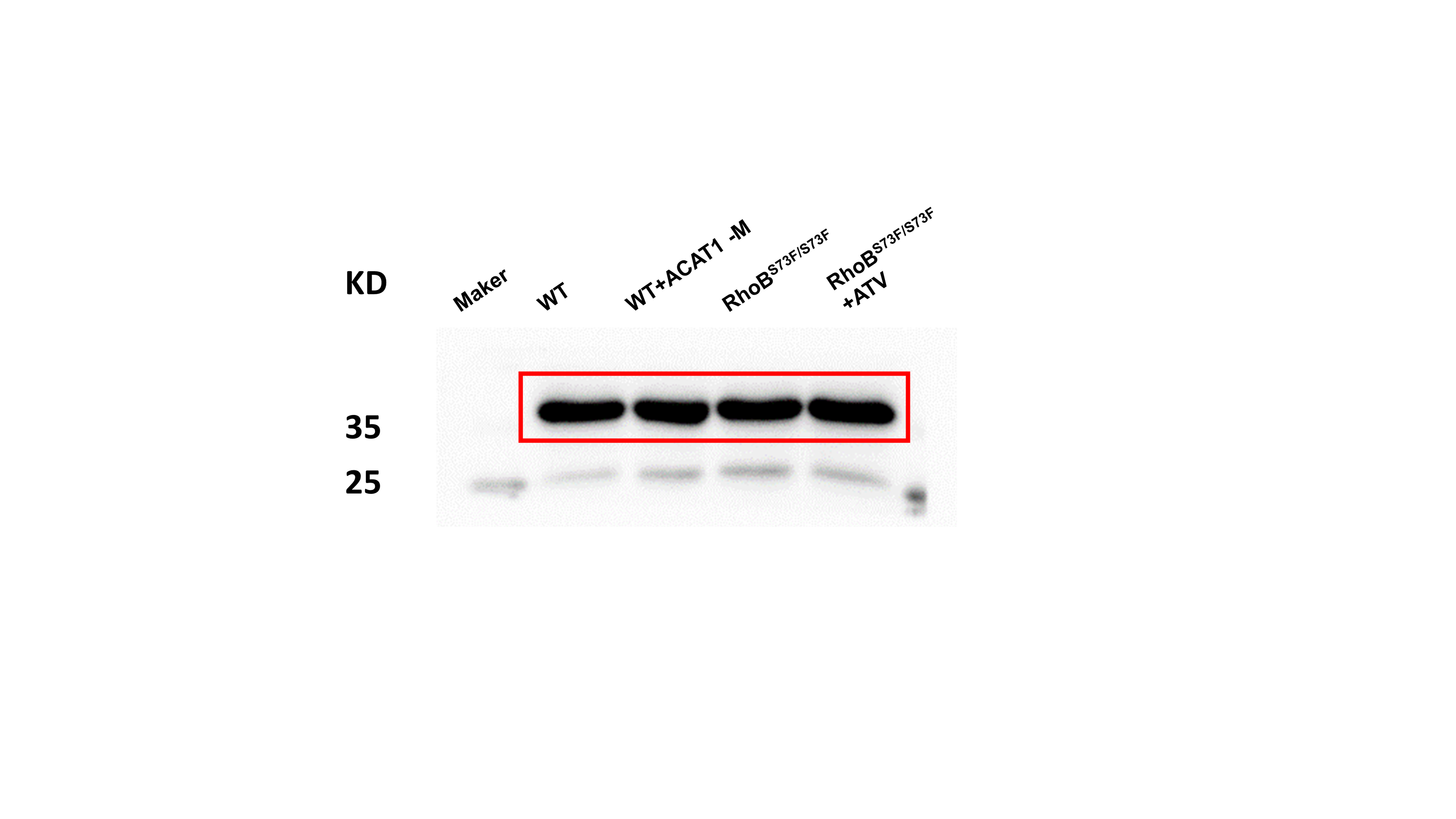

Supplement: Supplementary file 16 — Source data Fig. 6 [file 44321_2024_113_MOESM16_ESM.zip › Figure 6/6F/western Gapdh.tif]

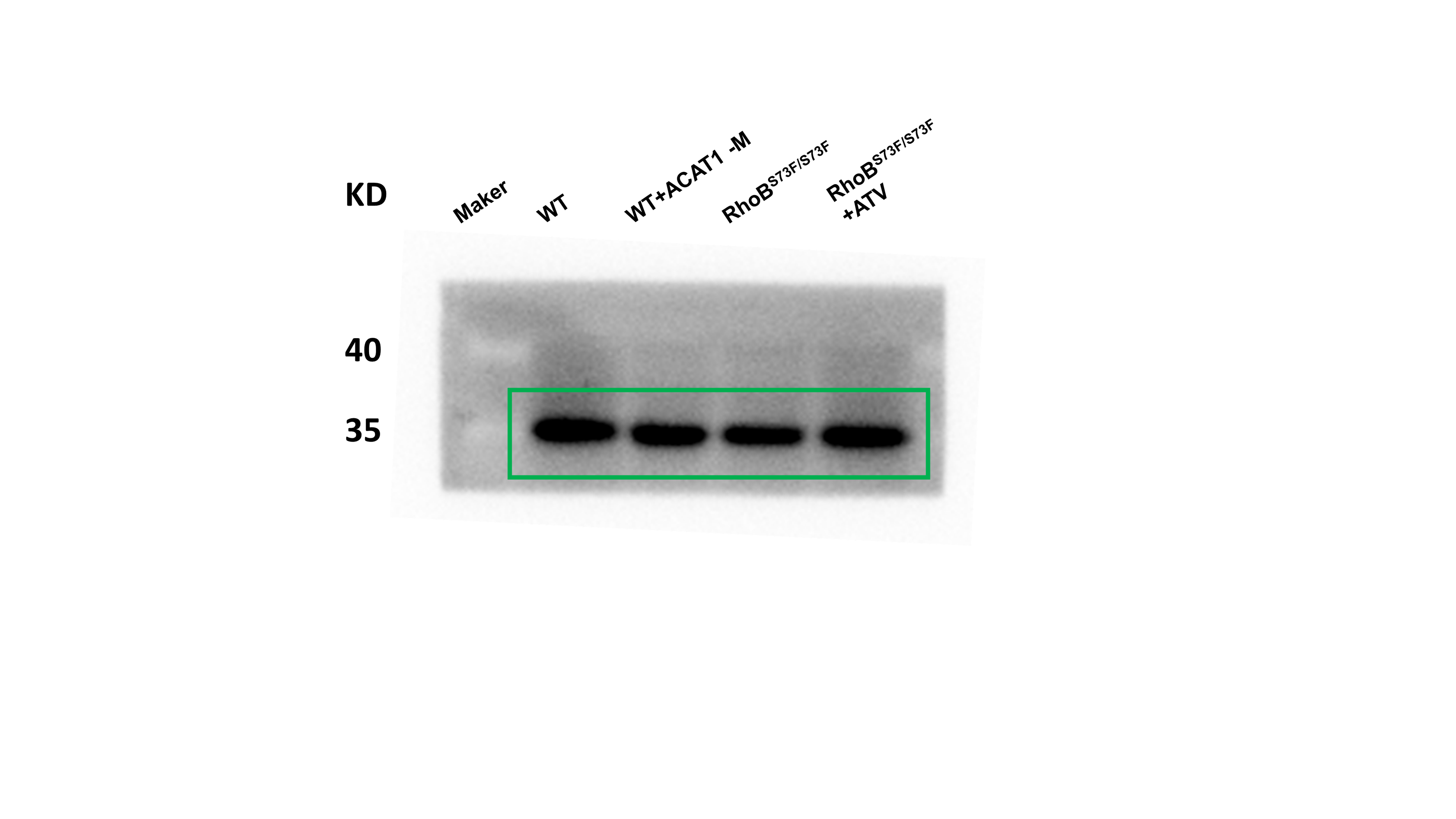

Supplement: Supplementary file 16 — Source data Fig. 6 [file 44321_2024_113_MOESM16_ESM.zip › Figure 6/6G/replicate/western Gapdh replicate (1).tif]

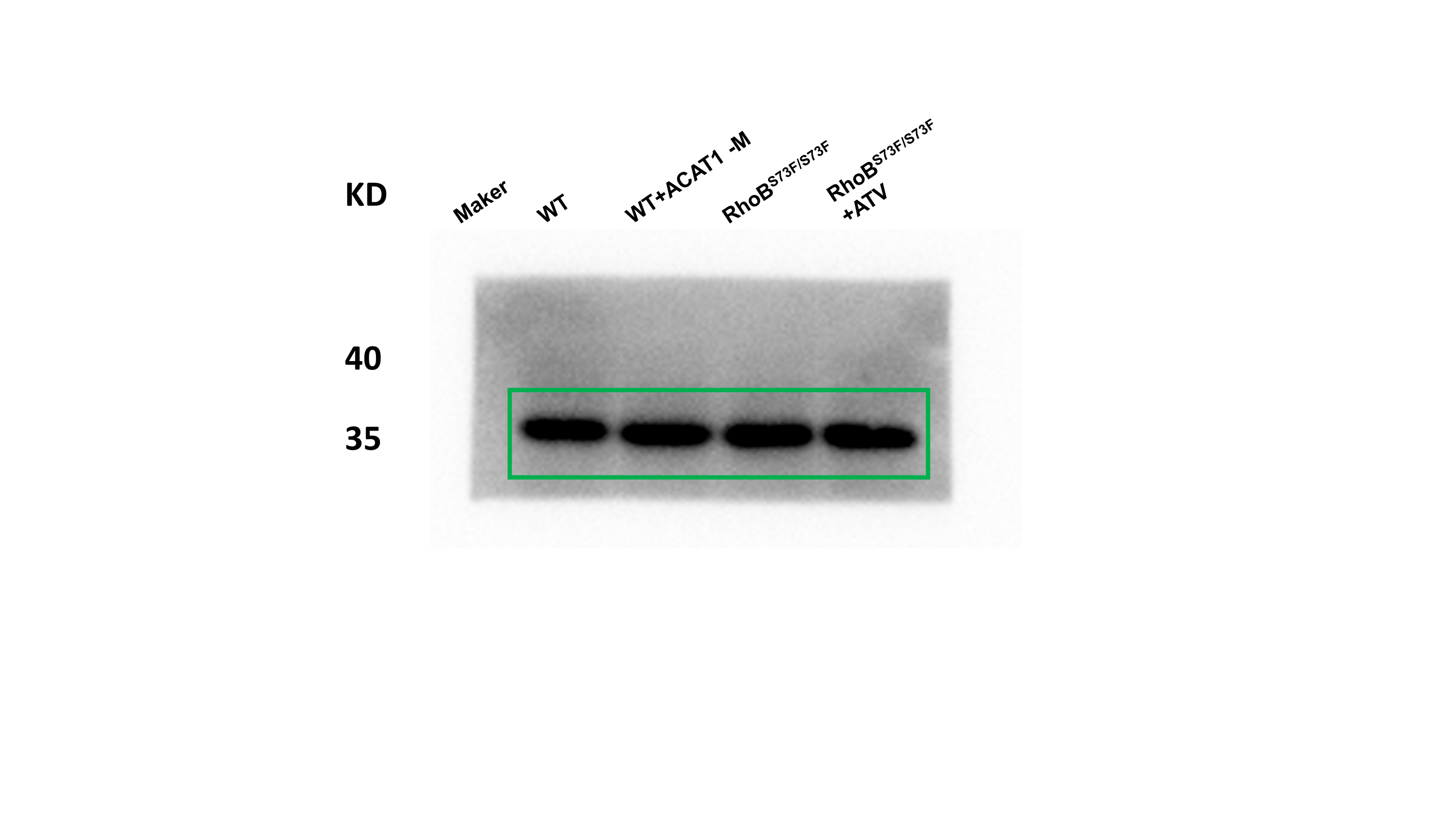

Supplement: Supplementary file 16 — Source data Fig. 6 [file 44321_2024_113_MOESM16_ESM.zip › Figure 6/6G/replicate/western Gapdh replicate (2).tif]

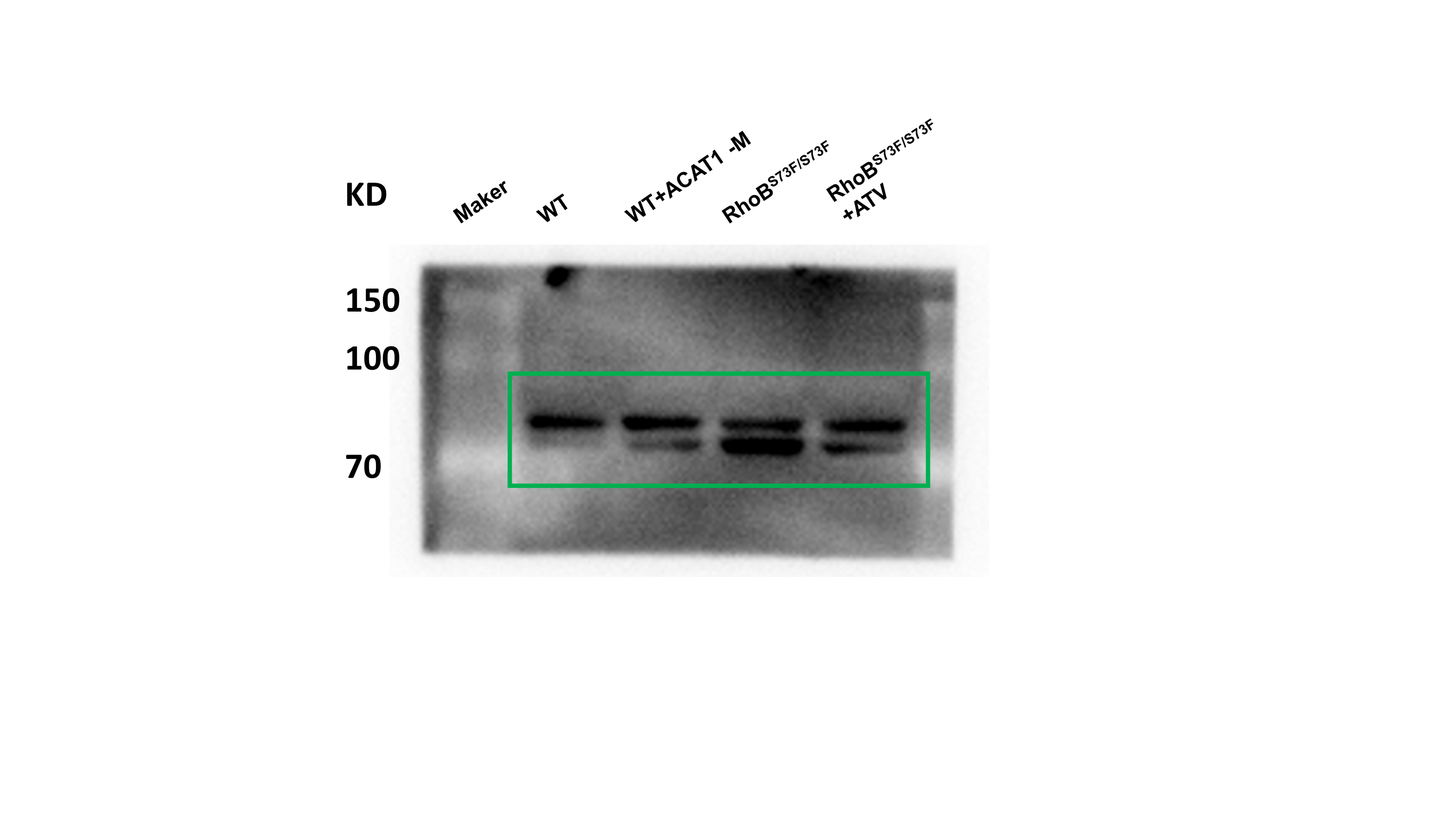

Supplement: Supplementary file 16 — Source data Fig. 6 [file 44321_2024_113_MOESM16_ESM.zip › Figure 6/6G/replicate/western GRP78 replicate (1).tif]

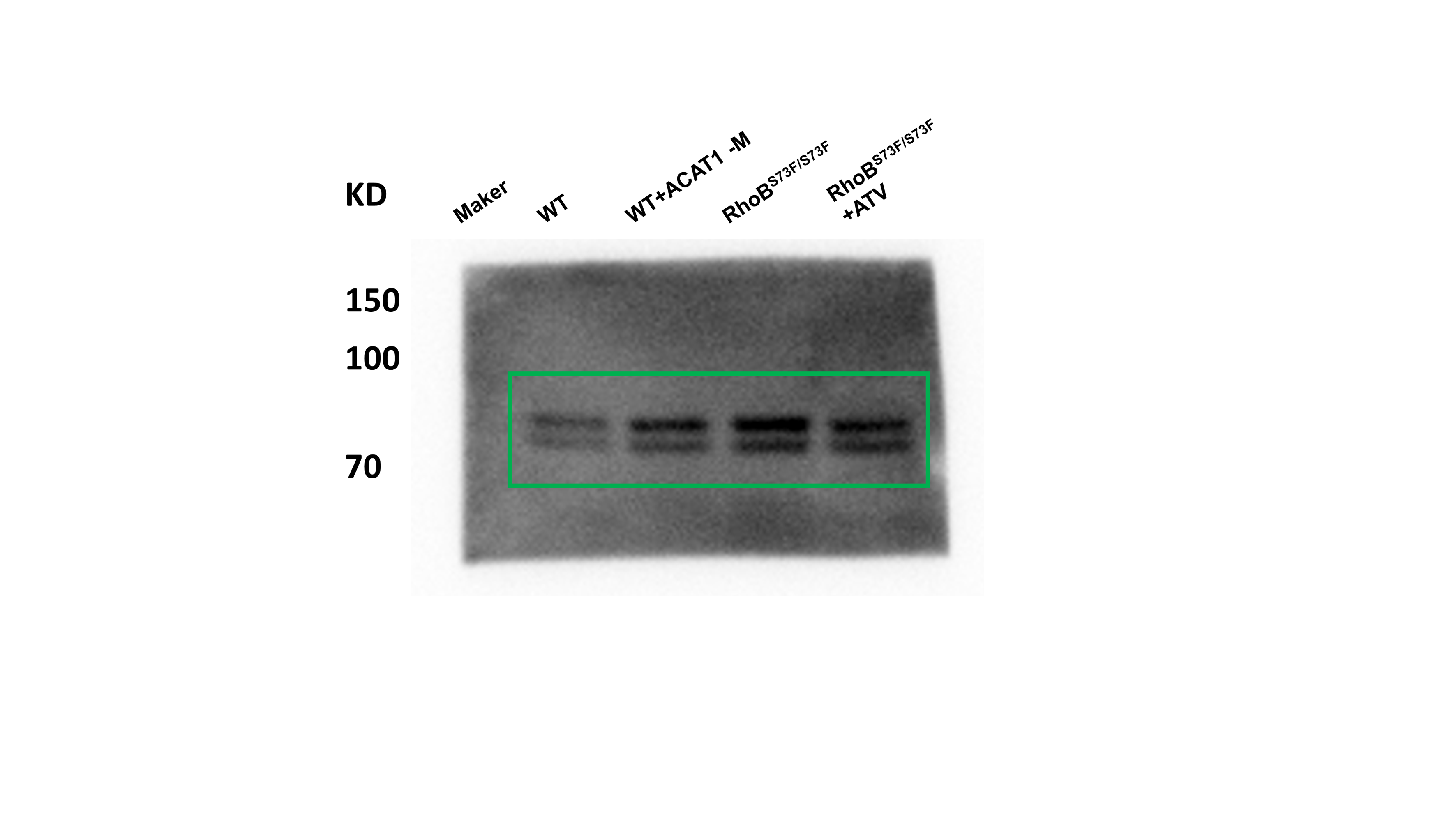

Supplement: Supplementary file 16 — Source data Fig. 6 [file 44321_2024_113_MOESM16_ESM.zip › Figure 6/6G/replicate/western GRP78 replicate (2).tif]

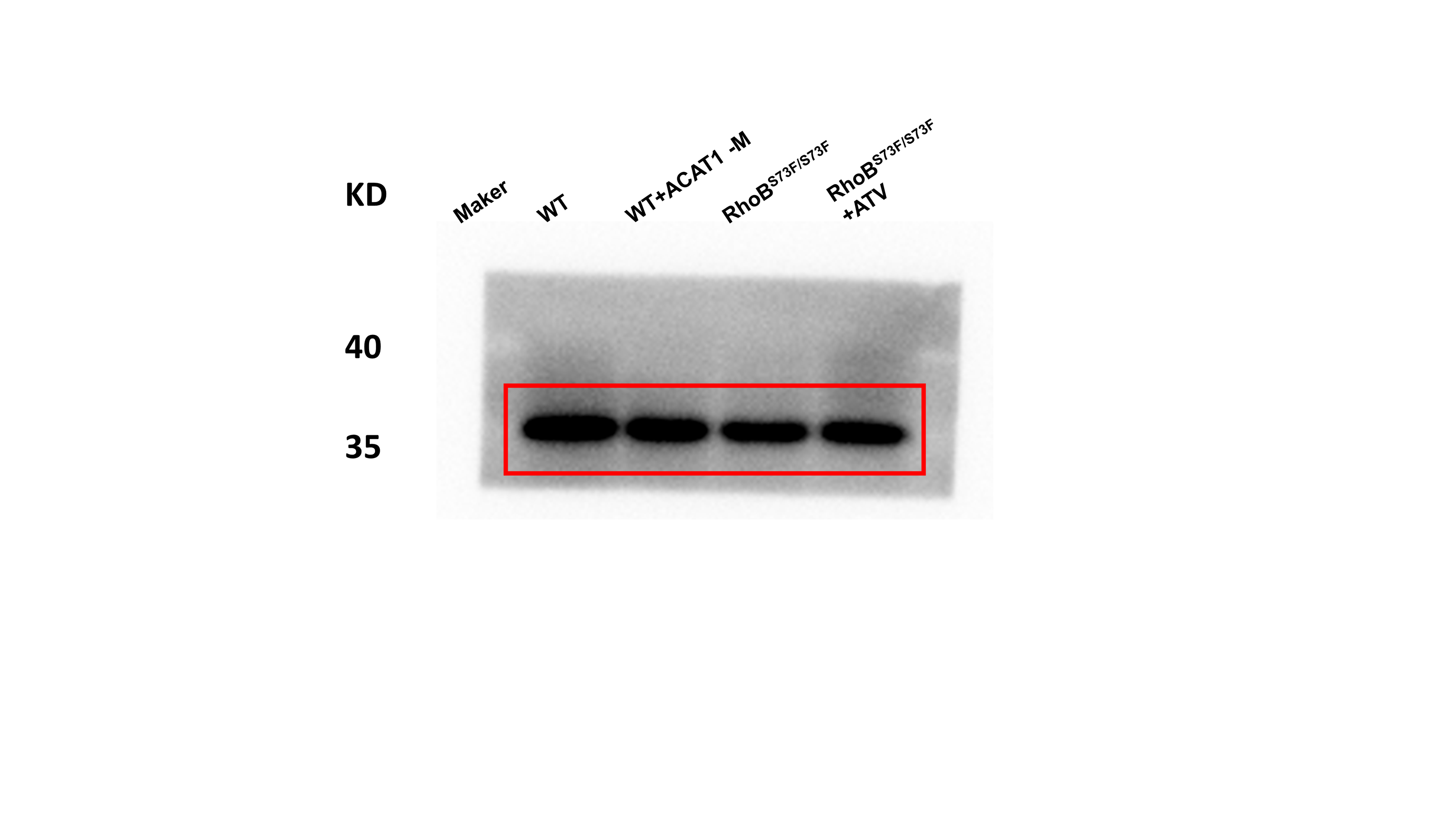

Supplement: Supplementary file 16 — Source data Fig. 6 [file 44321_2024_113_MOESM16_ESM.zip › Figure 6/6G/western Gapdh.tif]

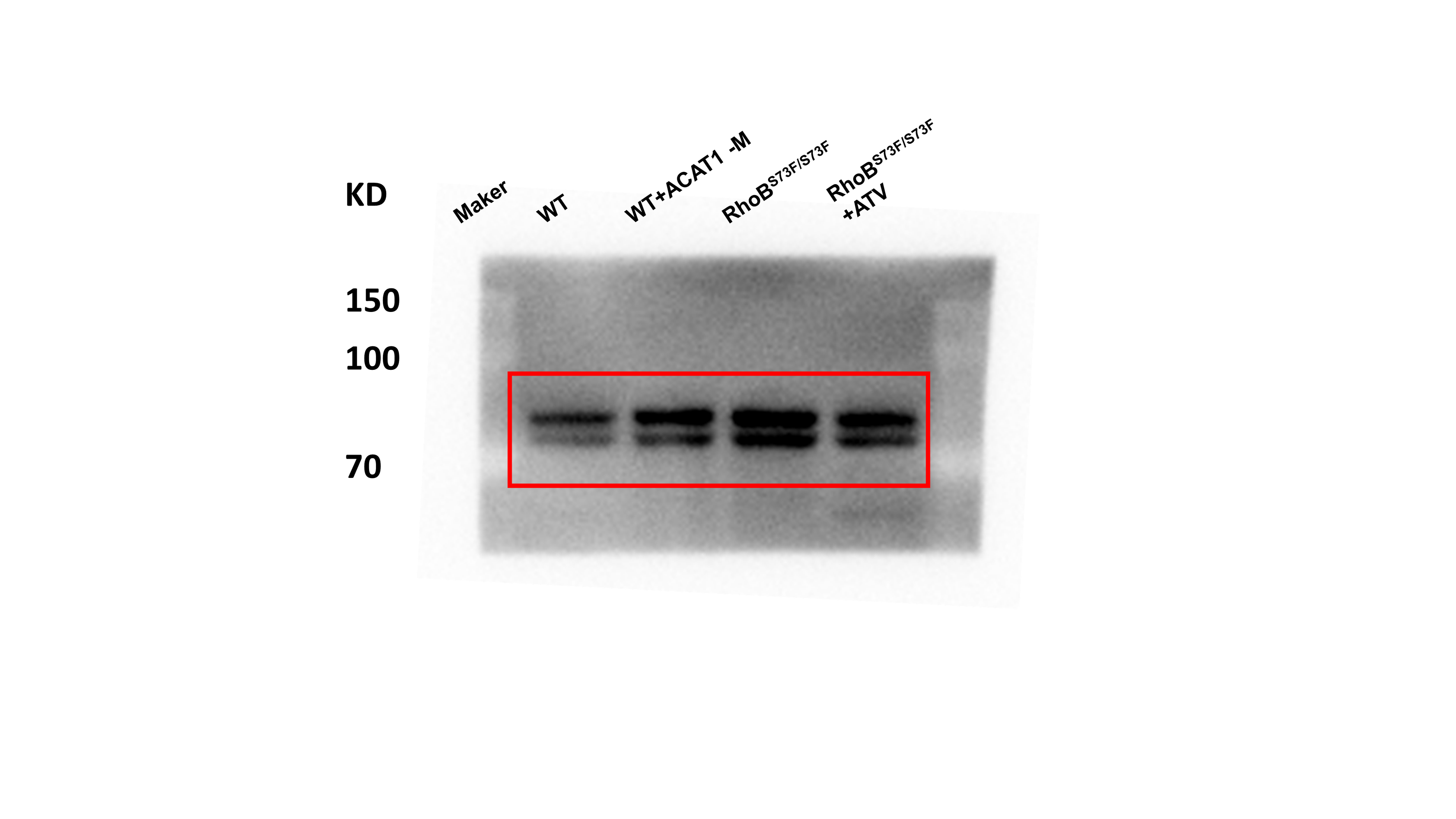

Supplement: Supplementary file 16 — Source data Fig. 6 [file 44321_2024_113_MOESM16_ESM.zip › Figure 6/6G/western GRP78.tif]
