# Supplementary material for: The RhoB p.S73F mutation leads to cerebral palsy through dysregulation of lipid homeostasis
Source: EMBO Mol Med. 2024 Jul 30;16(9):3. doi: 10.1038/s44321-024-00113-2 (PMC11393352; doi:10.1038/s44321-024-00113-2)
Supplement: Supplementary file 18 — Expanded View Figures [file 44321_2024_113_MOESM18_ESM.pdf]

Expanded View Figures

Figure EV1. (**A**) Summary of mutation efficiency and development of embryos after injection with the CRISPR/Cas9 system ($n = 30$ per group). (**B**) Sanger sequencing peak map of F0 generation rabbit ear clips. The red box indicates the site of the mutation. (**C**) Sanger sequencing results of RhoB gene mutation in newborn gene editing rabbits. Red denotes a mutant base; green denotes a PAM sequence; the underline indicates the design location of the SgRNA. (**D**) Summary of F1 generation rabbit breeding situation. F1 generation rabbits with a stable inheritance of the RhoB mutation were obtained. (**E**) Body weight curves of age-matched WT controls ($n = 3$) and $RhoB^{S73F/+}$ rabbits ($n = 3$). A significantly reduced body weight was observed in $RhoB^{S73F/+}$ rabbits. (**F**) Survival curves of age-matched WT controls ($n = 4$) and $RhoB^{S73F/+}$ rabbits ($n = 8$). A significantly reduced survival rate was observed in $RhoB^{S73F/+}$ rabbits. (**G**) Representative postural images of 12-week-old WT controls ($n = 3$) and $RhoB^{S73F/+}$ rabbits ($n = 3$). Severe motor and postural control issues were observed in $RhoB^{S73F/+}$ rabbits. (**H**, **I**) Representative gait trajectories (**H**) and gait analysis (**H**) of 12-week-old WT controls ($n = 5$) and $RhoB^{S73F/+}$ rabbits ($n = 5$). The significantly varied pace trajectory was observed in $RhoB^{S73F/+}$ rabbits. Red indicates front paw tracks; blue indicates hind paw tracks. The dataset includes measurements of stride length, step width, and step length of the hind limb footprint of rabbits during movement. See details on *P* values in Appendix Table S33. Data information: Data represent different numbers (*n*) of biological replicates. In (**I**), data were presented as mean ± SD (Unpaired two-tailed Student's *t*-tests). $^{****}P \leq 0.0001$. Source data are available online for this figure.

 © The Author(s)

A

	Zygotes, n	Two-cell, n (%)	Morula, n (%)	Blastocyst, n (%)	Mutant blastocyst, n (%)
Noninjection	30	29 (96.7)	28 (93.3)	26 (86.7)	0
Injection	30	28 (93.3)	27 (90)	25 (83.3)	25 (83.3)

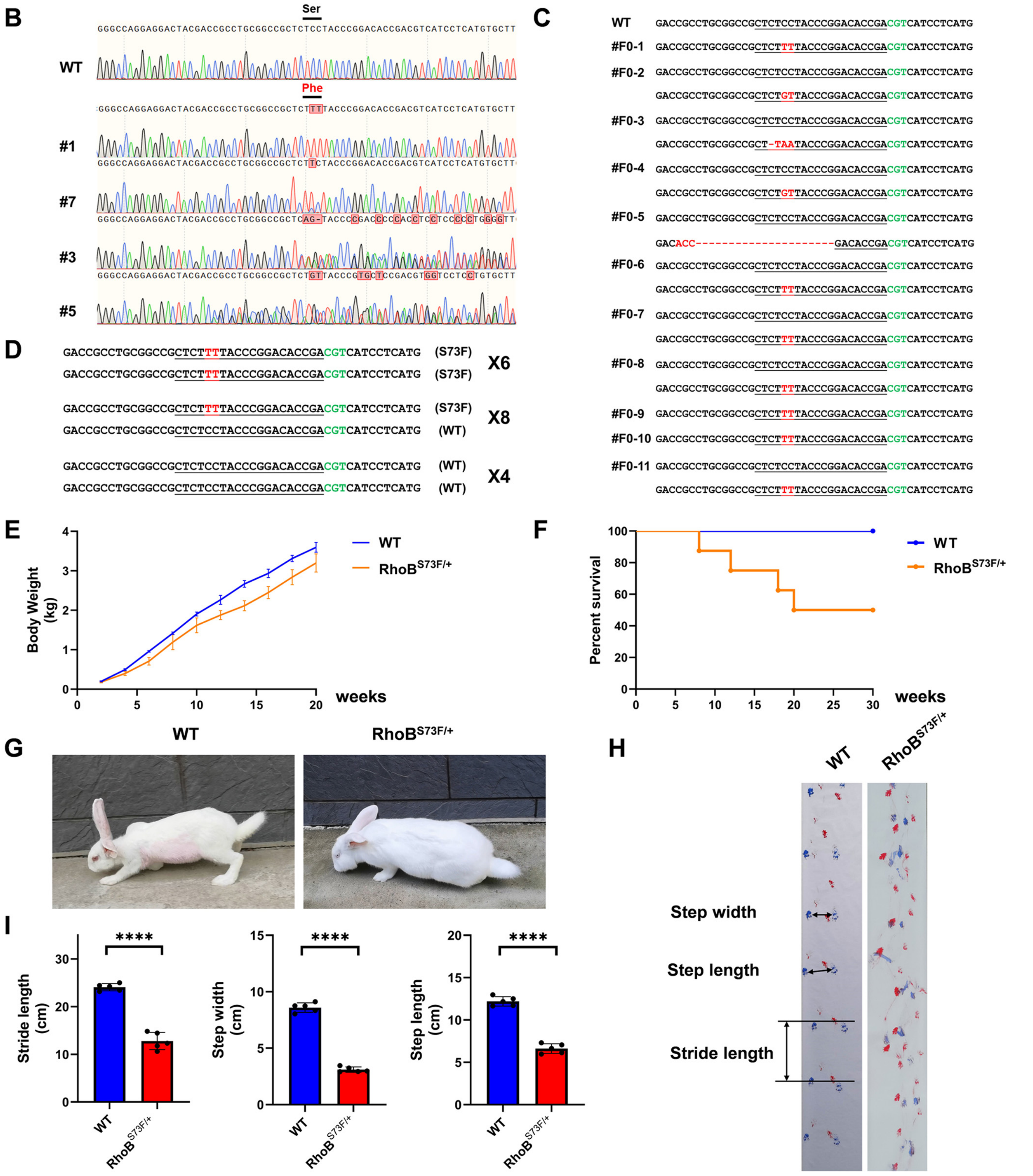

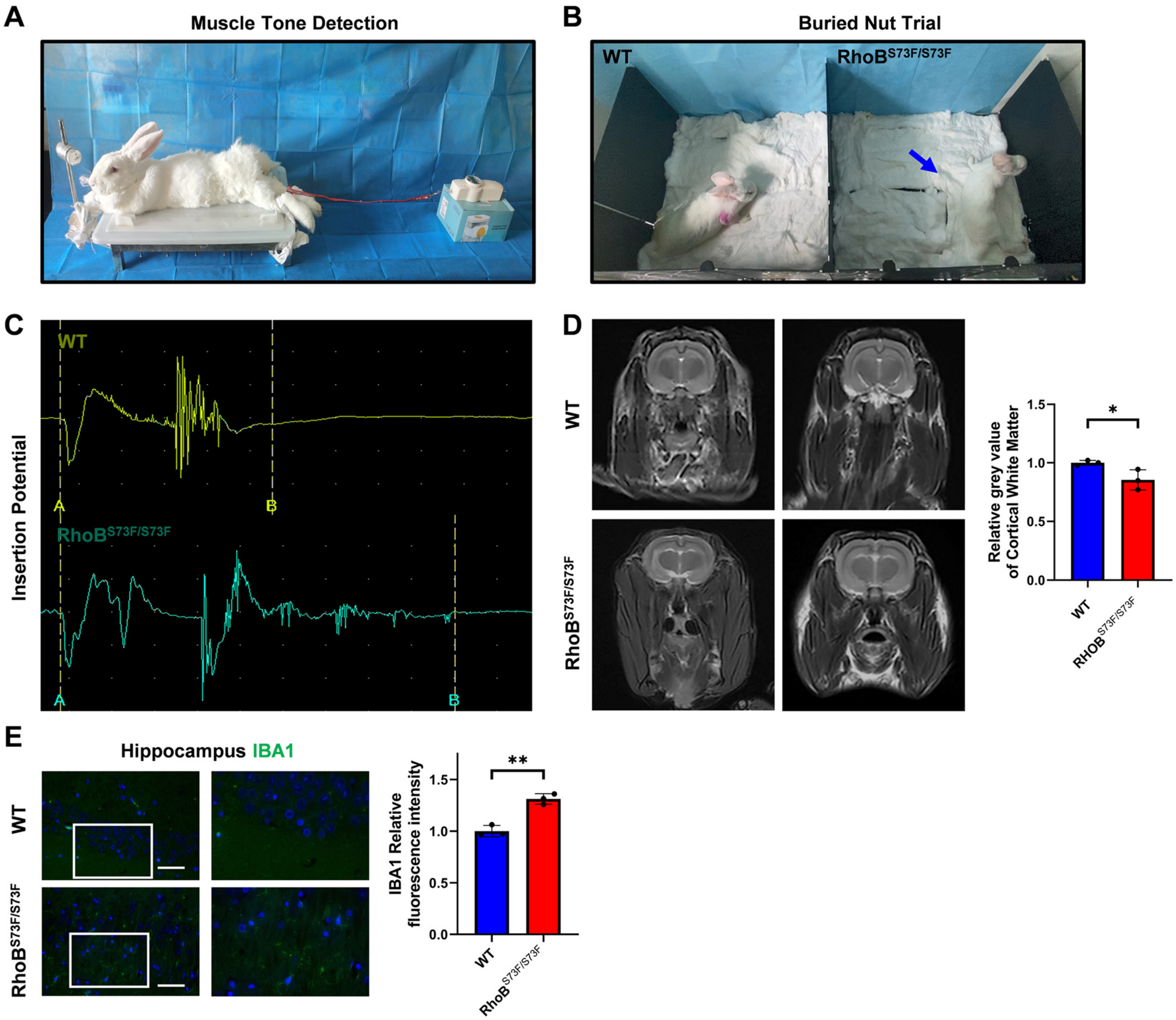

Figure EV2. (**A**) Representative schematic of lower limb muscle tone testing in rabbits. (**B**) Representative schematic of the buried nut trials in rabbits. (**C**) Representative needle electromyography images of insertion potential in the left hind limb gastrocnemius muscle from 12-week-old WT controls ($n = 3$) and RhoB$^{S73F/S73F}$ rabbits ($n = 3$). The significant prolongation of insertion potential was observed in RhoB$^{S73F/S73F}$ rabbits. Myoelectric Filters: 10 to 5 kHz; Scanning rate and sensitivity: 100 ms/div, 5 mV/div. Scale bars: 50 ms; 2.5 mV. (**D**) MRI detection images and relative gray value analysis of white matter in the brain from 12-week-old WT controls ($n = 3$) and RhoB$^{S73F/S73F}$ rabbits ($n = 3$). Reduced brain white matter density was observed in RhoB$^{S73F/S73F}$ rabbits. See details on *P* values in Appendix Table S34. (**E**) Immunofluorescence staining images and relative fluorescence intensity analysis of IBA1 (Green) in brain sections from 12-week-old WT controls ($n = 3$) and RhoB$^{S73F/S73F}$ rabbits ($n = 3$). The significantly activated microglia were observed in RhoB$^{S73F/S73F}$ rabbits. Nuclei DAPI-stained (Blue). Scale bars: 100 µm. See details on *P* values in Appendix Table S35. Data information: Data represent different numbers (n) of biological replicates. In (**D**, **E**), data were presented as mean ± SD (Unpaired two-tailed Student's *t*-tests). $^*P \leq 0.05$, $^{**}P \leq 0.01$. Source data are available online for this figure.

 © The Author(s)

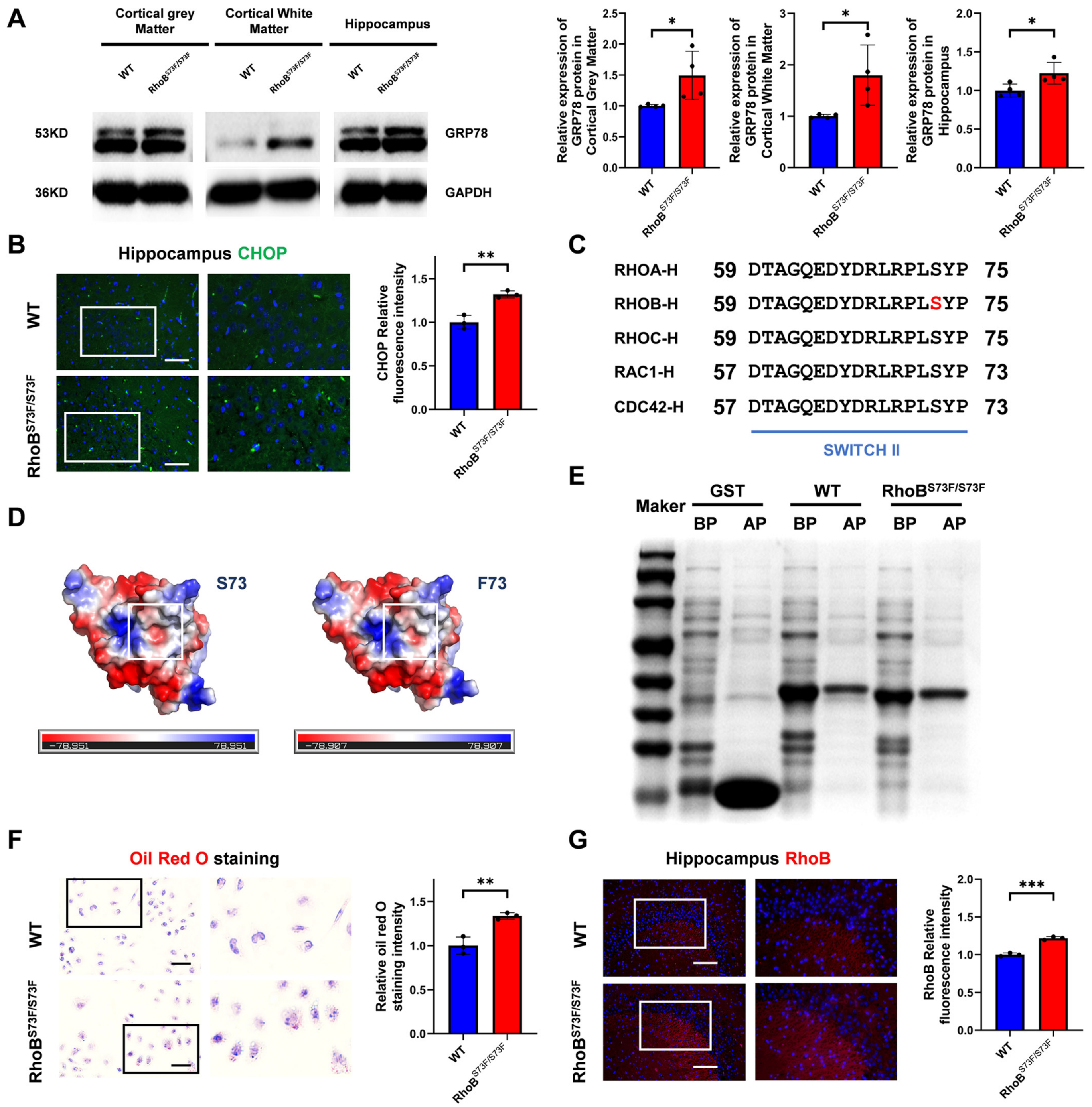
A
Cortical grey Matter
Cortical White Matter
Hippocampus
WT
RhoBS73F/S73F
53KD
36KD
GRP78
GAPDH
Relative expression of GRP78 protein in Cortical Grey Matter
Relative expression of GRP78 protein in Cortical White Matter
Relative expression of GRP78 protein in Hippocampus
B
Hippocampus CHOP
CHOP Relative fluorescence intensity
C
RHOA-H 59 DTAGQEDYDRLRPLSYP 75
RHOB-H 59 DTAGQEDYDRLRPLSYP 75
RHOC-H 59 DTAGQEDYDRLRPLSYP 75
RAC1-H 57 DTAGQEDYDRLRPLSYP 73
CDC42-H 57 DTAGQEDYDRLRPLSYP 73
SWITCH II
D
S73
F73
−78.951
78.951
−78.907
78.907
E
Maker
GST
BP
AP
F
Oil Red O staining
Relative oil red O staining intensity
G
Hippocampus RhoB
RhoB Relative fluorescence intensity

Figure EV3. (**A**) WB detection and relative expression analysis of GRP78 in the brain from 12-week-old WT controls ($n = 4$) and RhoB$^{S73F/S73F}$ rabbits ($n = 4$). The significantly upregulated GRP78 protein was observed in RhoB$^{S73F/S73F}$ rabbits. See details on *P* values in Appendix Table S36. (**B**) Immunofluorescence staining images and relative fluorescence intensity analysis of CHOP (Green) in brain sections from 12-week-old WT controls ($n = 3$) and RhoB$^{S73F/S73F}$ rabbits ($n = 3$). The significantly increased CHOP protein was observed in RhoB$^{S73F/S73F}$ rabbits. Nuclei DAPI-stained (Blue). Scale bars: 100 μm. See details on *P* values in Appendix Table S37. (**C**) Homologous family sequence alignment of the SWITCH II domain of RhoB protein. The Switch II structural domain of the RhoB gene is highly conserved within the homologous family. The mutant residue is showed in red. (**D**) Comparison of the surface potential of WT control and p.S73F mutant group of human-derived RhoB protein. The local surface potential change was observed in the RhoB$^{S73F/S73F}$ protein. Data from RCSB Protein Data Bank. (**E**) SDS-PAGE analysis for the purification of GST, WT, and RhoB$^{S73F/S73F}$ recombinant protein. GST indicates GST protein control; WT indicates GST-RhoB recombinant protein; RhoB$^{S73F/S73F}$ indicates GST-RhoBS$^{73F/S73F}$ mutant protein; BP indicates before purification; AP indicates after purification. (**F**) Oil red O staining images and relative intensity (OD value) analysis in WT controls ($n = 3$) and RhoB$^{S73F/S73F}$ cells ($n = 3$). Significant lipid droplet aggregation was observed in the RhoB$^{S73F/S73F}$ cells. Scale bars: 50 μm. See details on *P* values in Appendix Table S38. (**G**) Immunofluorescence staining images and relative fluorescence intensity analysis of RhoB (Red) in brain sections from 12-week-old WT controls ($n = 3$) and RhoB$^{S73F/S73F}$ rabbits ($n = 3$). The significantly upregulated RhoB protein was observed in RhoB$^{S73F/S73F}$ rabbits compared to the WT controls. Nuclei DAPI-stained (Blue). Scale bars: 200 μm. See details on *P* values in Appendix Table S39. Data information: Data represent different numbers (*n*) of biological replicates. In (**A**, **B**, **F, G**), data were presented as mean ± SD (Unpaired two-tailed Student's *t*-tests). $^{*}P \le 0.05$, $^{**}P \le 0.01$, $^{***}P \le 0.001$. Source data are available online for this figure.

 © The Author(s)
